# HPV, HBV, and HIV-1 Viral Integration Site Mapping: A Streamlined Workflow from NGS to Genomic Insights of Carcinogenesis

**DOI:** 10.3390/v16060975

**Published:** 2024-06-18

**Authors:** Jane Shen-Gunther, Acarizia Easley

**Affiliations:** 1 Gynecologic Oncology & Clinical Investigation, Department of Clinical Investigation, Brooke Army Medical Center, San Antonio, TX 78234, USA; 2 Department of Clinical Investigation, Brooke Army Medical Center, San Antonio, TX 78234, USA; acarizia@gmail.com

**Keywords:** bioinformatics, HBV, HIV-1, HPV, hybrid capture NGS, insertional mutagenesis, next-generation sequencing, oncovirus, virus taxonomy, virus database, viral mapping, virus integration

## Abstract

Viral integration within the host genome plays a pivotal role in carcinogenesis. Various disruptive mechanisms are involved, leading to genomic instability, mutations, and DNA damage. With next-generation sequencing (NGS), we can now precisely identify viral and host genomic breakpoints and chimeric sequences, which are useful for integration site analysis. In this study, we evaluated a commercial hybrid capture NGS panel specifically designed for detecting three key viruses: HPV, HBV, and HIV-1. We also tested workflows for Viral Hybrid Capture (VHC) and Viral Integration Site (VIS) analysis, leveraging customized viral databases in CLC Microbial Genomics. By analyzing sequenced data from virally infected cancer cell lines (including SiHa, HeLa, CaSki, C-33A, DoTc2, 2A3, SCC154 for HPV; 3B2, SNU-182 for HBV; and ACH-2 for HIV-1), we precisely pinpointed viral integration sites. The workflow also highlighted disrupted and neighboring human genes that may play a crucial role in tumor development. Our results included informative virus–host read mappings, genomic breakpoints, and integration circular plots. These visual representations enhance our understanding of the integration process. In conclusion, our seamless end-to-end workflow bridges the gap in understanding viral contributions to cancer development, paving the way for improved diagnostics and treatment strategies.

## 1. Introduction

In 1911, Peyton Rous isolated a transmissible agent from a large tumor on the breast of a Plymouth Rock hen [1,2]. His groundbreaking work demonstrated that malignant tumors may have infectious origins. The impact of his novel discovery ushered in the field of tumor virology which deepened our understanding of carcinogenesis by insertional mutagenesis [1,2]. Rous was eventually awarded the Nobel Prize in 1966, and the famed chicken retrovirus was eponymously named Rous sarcoma virus (RSV) [1,2]. Today, seven viruses have been classified as human carcinogens (Group 1) by the International Agency for Research on Cancer (IARC), which include Epstein–Barr virus (EBV); Hepatitis B virus (HBV), Hepatitis C virus (HCV), Human immunodeficiency virus type 1 (HIV-1), Human papillomavirus (HPV), Human T-cell lymphotropic virus type I, (HTLV-I), and Kaposi’s sarcoma-associated herpesvirus (KSHV) [3]. HIV-1 infection alone, interestingly, does not lead to cell transformation or immortalization. Instead, HIV-1 accelerates the process by interacting with oncoviruses, suppressing the immune system, and producing transferable HIV-1 proteins, thus acting as a co-factor in carcinogenesis [3,4].

Globally, the burden of cancer is staggering with an estimated incidence rate of 20 million new cases in 2022 with a projected increase to 35 million in 2050 [5]. One out of every eight cases are attributed to chronic infections. HPV and HBV are the two most common viral causes of cancer worldwide [5,6,7,8,9]. In 2020, there were 730,000 cancer cases attributable to HPV and 380,000 cancer cases attributable to HBV [5]. The most common cancers associated with HPV were cervical cancer (with 662,301 cases), followed by oropharyngeal and anogenital cancers. As for HBV, it was hepatocellular carcinoma (HCC). In 2022, the global population of people living with HIV (PLWH) was 39 million, and those acquiring new infections was 1.3 million [10]. With anti-viral treatment, life expectancy for HIV-infected persons has increased. However, among individuals co-infected with HPV and HBV, non-AIDS-defining cancers have become a concerning cause of mortality [4].

**Figure 1:**
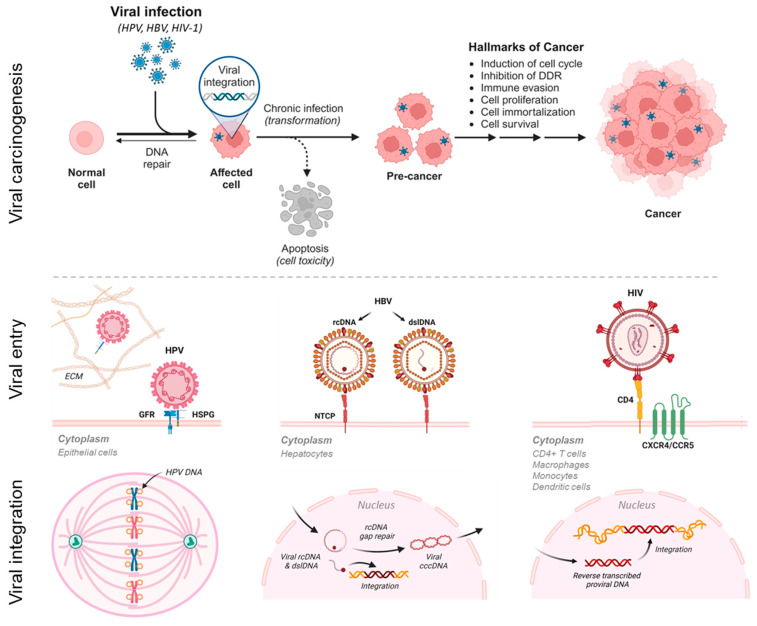
Viral carcinogenesis, viral entry, and viral–host genome integration. Hallmarks of viral carcinogenesis from integration to malignant transformation for human oncoviruses, i.e., HPV, HBV, and HIV-1 (accelerates oncovirus-mediated carcinogenesis). Viral entry into mammalian cells is host- and surface-receptor-specific [4,11,12]. The modes of viral–host integration differ, respectively, among HPV, HBV, and HIV-1 to include faulty viral partitioning to daughter cells during mitosis, non-homologous end-joining (NHEJ). or micro-homology mediated end-joining (MMEJ) at double-strand DNA breaks, and provirus insertion [13,14,15,16,17,18,19]. cccDNA, covalently closed circular DNA; CCRS, chemokine receptor type 5; CXCR4, chemokine receptor type 4; DDR, DNA damage repair; dslDNA, double-stranded linear DNA; ECM, extracellular matrix; GFR, growth factor receptor; HSPG, heparin sulfate proteoglycan; NTCP, Na+-taurocholate co-transporting polypeptide; and rcDNA, relaxed circular DNA (figure created with BioRender.com).

The journey from viral infection to malignant transformation of the host cell is complicated and disparate for HPV, HBV, and HIV-1 (Figure 1). Typically, these three viruses display host cell specificity, requiring the binding to specific cell surface proteins for entry [4,11,12]. After traversing the cytoplasm, the viral genomes enter the nucleus for replication (Figure 1). Viral DNA integration into the host genome, whether accidental or deliberate, can disrupt or alter the function of host cancer-associated genes and neighboring genes, ultimately leading to malignant transformation. The mechanism of integration for each virus is briefly described here and shown in Figure 1. During host cell division, the HPV circular genome tethers like a “hitchhiker” on sister chromatids [13,14]. The viral genome then unwinds bidirectionally, replicates, and partitions equally into the daughter cells [15,16]. Such intimate “liaisons” between viral and host DNA result in accidental integration at vulnerable sites (e.g., open chromatin and common fragile sites) [15,16]. For HBV, after virion entry into the hepatocyte, the relaxed circular DNA (rcDNA) and double-stranded linear DNA (dslDNA) traverse into the nucleus for conversion into covalently closed circular (cccDNA) [17]. Only the dslDNA integrates randomly at double-stranded DNA (dsDNA) breaks in the host genome through non-homologous end-joining (NHEJ) or micro-homology-mediated end-joining (MMEJ) [17,18]. The integrated viral DNA leads to viral persistence, pathogenesis, and carcinogenesis [17,18]. For HIV-1, upon entering the immune cell, the RNA genome of the virion undergoes reverse transcription, resulting in a dsDNA (provirus) [19]. The provirus then enters the nucleus for insertion into the host genome at random sites by the HIV-1 integrase enzyme [19]. 

Viral hybrid capture next-generation sequencing (hyb-cap NGS) is a widely used method for targeted or whole-genome sequencing [20]. It also enables the capture of virus–host chimeric reads, useful for identifying genomic breakpoints, especially for integration site analysis [21]. In 2022, a commercial hyb-cap NGS kit for HPV, HBV, and HIV-1 became available [22]. The anticipated benefits of using pre-designed, virus-specific probes for targeted sequencing include eliminating the effort involved in probe design, ensuring quality assurance, and standardizing protocols. As for post-sequencing analysis, our prior study demonstrated the efficiency of the Viral Hybrid Capture (VHC) and Viral Integration Site (VIS) workflows within the CLC Microbial Genomics Module (CLC MGM) for HPV integration site analysis [21]. Our current study builds upon this previous work. Attention was centered on the three oncoviruses with the highest global prevalence. We evaluated a commercial viral hyb-cap NGS kit and the VHC/VIS workflows for HPV, HBV, and HIV-1 integration analysis. Customized genomic databases were constructed to cover HBV and HIV-1. The results confirmed the effectiveness of a comprehensive laboratory pipeline that identifies viral integration sites and their impact on host genes. This process enhances our understanding of cancer development and facilitates the identification of diagnostic, prognostic, and therapeutic markers.

## 2. Materials and Methods

### 2.1. NGS Dataset of HPV-, HBV-, and HIV-Positive Cell Lines

Cell lines for cancer of the cervix (SiHa, HeLa, CaSki, C33-A, and DoTc2), hypopharynx (2A3), tongue (UPCI:SCC154 or SCC154), liver (Hep3B or 3B2.1-7, and SNU-182), and synthetic HIV-1 RNA plasmid (ATCC VR-3245SD) were acquired from the American Type Culture Collection (ATCC, Manassas, VA, USA) for testing [23]. The cell type, primary tumor site, and viral genotypes are shown in Table 1. The cells were cultured in media and conditions as prescribed by ATCC [23]. Briefly, cells were cultured in flasks with the following base medium supplemented with fetal bovine serum (FBS) to a final concentration of 10%, and 1% penicillin–streptomycin solution (ATCC). EMEM medium (ATCC) was used to grow SiHa, HeLa, C-33A, SCC154, and 3B2 cells. DMEM medium (ATCC) was used to culture DoTc2 and 2A3; and RPMI-1640 media (ATCC) was used to culture CaSki and SNU-182 cells. Cells were grown at 37 °C in a CO_2_ incubator until reaching 80–90% confluence prior to harvesting. All cells were utilized within the first 10 cell culture passages. After cellular DNA extraction, the genomic DNA (gDNA) (20 µL with concentration of ≥20 ng/µL) was submitted to Qiagen Genomic Services (QIAGEN, Germantown, MD, USA) for viral hyb-cap NGS. The QIAseq xHYB Viral STI Panel (QIAGEN, Germantown, MD, USA) was used for gDNA library target enrichment and genotyping HPV (19 types: 16, 18, 26, 31, 33, 35, 39, 45, 51, 52, 53, 56, 58, 59, 66, 68, 68a, 73, and 82), HBV, and HIV-1 [22]. Per manufacturer’s protocol, the gDNA NGS libraries were hybridized overnight to the hyb-cap panel of probes. The probe-target hybrids were then bound to streptavidin-coated magnetic beads and washed for removal of unbound library fragments. The target enriched libraries were amplified and paired-end-sequenced on the MiSeq sequencer (Illumina, San Diego, CA, USA). 

The synthetic HIV-1 RNA plasmids were reverse-transcribed to cDNA using SuperScript™ III First-Strand Synthesis SuperMix (Invitrogen, Waltham, MA, USA) according to the manufacturer’s instructions prior to hyb-cap NGS. 

We supplemented our samples with sequencing data from a representative HIV-1 latently infected cell line (ACH-2) (Table 1) [25]. The dataset is available from NCBI SRA under BioProject accession number, PRJNA524421 https://www.ncbi.nlm.nih.gov/sra (accessed on 5 January 2024) [25,26]. The raw sequencing files and metadata were downloaded into CLC Genomics workbench using the “Search for Reads in SRA” tool under Sequence Read Archive (SRA) Study (SRP187583) and Run (SRR8670572) accession numbers (AN) [26]. The files were imported into the VHC/VIS workflows for viral sequence analysis. 

### 2.2. Customized HPV, HBV, and HIV-1 Reference Databases for CLC Workflows and Tools

**Figure 2:**
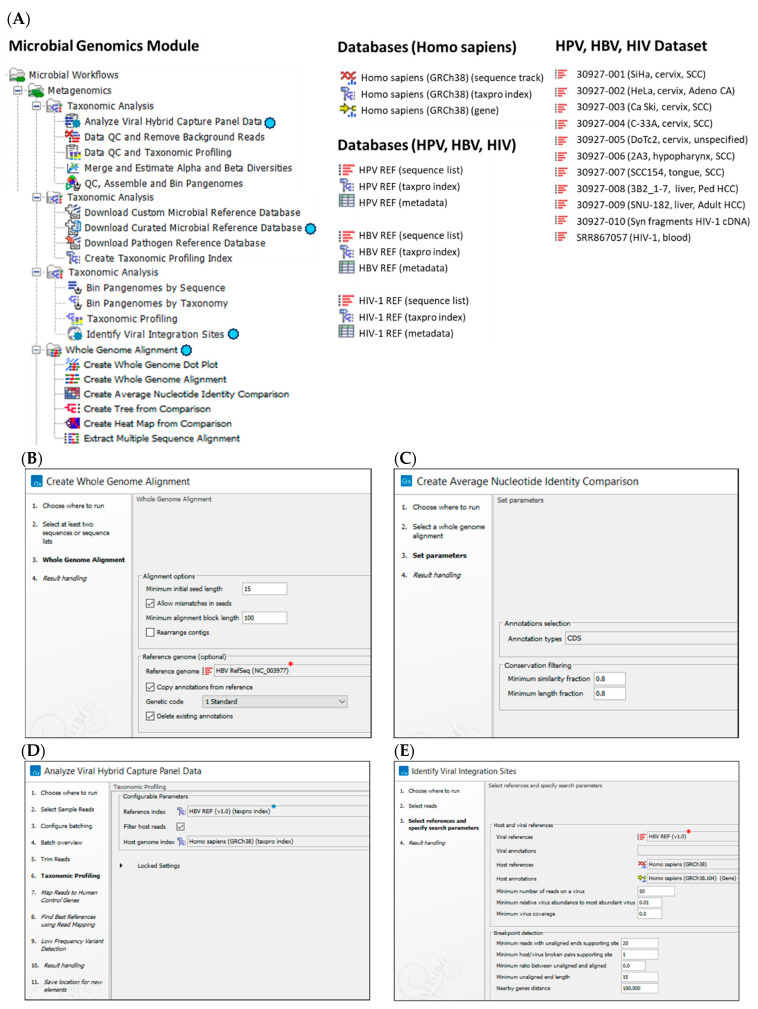
Bioinformatics methods. (**A**) CLC Microbial Genomics Module, databases and dataset used for Whole-Genome Alignment (WGA), Viral Hybrid Capture (VHC) data analysis, and Viral Integration Site (VIS) analysis. Primary workflows and tools used for this study are designated by the virus icon (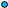); (**B**) WGA workflow steps (1–4) with user-defined parameter settings for WGA and annotation, e.g., HBV RefSeq (*) genome; (**C**) Create Average Nucleotide Identity Comparison workflow inputs the WGA file for quantification of the similarity between genomes, and outputs a pairwise comparison matrix; (**D**) VHC workflow steps (1–11) with user-defined parameter settings for Taxonomic Profiling (*), e.g., HBV reference index and host genome index; (**E**) VIS workflow steps (1–4) with selected HBV (*) and host reference genome databases and user-defined search parameters entered for this study.

HPV is a small, dsDNA virus consisting of approximately 8000 base pairs (bp), which encode six early genes (*E1*, *E2*, *E4*, *E5*, *E6*, and *E7*) and two late genes (*L1* and *L2*) [21]. Customized HPV reference (*n* = 219) and variant (*n* = 139) genome databases previously constructed were downloaded using the “Download Curated Microbial Reference Database” tool within the CLC Microbial Genomics Module (CLC MGM) of CLC Genomics Workbench Premium 23.0.4 (Redwood City, CA, USA) [27]. Two formats (taxonomic profiling index and sequence list) were downloaded and incorporated into the VHC and VIS workflows. The names of the databases in *index* and *list* formats, respectively, were: (1) “HPV REF_taxpro_index” and “HPV REF” for HPV reference genomes, and (2) “HPV VAR_taxpro_index” and “HPV VAR” for HPV variant genomes (Figure 2A) [21].

HBV is a small, enveloped DNA virus with a genome length of ~3200 bp. HBV exists in two primary forms (i.e., rcDNA and dslDNA), composing 90% and 10% of virions, respectively (Figure 1) [17]. The genome encodes four open reading frames, designated C (capsid protein), P (polymerase), S (surface proteins), and X (regulatory protein) [28]. To construct the customized database, HBV complete genomes, genotypes A–J (*n* = 268), were identified in NCBI Virus https://www.ncbi.nlm.nih.gov/labs/virus/vssi/#/ (accessed on 25 December 2023) for metadata (CSV) download [29]. The associated GenBank files (GB) were downloaded from NCBI Nucleotide https://www.ncbi.nlm.nih.gov/nuccore (accessed on 25 December 2023) [30]. The files from human (*n* = 268) and animal (*n* = 27) hosts were imported into CLC MGM for customization and use as separate databases. Customization involved creation of an author-defined, clinically relevant, common seven-level taxonomic nomenclature for each HBV genome file. The current HBV taxonomic nomenclature with nine primary ranks by International Committee on Taxonomy of Viruses (ICTV) 2022 Release https://ictv.global/msl (accessed on 25 December 2023) is as follows: Realm: Riboviria; Kingdom: Pararnavirae; Phylum: Artverviricota; Class: Revtraviricetes; Order: Blubervirales; Family: Hepadnaviridae; Genus: Orthohepadnavirus; and Species: Hepatitus B virus [31]. To deepen the taxonomic depth to sub-species, we created a customized taxonomy based on the attributes of the Baltimore classification and genotype/sub-lineage nomenclature [32,33]. Specifically, we defined our seven-level taxonomic ranks as: Virus_nucleic acid type; Family; Genus; Species; Genotype; Sub-lineage, and three-letter Country code [34]. For example, the taxonomy of HBV genotype A, sub-lineage 1 from a blood sample collected in Martinique (Accession: HE974362) was annotated as “Virus_dsDNA-RT_env; Hepadnaviridae; Orthohepadnavirus; HBV; A; 1; MTQ”. The customized taxonomy created in a metadata file replaced the original taxonomy of the sequence file for downstream applications as described in Section 2.3. For genome sequences devoid of a sub-lineage, the reserved space in the seven-level taxonomic nomenclature was left blank. 

HIV-1 is a retrovirus with a genome consisting of two identical, single-stranded ~9300 bp RNA molecules [35]. The genome encodes nine genes categorized according to three major protein products: (1) structural proteins: gag, pol, and env, (2) essential regulatory proteins: tat and rev, and (3) accessory regulatory proteins: nef, vpr, vif, and vpu [35]. The Los Alamos National Laboratory (LANL) HIV Databases (https://www.hiv.lanl.gov/content/index) were accessed on 22 January 2024 to obtain the NCBI GenBank AN for HIV-1/SIV reference genomes [36]. The AN were entered into the NCBI Nucleotide and NCBI Virus repositories for retrieval of GenBank (GB) and metadata (CSV) files (*n* = 53), respectively. HIV-1 is subcategorized as four groups (M, N, O, and P). The M group is further divided into nine subtypes (A through L) [36]. The files for human (*n* = 53) and animal (*n* = 27) hosts were imported into CLC MGM and customized for use as separate databases. The HIV nomenclature with nine primary ranks by the ICTV 2022 Release is as follows: Realm: Riboviria; Kingdom: Pararnavirae; Phylum: Artverviricota; Class: Revtraviricetes; Order: Ortervirales; Family: Retroviridae; Subfamily: Orthoretrovirinae; Genus: Lentivirus; and Species: Human immunodeficiency virus 1 [31]. A customized taxonomy based on the attributes of the Baltimore classification and group/sub-lineage nomenclature was created [32,36,37]. We defined our seven-level taxonomic ranks as: Virus_nucleic acid type; Family; Subfamily; Genus; Species; Group; and Sub-lineage. For example, the taxonomy of HIV-1 Group O, sub-lineage null from a blood sample collected in Cameroon (Accession: AY169812) was annotated as “Virus_ssRNA-RT_env; Retroviridae; Orthoretrovirinae; Lentivirus; HIV-1; O;”. The customized taxonomy created in a metadata file replaced the original taxonomy of the sequence file for downstream applications. 

For HBV and HIV-1, the GenBank sequence files imported into CLC MGM were converted into a singular sequence list using the “Create Sequence List” tool. Metadata (.xlsx format) were appended to enrich the sequence list using the “Update Sequence Attributes in Lists” tool. Finally, the singular sequence list was partitioned with the “Split Sequence List” tool based on sequence “Name” to revert to individual sequences in preparation for whole-genome alignment (WGA) and database creation (Figure 2A). 

The human reference genome (Homo sapiens Genome Reference Consortium Human Build 38 (GRCh38 or hg38) files were downloaded using the “Download Genomes from Public Repositories” function within the CLC MGM (Figure 2A) for use within the workflows.

### 2.3. Whole-Genome Alignment and Phylogenetic Analysis of Database Sequences

CLC Genomics Workbench Premium 23.0.4, inclusive of the CLC MGM (Redwood City, CA, USA), was installed on an HP notebook computer (specifications: Windows 10 operating system, Intel i7–7500U dual-core processor @ 2.70 GHz and 8 GB RAM) for all analyses. The CLC system requirements are provided online [27]. The “Whole-Genome Alignment” plugin was downloaded from within the CLC Workbench and the “Create Whole-Genome Alignment” tool was used for automated data analysis (Figure 2A). The analysis consisted of three primary steps: (1) sequence import, (2) alignment parameter selection, and (3) annotation copying from a reference genome (optional) (Figure 2B). The WGA output displayed the aligned regions between all genomes. 

The HBV and HIV-1 genome sequences were aligned by the WGA tool; the annotation of the respective reference genomes (NCBI Nucleotide AN: NC_003977 and NC_001802) were employed to standardize the annotation for all genomes. The WGA output was entered into the “Create Average Nucleotide Identity Comparison” tool to quantify the similarity between genomes (Figure 2A,C). For each pair of genomes, the aligned regions were identified for calculation of two measurements: (1) Alignment Percentage (AP) defined as average percentage of two genomes which is aligned, and (2) Average Nucleotide Identity (ANI) defined as average percentage of matching nucleotides for the aligned regions. The tool generated a pairwise comparison table (type AP or ANI) for input into the ”Create Tree from Comparison” tool for construction of a Neighbor Joining (NJ) or Unweighted Pair Group Method with Arithmetic Mean (UPGMA) tree (Figure 2A). The visual information displayed by the phylogenetic tree was augmented with metadata to decorate or colorize the clades, nodes, and labels. 

### 2.4. Viral Hybrid Capture (VHC) Analysis and Workflow

The “Analyze Viral Hybrid Capture (VHC) Data” ready-to-use workflow of CLC MGM was used for automated data analysis (Figure 2A,D). The analysis consisted of four primary steps: (1) Data import, (2) Data quality control (QC), (3) Taxonomic Profiling of reads mapping to viral and human reference genomes, and (4) Low frequency variant detection. Post-workflow output included tables and visualization tracks for best-matched sequence, read mapping, annotated CDS, low-coverage areas, and annotated genetic variants. 

### 2.5. Viral Integration Site (VIS) Analysis and Workflow

The “Identify Viral Integration Sites (VIS)” ready-to-use workflow of CLC MGM was used for automated data analysis (Figure 2A,E). The analysis consisted of four primary steps: (1) Data import, (2) Reads mapping to human and viral reference genomes, (3) Breakpoint detection in human and viral genomes, and (4) Gene identification surrounding breakpoint(s). Workflow outputs included tables, read mapping to host and viral genomes, and circular plot of viral–host genomes zoomable from the chromosome to gene level. 

## 3. Results

### 3.1. Whole-Genome Alignment (WGA) and Comparison of HBV and HIV-1 Genomes

After the construction of the customized HBV and HIV-1 reference databases as described in Section 2.2, WGA was performed with a respective runtime of 27 and 2 min. The runtimes for the “Create Average Nucleotide Indentity Comparison” tool were only 15 and 2 s, respectively. The output, i.e., pairwise comparison (PWC) table, revealed the quantitative measures of similarity between HBV genomes: (1) the Alignment Percentage (AP) between two HBV genomes (range, 88–100%), and (2) the Average Nucleotide Identity (ANI) or the percentage of exact nucleotide matches of the aligned regions (range, 99–100%). In the context of HIV-1 genomes, the AP ranged from 88% to 100%, while the ANI spanned from 99% to 100%.

#### Phylogenetic Trees of HBV and HIV-1 Genomes

**Figure 3:**
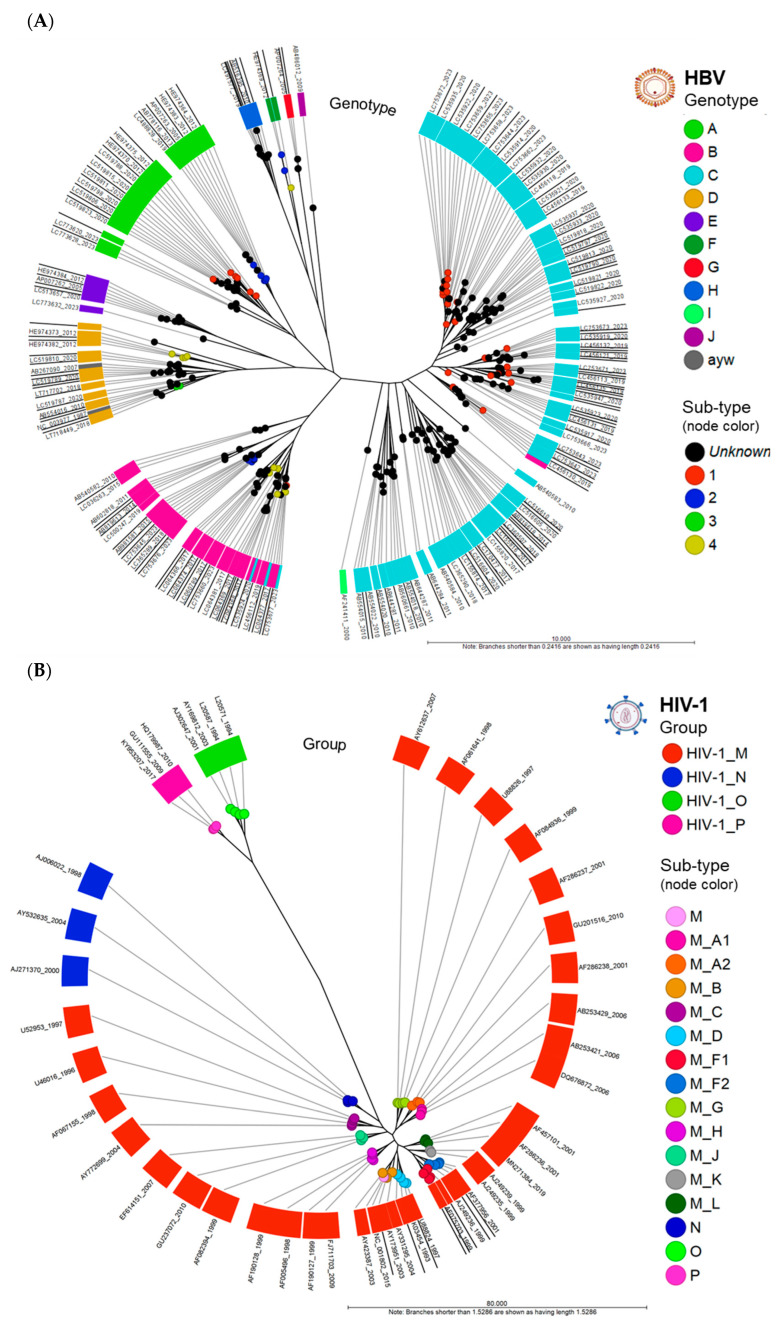
Circular phylograms of HBV and HIV-1 genomes. (**A**) Phylogram of aligned HBV whole genomes (*n* = 268) clustered into 10 genotypes (A–J). The genotypes (clades) and subtypes (nodes) reveal the relatedness of their member samples. Two genomes, NC_003977 (HBV RefSeq) and AB267090, carried the conventional classification by serology (*adw*, *adr*, *ayw*, and *ayr*) based on HBV surface antigen (HBsAg) reactivity. All genomes clustered according to their assigned genotype except for four accessions found in genotypes B and C (pink/blue discordancy in figure). (**B**) Phylogram of aligned HIV-1 whole genomes (*n* = 53) clustered into 4 groups (M–P). All genomes clustered according to their assigned group (clade) and subtype (nodes). The outermost ring (label) displays the NCBI accession number and release date (year).

Circular phylograms of the HBV and HIV-1 genomes were created from the PWC table using the “Create Tree from Comparison” tool (runtime: 1 s). Metadata enriched the visualization of the HBV genotypes and HIV-1 groups, along with their respective sub-lineages. The radial phylogram of the aligned HBV whole genomes (*n* = 268) revealed the clustering of the 10 genotypes (A–J) into clades (Figure 3A). Two genomes, NCBI AN (NC_003977 (HBV RefSeq) and AB267090), carried the conventional (predated) serological classification and nomenclature (*adw*, *adr*, *ayw*, and *ayr*) based on the HBV surface antigen (HBsAg) reactivity. The serotype *ayw* of the two genomes corresponded to genotype D by alignment. All genomes except for four accessions were clustered according to their assigned genotype in NCBI. These discrepant genomes (AN: LC064379, LC535924, LC535945, and LC753677) were found in genotypes B and C (pink/blue in the figure) possibly due to a genotype assignment error. The phylogram of the aligned HIV-1 whole genomes (*n* = 53) were clustered into four groups (M–P) (Figure 3B). All genomes were grouped and clustered correctly based on their assigned clade and subtype, as designated by NCBI. For groups N, O, and P, which lacked subtypes, the nodes were colorized according to their respective clades (as shown in Figure 3B). Additionally, there were a few genomes within group M, also lacking subtypes; the nodes were colored pink in the same figure. The ANI NJ unrooted trees were constructed from the PWC table.

### 3.2. Viral Hybrid Capture (VHC) Analysis and Visualization

The entire dataset comprised 11 FASTQ files, and 10.5 GB of digital information was imported for analysis. The cell-line characteristics are provided in Table 1. The VHC workflow median runtime per sample was 17 min (range, 1 to 61 min). The QC workflow generated the following outputs: (1) QC for sequencing reads (graphical report and supplementary report), and (2) abundance table. Specifically, the graphical report summarized the total number of sequences and nucleotides in a sample, per-sequence analysis, per-base analysis, over-representation analyses, sequence duplication levels, and duplicated sequences. The QC supplementary report includes two additional columns, i.e., “coverage” and “abs” for absolute numbers of sequences or bases for the per-sequence or per-base analyses. The reader is referred to the CLC MGM manual online for an in-depth explanation of QC metrics [27].

#### 3.2.1. HPV Taxonomic Profiling

**Figure 4:**
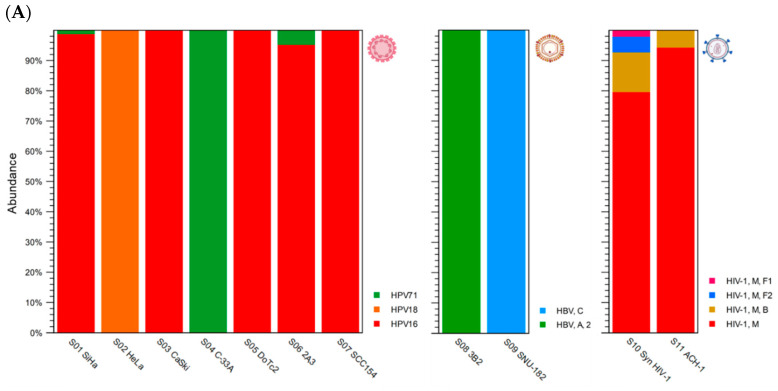

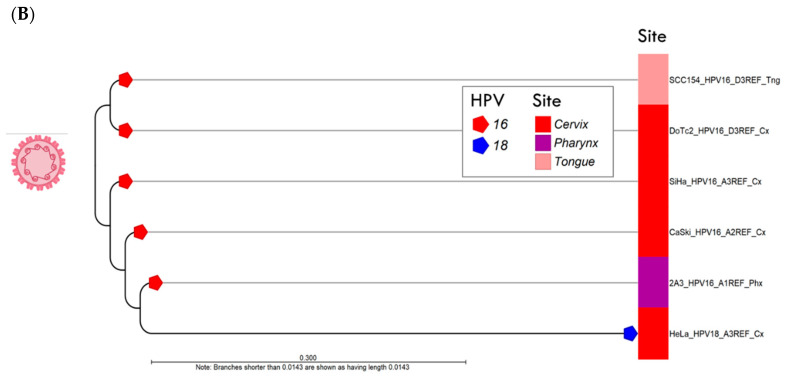
Viral Hybrid Capture (VHC) analysis. (**A**) Relative abundance of HPV, HBV, and HIV-1 genotypes found in individual samples (*n* = 11) after taxonomic profiling are shown as stacked bars. For HPV-positive cell lines (S01–S07), three HPV genotypes were identified in the cohort. For S08 through S11, the HBV and HIV-1 genotypes/groups and sub-lineages were deciphered by taxonomic profiling (see legend). (**B**) For HPV-16 and -18 positive cervical and oral samples, the sub-lineages (alphanumeric code in the label) were determined by BLAST against the HPV variant reference database. The sub-lineages were genetically distinct as shown by the divergent branches in the phylogenetic tree. Cx, cervix; Phx, pharynx; and Tng, tongue.

The HPV taxonomic profiling workflow produced individual abundance tables that displays the names of the identified taxa, seven-level taxonomic nomenclature, coverage estimate, and abundance value (raw or relative number of reads found in the sample associated with the taxon). Low abundance genotypes were cut off at a threshold of <1% of total composition. The merged abundance table (Supplementary Table S1) lists all taxonomic profiling results and the summary statistics, e.g., combined abundance of reads for the taxon across all samples, and the minimum, maximum, mean, median, and standard deviation of the number of reads for the taxa across all samples. The graphical output of the merged abundance table is shown as a stacked bar chart in Figure 4A. For S04 C-33A (p53+ and pRB+), only 346 reads for HPV-71 were identified. According to IARC, HPV-71 is classified as “not classifiable/probably not carcinogenic” [3]. Given the low read counts, this is likely an incidental finding for commensal HPV-71. Instead, the mutations driving this malignant cell line are attributed to p53 and pRB. 

To determine the HPV sub-lineage of the dominant genotype within each sample, the “HPV consensus sequence” generated from the VHC workflow was aligned against the “HPV VAR” BLAST database using the CLC BLAST tool. The BLAST output table is provided in Supplementary Table S2. To construct the phylogenetic tree based on HPV genotype and sub-lineages, the “HPV consensus sequence” output from the VHC workflow of the six samples were aligned collectively and analyzed phylogenetically using the “Create Alignment” and “Create Tree” tools sequentially. The resulting NJ tree was labeled according to the cell-line nomenclature, HPV genotype, sub-lineage, and tumor site as shown in Figure 4B. The identification of HPV-16 and -18 sub-lineages and variants is clinically significant in terms of carcinogenic risk. A global investigation into the dispersal of HPV-16 sub-lineages (A, B, C, and D) revealed that regional specificity (i.e., A3–4 for East Asia, B1–4 and C1–4 for Africa, D2 for the Americas, and B4, C4, and D4 for North Africa) may significantly impact cervical cancer risk [38]. 

#### 3.2.2. Viral Hybrid Capture (VHC) Track Lists

**Figure 5:**
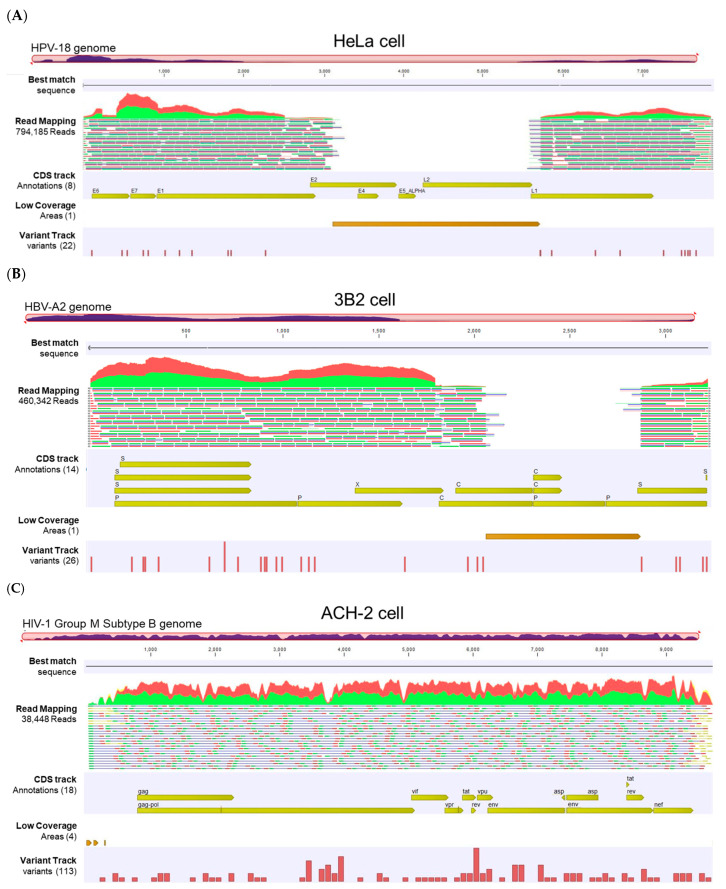
Viral Hybrid Capture (VHC) track list view. (**A**–**C**) Representative VHC track lists for HeLa, 3B2, and ACH-2 cell lines containing HPV, HBV, and HIV-1, respectively. The displayed tracks include (top to bottom): best-match sequence, read mapping against the viral reference genome, coding sequence (CDS) track, low-coverage areas, and annotated variant track. Low-coverage regions correspond to viral genomic gaps (breaks) for the individual viruses. The read mapping track was truncated due to its extensive length.

The VHC workflow generated a “Track List” containing a (1) read mapping track, (2) annotated variant track, (3) amino acid track, and (4) “low-coverage areas” track. Representative track lists from the HeLa, 3B2, and ACH-2 cell lines show paired-reads mapped onto the linearized HPV-18, HBV-A2, and HIV-1 Group M reference genomes, respectively (Figure 5). The auto-generated tracks provide a visual representation of the read abundance, spanning from 38,448 to 794,185 reads, as well as the sequencing coverage and breadth of genome coverage for the three samples. The large gap (break) between reads is automatically detected and shown in the “low-coverage areas” track using the default threshold criteria (best-match reference genome coverage < 30×). The annotated variant track shows low-frequency variants detected using the default threshold (coverage > 30× and frequency ≥20%). Finally, the amino acid track shows the virus-coded amino acids generated from the coding DNA sequence (CDS) annotation of the reference sequence list chosen as the “Best Reference for Read Mapping” in the workflow. Zooming in on the track list to the nucleotide or amino acid level allows for a detailed comparison to the reference genome and the detection of variants. The track lists for all samples (S01 to S11) are displayed in Supplementary Figure S1.

### 3.3. Viral Integration Site (VIS) Analysis and Visualization

**Figure 6:**
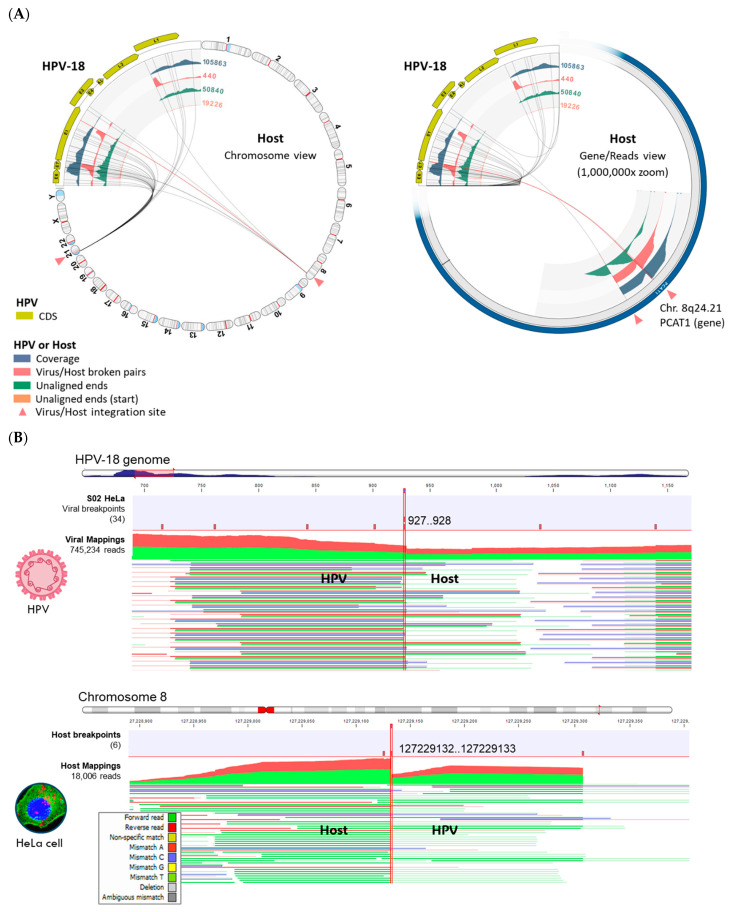
Viral–Host Integration Site (VIS) analysis of HeLa cells. (**A**) VIS circular plots in chromosome view (**left**) and gene view (**right**) revealed two integration sites at chromosomal cytobands 8p24.21 and 21p11.2. The dynamic functions of the VIS circular plot (i.e., genome rotation and zoom) facilitated rapid inspection of the integration sites. (**B**) Read mappings to HPV-18 and chromosome 8 at viral or host breakpoints (vertical brown bars with genomic co-ordinates) reveal both forward (green) and reverse (red) viral–host chimeric reads (bolded HPV/Host). The unaligned chimeric segment of a read sequence stands out with its subdued color. Read mappings were truncated due to their extensive length.

The VIS workflow median runtime per sample was 79 min (range 11 to 158 min). The workflow generated the following output files: (1) the viral mapping and breakpoints annotation track, (2) the host mapping and breakpoints annotation track, (3) the zoomable and rotatable VIS circular plot, and (4) the VIS summary report. A representative VIS circular plot of S02 HeLa presents the entire HPV and human genome in a circular layout with four inner circles of different read tracks (Figure 6A). Virus–host integration linkages i.e., chimeric reads, are shown as bi-directional curvilinear lines, and the read coverage (color-coded histogram tracks) are mapped onto genome co-ordinates (Figure 6A). For S02 HeLa, a large break in the HPV genome between the *E2* and *L2* genes was easily discernable, and viral–host integration within cytobands 8q24.21 and 21p11.2 were detected. The 1,000,000× gene-level view reveals the host integration site(s), disrupted genes (*PCAT1* and *CASC19*), and nearby gene (*MYC*) using the rotational function of the VIS circular plot. In Figure 6B, the HPV and host read mappings for S02 HeLa are depicted. The figure highlights sites of broken read-pairs and viral–host chimeric forward/reverse reads, which have been magnified to show nucleotide-level details. The unaligned segment of chimeric reads is distinguished by its subdued color. 

**Figure 7:**
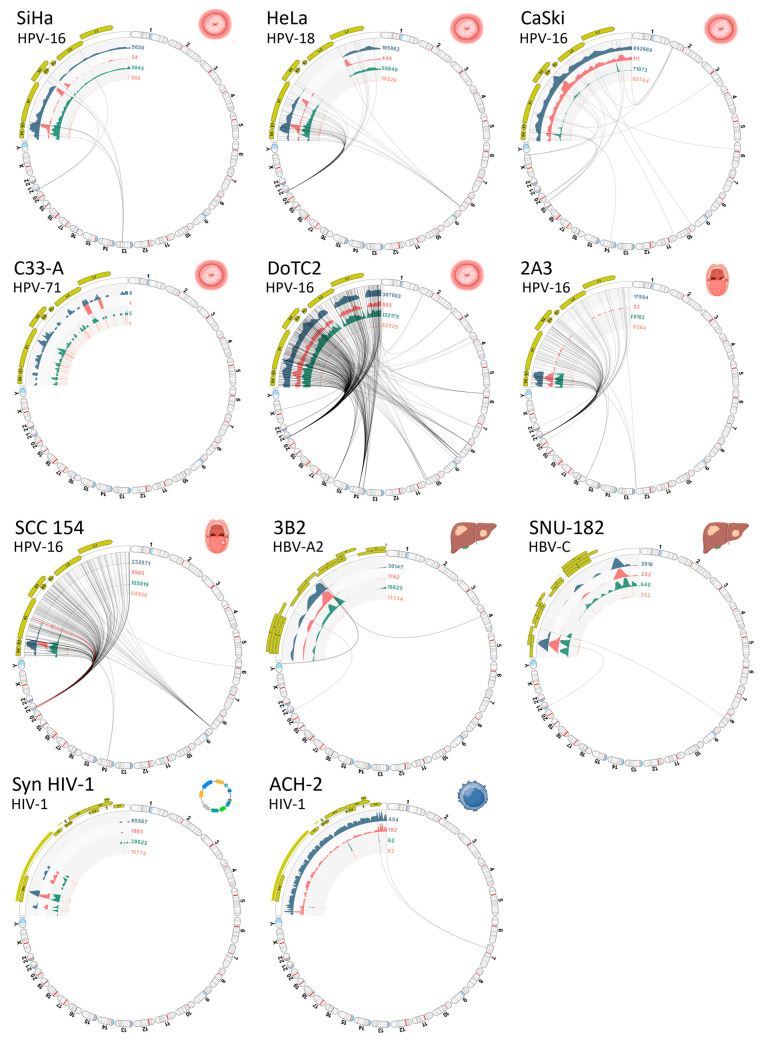
Viral Integration Site (VIS) circular plots. Collage of VIS circular plots for samples (S01 to S11) in chromosome view. Virus–host integration linkages manifested as chimeric reads are designated by the bi-directional curvilinear lines. As expected, the C-33A cervical cancer cell line (p53+ and pRB+) and the synthetic HIV-1 plasmid lacking viral incorporation did not show any viral–host integration. In contrast, the HIV-1-infected ACH-2 cell had a single integration site, while all other virally transformed cell lines exhibited multiple integration sites (anatomical icons created with BioRender.com).

A collage of VIS circular plots in the chromosome view for S01 through S11 is presented in Figure 7. The gaps in viral read mappings are easily identified by the absence of (or low) read coverage represented by the first inner circle (blue-gray histogram). The read counts (numerals) adjoining the inner circular tracks facilitate the quick assessment of read quantity and coverage. Virus–host integration linkages manifested as chimeric reads are represented by the bi-directional curvilinear lines. As expected, the C-33A cervical cancer cell line (expressing p53+ and pRB+) and the synthetic HIV-1 plasmid, which are both devoid of viral incorporation, exhibited no signs of viral–host integration. The HIV-1 infected ACH-2 cells showed a single integration site, where all other virally transformed cell lines displayed multiple integration sites. 

The auto-generated VIS summary reports with tables of disrupted and nearby genes for all samples are provided in Supplementary Table S4. A condensed version lists the major viral integration events detected in all virally transformed cell lines (Table 2). To compare with the existing literature, the genomic co-ordinates in the report were converted to the International System for Human Cytogenetic Nomenclature (ISCN), specifically referring to cytobands [39,40]. Furthermore, an extensive search in PubMed and HumCFS https://webs.iiitd.edu.in/raghava/humcfs/index.html (accessed on 1 January 2024) was performed to determine if a chromosomal region has been deemed a Common Fragile Site (CFS) or viral integration hotspot [41,42]. The disrupted and nearby genes identified in the report were also searched in NCBI Gene and PubMed to determine their function and association with carcinogenesis [43]. Statistically, the median number of viral integration events by chromosomal cytoband for the cohort was 5 (range, 1 to 13). The CFSs identified in this study included chr. 1q25.1, 3p25.1, 4p15.31, 8q24.3, 8q24.13, 8q24.21, 10q26.11, 12q24.33, 13q22.1, 19q13.42, 20q11.23, and Xq27.3 [41]. On average, one integration event per sample was located at a CFS (range, 0 to 3) with a median CFS/VIS of 0.2 (range, 0 to 0.5). Hotspots for HPV integration were identified in SiHa and HeLa cell lines at chr. 13q22.1 and 8q24.21, respectively [42]. Chromosomal regions (chr. 13q22.1, 8q24, and 21p11.2) associated with carcinogenesis were also identified in the cell lines [44,45,46,47,48,49]. The median number of integration events at/near a cytoband or gene associated with carcinogenesis per sample was 2 (range, 2 to 10). Host genes adjacent to the VIS, which have been identified in the literature as oncogenes, tumor suppressor genes, or cancer-associated genes, were denoted in Table 2 by superscripted numerals [44,45,46,47,48,49,50,51,52,53,54,55,56,57,58,59,60,61,62,63,64,65,66,67,68,69,70,71,72,73,74,75,76,77,78]. Taken together, the findings offer valuable insights into the intricate relationship between viral integration, chromosomal location, and genetic alterations that contribute to carcinogenesis.

### 3.4. Workflow Runtimes

**Figure 8:**
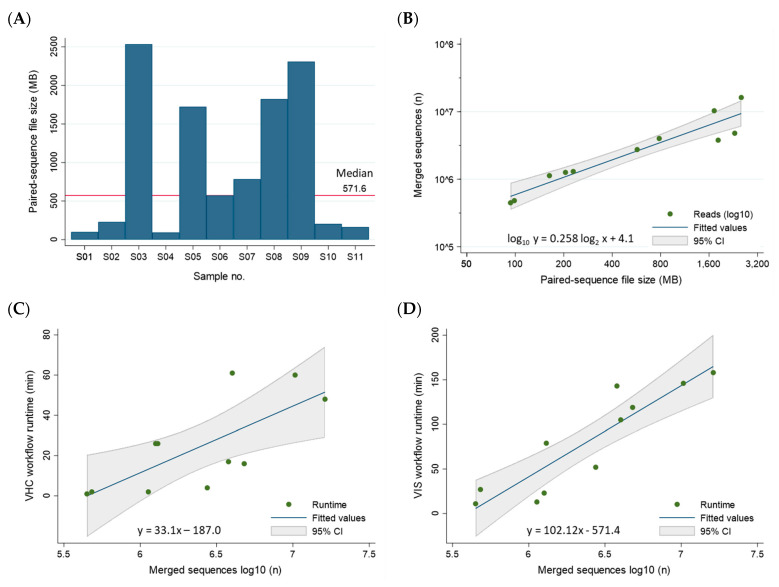
Correlation between NGS reads and VHC/VIS workflow runtimes. (**A**) The sequencing file sizes of the 11 samples ranged broadly between 93 and 2535 MB with a median of 571.6 MB. (**B**) The file size correlated near-perfectly with the number of merged sequences after log_2_–log_10_ transformation, respectively (R^2^ = 0.90). (**C**,**D**) The number of merged reads (log_10_) correlated positively with VHC and VIS workflow runtimes in a linear–log relationship. The correlation was modest for both VHC and VIS with R^2^ = 0.53 and R^2^ = 0.81, respectively. The regression equations are useful for estimation of workflow runtimes based on number of merged reads. Log transformation was used to compress the wide range of X- or Y-values, making them suitable for linear modeling.

The sequencing file size of the 11 samples ranged broadly between 93 and 2535 MB with a median of 571.6 MB consisting of 2,750,036 merged reads (Figure 8A). The file size correlated near-perfectly with the number of merged sequences after log_2_–log_10_ transformation (R^2^ = 0.90) (Figure 8B). The median runtime per sample for the VHC and VIS workflows were 17 min (range, 1 to 61 min) and 79 min (range, 11 to 158 min), respectively. The combined VHC/VIS median runtime per sample was 105 min (range 12 to 206 min). These timed results serve to establish a benchmark for future studies and demonstrate the workflow efficiency for a unified bioinformatics analysis. A modest correlation between the number of merged sequences/sample and VHC and VIS runtimes was found with R^2^ = 0.53 and R^2^ = 0.81, respectively (Figure 8C,D). The regression equations are useful for estimating runtimes based on merged reads/sample (Figure 8C,D). Analyses were performed using STATA/IC 17.0 (StataCorp LP, College Station, TX, USA).

## 4. Discussion

In this study, we developed and tested an end-to-end workflow from NGS to mapping virus–host integration sites. Eleven samples, comprising nine established cancer cell lines, one synthetic HIV-1 plasmid, and one publicly available HIV-1 dataset, were subjected to testing. Overall, the pre-designed hybrid capture probes of the QIAseq xHYB Viral STI Panel performed well in terms of sequence quality and quantity, as well as the breadth and depth of viral genome coverage. 

The creation of curated genomic databases played a vital role in the analytical workflows for both HBV and HIV-1. Unlike HPV, which benefits from an organized and curated genomic database, namely, Papilloma Virus Episteme (PaVE), previously customized for use within CLC MGM [79], both HBV and HIV-1 necessitated considerable manual curation, which were structured similarly to our prototypical HPV database [79]. For HBV, an exhaustive literature search for HBV databases identified only one online database, i.e., HBVdb release 59 https://hbvdb.lyon.inserm.fr/HBVdb/HBVdbIndex (accessed on 25 December 2023), with 106,100 entries and tools for FASTA sequence annotation, genotyping, and drug resistance profiling [80]. Given that HBVdb was not designed for NGS data analysis, we developed our own database sourced from NCBI Virus [29]. The goal was to create a comprehensive and representative database with the following features: (1) sufficient resolution at both the genotype and subtype levels, (2) adequate genomic diversity without being exhaustive computationally, and (3) compatibility with CLC MGM for NGS analysis. For HIV-1, the LANL HIV-1 database served as the foundational resource, providing accession numbers for a comprehensive range of well-characterized HIV-1 reference genomes. However, the GenBank taxonomic information associated with each viral genome needed revision to incorporate genotype and subtype nomenclature. This modification was essential in order to enable precise subtyping for taxonomic profiling and variant analysis. After the construction of the databases and alignment of the genomes, we successfully visualized the phylogenetic distances and relationships. The resulting tree allowed us to examine the genotype and sub-lineage representation, ensuring the accuracy of the databases. With continual viral evolution, it is essential that submissions to the NCBI Virus repository remain current, comprehensive, and globally diverse. A comprehensive range of viral genotypes translated into viral reference databases, such as ours, is indispensable for taxonomic profiling. Without this, data generation bias, where certain groups or variables are underrepresented, may lead to skewed results. Viral sub-lineage classification is of the utmost importance in the realms of epidemiologic research, public health, and outbreak investigations. Recently, the Bacterial and Viral Bioinformatics Resource Center (BV-BRC) held the 2024 Viral Sub-species Classification Workshop to address the complexity, enormity, and challenges of classifying and tracing viral evolution [81]. Establishing a clinically relevant, widely accepted terminology is crucial for clinical virology, especially in the context of diagnostics, vaccine research, and therapeutics [81]. 

The utility of all three databases was demonstrated by using our deep-sequenced samples. By integrating curated viral and human genome databases into CLC MGM workflows, we streamlined the processing of hybrid capture NGS data. This involved inputting FASTQ files, selecting reference genomes, and configuring necessary parameters. The efficient and rapid taxonomic classification and visualization of viral metagenomes allowed us to uncover compositional differences between samples. The VHC mapping, along with its track list and zoomable visualization, facilitated the easy inspection of mapped regions, variants, and low-coverage areas at both the nucleotide and amino acid levels. Remarkably, the median processing time for the VHC workflow was only 17 min per sample using a laptop computer. Furthermore, the HPV consensus sequences obtained from the VHC workflow proved valuable in revealing HPV sub-lineages and elucidating evolutionary relationships between samples. The VIS workflow efficiently processed NGS data, achieving a median runtime of 79 min per sample. The autogenerated VIS outputs featured tracks with viral and host breakpoint annotations, a zoomable and rotatable circular plot, and a summary report highlighting disrupted and surrounding genes. The tabulated report facilitated the review and identification of pathogenic genetic alterations.

The primary locations of viral–host integration identified herein for the SiHa, HeLa, CaSki, SCC154, and ACH-2 cell lines were consistent with prior investigations, although some differences in minor integration sites were noted [24,42,44,45,48,82,83,84,85,86,87,88,89,90,91]. The discrepant results may be attributed to differences in sequencing methods, software platforms, parameters, and cut-off definitions. Additionally, the identification of disrupted and nearby genes depended on user-defined search parameters (e.g., choosing between 100 KB and 500 KB for a nearby gene distance), which can either restrict or expand the results. Studies specifically related to viral integration in the DoTc2, 2A3, Hep 3B2, and SNU-182 cell lines were not found in PubMed or the online databases VISDB and VIS Atlas [88,89,90,91]. Previously, DoTc2 cells were identified as HPV-negative by ATCC [23]. However, a recent study by Vuckovic et al. and subsequent retesting using a novel set of primers by ATCC confirmed the HPV-16 integration [23,92]. Our findings corroborated the HPV-16 integration in DoTc2 and revealed disrupted segments of HPV *L1* and *L2* genes by VHC analysis as the cause of false-negative PCR results. Hence, the findings generated by this study will serve as a valuable reference for future investigations. 

Exploring the functions and interactions of genes adjacent to the viral integration sites provides valuable insights into their roles in carcinogenesis. For instance, in SiHA cells with HPV-16 integration, the gene LINC00393 on chromosome 13q22.1 has been implicated in altering the 3D chromatin structure, leading to the downregulation of the tumor suppressor gene *KLF12* [44]. The HPV-18 DNA fragments in HeLa cells were detected approximately 500 kb upstream of the *MYC* proto-oncogene (located at chr. 8: 127,735,434–127,742,951). *MYC* is the human homolog of the oncogene (*v-myc*) carried by the avian retrovirus, which is associated with myelocytomatosis and other neoplasms [93]. A long-range chromatin interaction between HPV-18 fragments, the *MYC* gene, and the cytoband 8q24.21 has been demonstrated to constitutively activate the *MYC* gene, leading to cell proliferation and tumorigenesis [45]. Notably, the 8q24.21 region is recognized as a hotspot for genetic mutations associated with various cancer types [46,47]. In a recent review focusing on gastric cancer (GC), genetic alterations within the 8q24 cytoband and its sub-bands (8q24.3, 8q24.11-13, 8q24.21, and 8q24.22) were explored [47]. Among the genes frequently associated with GC within the 8q24 region are NSMCE2, PCAT1, CASC19, CASC8, CCAT2, PRNCR1, POU5F1B, PSCA, JRK, MYC, PVT1, and PTK2 [47]. The presence of similar genetic alterations across different cancer types suggests a common mechanism of oncogenesis. In our study, cytoband 21p11.2 emerged as another frequent and significant site. Bi et al. reported that 21p11.2 was the most frequently integrated region in cervical squamous cell carcinoma [48]. Among the affected downstream genes, RNA5-8SN1 to N3 which encode 45S ribosomal RNA promoters could potentially be exploited for expressing viral oncoproteins [48]. Additionally, the downstream gene MIR3648-1 (located at chr. 21: 8,208,473–8,208,652) may play a role as a tumor-suppressive miRNA within the *MIR-3648/FRAT1-FRAT2/MYC* negative feedback loop [50]. In SNU-182 cells, HBV DNA fragments were integrated at *HAS2-AS1* downstream of the *HAS2* gene on cytoband 8q24.13 (located at chr. 8: 118,300,001–121,500,000). A pan-cancer analysis, including HCCA, revealed that the significant downregulation of *HAS2* contributes to cancer progression and metastasis [73,74]. In ACH-2 cells, we identified the integration of the HIV-1 provirus at the *NT5C3A* gene on cytoband 7p14.3 (located at chr. 7: 33,014,113–33,062,776). The NT5C3A-encoded enzyme, pyrimidine 5′ nucleotidase, catalyzes the dephosphorylation of pyrimidine 5′ monophosphates, including the antiretroviral AZT monophosphate (AZT-MP) used in HIV/AIDS treatment [75]. The combined evidence highlights the substantial impact of virally integrated sites and neighboring genes on carcinogenesis or cellular function modification. Elucidating the altered genes and pathways leading to malignant transformation will ultimately identify potential targets for drug development, thereby improving patient outcomes. 

This study has several strengths. Firstly, we demonstrated the benefit of using an off-the-shelf, pre-designed hyb-cap NGS kit for detecting HPV, HBV, and HIV-1. Unlike a custom probe design, which typically demands expert knowledge of the target virus, molecular biology, and bioinformatics, the pre-designed kit circumvented those exacting, time-consuming requirements. Contrarily, custom NGS kits may be tailored to specific research needs and offer flexibility for unique sample types, viral target regions, or sequencing depths. The choice between custom and standard kits will ultimately be guided by the experimental goals and experience of the user. With all target enrichment protocols (standard or custom), NGS amplification bias and uneven coverage must be considered. Factors such as PCR efficiency, sequence complexity, and GC content may lead to the over- or under-representation of viral genomic regions affecting the results. Furthermore, this study represents the initial assessment of the result quality achieved using the QIAseq xHYB Viral STI Panel, providing an important benchmark for future comparisons. Conducting a comparative analysis of hybrid capture NGS kits from various manufacturers will provide valuable information regarding analytical and clinical performance. Secondly, the VHC and VIS workflows, equipped with embedded customized viral databases, efficiently localized viral–host integration sites, and identified disrupted human genes. This end-to-end workflow serves to facilitate translational research and enhance our understanding of viral oncogenesis.

We acknowledge the limitations of our study, which focused on cancer cell lines and a single synthetic plasmid for performance testing. Cell lines may not be representative of the original tumor due to the loss of heterogeneity, as well as genetic mutations and drift over time [94]. As for the HIV-1 plasmids, the absence of cellular genomes with integrated proviruses prevented complete performance testing. To further our investigation, we intend to broaden our testing to include clinical samples and datasets. Hybrid capture NGS technology exhibits remarkable versatility and can be applied to diverse starting materials e.g., genomic RNA or DNA extracted from cells, and fresh or formalin-fixed paraffin-embedded (FFPE) tissues [22]. Additionally, the NGS analysis of cell-free (cfDNA) or circulating tumor DNA (ctDNA) holds great promise for mutational profiling [95]. Sastre-Garau et al. demonstrated that hybrid capture NGS of liquid biopsies (using a standard 10 mL blood sample) from patients with carcinoma of the cervix, oropharynx, oral cavity, anus, and vulva enabled the molecular characterization of HPV DNA and identification of host insertion sites [96]. Similarly, hybrid capture NGS successfully detected cell-free, virus–host chimeric DNA in liquid biopsies obtained from patients with HCCA [97]. NGS hybrid capture probes have also been developed to target all HIV-1 subtypes (groups M, N, O, and P) and HIV-2 subtypes (A and B) for monitoring sequence diversity and tracking viral evolution [98]. In the future, we plan to implement our streamlined approach for detecting viral DNA/RNA fragments in liquid biopsies. This promising, non-invasive test has the potential to assess the therapeutic response and detect residual or recurrent disease in virally induced cancers. 

## 5. Conclusions

In summary, our streamlined workflow—from sequencing to insights—has effectively mapped viral–host integration sites with speed and accuracy. Our approach is well-positioned to expedite genomic exploration and drive progress in the century-old field of tumor virology.

## Figures and Tables

**Table 1:** Cell line and construct information.

Sample No.	Cell Line or Construct ^1^	Age	Genome Ancestry	Virus Genotype	Tumor Site	Histology
S01	SiHa	55	NE Asian (Japanese)	HPV-16	Cervix	SCCA
S02	HeLa	30	African (American)	HPV-18	Cervix	AdenoCA
S03	CaSki	40	N European	HPV-16	Cervix (met) ^5^	SCCA
S04	C-33A	66	N European	P53+, pRB+	Cervix	SCCA
S05	DoTc2	NS	N European	HPV-16	Cervix	NS
S06	2A3	56	SE Asian (Indian)	HPV-*16* ^4^	Hypopharynx	SCCA
S07	SCC154	54	Caucasian	HPV-*16* ^4^	Tongue	SCCA
S08	3B2.1-7	8	African (American)	HBV-*A2* ^4^	Liver	HCCA
S09	SNU-182	24	NE Asian (Korean)	HBV-*C* ^4^	Liver	HCCA
S10	Syn HIV-1 ^2^	NA	NA	HIV-1 *M* ^4^	NA	NA
S11	ACH-2 ^3^	3	Caucasian	HIV-1 *M* ^4^	Blood	T-cell

AdenoCA, adenocarcinoma; HCCA, hepatocellular carcinoma; met, metastasis; NA, not applicable; N, northern; NE, northeastern; NS, not specified; SE, southeastern; and SCCA, squamous cell carcinoma. ^1^ Cell lines (S01–S09) were acquired from ATCC for sequencing [23]. The demographic, virus, and tumor information for samples (S01–S09 and S11) were gleaned from ATCC and Cellosaurus [23,24]. ^2^ Syn HIV-1 is a quantitative synthetic construct of HIV-1 RNA which contains fragments of the 5′ LTR, *gag*, *pot*, *tat*, *rev*, and *nef* genes acquired from ATCC [23]. ^3^ ACH-2 is an HIV-1 latently infected cell line [25]. ^4^ Viral genotypes or groups in italic font were determined by this study. ^5^ Cell line established from a cervical SCCA metastasis on the small bowel mesentery [23].

**Table 2:** Cell lines, viral integration sites, and disrupted host genes.

VIS	Cell Line								
	Cervix				Oropharynx		Liver		T-Cell
	SiHa	HeLa	CaSki	DoTc2	2A3	SCC154	3B2	SNU-182	ACH-2
1	13q22.1 ^1,2,3^ *LINC00393* ^4^ *KLF12* ^5^	8q24.21 ^2,3^ *PCAT1* ^4^ *CASC19* ^4^ *MYC ^5^*	2p11.2 *LINC01830* *PRR30*	2p22.3 *LINC00486* ^4^	12q24.33 ^1^ *FBRSL1*	6p12.2 *7SK* ^4^	4q13.3 ^6^	1q25.1 ^1^ *TNR*	7p14.3 *NT5C3A FKBP9* ^5^ *RP9* ^5^
2	21p11.2 ^2^ *MIR3648-1* ^5^ *RNA5-8SNx*	21p11.2 ^2^ *MIR3648-1* ^5^ *RNA5-8SNx*	3q23 ^6^	3p25.1 ^1^ *ANKRD28* ^4^ *RN7SL4P* ^4^	16p13.3 *METTL26*	8q24.3 ^1,2^ *HGH1* ^4^	13q31.3 ^5^	2q34 *UNC80* ^4^	
3			6p21.1 ^6^	4p15.31 ^1^ *IGFBP7* ^5^	20q11.23 ^1^ *RPN2* ^4^	14q21.3 *RN7SL2 ^4^*	16q11.2 ^6^	4q31.3 *^5^*	
4			10p14 ^6^	6p21.32 *ZBTB22*	21p11.2 ^2^ *MIR3648-1* ^5^ *RNA5-8SNx*	21p11.2 ^2^ *MIR3648-1* ^5^ *RNA5-8SNx*	21p11.2 ^2^ *MIR3648-1* ^5^ *RNA5-8SNx*	8q24.13 ^1,2^ *HAS2-AS1* ^4^	
5			11p15.4 ^6^	7q21.3 *GNGT1* ^4^	22q11.22 *PPIL2* ^4^	21q21.1 ^7^	Yq12 ^6^	17p11.1 ^6^	
6			14q21.3 *MDGA2* ^4^	8p11.21 *PLAT* ^4^ *HGH1* ^4^				21p11.2 ^2^ *MIR3648-1* ^5^ *RNA5-8SNx*	
7			19q13.42 ^1^ *BRSK1* ^4^	9p23 *PTPRD* ^4^ *RN7SL5P* *RMRP* ^4^					
8			20p11.1 ^6^	10q26.11 ^1^ *TUBGCP2* ^4^ *RGS10* ^4^					
9			Xq27.3 ^1,6^	14q21.3 *RPPH1* ^4^ *RN7SL2* ^4^ *RPS29* ^4^ *RN7SL1* ^4^					
10				16p13.3 *METTL26*					
11				21p11.2 ^2^ *MIR3648-1* ^5^ *RNA5-8SNx*					
12				22q11.22 *PPIL2* ^4^					
13				Xq21.2 *DACH2* ^4^					

VIS, viral integration site numbered in sequence; *x*, 1 to 3. ^1^ Chromosomal region and band recognized as a human common fragile site [41]. ^2^ Chromosomal region and band associated with carcinogenesis [44,45,46,47,48,49]. ^3^ Chromosomal region and band recognized as an HPV integration hotspot (i.e., 2q22.3, 3p14.2, 3q28, 8q24.21, 8q24.22, 13q22.1, 14q24.1, 17p11.1, 17q23.1, and 17q23.2) [42]. ^4^ Disrupted oncogenic, tumor suppressor, or cancer-associated gene(s) [44,45,46,47,48,49,50,51,52,53,54,55,56,57,58,59,60,61,62,63,64,65,66,67,68,69,70,71,72,73,74,75,76,77,78]. ^5^ Nearby oncogenic, tumor suppressor, or cancer-associated gene(s) [44,45,46,47,48,49,50,51,52,53,54,55,56,57,58,59,60,61,62,63,64,65,66,67,68,69,70,71,72,73,74,75,76,77,78]. ^6^ Viral integration site without disrupted gene(s). Nearby genes listed in Supplementary Table S4. ^7^ Disrupted uncharacterized long non-coding RNA gene listed in Supplementary Table S4.

## Data Availability

The data presented in this study are openly available in the NCBI Sequence Read Archive (SRA) under BioProject Accession Number: PRJNA1114395, Title: HPV, HBV, and HIV-1 Viral Integration Site Mapping: A Streamlined Workflow from NGS to Genomic Insights of Carcinogenesis. The HBV REF v1.0 and HIV-1 REF v1.0 that support the findings of this study are available as respective Supplementary Materials, Databases S1 and S2.

## Supplementary Materials

The following supporting information can be downloaded at: https://www.mdpi.com/article/10.3390/v16060975/s1, Figure S1: Viral Hybrid Capture (VHC) track lists, Table S1: Taxonomic profiling results, Table S2: BLAST identification of HPV sub-lineages [99], Table S3: Best viral reference by read mapping reports; Table S4: Viral integration sites summary reports; Database S1: HBV REF v1.0 (.clc format); Database S2: HIV-1 REF v1.0 (.clc format).

## Abbreviations

AdenoCA, adenocarcinoma; AN, accession number; ANI, Average Nucleotide Identity; AP, Alignment Percentage; BLAST, Basic local alignment search tool; BV-BRC, Bacterial and Viral Bioinformatics Resource Center; cccDNA, covalently closed circular DNA; CCRS, chemokine receptor type 5; CDS, coding DNA sequence; cfDNA, cell-free DNA; CFS, Common Fragile Site; chr, chromosome; CLC MGM, CLC Microbial Genomics Module; ctDNA, circulating tumor DNA; CXCR4, chemokine receptor type 4; DDR, DNA damage repair; dsDNA, double-stranded DNA; dslDNA, double-stranded linear DNA; ECM, extracellular matrix; gDNA, genomic DNA; GFR, growth factor receptor; HBV, Hepatitis B virus; HCCA, hepatocellular carcinoma; HIV, Human immunodeficiency virus type 1; HPV, Human papillomavirus; HSPG, heparin sulfate proteoglycan; hyb-cap NGS, hybrid capture next-generation sequencing; IARC, International Agency for Research on Cancer; ICTV, International Committee on Taxonomy of Viruses; ISCN, International System for Human Cytogenetic Nomenclature; LANL, Los Alamos National Laboratory; MMEJ, micro-homology mediated end-joining; NGS, next-generation sequencing; NHEJ, non-homologous end-joining; NJ, Neighbor Joining; NTCP, Na+-taurocholate co-transporting polypeptide; PWC, pairwise comparison; rcDNA, relaxed circular DNA; RSV, Rous sarcoma virus; SCCA, squamous cell carcinoma; SRA, Sequence Read Archive; VHC, Viral Hybrid Capture; VIS, Viral Integration Site; WGA, whole-genome alignment.
