# Supplementary material for: HPV, HBV, and HIV-1 Viral Integration Site Mapping: A Streamlined Workflow from NGS to Genomic Insights of Carcinogenesis
Source: Viruses. 2024 Jun 18;16(6):975. doi: 10.3390/v16060975 (PMC11209625; doi:10.3390/v16060975)
Supplement: Supplementary file 1 [file viruses-16-00975-s001.zip › FIG S1 VHC TRACKLISTS.pdf]

A

S01 SiHa (cervix)

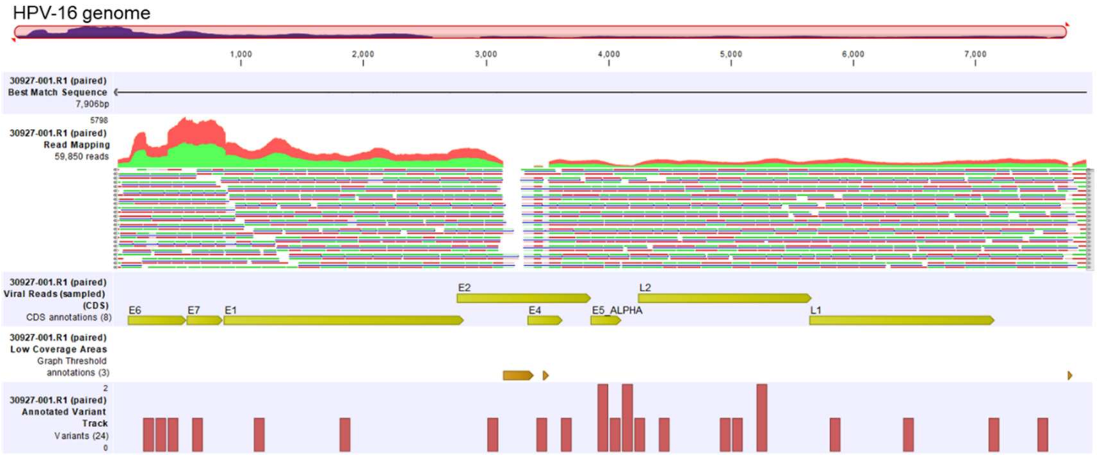

B

S02 HeLa (cervix)

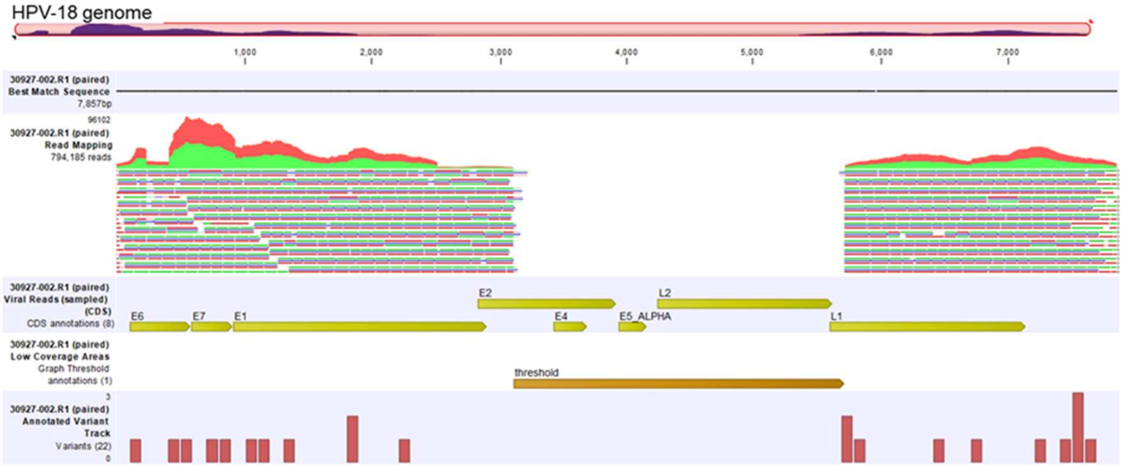

C

S03 CaSki (cervix)

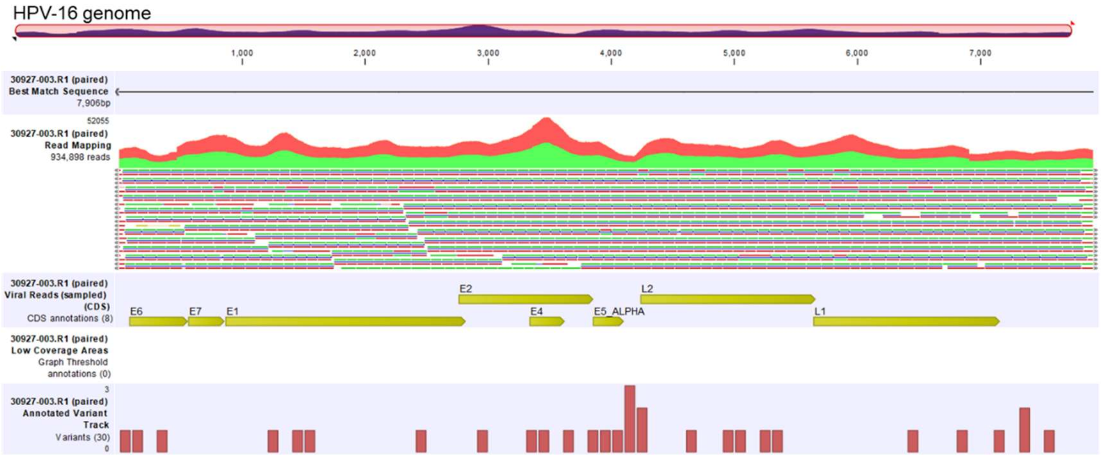

D

S04 C-33A (cervix)

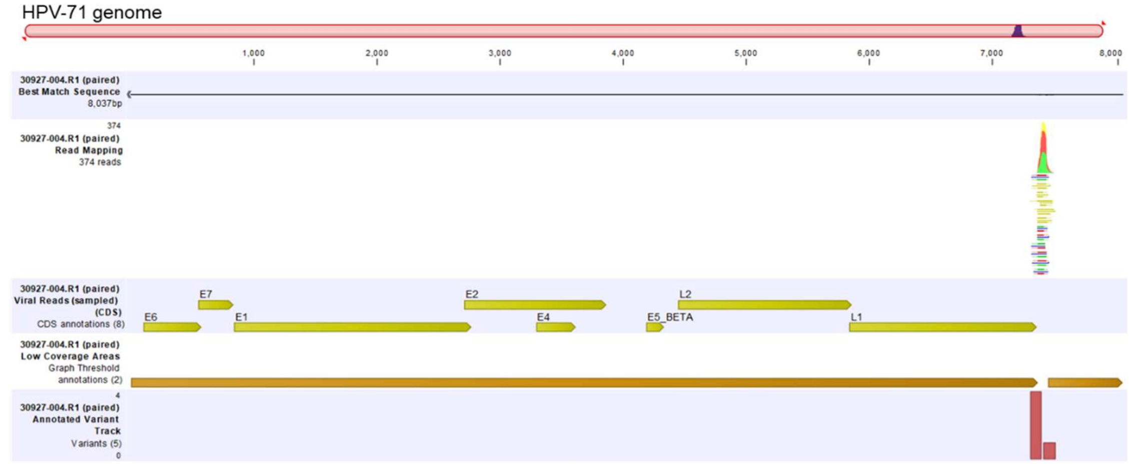

E

S05 DoTc2 (cervix)

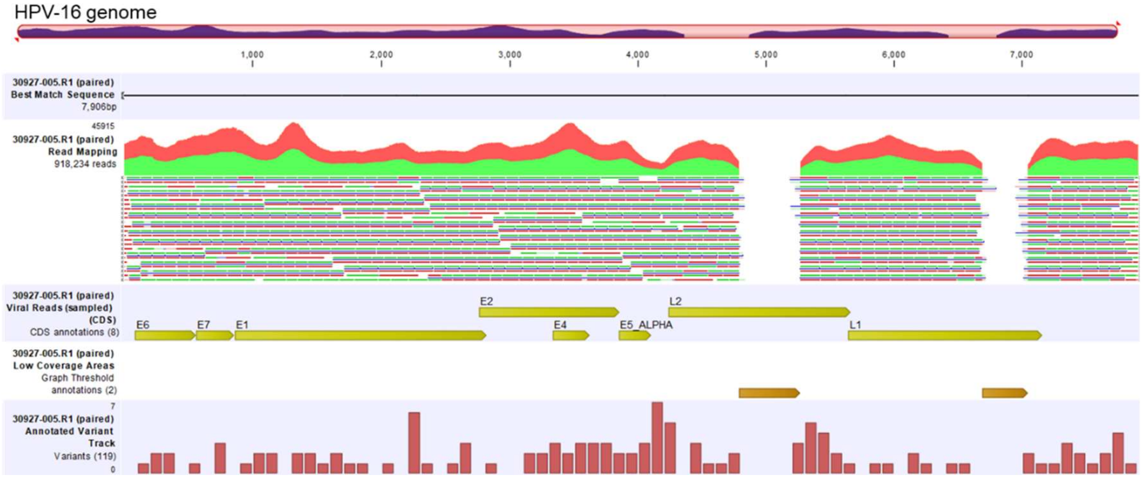

F

S06 2A3 (hypopharynx)

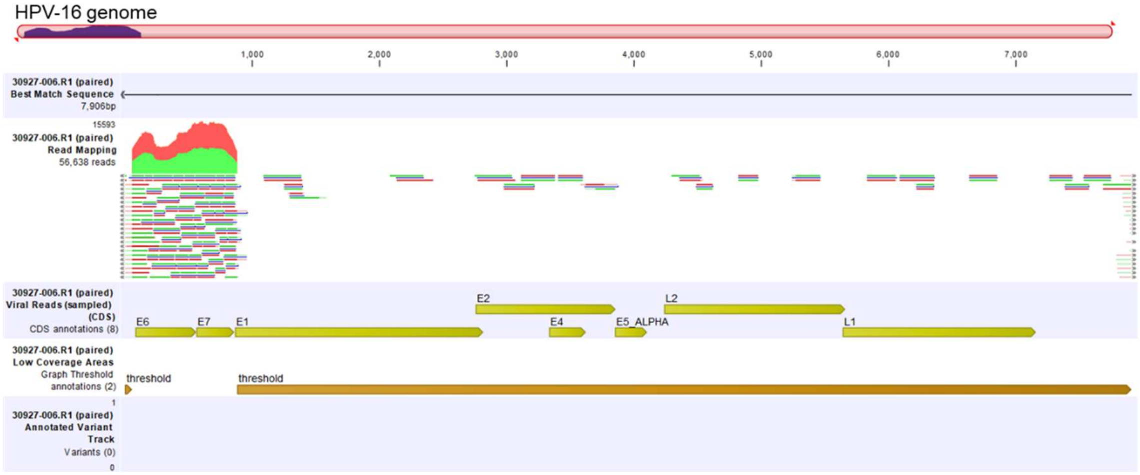

G

S07 SCC154 (tongue)

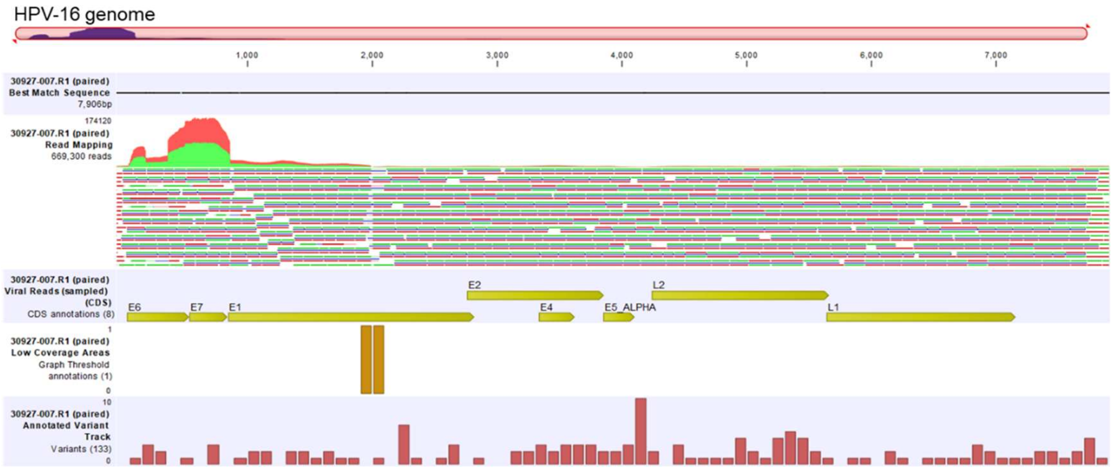

H

S08 3B2.1-7 (liver)

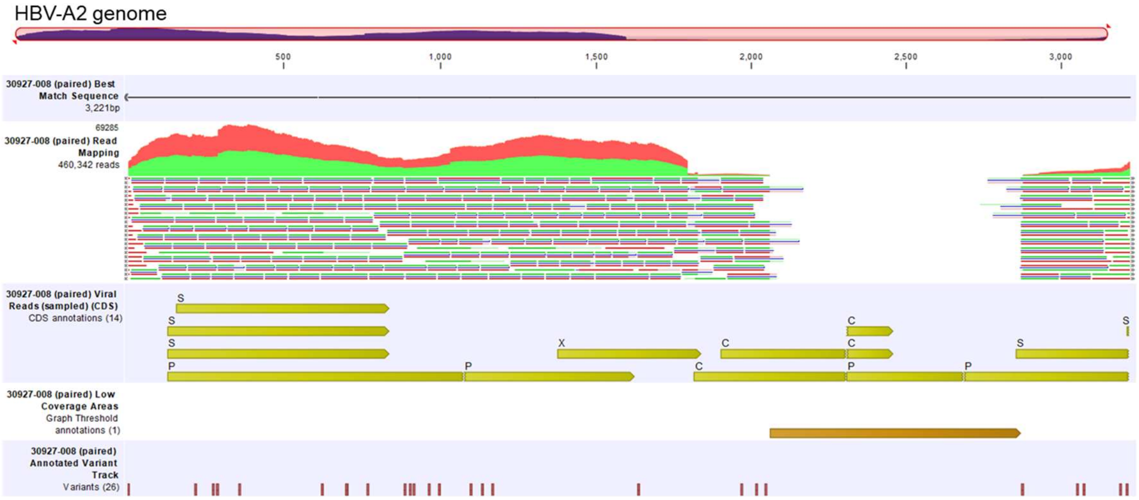

I

S09 SNU-182 (liver)

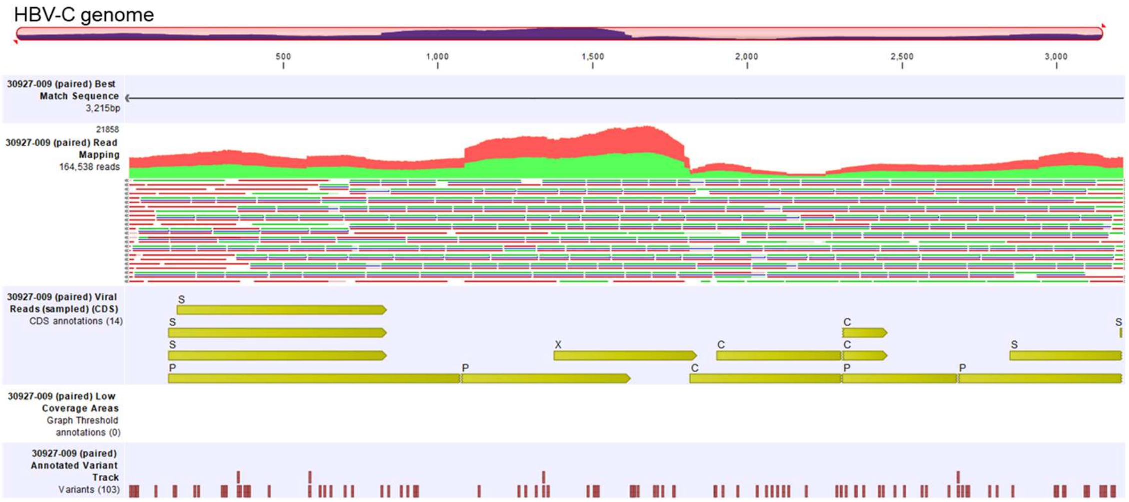

J

S10 Syn HIV-1

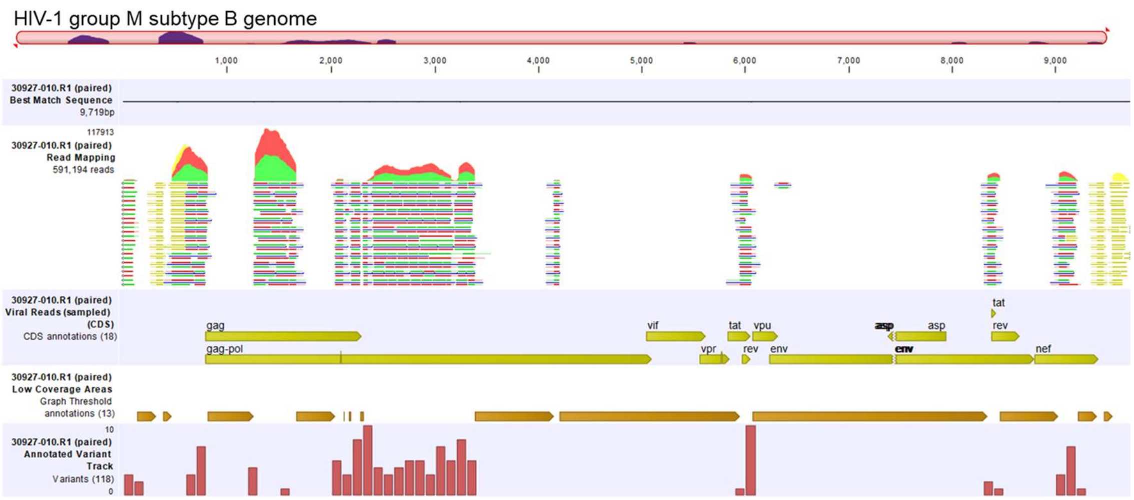

K

S11 ACH-2 (blood)

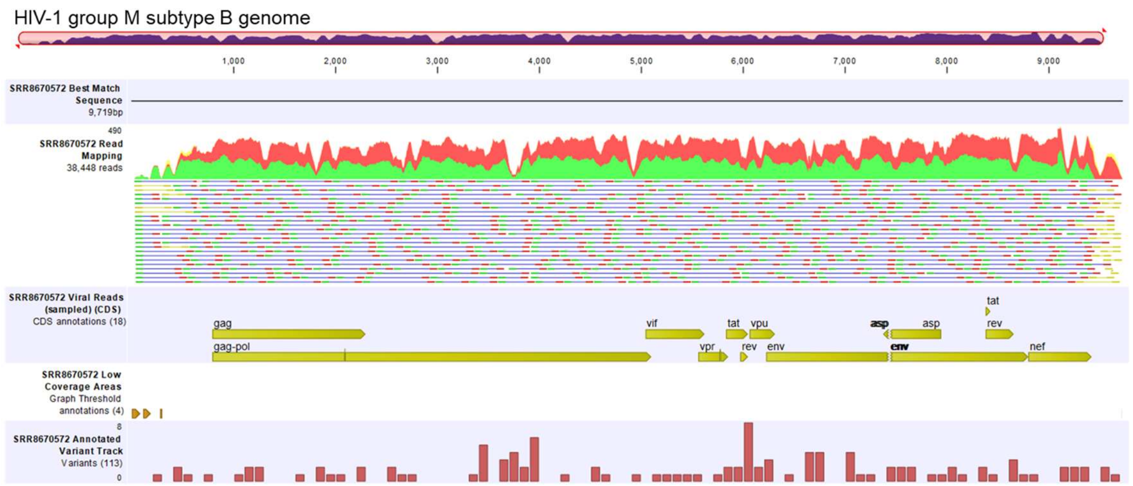

L

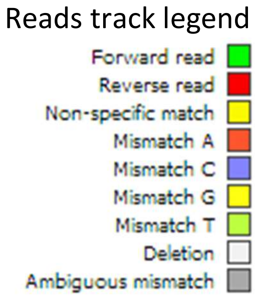

Figure S1. Viral Hybrid Capture (VHC) track list of the best match HPV, HBV and HIV-1 genotype identified by read mapping **(A-K**) for samples S01 to S11. The reads track legend is shown in panel **L**.
