# Supplementary material for: HPV, HBV, and HIV-1 Viral Integration Site Mapping: A Streamlined Workflow from NGS to Genomic Insights of Carcinogenesis
Source: Viruses. 2024 Jun 18;16(6):975. doi: 10.3390/v16060975 (PMC11209625; doi:10.3390/v16060975)
Supplement: Supplementary file 1 [file viruses-16-00975-s001.zip › TABLE S1 TAXON PROFILING.pdf]

Table S1 Taxonomic profiling results

Name	Taxonomy[1]	Combined Abundance	Min	Max	Mean	Median	Std
HPV16	Virus_dsDNA; Papillomaviridae; Alpha; Alpha 9; HPV16	24280622	0	15524456	3,468,660.29	63928	6,026,538.31
HPV18	Virus_dsDNA; Papillomaviridae; Alpha; Alpha 7; HPV18	864392	0	864392	123,484.57	0	326,709.47
HPV71	Virus_dsDNA; Papillomaviridae; Alpha; Alpha 14; HPV71	4228	0	3162	604	0	1,160.65

Name	Taxonomy	Combined Abundance	Min	Max	Mean	Median	Std
HBV A	Virus_dsDNA-RT_env; Hepadnaviridae; Orthohepadnavirus; HBV; A	176056	0	176056	88,028.00	88028	124,490.39
HBV C	Virus_dsDNA-RT_env; Hepadnaviridae; Orthohepadnavirus; HBV; C	151738	0	151738	75,869.00	75869	107,294.97

Name	Taxonomy	Combined Abundance	Min	Max	Mean	Median	Std
HIV-1 M	Virus_ssRNA-RT_env; Retroviridae; Orthoretrovirinae; Lentivirus; HIV-1; M	789380	36298	753082	394,690.00	394690	506,842.83
HIV-1 M, B	Virus_ssRNA-RT_env; Retroviridae; Orthoretrovirinae; Lentivirus; HIV-1; M; B	126392	2156	124236	63,196.00	63196	86,323.60
HIV-1 M, F1	Virus_ssRNA-RT_env; Retroviridae; Orthoretrovirinae; Lentivirus; HIV-1; M; F1	19032	0	19032	9,516.00	9516	13,457.66
HIV-1 M, F2	Virus_ssRNA-RT_env; Retroviridae; Orthoretrovirinae; Lentivirus; HIV-1; M; F2	48856	0	48856	24,428.00	24428	34,546.41

Name, name of reference genome.
[1]An in-depth explanation of Taxonomic Profiling metrics (column headers) is provided in the CLC Microbial Genomics Module manual online [27].
[2]Abundance of reads mapping to a specific reference genome are shown as counts (n).

Table S1 Taxonomic profiling results

Name	30927-001 (paired, trimmed pairs) Abundance[2]	30927-002 (paired, trimmed pairs) Abundance	30927-003 (paired, trimmed pairs) Abundance	30927-004 (paired, trimmed pairs) Abundance	30927-005 (paired, trimmed pairs) Abundance	30927-006 (paired, trimmed pairs) Abundance	30927-007 (paired, trimmed pairs) Abundance
HPV16	61218	0	15524456	0	7770390	63928	860630
HPV18	0	864392	0	0	0	0	0
HPV71	720	0	0	346	0	3162	0

Name	30927-008 (paired, trimmed pairs) Abundance[2]	30927-009 (paired, trimmed pairs) Abundance
HBV A	176056	0
HBV C	0	151738

Name	30927-010 (paired, trimmed pairs) Abundance[2]	SRR8670572 (trimmed pairs) Abundance
HIV-1 M	753082	36298
HIV-1 M, B	124236	2156
HIV-1 M, F1	19032	0
HIV-1 M, F2	48856	0
