# Supplementary material for: HPV, HBV, and HIV-1 Viral Integration Site Mapping: A Streamlined Workflow from NGS to Genomic Insights of Carcinogenesis
Source: Viruses. 2024 Jun 18;16(6):975. doi: 10.3390/v16060975 (PMC11209625; doi:10.3390/v16060975)
Supplement: Supplementary file 1 [file viruses-16-00975-s001.zip › TABLE S2 BLAST HPV SUBLINEAGES.pdf]

Table S2 BLAST identification of HPV sublineages

Query[1]	Dominant, Integrated genotype	No. of HSPs	Lowest E-value	Accession (E-value)	Description (E-value)
30927-001.R1 Read Mapping HPV16REF consensus	Int, D=I	1157	0	HPV16,_A3REF_EU_HQ644236	HPV 16 isolate AS411, complete genome.
30927-002.R1 Read Mapping HPV18REF consensus	Int, D=I	911	0	HPV18,_A3REF_EF202147	HPV 18 isolate Qv15586, complete genome.
30927-003.R1 Read Mapping HPV16REF consensus	Int, D=I	1101	0	HPV16,_A2REF_EU_AF536179	HPV 16 isolate Euro German 131, complete genome.
30927-004.R1 Read Mapping HPV71REF consensus	Dom	0		not available	not available
30927-005.R1 Read Mapping HPV16REF consensus	Int, D=I	1035	0	HPV16,_D3REF,_AsA1_AF402678	HPV 16 Asian-American variant, complete genome.
30927-006.R1 Read Mapping HPV16REF consensus	Int, D=I	162	0	HPV16,_A1REF_EU_PPH16	HPV 16 (HPV16), complete genome.
30927-007.R1 Read Mapping HPV16REF consensus	Int, D=I	1072	0	HPV16,_D3REF,_AsA1_AF402678	HPV 16 Asian-American variant, complete genome.

Dom, dominant HPV genotype; D = I, dominant and integrated HPV types are identical; D /= I, dominant and integrated HPV types are nonidentical; Int, integrated HPV genotype; REF, reference

[1]Query sequences and BLAST results. BLAST statistics nomenclature and definitions from left to right of table columns [99]: HSP (n), Number of high scoring pairs from the source database; Lowest E- val, Lowest Expect value; Accession (E-val), Accession name of the matched database sequence with the lowest E-value; HPV Type, HPV genotype (numeral), variant and sub-lineage (alphanumeric) of the Accession sequence; Hit, Name of the sequence found in the BLAST search with the max score; Total score, Total alignment score for all HSPs; Max score, Maximum (best) score or highest alignment score of all HSPs; Min E-value, Minimum (best) e-value of all HSPs; Max bit score, Maximum (best) bit score of all HSPs; Max id, Maximum number of identical residues in the query and Hit sequence; Max %id, Percentage of maximum identical residues in the query and Hit sequence; Max pos, Maximum number of similar but not necessarily identical residues in the query and Hit sequence; Max %pos, Percentage of maximum similar but not necessarily identical residues in the query and Hit sequence.

Table S2 BLAST identification of HPV sublineages

Query	Greatest identity %	Accession (identity %)	Description (identity %)
30927-001.R1 Read Mapping HPV16REF consensus	100	HPV16,_A3REF_EU_HQ644236	HPV 16 isolate AS411, complete genome.
30927-002.R1 Read Mapping HPV18REF consensus	100	HPV85,_A1REF_AF131950	HPV candHPV85, complete genome.
30927-003.R1 Read Mapping HPV16REF consensus	100	HPV16,_A2REF_EU_AF536179	HPV 16 isolate Euro German 131, complete genome.
30927-004.R1 Read Mapping HPV71REF consensus		not available	not available
30927-005.R1 Read Mapping HPV16REF consensus	100	HPV16,_D3REF,_AsA1_AF402678	HPV 16 Asian-American variant, complete genome.
30927-006.R1 Read Mapping HPV16REF consensus	100	HPV16,_A1REF_EU_PPH16	HPV 16 (HPV16), complete genome.
30927-007.R1 Read Mapping HPV16REF consensus	100	HPV16,_D3REF,_AsA1_AF402678	HPV 16 Asian-American variant, complete genome.

Table S2 BLAST identification of HPV sublineages

Query	Greatest positive %	Accession (positive %)	Description (positive %)
30927-001.R1 Read Mapping HPV16REF consensus	100	HPV16,_A3REF_EU_HQ644236	HPV 16 isolate AS411, complete genome.
30927-002.R1 Read Mapping HPV18REF consensus	100	HPV85,_A1REF_AF131950	HPV candHPV85, complete genome.
30927-003.R1 Read Mapping HPV16REF consensus	100	HPV16,_A2REF_EU_AF536179	HPV 16 isolate Euro German 131, complete genome.
30927-004.R1 Read Mapping HPV71REF consensus		not available	not available
30927-005.R1 Read Mapping HPV16REF consensus	100	HPV16,_D3REF,_AsA1_AF402678	HPV 16 Asian-American variant, complete genome.
30927-006.R1 Read Mapping HPV16REF consensus	100	HPV16,_A1REF_EU_PPH16	HPV 16 (HPV16), complete genome.
30927-007.R1 Read Mapping HPV16REF consensus	100	HPV16,_D3REF,_AsA1_AF402678	HPV 16 Asian-American variant, complete genome.

Table S2 BLAST identification of HPV sublineages

Query	Greatest HSP length	Accession (HSP length)	Description (HSP length)
30927-001.R1 Read Mapping HPV16REF consensus	4525	HPV16,_B2REF_AF1_HQ644298	HPV 16 isolate Z109, complete genome.
30927-002.R1 Read Mapping HPV18REF consensus	3100	HPV18,_A3REF_EF202147	HPV 18 isolate Qv15586, complete genome.
30927-003.R1 Read Mapping HPV16REF consensus	7909	HPV16,_B2REF_AF1_HQ644298	HPV 16 isolate Z109, complete genome.
30927-004.R1 Read Mapping HPV71REF consensus		not available	not available
30927-005.R1 Read Mapping HPV16REF consensus	4790	HPV16,_B2REF_AF1_HQ644298	HPV 16 isolate Z109, complete genome.
30927-006.R1 Read Mapping HPV16REF consensus	838	HPV58,_D2REF_HQ537770	HPV 58 isolate Rw697, complete genome.
30927-007.R1 Read Mapping HPV16REF consensus	7909	HPV16,_B2REF_AF1_HQ644298	HPV 16 isolate Z109, complete genome.

Table S2 BLAST identification of HPV sublineages

Query	Greatest bit score	Accession (bit score)	Description (bit score)
30927-001.R1 Read Mapping HPV16REF consensus	7790.04	HPV16,_A3REF_EU_HQ644236	HPV 16 isolate AS411, complete genome.
30927-002.R1 Read Mapping HPV18REF consensus	5551.15	HPV18,_A3REF_EF202147	HPV 18 isolate Qv15586, complete genome.
30927-003.R1 Read Mapping HPV16REF consensus	14149.6	HPV16,_A2REF_EU_AF536179	HPV 16 isolate Euro German 131, complete genome.
30927-004.R1 Read Mapping HPV71REF consensus		not available	not available
30927-005.R1 Read Mapping HPV16REF consensus	8521.3	HPV16,_D3REF,_AsA1_AF402678	HPV 16 Asian-American variant, complete genome.
30927-006.R1 Read Mapping HPV16REF consensus	1489.07	HPV16,_A1REF_EU_PPH16	HPV 16 (HPV16), complete genome.
30927-007.R1 Read Mapping HPV16REF consensus	14153.2	HPV16,_D3REF,_AsA1_AF402678	HPV 16 Asian-American variant, complete genome.
