# Supplementary material for: HPV, HBV, and HIV-1 Viral Integration Site Mapping: A Streamlined Workflow from NGS to Genomic Insights of Carcinogenesis
Source: Viruses. 2024 Jun 18;16(6):975. doi: 10.3390/v16060975 (PMC11209625; doi:10.3390/v16060975)
Supplement: Supplementary file 1 [file viruses-16-00975-s001.zip › TABLE S3 BEST MATCH VIRUS REF GENOME.pdf]

S01 SiHa

1. Find Best References using Read Mapping summary

Input reads	62,410
Reads mapped to references	61,582
Reads mapped to host	N/A
Reads mapped to references (%)	98.67
Reads mapped to host (%)	N/A

2. References

Reference	Reads mapped	Unambiguously mapped reads	Fraction of reference covered	Coverage
HPV16REF	61,582	61,576	0.98	1,428.02

Reference	Taxonomy	Description	Latin name
HPV16REF	Virus_dsDNA; Papillomaviridae; Alpha; Alpha 9; HPV16; ;	Human papillomavirus 16 (HPV16), complete genome	HPV16REF

S02 HeLa

1. Find Best References using Read Mapping summary

Input reads	869,408
Reads mapped to references	868,858
Reads mapped to host	N/A
Reads mapped to references (%)	99.94
Reads mapped to host (%)	N/A

2. References

Reference	Reads mapped	Unambiguously mapped reads	Fraction of reference covered	Coverage
HPV18REF	868,858	868,566	0.67	20,425.66

Reference	Taxonomy	Description	Latin name
HPV18REF	Virus_dsDNA; Papillomaviridae; Alpha; Alpha 7; HPV18; ;	Human papillomavirus 18 (HPV18), complete genome	HPV18REF

S03 CaSki

1. Find Best References using Read Mapping summary

Input reads	1,000,000
Reads mapped to references	999,970
Reads mapped to host	N/A
Reads mapped to references (%)	100.00
Reads mapped to host (%)	N/A

2. References

Reference	Reads mapped	Unambiguously mapped reads	Fraction of reference covered	Coverage
HPV16REF	999,970	999,912	1.00	25,836.87

S04 C-33A

1. Find Best References using Read Mapping summary

Input reads	386
Reads mapped to references	348
Reads mapped to host	N/A
Reads mapped to references (%)	90.16
Reads mapped to host (%)	N/A

2. References

Reference	Reads mapped	Unambiguously mapped reads	Fraction of reference covered	Coverage
HPV71REF	348	278	0.01	2.13

Reference	Taxonomy	Description	Latin name
HPV71REF	Virus_dsDNA; Papillomaviridae; Alpha; Alpha 14; HPV71; ;	Human papillomavirus 71 (HPV71), complete genome	HPV71REF

S05 DoTc2

1. Find Best References using Read Mapping summary

Input reads	1,000,000
Reads mapped to references	999,594
Reads mapped to host	N/A
Reads mapped to references (%)	99.96
Reads mapped to host (%)	N/A

2. References

Reference	Reads mapped	Unambiguously mapped reads	Fraction of reference covered	Coverage
HPV16REF	999,594	999,508	0.90	25,986.27

S06 2A3

1. Find Best References using Read Mapping summary

Input reads	67,760
Reads mapped to references	64,240
Reads mapped to host	N/A
Reads mapped to references (%)	94.81
Reads mapped to host (%)	N/A

2. References

Reference	Reads mapped	Unambiguously mapped reads	Fraction of reference covered	Coverage
HPV16REF	64,240	64,224	0.60	1,381.48

Reference	Taxonomy	Description	Latin name
HPV16REF	Virus_dsDNA; Papillomaviridae; Alpha; Alpha 9; HPV16; ;	Human papillomavirus 16 (HPV16), complete genome	HPV16REF

S07 SCC154

1. Find Best References using Read Mapping summary

Input reads	870,942
Reads mapped to references	867,696
Reads mapped to host	N/A
Reads mapped to references (%)	99.63
Reads mapped to host (%)	N/A

2. References

Reference	Reads mapped	Unambiguously mapped reads	Fraction of reference covered	Coverage
HPV16REF	867,696	867,078	1.00	19,414.47

Reference	Taxonomy	Description	Latin name
HPV16REF	Virus_dsDNA; Papillomaviridae; Alpha; Alpha 9; HPV16; ;	Human papillomavirus 16 (HPV16), complete genome	HPV16REF

S08 3B2

1. Find Best References using Read Mapping summary

Input reads	591,262
Reads mapped to references	96,872
Reads mapped to host	N/A
Reads mapped to references (%)	16.38
Reads mapped to host (%)	N/A

2. References

Reference	Reads mapped	Unambiguously mapped reads	Fraction of reference covered	Coverage
HE974383	96,872	38,044	0.74	5,327.65

Reference	Host	Taxonomy	Description	Latin name
HE974383	Homo sapiens	Virus_dsDNA-RT_env; Hepadnaviridae; Orthohepadnavirus; HBV; A; 2; MTQ	HBV; A; 2_Mart-B74	HBV A2_HE974383

S09 SNU-182

1. Find Best References using Read Mapping summary

Input reads	203,530
Reads mapped to references	20,718
Reads mapped to host	N/A
Reads mapped to references (%)	10.18
Reads mapped to host (%)	N/A

2. References

Reference	Reads mapped	Unambiguously mapped reads	Fraction of reference covered	Coverage
AB981580	20,718	11,807	0.84	1,429.14

Reference	Host	Taxonomy	Description	Latin name
AB981580	Homo sapiens	Virus_dsDNA-RT_env; Hepadnaviridae; Orthohepadnavirus; HBV; C; ; JPN	HBV; C; _P1-090725	HBV C_AB981580

S10 Syn HIV-1

1. Find Best References using Read Mapping summary

Input reads	945,208
Reads mapped to references	348,812
Reads mapped to host	N/A
Reads mapped to references (%)	36.90
Reads mapped to host (%)	N/A

2. References

Reference	Reads mapped	Unambiguously mapped reads	Fraction of reference covered	Coverage
K03455	348,812	4,039	0.30	5,431.81

Reference	Host	Taxonomy	Description	Latin name
K03455	Homo sapiens	Virus_ssRNA-RT_env; Retroviridae; Orthoretrovirinae; Lentivirus; HIV-1; M; B	HIV-1; M; B	HIV-1_M_K03455

S11 ACH-1

1. Find Best References using Read Mapping summary

Input reads	38,454
Reads mapped to references	19,212
Reads mapped to host	N/A
Reads mapped to references (%)	49.96
Reads mapped to host (%)	N/A

2. References

Reference	Reads mapped	Unambiguously mapped reads	Fraction of reference covered	Coverage
K03455	19,212	1,228	1.00	149.43

Reference	Host	Taxonomy	Description	Latin name
K03455	Homo sapiens	Virus_ssRNA-RT_env; Retroviridae; Orthoretrovirinae; Lentivirus; HIV-1; M; B	HIV-1; M; B	HIV-1_M_K03455
