# Supplementary material for: HPV, HBV, and HIV-1 Viral Integration Site Mapping: A Streamlined Workflow from NGS to Genomic Insights of Carcinogenesis
Source: Viruses. 2024 Jun 18;16(6):975. doi: 10.3390/v16060975 (PMC11209625; doi:10.3390/v16060975)
Supplement: Supplementary file 1 [file viruses-16-00975-s001.zip › TABLE S4 VIS REPORT.pdf]

30927-001.R1 (paired) (Report)

1. Identify Viral Integration Sites summary

Input reads	481,770
Host reads	315,088
Virus reads	54,486
Unmapped reads	112,196
Host reads (%)	65.40
Virus reads (%)	11.31
Unmapped reads (%)	23.29
Breakpoints identified	8
Viruses identified	1

2. Identify Viral Integration Sites virus content

Virus	Reads mapped
HPV16REF	27,536

3. Identify Viral Integration Sites breakpoint summary

Host chromosome	Host region	Virus	Virus region	Unaligned ends host
13	complement (73214728..73214729)	HPV16REF	3388..3389	163
13	73513425..73513426	HPV16REF	3133..3134	145
13	73413638..73413639	HPV16REF	880..881	30
13	73456964..73456965	HPV16REF	232..233	48
13	73456965..73456966	HPV16REF	874..875	85
21	complement (8443204..8443205)	HPV16REF	3133..3134	36
21	8211277..8211278	HPV16REF	5335..5336	34
21	8218813..8218814	HPV16REF	880..881	54

Host chromosome	Unaligned ends virus	Broken reads host	Disrupted genes	Nearby genes
13	N/A	15		RNU4-10P (-23061), RNY1P8 (+12418)
13	369	25	LINC00393	LINC00393, LINC00392 (+50818)

30927-001.R1 (paired) (Report)

Host chromosome	Unaligned ends virus	Broken reads host	Disrupted genes	Nearby genes
13	515	4	LINC00393	gene: ENSG00000279754 (-14433), LINC00393
13	72	13	LINC00393	gene: ENSG00000279754 (-57759), LINC00393
13	40	13	LINC00393	gene: ENSG00000279754 (-57760), LINC00393
21	369	2	gene: ENSG00000280441	gene: ENSG00000280441, MIR6724-3 (-54751), gene: ENSG00000274868 (-54217), gene: ENSG00000280614 (-48863), RNA5-8SN3 (-47445), gene: ENSG00000286267 (-24969), gene: ENSG00000286146 (-22201), gene: ENSG00000286054 (-21088), gene: ENSG00000286178 (-19133), MIR6724-4 (-10583), gene: ENSG00000278775 (-10030), gene: ENSG00000281181 (-4653), RNA5-8SN1 (-3229), gene: ENSG00000286149 (+19115), gene: ENSG00000286032 (+22049), gene: ENSG00000286091 (+23261), gene: ENSG00000286148 (+25115)

30927-001.R1 (paired) (Report)

Host chromosome	Unaligned ends virus	Broken reads host	Disrupted genes	Nearby genes
21	N/A	1	gene: ENSG00000278996, gene: ENSG00000280800	gene: ENSG00000278996, MIR6724-1 (-5871), gene: ENSG00000275664 (-5337), MIR3648-1 (-2625), gene: ENSG00000277437 (-2373), gene: ENSG00000280800, RNA5-8SN2 (+1294), gene: ENSG00000286155 (+24076), gene: ENSG00000286252 (+26371), gene: ENSG00000286057 (+27581), gene: ENSG00000286012 (+29458), MIR6724-2 (+38227), gene: ENSG00000277671 (+38782), gene: ENSG00000281383 (+43314), 5_8S_rRNA (+45503)
21	515	1	gene: ENSG00000278996	gene: ENSG00000278996, MIR6724-1 (-13407), gene: ENSG00000275664 (-12873), MIR3648-1 (-10161), gene: ENSG00000277437 (-9909), gene: ENSG00000280800 (-7507), RNA5-8SN2 (-6089), gene: ENSG00000286155 (+16540), gene: ENSG00000286252 (+18835), gene: ENSG00000286057 (+20045), gene: ENSG00000286012 (+21922), MIR6724-2 (+30691), gene: ENSG00000277671 (+31246), gene: ENSG00000281383 (+35778), 5_8S_rRNA (+37967)

1. Identify Viral Integration Sites summary

Input reads	1,303,712
Host reads	308,235
Virus reads	745,234
Unmapped reads	250,243
Host reads (%)	23.64
Virus reads (%)	57.16
Unmapped reads (%)	19.19
Breakpoints identified	34
Viruses identified	1

2. Identify Viral Integration Sites virus content

Virus	Reads mapped
HPV18REF	377,255

3. Identify Viral Integration Sites breakpoint summary

Host chromosome	Host region	Virus	Virus region	Unaligned ends host
8	complement (127218386.. 127218387)	HPV18REF	complement(5735.. 5736)	1,225
8	complement (127218809.. 127218810)	HPV18REF	23..24	190
8	127229125.. 127229126	HPV18REF	927..928	101
8	127229132.. 127229133	HPV18REF	927..928	1,819
8	127229306.. 127229307	HPV18REF	2497..2498	1,746
8	127221122.. 127221123	HPV18REF	3100..3101	304
21	complement (8260533..8260534)	HPV18REF	complement(1139.. 1140)	31
21	complement (8442306..8442307)	HPV18REF	6527..6528	29

30927-002.R1 (paired) (Report)

Host chromosome	Host region	Virus	Virus region	Unaligned ends host
21	complement (8442474..8442475)	HPV18REF	6766..6767	93
21	complement (8398891..8398892)	HPV18REF	109..110	25
21	complement (8442240..8442241)	HPV18REF	2177..2178	99
21	complement (8401610..8401611)	HPV18REF	1987..1988	238
21	complement (8401683..8401684)	HPV18REF	1298..1299	50
21	complement (8437076..8437077)	HPV18REF	715..716	29
21	complement (8437085..8437086)	HPV18REF	676..677	29
21	complement (8211100..8211101)	HPV18REF	901..902	44
21	complement (8442232..8442233)	HPV18REF	6042..6043	52
21	complement (8442332..8442333)	HPV18REF	842..843	27
21	complement (8442525..8442526)	HPV18REF	complement(495..496)	156
21	complement (8442968..8442969)	HPV18REF	2953..2954	64
21	complement (8215236..8215237)	HPV18REF	684..685	46
21	complement (8216355..8216356)	HPV18REF	1907..1908	26
21	complement (8445819..8445820)	HPV18REF	complement(1498..1499)	125
21	complement (8445981..8445982)	HPV18REF	761..762	42
21	complement (8441240..8441241)	HPV18REF	complement(1498..1499)	29
21	complement (8445842..8445843)	HPV18REF	2126..2127	186
21	complement (8218555..8218556)	HPV18REF	6622..6623	21
21	complement (8218572..8218573)	HPV18REF	complement(1817..1818)	178
21	complement (8218711..8218712)	HPV18REF	1038..1039	31
21	complement (8445933..8445934)	HPV18REF	complement(542..543)	25
21	complement (8442211..8442212)	HPV18REF	685..686	33

30927-002.R1 (paired) (Report)

Host chromosome	Host region	Virus	Virus region	Unaligned ends host
21	complement (8442352..8442353)	HPV18REF	235..236	26
21	8398660..8398661	HPV18REF	618..619	55
21	8437409..8437410	HPV18REF	7508..7509	55

Host chromosome	Unaligned ends virus	Broken reads host	Disrupted genes	Nearby genes
8	3,654	377	PCAT1, CASC19	PCAT1, CASC19, gene: ENSG00000287781 (+34826), CASC8 (+71430)
8	N/A	72	PCAT1, CASC19	PCAT1, CASC19, gene: ENSG00000287781 (+34403), CASC8 (+71007)
8	366	46	PCAT1	PCAT1, CASC19 (-1584), gene: ENSG00000287781 (+24087), CASC8 (+60691), POU5F1B (+93057)
8	366	343	PCAT1	PCAT1, CASC19 (-1591), gene: ENSG00000287781 (+24080), CASC8 (+60684), POU5F1B (+93050)
8	4,232	652	PCAT1	PCAT1, CASC19 (-1765), gene: ENSG00000287781 (+23906), CASC8 (+60510), POU5F1B (+92876)
8	995	49	PCAT1, CASC19	PCAT1, CASC19, gene: ENSG00000287781 (+32090), CASC8 (+68694)

30927-002.R1 (paired) (Report)

Host chromosome	Unaligned ends virus	Broken reads host	Disrupted genes	Nearby genes
21	2,121	1		gene: ENSG00000278996 (-32887), MIR6724-1 (-55127), gene: ENSG00000275664 (-54593), MIR3648-1 (-51881), gene: ENSG00000277437 (-51629), gene: ENSG00000280800 (-49227), RNA5-8SN2 (-47809), gene: ENSG00000286155 (-24701), gene: ENSG00000286252 (-22554), gene: ENSG00000286057 (-21441), gene: ENSG00000286012 (-19486), MIR6724-2 (-10937), gene: ENSG00000277671 (-10384), gene: ENSG00000281383 (-5019), 5_8S_rRNA (-3600)

30927-002.R1 (paired) (Report)

Host chromosome	Unaligned ends virus	Broken reads host	Disrupted genes	Nearby genes
21	448	9	gene: ENSG00000280441	gene: ENSG00000280441, MIR6724-3 (-53853), gene: ENSG00000274868 (-53319), gene: ENSG00000280614 (-47965), RNA5-8SN3 (-46547), gene: ENSG00000286267 (-24071), gene: ENSG00000286146 (-21303), gene: ENSG00000286054 (-20190), gene: ENSG00000286178 (-18235), MIR6724-4 (-9685), gene: ENSG00000278775 (-9132), gene: ENSG00000281181 (-3755), RNA5-8SN1 (-2331), gene: ENSG00000286149 (+20013), gene: ENSG00000286032 (+22947), gene: ENSG00000286091 (+24159), gene: ENSG00000286148 (+26013)

30927-002.R1 (paired) (Report)

Host chromosome	Unaligned ends virus	Broken reads host	Disrupted genes	Nearby genes
21	N/A	9	gene: ENSG00000280441	gene: ENSG00000280441, MIR6724-3 (-54021), gene: ENSG00000274868 (-53487), gene: ENSG00000280614 (-48133), RNA5-8SN3 (-46715), gene: ENSG00000286267 (-24239), gene: ENSG00000286146 (-21471), gene: ENSG00000286054 (-20358), gene: ENSG00000286178 (-18403), MIR6724-4 (-9853), gene: ENSG00000278775 (-9300), gene: ENSG00000281181 (-3923), RNA5-8SN1 (-2499), gene: ENSG00000286149 (+19845), gene: ENSG00000286032 (+22779), gene: ENSG00000286091 (+23991), gene: ENSG00000286148 (+25845)

30927-002.R1 (paired) (Report)

Host chromosome	Unaligned ends virus	Broken reads host	Disrupted genes	Nearby genes
21	N/A	9	gene: ENSG00000280441	gene: ENSG00000280441, MIR6724-3 (-10438), gene: ENSG00000274868 (-9904), gene: ENSG00000280614 (-4550), RNA5-8SN3 (-3132), gene: ENSG00000286267 (+18865), gene: ENSG00000286146 (+21783), gene: ENSG00000286054 (+22991), gene: ENSG00000286178 (+24868), MIR6724-4 (+33638), gene: ENSG00000278775 (+34193), gene: ENSG00000281181 (+38737), RNA5-8SN1 (+40931), gene: ENSG00000286149 (+63428), gene: ENSG00000286032 (+66362), gene: ENSG00000286091 (+67574), gene: ENSG00000286148 (+69428)

30927-002.R1 (paired) (Report)

Host chromosome	Unaligned ends virus	Broken reads host	Disrupted genes	Nearby genes
21	516	12	gene: ENSG00000280441	gene: ENSG00000280441, MIR6724-3 (-53787), gene: ENSG00000274868 (-53253), gene: ENSG00000280614 (-47899), RNA5-8SN3 (-46481), gene: ENSG00000286267 (-24005), gene: ENSG00000286146 (-21237), gene: ENSG00000286054 (-20124), gene: ENSG00000286178 (-18169), MIR6724-4 (-9619), gene: ENSG00000278775 (-9066), gene: ENSG00000281181 (-3689), RNA5-8SN1 (-2265), gene: ENSG00000286149 (+20079), gene: ENSG00000286032 (+23013), gene: ENSG00000286091 (+24225), gene: ENSG00000286148 (+26079)

30927-002.R1 (paired) (Report)

Host chromosome	Unaligned ends virus	Broken reads host	Disrupted genes	Nearby genes
21	400	5	gene: ENSG00000280441	gene: ENSG00000280441, MIR6724-3 (-13157), gene: ENSG00000274868 (-12623), gene: ENSG00000280614 (-7269), RNA5-8SN3 (-5851), gene: ENSG00000286267 (+16146), gene: ENSG00000286146 (+19064), gene: ENSG00000286054 (+20272), gene: ENSG00000286178 (+22149), MIR6724-4 (+30919), gene: ENSG00000278775 (+31474), gene: ENSG00000281181 (+36018), RNA5-8SN1 (+38212), gene: ENSG00000286149 (+60709), gene: ENSG00000286032 (+63643), gene: ENSG00000286091 (+64855), gene: ENSG00000286148 (+66709)

30927-002.R1 (paired) (Report)

Host chromosome	Unaligned ends virus	Broken reads host	Disrupted genes	Nearby genes
21	547	1	gene: ENSG00000280441	gene: ENSG00000280441, MIR6724-3 (-13230), gene: ENSG00000274868 (-12696), gene: ENSG00000280614 (-7342), RNA5-8SN3 (-5924), gene: ENSG00000286267 (+16073), gene: ENSG00000286146 (+18991), gene: ENSG00000286054 (+20199), gene: ENSG00000286178 (+22076), MIR6724-4 (+30846), gene: ENSG00000278775 (+31401), gene: ENSG00000281181 (+35945), RNA5-8SN1 (+38139), gene: ENSG00000286149 (+60636), gene: ENSG00000286032 (+63570), gene: ENSG00000286091 (+64782), gene: ENSG00000286148 (+66636)

30927-002.R1 (paired) (Report)

Host chromosome	Unaligned ends virus	Broken reads host	Disrupted genes	Nearby genes
21	870	3	gene: ENSG00000280441	gene: ENSG00000280441, MIR6724-3 (-48623), gene: ENSG00000274868 (-48089), gene: ENSG00000280614 (-42735), RNA5-8SN3 (-41317), gene: ENSG00000286267 (-18841), gene: ENSG00000286146 (-16073), gene: ENSG00000286054 (-14960), gene: ENSG00000286178 (-13005), MIR6724-4 (-4455), gene: ENSG00000278775 (-3902), gene: ENSG00000281181 (+552), RNA5-8SN1 (+2746), gene: ENSG00000286149 (+25243), gene: ENSG00000286032 (+28177), gene: ENSG00000286091 (+29389), gene: ENSG00000286148 (+31243)

Table S4 Viral integration sites reports

30927-002.R1 (paired) (Report)

Host chromosome	Unaligned ends virus	Broken reads host	Disrupted genes	Nearby genes
21	1,123	3	gene: ENSG00000280441	gene: ENSG00000280441, MIR6724-3 (-48632), gene: ENSG00000274868 (-48098), gene: ENSG00000280614 (-42744), RNA5-8SN3 (-41326), gene: ENSG00000286267 (-18850), gene: ENSG00000286146 (-16082), gene: ENSG00000286054 (-14969), gene: ENSG00000286178 (-13014), MIR6724-4 (-4464), gene: ENSG00000278775 (-3911), gene: ENSG00000281181 (+543), RNA5-8SN1 (+2737), gene: ENSG00000286149 (+25234), gene: ENSG00000286032 (+28168), gene: ENSG00000286091 (+29380), gene: ENSG00000286148 (+31234)

30927-002.R1 (paired) (Report)

Host chromosome	Unaligned ends virus	Broken reads host	Disrupted genes	Nearby genes
21	1,286	2	gene: ENSG00000278996, gene: ENSG00000280800	gene: ENSG00000278996, MIR6724-1 (-5694), gene: ENSG00000275664 (-5160), MIR3648-1 (-2448), gene: ENSG00000277437 (-2196), gene: ENSG00000280800, RNA5-8SN2 (+1471), gene: ENSG00000286155 (+24253), gene: ENSG00000286252 (+26548), gene: ENSG00000286057 (+27758), gene: ENSG00000286012 (+29635), MIR6724-2 (+38404), gene: ENSG00000277671 (+38959), gene: ENSG00000281383 (+43491), 5_8S_rRNA (+45680)

30927-002.R1 (paired) (Report)

Host chromosome	Unaligned ends virus	Broken reads host	Disrupted genes	Nearby genes
21	N/A	13	gene: ENSG00000280441	gene: ENSG00000280441, MIR6724-3 (-53779), gene: ENSG00000274868 (-53245), gene: ENSG00000280614 (-47891), RNA5-8SN3 (-46473), gene: ENSG00000286267 (-23997), gene: ENSG00000286146 (-21229), gene: ENSG00000286054 (-20116), gene: ENSG00000286178 (-18161), MIR6724-4 (-9611), gene: ENSG00000278775 (-9058), gene: ENSG00000281181 (-3681), RNA5-8SN1 (-2257), gene: ENSG00000286149 (+20087), gene: ENSG00000286032 (+23021), gene: ENSG00000286091 (+24233), gene: ENSG00000286148 (+26087)

30927-002.R1 (paired) (Report)

Host chromosome	Unaligned ends virus	Broken reads host	Disrupted genes	Nearby genes
21	965	8	gene: ENSG00000280441	gene: ENSG00000280441, MIR6724-3 (-53879), gene: ENSG00000274868 (-53345), gene: ENSG00000280614 (-47991), RNA5-8SN3 (-46573), gene: ENSG00000286267 (-24097), gene: ENSG00000286146 (-21329), gene: ENSG00000286054 (-20216), gene: ENSG00000286178 (-18261), MIR6724-4 (-9711), gene: ENSG00000278775 (-9158), gene: ENSG00000281181 (-3781), RNA5-8SN1 (-2357), gene: ENSG00000286149 (+19987), gene: ENSG00000286032 (+22921), gene: ENSG00000286091 (+24133), gene: ENSG00000286148 (+25987)

30927-002.R1 (paired) (Report)

Host chromosome	Unaligned ends virus	Broken reads host	Disrupted genes	Nearby genes
21	910	8	gene: ENSG00000280441	gene: ENSG00000280441, MIR6724-3 (-54072), gene: ENSG00000274868 (-53538), gene: ENSG00000280614 (-48184), RNA5-8SN3 (-46766), gene: ENSG00000286267 (-24290), gene: ENSG00000286146 (-21522), gene: ENSG00000286054 (-20409), gene: ENSG00000286178 (-18454), MIR6724-4 (-9904), gene: ENSG00000278775 (-9351), gene: ENSG00000281181 (-3974), RNA5-8SN1 (-2550), gene: ENSG00000286149 (+19794), gene: ENSG00000286032 (+22728), gene: ENSG00000286091 (+23940), gene: ENSG00000286148 (+25794)

30927-002.R1 (paired) (Report)

Host chromosome	Unaligned ends virus	Broken reads host	Disrupted genes	Nearby genes
21	N/A	6	gene: ENSG00000280441	gene: ENSG00000280441, MIR6724-3 (-54515), gene: ENSG00000274868 (-53981), gene: ENSG00000280614 (-48627), RNA5-8SN3 (-47209), gene: ENSG00000286267 (-24733), gene: ENSG00000286146 (-21965), gene: ENSG00000286054 (-20852), gene: ENSG00000286178 (-18897), MIR6724-4 (-10347), gene: ENSG00000278775 (-9794), gene: ENSG00000281181 (-4417), RNA5-8SN1 (-2993), gene: ENSG00000286149 (+19351), gene: ENSG00000286032 (+22285), gene: ENSG00000286091 (+23497), gene: ENSG00000286148 (+25351)

30927-002.R1 (paired) (Report)

Host chromosome	Unaligned ends virus	Broken reads host	Disrupted genes	Nearby genes
21	707	9	gene: ENSG00000278996	gene: ENSG00000278996, MIR6724-1 (-9830), gene: ENSG00000275664 (-9296), MIR3648-1 (-6584), gene: ENSG00000277437 (-6332), gene: ENSG00000280800 (-3930), RNA5-8SN2 (-2512), gene: ENSG00000286155 (+20117), gene: ENSG00000286252 (+22412), gene: ENSG00000286057 (+23622), gene: ENSG00000286012 (+25499), MIR6724-2 (+34268), gene: ENSG00000277671 (+34823), gene: ENSG00000281383 (+39355), 5_8S_rRNA (+41544)
21	414	3	gene: ENSG00000278996	gene: ENSG00000278996, MIR6724-1 (-10949), gene: ENSG00000275664 (-10415), MIR3648-1 (-7703), gene: ENSG00000277437 (-7451), gene: ENSG00000280800 (-5049), RNA5-8SN2 (-3631), gene: ENSG00000286155 (+18998), gene: ENSG00000286252 (+21293), gene: ENSG00000286057 (+22503), gene: ENSG00000286012 (+24380), MIR6724-2 (+33149), gene: ENSG00000277671 (+33704), gene: ENSG00000281383 (+38236), 5_8S_rRNA (+40425)

30927-002.R1 (paired) (Report)

Host chromosome	Unaligned ends virus	Broken reads host	Disrupted genes	Nearby genes
21	1,532	9	gene: ENSG00000280441	gene: ENSG00000280441, MIR6724-3 (-57366), gene: ENSG00000274868 (-56832), gene: ENSG00000280614 (-51478), RNA5-8SN3 (-50060), gene: ENSG00000286267 (-27584), gene: ENSG00000286146 (-24816), gene: ENSG00000286054 (-23703), gene: ENSG00000286178 (-21748), MIR6724-4 (-13198), gene: ENSG00000278775 (-12645), gene: ENSG00000281181 (-7268), RNA5-8SN1 (-5844), gene: ENSG00000286149 (+16500), gene: ENSG00000286032 (+19434), gene: ENSG00000286091 (+20646), gene: ENSG00000286148 (+22500)

30927-002.R1 (paired) (Report)

Host chromosome	Unaligned ends virus	Broken reads host	Disrupted genes	Nearby genes
21	678	1	gene: ENSG00000280441	gene: ENSG00000280441, MIR6724-3 (-57528), gene: ENSG00000274868 (-56994), gene: ENSG00000280614 (-51640), RNA5-8SN3 (-50222), gene: ENSG00000286267 (-27746), gene: ENSG00000286146 (-24978), gene: ENSG00000286054 (-23865), gene: ENSG00000286178 (-21910), MIR6724-4 (-13360), gene: ENSG00000278775 (-12807), gene: ENSG00000281181 (-7430), RNA5-8SN1 (-6006), gene: ENSG00000286149 (+16338), gene: ENSG00000286032 (+19272), gene: ENSG00000286091 (+20484), gene: ENSG00000286148 (+22338)

30927-002.R1 (paired) (Report)

Host chromosome	Unaligned ends virus	Broken reads host	Disrupted genes	Nearby genes
21	1,532	4	gene: ENSG00000280441	gene: ENSG00000280441, MIR6724-3 (-52787), gene: ENSG00000274868 (-52253), gene: ENSG00000280614 (-46899), RNA5-8SN3 (-45481), gene: ENSG00000286267 (-23005), gene: ENSG00000286146 (-20237), gene: ENSG00000286054 (-19124), gene: ENSG00000286178 (-17169), MIR6724-4 (-8619), gene: ENSG00000278775 (-8066), gene: ENSG00000281181 (-2689), RNA5-8SN1 (-1265), gene: ENSG00000286149 (+21079), gene: ENSG00000286032 (+24013), gene: ENSG00000286091 (+25225), gene: ENSG00000286148 (+27079)

30927-002.R1 (paired) (Report)

Host chromosome	Unaligned ends virus	Broken reads host	Disrupted genes	Nearby genes
21	N/A	7	gene: ENSG00000280441	gene: ENSG00000280441, MIR6724-3 (-57389), gene: ENSG00000274868 (-56855), gene: ENSG00000280614 (-51501), RNA5-8SN3 (-50083), gene: ENSG00000286267 (-27607), gene: ENSG00000286146 (-24839), gene: ENSG00000286054 (-23726), gene: ENSG00000286178 (-21771), MIR6724-4 (-13221), gene: ENSG00000278775 (-12668), gene: ENSG00000281181 (-7291), RNA5-8SN1 (-5867), gene: ENSG00000286149 (+16477), gene: ENSG00000286032 (+19411), gene: ENSG00000286091 (+20623), gene: ENSG00000286148 (+22477)

30927-002.R1 (paired) (Report)

Host chromosome	Unaligned ends virus	Broken reads host	Disrupted genes	Nearby genes
21	N/A	6	gene: ENSG00000278996	gene: ENSG00000278996, MIR6724-1 (-13149), gene: ENSG00000275664 (-12615), MIR3648-1 (-9903), gene: ENSG00000277437 (-9651), gene: ENSG00000280800 (-7249), RNA5-8SN2 (-5831), gene: ENSG00000286155 (+16798), gene: ENSG00000286252 (+19093), gene: ENSG00000286057 (+20303), gene: ENSG00000286012 (+22180), MIR6724-2 (+30949), gene: ENSG00000277671 (+31504), gene: ENSG00000281383 (+36036), 5_8S_rRNA (+38225)
21	2,579	6	gene: ENSG00000278996	gene: ENSG00000278996, MIR6724-1 (-13166), gene: ENSG00000275664 (-12632), MIR3648-1 (-9920), gene: ENSG00000277437 (-9668), gene: ENSG00000280800 (-7266), RNA5-8SN2 (-5848), gene: ENSG00000286155 (+16781), gene: ENSG00000286252 (+19076), gene: ENSG00000286057 (+20286), gene: ENSG00000286012 (+22163), MIR6724-2 (+30932), gene: ENSG00000277671 (+31487), gene: ENSG00000281383 (+36019), 5_8S_rRNA (+38208)

30927-002.R1 (paired) (Report)

Host chromosome	Unaligned ends virus	Broken reads host	Disrupted genes	Nearby genes
21	613	1	gene: ENSG00000278996	gene: ENSG00000278996, MIR6724-1 (-13305), gene: ENSG00000275664 (-12771), MIR3648-1 (-10059), gene: ENSG00000277437 (-9807), gene: ENSG00000280800 (-7405), RNA5-8SN2 (-5987), gene: ENSG00000286155 (+16642), gene: ENSG00000286252 (+18937), gene: ENSG00000286057 (+20147), gene: ENSG00000286012 (+22024), MIR6724-2 (+30793), gene: ENSG00000277671 (+31348), gene: ENSG00000281383 (+35880), 5_8S_rRNA (+38069)

30927-002.R1 (paired) (Report)

Host chromosome	Unaligned ends virus	Broken reads host	Disrupted genes	Nearby genes
21	1,642	2	gene: ENSG00000280441	gene: ENSG00000280441, MIR6724-3 (-57480), gene: ENSG00000274868 (-56946), gene: ENSG00000280614 (-51592), RNA5-8SN3 (-50174), gene: ENSG00000286267 (-27698), gene: ENSG00000286146 (-24930), gene: ENSG00000286054 (-23817), gene: ENSG00000286178 (-21862), MIR6724-4 (-13312), gene: ENSG00000278775 (-12759), gene: ENSG00000281181 (-7382), RNA5-8SN1 (-5958), gene: ENSG00000286149 (+16386), gene: ENSG00000286032 (+19320), gene: ENSG00000286091 (+20532), gene: ENSG00000286148 (+22386)

30927-002.R1 (paired) (Report)

Host chromosome	Unaligned ends virus	Broken reads host	Disrupted genes	Nearby genes
21	627	14	gene: ENSG00000280441	gene: ENSG00000280441, MIR6724-3 (-53758), gene: ENSG00000274868 (-53224), gene: ENSG00000280614 (-47870), RNA5-8SN3 (-46452), gene: ENSG00000286267 (-23976), gene: ENSG00000286146 (-21208), gene: ENSG00000286054 (-20095), gene: ENSG00000286178 (-18140), MIR6724-4 (-9590), gene: ENSG00000278775 (-9037), gene: ENSG00000281181 (-3660), RNA5-8SN1 (-2236), gene: ENSG00000286149 (+20108), gene: ENSG00000286032 (+23042), gene: ENSG00000286091 (+24254), gene: ENSG00000286148 (+26108)

30927-002.R1 (paired) (Report)

Host chromosome	Unaligned ends virus	Broken reads host	Disrupted genes	Nearby genes
21	1,782	8	gene: ENSG00000280441	gene: ENSG00000280441, MIR6724-3 (-53899), gene: ENSG00000274868 (-53365), gene: ENSG00000280614 (-48011), RNA5-8SN3 (-46593), gene: ENSG00000286267 (-24117), gene: ENSG00000286146 (-21349), gene: ENSG00000286054 (-20236), gene: ENSG00000286178 (-18281), MIR6724-4 (-9731), gene: ENSG00000278775 (-9178), gene: ENSG00000281181 (-3801), RNA5-8SN1 (-2377), gene: ENSG00000286149 (+19967), gene: ENSG00000286032 (+22901), gene: ENSG00000286091 (+24113), gene: ENSG00000286148 (+25967)

30927-002.R1 (paired) (Report)

Host chromosome	Unaligned ends virus	Broken reads host	Disrupted genes	Nearby genes
21	531	6	gene: ENSG00000280441	gene: ENSG00000280441, MIR6724-3 (-10207), gene: ENSG00000274868 (-9673), gene: ENSG00000280614 (-4319), RNA5-8SN3 (-2901), gene: ENSG00000286267 (+19096), gene: ENSG00000286146 (+22014), gene: ENSG00000286054 (+23222), gene: ENSG00000286178 (+25099), MIR6724-4 (+33869), gene: ENSG00000278775 (+34424), gene: ENSG00000281181 (+38968), RNA5-8SN1 (+41162), gene: ENSG00000286149 (+63659), gene: ENSG00000286032 (+66593), gene: ENSG00000286091 (+67805), gene: ENSG00000286148 (+69659)

30927-002.R1 (paired) (Report)

Host chromosome	Unaligned ends virus	Broken reads host	Disrupted genes	Nearby genes
21	N/A	4	gene: ENSG00000280441	gene: ENSG00000280441, MIR6724-3 (-48956), gene: ENSG00000274868 (-48422), gene: ENSG00000280614 (-43068), RNA5-8SN3 (-41650), gene: ENSG00000286267 (-19174), gene: ENSG00000286146 (-16406), gene: ENSG00000286054 (-15293), gene: ENSG00000286178 (-13338), MIR6724-4 (-4788), gene: ENSG00000278775 (-4235), gene: ENSG00000281181 (+219), RNA5-8SN1 (+2413), gene: ENSG00000286149 (+24910), gene: ENSG00000286032 (+27844), gene: ENSG00000286091 (+29056), gene: ENSG00000286148 (+30910)

30927-003.R1 (paired) Viral Reads (Report)

1. Identify Viral Integration Sites summary

Input reads	15,544,680
Host reads	5,078
Virus reads	15,539,434
Unmapped reads	168
Host reads (%)	0.03
Virus reads (%)	99.97
Unmapped reads (%)	0.00
Breakpoints identified	18
Viruses identified	1

2. Identify Viral Integration Sites virus content

Virus	Reads mapped
HPV16REF	7,770,544

3. Identify Viral Integration Sites breakpoint summary

Host chromosome	Host region	Virus	Virus region	Unaligned ends host
2	complement (22416229..22416230)	HPV16REF	7261..7262	73
2	complement (27137148..27137149)	HPV16REF	7902..7903	31
2	27136178..27136179	HPV16REF	1367..1368	47
2	22422774..22422775	HPV16REF	5402..5403	21
3	140761171.. 140761172	HPV16REF	105..106	25
6	45691388..45691389	HPV16REF	3729..3730	308
10	complement (11700484..11700485)	HPV16REF	5667..5668	36
11	complement (6741029..6741030)	HPV16REF	4592..4593	25
14	complement (47515164..47515165)	HPV16REF	complement(469..470)	27
19	complement (55307400..55307401)	HPV16REF	6906..6907	72
19	55310208..55310209	HPV16REF	581..582	28

30927-003.R1 (paired) Viral Reads (Report)

Host chromosome	Host region	Virus	Virus region	Unaligned ends host
19	55310219..55310220	HPV16REF	3521..3522	39
20	complement (26358219..26358220)	HPV16REF	4443..4444	20
20	complement (26276706..26276707)	HPV16REF	complement(469..470)	131
20	26357922..26357923	HPV16REF	7122..7123	80
X	complement (145693637.. 145693638)	HPV16REF	1453..1454	24
X	complement (145708230.. 145708231)	HPV16REF	2986..2987	52
X	145716514.. 145716515	HPV16REF	3156..3157	72

Host chromosome	Unaligned ends virus	Broken reads host	Disrupted genes	Nearby genes
2	N/A	21	gene: ENSG00000228999, LINC01830	gene: ENSG00000228999, LINC01830, LINC01884 (+91899)
2	N/A	7	PRR30	TMEM214 (-95454), AGBL5 (-66526), AGBL5-AS1 (-86884), gene: ENSG00000272056 (-82872), AGBL5-IT1 (-75333), gene: ENSG00000272148 (-74241), OST4 (-65494), EMILIN1 (-50745), KHK (-36386), CGREF1 (-18020), ABHD1 (-6336), PREB (-2482), PRR30, TCF23 (+11855), SLC5A6 (+62438), ATRAID (+74878), CAD (+80220)

Table S4 Viral integration sites reports

30927-003.R1 (paired) Viral Reads (Report)

Host chromosome	Unaligned ends virus	Broken reads host	Disrupted genes	Nearby genes
2	N/A	11		TMEM214 (-94484), AGBL5 (-65556), AGBL5-AS1 (-85914), gene: ENSG00000272056 (-81902), AGBL5-IT1 (-74363), gene: ENSG00000272148 (-73271), OST4 (-64524), EMILIN1 (-49775), KHK (-35416), CGREF1 (-17050), ABHD1 (-5366), PREB (-1512), PRR30 (+669), TCF23 (+12825), SLC5A6 (+63408), ATRAID (+75848), CAD (+81190)
2	N/A	1	gene: ENSG00000228999, LINC01830	gene: ENSG00000228999, LINC01830, LINC01884 (+85354)
3	N/A	5		TRIM42 (-60021)
6	8,488	55		RUNX2 (-27039)
10	N/A	10		USP6NL (-88730), USP6NL-AS1 (-88257), gene: ENSG00000271046 (-19970), ECHDC3 (+41881)
11	7,695	1		DCHS1 (-85220), gene: ENSG00000255410 (-82633), MRPL17 (-57689), OR2AG2 (+24596), OR2AG1 (+41990), OR6A2 (+50706), gene: ENSG00000283415 (+80912)
14	65,616	5	MDGA2	MDGA2

30927-003.R1 (paired) Viral Reads (Report)

Host chromosome	Unaligned ends virus	Broken reads host	Disrupted genes	Nearby genes
19	61,025	8	BRSK1	PTPRH (-97894), gene: ENSG00000286230 (-90119), gene: ENSG00000267649 (-85784), TMEM86B (-78616), gene: ENSG00000276570 (-77121), PPP6R1 (-48383), MIR6804 (-76448), MIR6802 (-67424), MIR6803 (-62150), HSPBP1 (-27019), BRSK1, gene: ENSG00000268729 (+4628), TMEM150B (+5400), gene: ENSG00000276831 (+31544), KMT5C (+32452), COX6B2 (+41905), gene: ENSG00000267706 (+44900), FAM71E2 (+47507), IL11 (+56981), TMEM190 (+69425), gene: ENSG00000269275 (+70178), TMEM238 (+71843), RPL28 (+78531), MIR6805 (+80780), UBE2S (+92344)

30927-003.R1 (paired) Viral Reads (Report)

Host chromosome	Unaligned ends virus	Broken reads host	Disrupted genes	Nearby genes
19	N/A	4	BRSK1	gene: ENSG00000286230 (-92927), gene: ENSG00000267649 (-88592), TMEM86B (-81424), gene: ENSG00000276570 (-79929), PPP6R1 (-51191), MIR6804 (-79256), MIR6802 (-70232), MIR6803 (-64958), HSPBP1 (-29827), BRSK1, gene: ENSG00000268729 (+1820), TMEM150B (+2592), gene: ENSG00000276831 (+28736), KMT5C (+29644), COX6B2 (+39097), gene: ENSG00000267706 (+42092), FAM71E2 (+44699), IL11 (+54173), TMEM190 (+66617), gene: ENSG00000269275 (+67370), TMEM238 (+69035), RPL28 (+75723), MIR6805 (+77972), UBE2S (+89536)

30927-003.R1 (paired) Viral Reads (Report)

Host chromosome	Unaligned ends virus	Broken reads host	Disrupted genes	Nearby genes
19	4,370	7	BRSK1	gene: ENSG00000286230 (-92938), gene: ENSG00000267649 (-88603), TMEM86B (-81435), gene: ENSG00000276570 (-79940), PPP6R1 (-51202), MIR6804 (-79267), MIR6802 (-70243), MIR6803 (-64969), HSPBP1 (-29838), BRSK1, gene: ENSG00000268729 (+1809), TMEM150B (+2581), gene: ENSG00000276831 (+28725), KMT5C (+29633), COX6B2 (+39086), gene: ENSG00000267706 (+42081), FAM71E2 (+44688), IL11 (+54162), TMEM190 (+66606), gene: ENSG00000269275 (+67359), TMEM238 (+69024), RPL28 (+75712), MIR6805 (+77961), UBE2S (+89525)
20	N/A	8		
20	65,616	31		gene: ENSG00000287211 (-88515), MIR663AHG (-25160), gene: ENSG00000238129 (-79815), MIR663A (-68428)
20	N/A	34		
X	N/A	6		gene: ENSG00000286085 (+5418)
X	N/A	7	gene: ENSG00000286085	gene: ENSG00000286085, gene: ENSG00000279585 (+89144)
X	N/A	18	gene: ENSG00000286085	gene: ENSG00000286085, gene: ENSG00000279585 (+80860)

30927-004.R1 (paired) (Report)

1. Identify Viral Integration Sites summary

Input reads	447,876
Host reads	325,335
Virus reads	55
Unmapped reads	122,486
Host reads (%)	72.64
Virus reads (%)	0.01
Unmapped reads (%)	27.35
Breakpoints identified	0
Viruses identified	1

2. Identify Viral Integration Sites virus content

Virus	Reads mapped
HPV16REF	50

3. Identify Viral Integration Sites breakpoint summary

30927-005.R1 (paired) (Report)

1. Identify Viral Integration Sites summary

Input reads	10,317,948
Host reads	1,821,445
Virus reads	7,132,416
Unmapped reads	1,364,087
Host reads (%)	17.65
Virus reads (%)	69.13
Unmapped reads (%)	13.22
Breakpoints identified	392
Viruses identified	1

2. Identify Viral Integration Sites virus content

Virus	Reads mapped
HPV16REF	3,602,533

3. Identify Viral Integration Sites breakpoint summary

Host chromosome	Host region	Virus	Virus region	Unaligned ends host
2	32916559..32916560	HPV16REF	339..340	28
2	32916625..32916626	HPV16REF	complement(7729..7730)	32
3	complement (15738565..15738566)	HPV16REF	2198..2199	25
3	15738691..15738692	HPV16REF	complement(7684..7685)	58
4	complement (18330469..18330470)	HPV16REF	complement(795..796)	285
4	18330457..18330458	HPV16REF	3049..3050	265
4	57209788..57209789	HPV16REF	1996..1997	225
6	complement (33315829..33315830)	HPV16REF	4293..4294	47
6	complement (33315831..33315832)	HPV16REF	5745..5746	69
6	33316080..33316081	HPV16REF	5714..5715	29
6	33316430..33316431	HPV16REF	6028..6029	36

30927-005.R1 (paired) (Report)

Host chromosome	Host region	Virus	Virus region	Unaligned ends host
6	33316399..33316400	HPV16REF	complement(3290.. 3291)	38
7	complement (93905525..93905526)	HPV16REF	5633..5634	55
7	93902553..93902554	HPV16REF	1569..1570	359
8	complement (42207493..42207494)	HPV16REF	complement(3362.. 3363)	46
8	complement (42216373..42216374)	HPV16REF	1466..1467	140
8	complement (42216471..42216472)	HPV16REF	complement(3362.. 3363)	47
8	144140102.. 144140103	HPV16REF	7870..7871	61
8	42216345..42216346	HPV16REF	complement(1207.. 1208)	298
8	42189073..42189074	HPV16REF	112..113	451
8	42193213..42193214	HPV16REF	226..227	59
8	42193214..42193215	HPV16REF	880..881	74
8	144140091.. 144140092	HPV16REF	3591..3592	144
8	144140115.. 144140116	HPV16REF	2794..2795	87
8	144140117.. 144140118	HPV16REF	3515..3516	55
8	144140149.. 144140150	HPV16REF	complement(3290.. 3291)	177
8	144140175.. 144140176	HPV16REF	1685..1686	115
8	144140183.. 144140184	HPV16REF	3962..3963	57
8	144140188.. 144140189	HPV16REF	2232..2233	92
8	144140201.. 144140202	HPV16REF	complement(5268.. 5269)	81
8	144140211.. 144140212	HPV16REF	complement(4606.. 4607)	82
8	144140217.. 144140218	HPV16REF	complement(3290.. 3291)	124
8	144140248.. 144140249	HPV16REF	2317..2318	87
8	144140249.. 144140250	HPV16REF	1959..1960	69
9	complement (9442110..9442111)	HPV16REF	complement(3787.. 3788)	170
9	complement (9442114..9442115)	HPV16REF	6324..6325	262

30927-005.R1 (paired) (Report)

Host chromosome	Host region	Virus	Virus region	Unaligned ends host
9	9442236..9442237	HPV16REF	3723..3724	89
9	9442238..9442239	HPV16REF	6688..6689	81
9	9442255..9442256	HPV16REF	complement(5268.. 5269)	29
9	9442260..9442261	HPV16REF	2227..2228	29
9	9442264..9442265	HPV16REF	3126..3127	64
9	35657937..35657938	HPV16REF	complement(2059.. 2060)	75
9	9442268..9442269	HPV16REF	6503..6504	95
10	complement (133251485.. 133251486)	HPV16REF	2695..2696	137
10	complement (133284912.. 133284913)	HPV16REF	4358..4359	318
10	complement (119525968.. 119525969)	HPV16REF	1795..1796	389
10	complement (119527304.. 119527305)	HPV16REF	934..935	390
10	133251121.. 133251122	HPV16REF	complement(7729.. 7730)	198
10	119525903.. 119525904	HPV16REF	6212..6213	463
10	119527427.. 119527428	HPV16REF	678..679	82
10	133297668.. 133297669	HPV16REF	3584..3585	293
14	complement (20343089..20343090)	HPV16REF	1753..1754	158
14	complement (49862640..49862641)	HPV16REF	2638..2639	71
14	complement (49586579..49586580)	HPV16REF	2281..2282	518
14	complement (49586598..49586599)	HPV16REF	complement(7902.. 7903)	1,104
14	complement (49586600..49586601)	HPV16REF	complement(3787.. 3788)	88
14	complement (49586617..49586618)	HPV16REF	5810..5811	65
14	complement (49586622..49586623)	HPV16REF	3619..3620	65
14	complement (49586625..49586626)	HPV16REF	2191..2192	99

30927-005.R1 (paired) (Report)

Host chromosome	Host region	Virus	Virus region	Unaligned ends host
14	complement (49586634..49586635)	HPV16REF	complement(7069.. 7070)	1,226
14	complement (49586635..49586636)	HPV16REF	complement(4606.. 4607)	120
14	complement (49586636..49586637)	HPV16REF	1673..1674	266
14	complement (49586647..49586648)	HPV16REF	5961..5962	1,234
14	complement (49586649..49586650)	HPV16REF	complement(843..844)	478
14	complement (49586667..49586668)	HPV16REF	7588..7589	104
14	complement (49586675..49586676)	HPV16REF	complement(2059.. 2060)	90
14	complement (49586712..49586713)	HPV16REF	7496..7497	82
14	complement (49586688..49586689)	HPV16REF	3515..3516	76
14	complement (49862585..49862586)	HPV16REF	4017..4018	124
14	complement (49586599..49586600)	HPV16REF	7558..7559	914
14	complement (49862568..49862569)	HPV16REF	complement(2059.. 2060)	66
14	complement (49862571..49862572)	HPV16REF	4926..4927	113
14	complement (49862573..49862574)	HPV16REF	complement(4606.. 4607)	255
14	complement (49862574..49862575)	HPV16REF	4707..4708	71
14	complement (49862577..49862578)	HPV16REF	3104..3105	149
14	complement (49862590..49862591)	HPV16REF	1466..1467	193
14	complement (20343072..20343073)	HPV16REF	1722..1723	101
14	complement (20343088..20343089)	HPV16REF	3684..3685	164
14	complement (20343160..20343161)	HPV16REF	6244..6245	94
14	49586729..49586730	HPV16REF	3201..3202	146
14	49586732..49586733	HPV16REF	3585..3586	87
14	49586744..49586745	HPV16REF	6174..6175	62
14	49586755..49586756	HPV16REF	1413..1414	66
14	49586756..49586757	HPV16REF	complement(3362.. 3363)	256

30927-005.R1 (paired) (Report)

Host chromosome	Host region	Virus	Virus region	Unaligned ends host
14	49586757..49586758	HPV16REF	complement(7729..7730)	237
14	49586758..49586759	HPV16REF	6688..6689	174
14	49586759..49586760	HPV16REF	2637..2638	165
14	49586760..49586761	HPV16REF	complement(2059..2060)	100
14	49586764..49586765	HPV16REF	complement(524..525)	48
14	49586767..49586768	HPV16REF	5467..5468	59
14	49586784..49586785	HPV16REF	complement(7902..7903)	83
14	49586785..49586786	HPV16REF	7186..7187	167
14	49586801..49586802	HPV16REF	5584..5585	101
14	49586817..49586818	HPV16REF	3515..3516	45
14	49586838..49586839	HPV16REF	complement(3449..3450)	528
14	49586839..49586840	HPV16REF	1697..1698	388
14	49586840..49586841	HPV16REF	1413..1414	225
14	49586843..49586844	HPV16REF	1590..1591	292
14	49586854..49586855	HPV16REF	678..679	561
14	49586855..49586856	HPV16REF	1676..1677	281
14	49586857..49586858	HPV16REF	2460..2461	616
14	49586868..49586869	HPV16REF	7336..7337	240
14	49586871..49586872	HPV16REF	complement(524..525)	81
14	49586872..49586873	HPV16REF	5762..5763	98
14	20343298..20343299	HPV16REF	complement(7045..7046)	55
14	49862738..49862739	HPV16REF	complement(7729..7730)	285
14	49862740..49862741	HPV16REF	3515..3516	36
14	49862759..49862760	HPV16REF	3126..3127	33
14	49862761..49862762	HPV16REF	7588..7589	74
14	49862783..49862784	HPV16REF	7312..7313	69
14	49862800..49862801	HPV16REF	complement(7045..7046)	52
14	49862804..49862805	HPV16REF	complement(2059..2060)	100
14	20343292..20343293	HPV16REF	complement(5268..5269)	99
14	49586779..49586780	HPV16REF	complement(3787..3788)	197
14	20343387..20343388	HPV16REF	112..113	31
14	49862779..49862780	HPV16REF	2799..2800	192

30927-005.R1 (paired) (Report)

Host chromosome	Host region	Virus	Virus region	Unaligned ends host
16	complement (634435..634436)	HPV16REF	5952..5953	335
16	complement (634459..634460)	HPV16REF	303..304	150
16	complement (634464..634465)	HPV16REF	1830..1831	126
16	complement (634481..634482)	HPV16REF	complement(7684.. 7685)	214
16	complement (634487..634488)	HPV16REF	3706..3707	167
16	complement (634494..634495)	HPV16REF	3220..3221	830
16	complement (634497..634498)	HPV16REF	2279..2280	201
16	complement (634501..634502)	HPV16REF	7249..7250	297
16	complement (634504..634505)	HPV16REF	3058..3059	1,114
16	complement (634510..634511)	HPV16REF	complement(3290.. 3291)	251
16	complement (634523..634524)	HPV16REF	2526..2527	169
16	complement (634524..634525)	HPV16REF	complement(406..407)	71
16	complement (634480..634481)	HPV16REF	140..141	257
21	complement (8258196..8258197)	HPV16REF	2764..2765	132
21	complement (8258203..8258204)	HPV16REF	7870..7871	77
21	complement (8258221..8258222)	HPV16REF	7403..7404	58
21	complement (8259438..8259439)	HPV16REF	complement(1207.. 1208)	118
21	complement (8259441..8259442)	HPV16REF	4448..4449	58
21	complement (8259461..8259462)	HPV16REF	complement(7760.. 7761)	82
21	complement (8259465..8259466)	HPV16REF	complement(524..525)	226
21	complement (8259473..8259474)	HPV16REF	3230..3231	272
21	complement (8259475..8259476)	HPV16REF	6912..6913	149
21	complement (8259491..8259492)	HPV16REF	complement(5268.. 5269)	662

30927-005.R1 (paired) (Report)

Host chromosome	Host region	Virus	Virus region	Unaligned ends host
21	complement (8259493..8259494)	HPV16REF	1918..1919	119
21	complement (8259503..8259504)	HPV16REF	3854..3855	58
21	complement (8259586..8259587)	HPV16REF	7968..7969	136
21	complement (8259594..8259595)	HPV16REF	6003..6004	135
21	complement (8259604..8259605)	HPV16REF	3595..3596	62
21	complement (8259630..8259631)	HPV16REF	1374..1375	148
21	complement (8259638..8259639)	HPV16REF	3887..3888	454
21	complement (8259706..8259707)	HPV16REF	678..679	88
21	complement (8259904..8259905)	HPV16REF	1894..1895	92
21	complement (8260008..8260009)	HPV16REF	complement(795..796)	131
21	complement (8260084..8260085)	HPV16REF	678..679	143
21	complement (8260086..8260087)	HPV16REF	7623..7624	139
21	complement (8260543..8260544)	HPV16REF	1802..1803	54
21	complement (8260550..8260551)	HPV16REF	3685..3686	107
21	complement (8260558..8260559)	HPV16REF	1653..1654	66
21	complement (8260658..8260659)	HPV16REF	1413..1414	69
21	complement (8392858..8392859)	HPV16REF	complement(524..525)	120
21	complement (8392879..8392880)	HPV16REF	6581..6582	110
21	complement (8392904..8392905)	HPV16REF	complement(5268.. 5269)	297
21	complement (8394054..8394055)	HPV16REF	3515..3516	55
21	complement (8394060..8394061)	HPV16REF	complement(524..525)	100
21	complement (8394113..8394114)	HPV16REF	complement(7786.. 7787)	186
21	complement (8397013..8397014)	HPV16REF	complement(7760.. 7761)	142

30927-005.R1 (paired) (Report)

Host chromosome	Host region	Virus	Virus region	Unaligned ends host
21	complement (8397014..8397015)	HPV16REF	7277..7278	138
21	complement (8397020..8397021)	HPV16REF	7870..7871	53
21	complement (8397046..8397047)	HPV16REF	112..113	95
21	complement (8397358..8397359)	HPV16REF	3599..3600	283
21	complement (8398245..8398246)	HPV16REF	2206..2207	468
21	complement (8398253..8398254)	HPV16REF	6413..6414	66
21	complement (8398263..8398264)	HPV16REF	2402..2403	76
21	complement (8398265..8398266)	HPV16REF	4087..4088	143
21	complement (8398270..8398271)	HPV16REF	934..935	240
21	complement (8398273..8398274)	HPV16REF	998..999	63
21	complement (8398278..8398279)	HPV16REF	2471..2472	277
21	complement (8398296..8398297)	HPV16REF	3875..3876	648
21	complement (8398314..8398315)	HPV16REF	complement(3290.. 3291)	170
21	complement (8398337..8398338)	HPV16REF	112..113	76
21	complement (8398344..8398345)	HPV16REF	4366..4367	56
21	complement (8398363..8398364)	HPV16REF	1767..1768	69
21	complement (8398414..8398415)	HPV16REF	1014..1015	129
21	complement (8398420..8398421)	HPV16REF	3065..3066	117
21	complement (8398434..8398435)	HPV16REF	complement(524..525)	128
21	complement (8398464..8398465)	HPV16REF	112..113	181
21	complement (8398481..8398482)	HPV16REF	3515..3516	148
21	complement (8398508..8398509)	HPV16REF	complement(3787.. 3788)	85
21	complement (8398721..8398722)	HPV16REF	4708..4709	56

30927-005.R1 (paired) (Report)

Host chromosome	Host region	Virus	Virus region	Unaligned ends host
21	complement (8398894..8398895)	HPV16REF	complement(1225.. 1226)	95
21	complement (8398895..8398896)	HPV16REF	1466..1467	98
21	complement (8399067..8399068)	HPV16REF	6688..6689	133
21	complement (8399338..8399339)	HPV16REF	3962..3963	142
21	complement (8399352..8399353)	HPV16REF	complement(406..407)	62
21	complement (8399441..8399442)	HPV16REF	1861..1862	309
21	complement (8399735..8399736)	HPV16REF	2003..2004	97
21	complement (8399805..8399806)	HPV16REF	1518..1519	60
21	complement (8400913..8400914)	HPV16REF	complement(4606.. 4607)	67
21	complement (8401554..8401555)	HPV16REF	112..113	101
21	complement (8401560..8401561)	HPV16REF	5545..5546	84
21	complement (8401587..8401588)	HPV16REF	6390..6391	230
21	complement (8401610..8401611)	HPV16REF	2658..2659	281
21	complement (8401669..8401670)	HPV16REF	2483..2484	78
21	complement (8401674..8401675)	HPV16REF	6559..6560	83
21	complement (8401675..8401676)	HPV16REF	226..227	86
21	complement (8401697..8401698)	HPV16REF	complement(4606.. 4607)	180
21	complement (8401699..8401700)	HPV16REF	1017..1018	150
21	complement (8401708..8401709)	HPV16REF	4190..4191	222
21	complement (8401718..8401719)	HPV16REF	1143..1144	71
21	complement (8401721..8401722)	HPV16REF	4025..4026	119
21	complement (8401725..8401726)	HPV16REF	2887..2888	75
21	complement (8401736..8401737)	HPV16REF	6205..6206	73

30927-005.R1 (paired) (Report)

Host chromosome	Host region	Virus	Virus region	Unaligned ends host
21	complement (8442299..8442300)	HPV16REF	6124..6125	232
21	complement (8210002..8210003)	HPV16REF	4317..4318	159
21	complement (8211025..8211026)	HPV16REF	112..113	93
21	complement (8211078..8211079)	HPV16REF	3851..3852	178
21	complement (8211100..8211101)	HPV16REF	complement(1207.. 1208)	153
21	complement (8442938..8442939)	HPV16REF	1894..1895	84
21	complement (8213982..8213983)	HPV16REF	3121..3122	68
21	complement (8214008..8214009)	HPV16REF	2870..2871	89
21	complement (8214309..8214310)	HPV16REF	6344..6345	67
21	complement (8214320..8214321)	HPV16REF	1413..1414	270
21	complement (8215209..8215210)	HPV16REF	5472..5473	138
21	complement (8215211..8215212)	HPV16REF	complement(795..796)	428
21	complement (8215220..8215221)	HPV16REF	2994..2995	57
21	complement (8215237..8215238)	HPV16REF	complement(7729.. 7730)	120
21	complement (8215244..8215245)	HPV16REF	2450..2451	273
21	complement (8215254..8215255)	HPV16REF	4002..4003	113
21	complement (8215255..8215256)	HPV16REF	complement(1207.. 1208)	71
21	complement (8215274..8215275)	HPV16REF	6550..6551	53
21	complement (8215280..8215281)	HPV16REF	1767..1768	146
21	complement (8215375..8215376)	HPV16REF	6688..6689	80
21	complement (8215380..8215381)	HPV16REF	6214..6215	106
21	complement (8215400..8215401)	HPV16REF	2722..2723	139
21	complement (8215401..8215402)	HPV16REF	1374..1375	150

30927-005.R1 (paired) (Report)

Host chromosome	Host region	Virus	Virus region	Unaligned ends host
21	complement (8215402..8215403)	HPV16REF	6133..6134	157
21	complement (8215409..8215410)	HPV16REF	2357..2358	421
21	complement (8215705..8215706)	HPV16REF	1650..1651	250
21	complement (8216407..8216408)	HPV16REF	602..603	288
21	complement (8218522..8218523)	HPV16REF	1374..1375	190
21	complement (8218549..8218550)	HPV16REF	complement(406..407)	818
21	complement (8218572..8218573)	HPV16REF	complement(2059.. 2060)	1,012
21	complement (8218578..8218579)	HPV16REF	1916..1917	66
21	complement (8218580..8218581)	HPV16REF	3660..3661	135
21	complement (8218590..8218591)	HPV16REF	complement(2059.. 2060)	85
21	complement (8218591..8218592)	HPV16REF	complement(7684.. 7685)	74
21	complement (8218592..8218593)	HPV16REF	complement(524..525)	333
21	complement (8218607..8218608)	HPV16REF	complement(3377.. 3378)	172
21	complement (8218608..8218609)	HPV16REF	complement(524..525)	161
21	complement (8218610..8218611)	HPV16REF	5793..5794	563
21	complement (8218617..8218618)	HPV16REF	3149..3150	120
21	complement (8218626..8218627)	HPV16REF	6157..6158	103
21	complement (8218627..8218628)	HPV16REF	1397..1398	96
21	complement (8218636..8218637)	HPV16REF	2824..2825	135
21	complement (8218637..8218638)	HPV16REF	6340..6341	145
21	complement (8218642..8218643)	HPV16REF	2915..2916	74
21	complement (8218652..8218653)	HPV16REF	6835..6836	79
21	complement (8218664..8218665)	HPV16REF	1085..1086	79

30927-005.R1 (paired) (Report)

Host chromosome	Host region	Virus	Virus region	Unaligned ends host
21	complement (8218686..8218687)	HPV16REF	7287..7288	308
21	complement (8443130..8443131)	HPV16REF	2854..2855	86
21	complement (8434055..8434056)	HPV16REF	1837..1838	118
21	complement (8437114..8437115)	HPV16REF	140..141	323
21	complement (8437178..8437179)	HPV16REF	6266..6267	53
21	complement (8438236..8438237)	HPV16REF	6376..6377	145
21	complement (8438298..8438299)	HPV16REF	1397..1398	112
21	complement (8438323..8438324)	HPV16REF	complement(7786.. 7787)	179
21	complement (8438345..8438346)	HPV16REF	5893..5894	144
21	complement (8441155..8441156)	HPV16REF	4362..4363	125
21	complement (8441165..8441166)	HPV16REF	1732..1733	76
21	complement (8441196..8441197)	HPV16REF	complement(524..525)	141
21	complement (8441198..8441199)	HPV16REF	1981..1982	105
21	complement (8441201..8441202)	HPV16REF	1392..1393	64
21	complement (8441234..8441235)	HPV16REF	5684..5685	150
21	complement (8441240..8441241)	HPV16REF	3237..3238	57
21	complement (8441565..8441566)	HPV16REF	45..46	128
21	complement (8441580..8441581)	HPV16REF	complement(1207.. 1208)	54
21	complement (8442091..8442092)	HPV16REF	complement(3377.. 3378)	86
21	complement (8442121..8442122)	HPV16REF	4349..4350	268
21	complement (8442152..8442153)	HPV16REF	6225..6226	76
21	complement (8442157..8442158)	HPV16REF	678..679	78
21	complement (8442191..8442192)	HPV16REF	5068..5069	92

30927-005.R1 (paired) (Report)

Host chromosome	Host region	Virus	Virus region	Unaligned ends host
21	complement (8442207..8442208)	HPV16REF	6385..6386	168
21	complement (8442214..8442215)	HPV16REF	2899..2900	177
21	complement (8442217..8442218)	HPV16REF	3675..3676	164
21	complement (8442223..8442224)	HPV16REF	6227..6228	343
21	complement (8442226..8442227)	HPV16REF	678..679	131
21	complement (8442236..8442237)	HPV16REF	5825..5826	82
21	complement (8442247..8442248)	HPV16REF	7313..7314	243
21	complement (8442252..8442253)	HPV16REF	1413..1414	272
21	complement (8442266..8442267)	HPV16REF	140..141	85
21	complement (8442310..8442311)	HPV16REF	7524..7525	90
21	complement (8442312..8442313)	HPV16REF	complement(2059..2060)	113
21	complement (8442313..8442314)	HPV16REF	7482..7483	130
21	complement (8442320..8442321)	HPV16REF	4346..4347	163
21	complement (8442323..8442324)	HPV16REF	2803..2804	208
21	complement (8442324..8442325)	HPV16REF	6688..6689	169
21	complement (8442331..8442332)	HPV16REF	2264..2265	82
21	complement (8442338..8442339)	HPV16REF	3133..3134	87
21	complement (8442343..8442344)	HPV16REF	complement(3449..3450)	119
21	complement (8442347..8442348)	HPV16REF	complement(3449..3450)	83
21	complement (8442470..8442471)	HPV16REF	complement(795..796)	70
21	complement (8442492..8442493)	HPV16REF	7426..7427	94
21	complement (8442494..8442495)	HPV16REF	1101..1102	141
21	complement (8442501..8442502)	HPV16REF	934..935	65

30927-005.R1 (paired) (Report)

Host chromosome	Host region	Virus	Virus region	Unaligned ends host
21	complement (8442507..8442508)	HPV16REF	226..227	315
21	complement (8442525..8442526)	HPV16REF	2802..2803	748
21	complement (8442527..8442528)	HPV16REF	2634..2635	122
21	complement (8442573..8442574)	HPV16REF	4366..4367	70
21	complement (8442578..8442579)	HPV16REF	complement(3290.. 3291)	63
21	complement (8442609..8442610)	HPV16REF	1374..1375	92
21	complement (8442620..8442621)	HPV16REF	complement(3449.. 3450)	155
21	complement (8442628..8442629)	HPV16REF	1676..1677	122
21	complement (8442648..8442649)	HPV16REF	complement(1207.. 1208)	244
21	complement (8442672..8442673)	HPV16REF	4525..4526	453
21	complement (8442737..8442738)	HPV16REF	6688..6689	83
21	complement (8442968..8442969)	HPV16REF	3658..3659	265
21	complement (8443118..8443119)	HPV16REF	3719..3720	250
21	complement (8443120..8443121)	HPV16REF	complement(5268.. 5269)	222
21	complement (8443122..8443123)	HPV16REF	8046..8047	231
21	complement (8443149..8443150)	HPV16REF	3614..3615	64
21	complement (8443150..8443151)	HPV16REF	2723..2724	115
21	complement (8443175..8443176)	HPV16REF	1793..1794	90
21	complement (8443205..8443206)	HPV16REF	7333..7334	199
21	complement (8443206..8443207)	HPV16REF	6316..6317	137
21	complement (8443208..8443209)	HPV16REF	5552..5553	112
21	complement (8443229..8443230)	HPV16REF	3515..3516	89
21	complement (8443240..8443241)	HPV16REF	2935..2936	202

30927-005.R1 (paired) (Report)

Host chromosome	Host region	Virus	Virus region	Unaligned ends host
21	complement (8443241..8443242)	HPV16REF	3144..3145	142
21	complement (8443246..8443247)	HPV16REF	5864..5865	331
21	complement (8443291..8443292)	HPV16REF	2562..2563	80
21	complement (8443587..8443588)	HPV16REF	complement(406..407)	67
21	complement (8443598..8443599)	HPV16REF	2239..2240	83
21	complement (8443676..8443677)	HPV16REF	5464..5465	322
21	complement (8443725..8443726)	HPV16REF	complement(7684.. 7685)	77
21	complement (8443970..8443971)	HPV16REF	5949..5950	97
21	complement (8445808..8445809)	HPV16REF	3016..3017	77
21	complement (8445819..8445820)	HPV16REF	complement(5268.. 5269)	798
21	complement (8445825..8445826)	HPV16REF	complement(3449.. 3450)	156
21	complement (8445826..8445827)	HPV16REF	3610..3611	53
21	complement (8445842..8445843)	HPV16REF	complement(7729.. 7730)	990
21	complement (8445856..8445857)	HPV16REF	7155..7156	65
21	complement (8445875..8445876)	HPV16REF	7425..7426	231
21	complement (8445877..8445878)	HPV16REF	complement(3449.. 3450)	130
21	complement (8445897..8445898)	HPV16REF	1413..1414	99
21	complement (8445912..8445913)	HPV16REF	2915..2916	78
21	complement (8445913..8445914)	HPV16REF	2124..2125	56
21	complement (8445917..8445918)	HPV16REF	complement(5268.. 5269)	224
21	complement (8445919..8445920)	HPV16REF	4190..4191	73
21	complement (8445956..8445957)	HPV16REF	7285..7286	305
21	complement (8445988..8445989)	HPV16REF	7620..7621	154

30927-005.R1 (paired) (Report)

Host chromosome	Host region	Virus	Virus region	Unaligned ends host
21	complement (8445995..8445996)	HPV16REF	7489..7490	79
21	8259640..8259641	HPV16REF	7536..7537	274
21	8259695..8259696	HPV16REF	2737..2738	325
21	8259705..8259706	HPV16REF	complement(406..407)	235
21	8260079..8260080	HPV16REF	complement(3787.. 3788)	277
21	8398445..8398446	HPV16REF	1466..1467	290
21	8398500..8398501	HPV16REF	6545..6546	403
21	8398660..8398661	HPV16REF	6338..6339	209
21	8398884..8398885	HPV16REF	2738..2739	287
21	8399510..8399511	HPV16REF	5540..5541	205
21	8399660..8399661	HPV16REF	678..679	235
21	8211278..8211279	HPV16REF	4016..4017	458
21	8214470..8214471	HPV16REF	678..679	202
21	8215463..8215464	HPV16REF	complement(2059.. 2060)	215
21	8215466..8215467	HPV16REF	5442..5443	348
21	8215850..8215851	HPV16REF	2578..2579	323
21	8218802..8218803	HPV16REF	complement(7743.. 7744)	223
21	8218809..8218810	HPV16REF	complement(7743.. 7744)	320
21	8218811..8218812	HPV16REF	complement(7729.. 7730)	198
21	8218814..8218815	HPV16REF	1853..1854	278
21	8218820..8218821	HPV16REF	3607..3608	301
21	8218851..8218852	HPV16REF	591..592	217
21	8218871..8218872	HPV16REF	2914..2915	202
21	8438523..8438524	HPV16REF	3693..3694	477
21	8442397..8442398	HPV16REF	678..679	333
21	8442455..8442456	HPV16REF	302..303	424
21	8442456..8442457	HPV16REF	3987..3988	243
21	8442457..8442458	HPV16REF	3515..3516	549
21	8442458..8442459	HPV16REF	3862..3863	594
21	8442460..8442461	HPV16REF	complement(795..796)	797
21	8442459..8442460	HPV16REF	678..679	590
21	8442461..8442462	HPV16REF	6120..6121	613
21	8442674..8442675	HPV16REF	6150..6151	302
21	8442726..8442727	HPV16REF	complement(3449.. 3450)	230
21	8442739..8442740	HPV16REF	2288..2289	230

30927-005.R1 (paired) (Report)

Host chromosome	Host region	Virus	Virus region	Unaligned ends host
21	8443326..8443327	HPV16REF	1541..1542	260
21	8443396..8443397	HPV16REF	678..679	277
21	8446081..8446082	HPV16REF	complement(7729.. 7730)	230
21	8446090..8446091	HPV16REF	2241..2242	316
21	8446092..8446093	HPV16REF	1466..1467	212
22	21695712..21695713	HPV16REF	6451..6452	50
22	21695747..21695748	HPV16REF	complement(3290.. 3291)	52
22	21695815..21695816	HPV16REF	2201..2202	28
22	21695836..21695837	HPV16REF	3732..3733	27
X	complement (86643984..86643985)	HPV16REF	7440..7441	283
X	86643963..86643964	HPV16REF	2635..2636	195

Host chromosome	Unaligned ends virus	Broken reads host	Disrupted genes	Nearby genes
2	N/A	304	LINC00486	TTC27 (-95508), LINC00486, Y_RNA (+28779), LTBP1 (+30393)
2	2,822	376	LINC00486	TTC27 (-95574), LINC00486, Y_RNA (+28713), LTBP1 (+30327)
3	N/A	1	ANKRD28, RN7SL4P	BTD (-16254), ANKRD28, MIR3134 (-41194), gene: ENSG00000279465 (-6029), RN7SL4P
3	1,930	1	ANKRD28, RN7SL4P	BTD (-16380), ANKRD28, MIR3134 (-41320), gene: ENSG00000279465 (-6155), RN7SL4P
4	3,510	66		gene: ENSG00000286046 (+88192)
4	N/A	56		gene: ENSG00000286046 (+88204)
4	N/A	56		IGFBP7 (-99403), IGFBP7-AS1 (-2329), gene: ENSG00000287369 (-98388), gene: ENSG00000250192 (-53336)

30927-005.R1 (paired) (Report)

Host chromosome	Unaligned ends virus	Broken reads host	Disrupted genes	Nearby genes
6	N/A	1	ZBTB22	gene: ENSG00000272217 (-68973), HCG25 (-60662), VPS52 (-43782), RPS18 (-39318), B3GALT4 (-30997), WDR46 (-26582), MIR6873 (-28540), PFDN6 (-17428), MIR6834 (-25504), RGL2 (-16887), TAPBP (-1442), ZBTB22, DAXX (+2728), gene: ENSG00000285064 (+5556), SMIM40 (+7798), KIFC1 (+75993), PHF1 (+94569)
6	N/A	1	ZBTB22	gene: ENSG00000272217 (-68975), HCG25 (-60664), VPS52 (-43784), RPS18 (-39320), B3GALT4 (-30999), WDR46 (-26584), MIR6873 (-28542), PFDN6 (-17430), MIR6834 (-25506), RGL2 (-16889), TAPBP (-1444), ZBTB22, DAXX (+2726), gene: ENSG00000285064 (+5554), SMIM40 (+7796), KIFC1 (+75991), PHF1 (+94567)

30927-005.R1 (paired) (Report)

Host chromosome	Unaligned ends virus	Broken reads host	Disrupted genes	Nearby genes
6	N/A	1	ZBTB22	gene: ENSG00000272217 (-69224), HCG25 (-60913), VPS52 (-44033), RPS18 (-39569), B3GALT4 (-31248), WDR46 (-26833), MIR6873 (-28791), PFDN6 (-17679), MIR6834 (-25755), RGL2 (-17138), TAPBP (-1693), ZBTB22, DAXX (+2477), gene: ENSG00000285064 (+5305), SMIM40 (+7547), KIFC1 (+75742), PHF1 (+94318)
6	N/A	1	ZBTB22	gene: ENSG00000272217 (-69574), HCG25 (-61263), VPS52 (-44383), RPS18 (-39919), B3GALT4 (-31598), WDR46 (-27183), MIR6873 (-29141), PFDN6 (-18029), MIR6834 (-26105), RGL2 (-17488), TAPBP (-2043), ZBTB22, DAXX (+2127), gene: ENSG00000285064 (+4955), SMIM40 (+7197), KIFC1 (+75392), PHF1 (+93968)

30927-005.R1 (paired) (Report)

Host chromosome	Unaligned ends virus	Broken reads host	Disrupted genes	Nearby genes
6	3,426	1	ZBTB22	gene: ENSG00000272217 (-69543), HCG25 (-61232), VPS52 (-44352), RPS18 (-39888), B3GALT4 (-31567), WDR46 (-27152), MIR6873 (-29110), PFDN6 (-17998), MIR6834 (-26074), RGL2 (-17457), TAPBP (-2012), ZBTB22, DAXX (+2158), gene: ENSG00000285064 (+4986), SMIM40 (+7228), KIFC1 (+75423), PHF1 (+93999)
7	N/A	70	GNGT1	GNGT1, TFPI2 (-14772), TFPI2-DT (-11924), GNG11 (+16209), gene: ENSG00000278959 (+48518), BET1 (+57236), BET1-AS1 (+63916)
7	N/A	121	GNGT1	GNGT1, TFPI2 (-11800), TFPI2-DT (-8952), GNG11 (+19181), gene: ENSG00000278959 (+51490), BET1 (+60208), BET1-AS1 (+66888)
8	5,325	7	PLAT	gene: ENSG00000271938 (-67741), gene: ENSG00000261449 (-54730), AP3M2 (-35820), PLAT, IKBKB-DT (+26180), IKBKB (+63808)
8	3,951	33		gene: ENSG00000271938 (-76621), gene: ENSG00000261449 (-63610), AP3M2 (-44700), PLAT (-8664), IKBKB-DT (+17300), IKBKB (+54928)

30927-005.R1 (paired) (Report)

Host chromosome	Unaligned ends virus	Broken reads host	Disrupted genes	Nearby genes
8	5,325	5		gene: ENSG00000271938 (-76719), gene: ENSG00000261449 (-63708), AP3M2 (-44798), PLAT (-8762), IKBKB-DT (+17202), IKBKB (+54830)
8	3,276	1	HGH1	SPATC1 (-92988), OPLAH (-76137), MIR6846 (-82722), gene: ENSG00000255224 (-60837), EXOSC4 (-59454), MIR6847 (-60160), GPAA1 (-53886), CYC1 (-42577), SHARPIN (-31978), MAF1 (-32491), WDR97 (-21774), HGH1, MROH1 (+7913)
8	4,496	87		gene: ENSG00000271938 (-76593), gene: ENSG00000261449 (-63582), AP3M2 (-44672), PLAT (-8636), IKBKB-DT (+17328), IKBKB (+54956)
8	92	90	PLAT	gene: ENSG00000271938 (-49321), gene: ENSG00000261449 (-36310), AP3M2 (-17400), PLAT, IKBKB-DT (+44600), IKBKB (+82228)
8	590	23	PLAT	gene: ENSG00000271938 (-53461), gene: ENSG00000261449 (-40450), AP3M2 (-21540), PLAT, IKBKB-DT (+40460), IKBKB (+78088)
8	11,265	23	PLAT	gene: ENSG00000271938 (-53462), gene: ENSG00000261449 (-40451), AP3M2 (-21541), PLAT, IKBKB-DT (+40459), IKBKB (+78087)

30927-005.R1 (paired) (Report)

Host chromosome	Unaligned ends virus	Broken reads host	Disrupted genes	Nearby genes
8	N/A	1	HGH1	SPATC1 (-92977), OPLAH (-76126), MIR6846 (-82711), gene: ENSG00000255224 (-60826), EXOSC4 (-59443), MIR6847 (-60149), GPAA1 (-53875), CYC1 (-42566), SHARPIN (-31967), MAF1 (-32480), WDR97 (-21763), HGH1, MROH1 (+7924)
8	N/A	1	HGH1	SPATC1 (-93001), OPLAH (-76150), MIR6846 (-82735), gene: ENSG00000255224 (-60850), EXOSC4 (-59467), MIR6847 (-60173), GPAA1 (-53899), CYC1 (-42590), SHARPIN (-31991), MAF1 (-32504), WDR97 (-21787), HGH1, MROH1 (+7900)
8	6,188	1	HGH1	SPATC1 (-93003), OPLAH (-76152), MIR6846 (-82737), gene: ENSG00000255224 (-60852), EXOSC4 (-59469), MIR6847 (-60175), GPAA1 (-53901), CYC1 (-42592), SHARPIN (-31993), MAF1 (-32506), WDR97 (-21789), HGH1, MROH1 (+7898)

30927-005.R1 (paired) (Report)

Host chromosome	Unaligned ends virus	Broken reads host	Disrupted genes	Nearby genes
8	3,426	1	HGH1	SPATC1 (-93035), OPLAH (-76184), MIR6846 (-82769), gene: ENSG00000255224 (-60884), EXOSC4 (-59501), MIR6847 (-60207), GPAA1 (-53933), CYC1 (-42624), SHARPIN (-32025), MAF1 (-32538), WDR97 (-21821), HGH1, MROH1 (+7866)
8	N/A	2	HGH1	SPATC1 (-93061), OPLAH (-76210), MIR6846 (-82795), gene: ENSG00000255224 (-60910), EXOSC4 (-59527), MIR6847 (-60233), GPAA1 (-53959), CYC1 (-42650), SHARPIN (-32051), MAF1 (-32564), WDR97 (-21847), HGH1, MROH1 (+7840)
8	N/A	2	HGH1	SPATC1 (-93069), OPLAH (-76218), MIR6846 (-82803), gene: ENSG00000255224 (-60918), EXOSC4 (-59535), MIR6847 (-60241), GPAA1 (-53967), CYC1 (-42658), SHARPIN (-32059), MAF1 (-32572), WDR97 (-21855), HGH1, MROH1 (+7832)

30927-005.R1 (paired) (Report)

Host chromosome	Unaligned ends virus	Broken reads host	Disrupted genes	Nearby genes
8	N/A	2	HGH1	SPATC1 (-93074), OPLAH (-76223), MIR6846 (-82808), gene: ENSG00000255224 (-60923), EXOSC4 (-59540), MIR6847 (-60246), GPAA1 (-53972), CYC1 (-42663), SHARPIN (-32064), MAF1 (-32577), WDR97 (-21860), HGH1, MROH1 (+7827)
8	61,253	2	HGH1	SPATC1 (-93087), OPLAH (-76236), MIR6846 (-82821), gene: ENSG00000255224 (-60936), EXOSC4 (-59553), MIR6847 (-60259), GPAA1 (-53985), CYC1 (-42676), SHARPIN (-32077), MAF1 (-32590), WDR97 (-21873), HGH1, MROH1 (+7814)
8	3,489	2	HGH1	SPATC1 (-93097), OPLAH (-76246), MIR6846 (-82831), gene: ENSG00000255224 (-60946), EXOSC4 (-59563), MIR6847 (-60269), GPAA1 (-53995), CYC1 (-42686), SHARPIN (-32087), MAF1 (-32600), WDR97 (-21883), HGH1, MROH1 (+7804)

30927-005.R1 (paired) (Report)

Host chromosome	Unaligned ends virus	Broken reads host	Disrupted genes	Nearby genes
8	3,426	2	HGH1	SPATC1 (-93103), OPLAH (-76252), MIR6846 (-82837), gene: ENSG00000255224 (-60952), EXOSC4 (-59569), MIR6847 (-60275), GPAA1 (-54001), CYC1 (-42692), SHARPIN (-32093), MAF1 (-32606), WDR97 (-21889), HGH1, MROH1 (+7798)
8	N/A	2	HGH1	SPATC1 (-93134), OPLAH (-76283), MIR6846 (-82868), gene: ENSG00000255224 (-60983), EXOSC4 (-59600), MIR6847 (-60306), GPAA1 (-54032), CYC1 (-42723), SHARPIN (-32124), MAF1 (-32637), WDR97 (-21920), HGH1, MROH1 (+7767)
8	N/A	2	HGH1	SPATC1 (-93135), OPLAH (-76284), MIR6846 (-82869), gene: ENSG00000255224 (-60984), EXOSC4 (-59601), MIR6847 (-60307), GPAA1 (-54033), CYC1 (-42724), SHARPIN (-32125), MAF1 (-32638), WDR97 (-21921), HGH1, MROH1 (+7766)
9	4,505	4	PTPRD, RN7SL5P	PTPRD, RN7SL5P
9	N/A	4	PTPRD, RN7SL5P	PTPRD, RN7SL5P
9	N/A	2	PTPRD, RN7SL5P	PTPRD, RN7SL5P
9	60,930	2	PTPRD, RN7SL5P	PTPRD, RN7SL5P
9	61,253	5	PTPRD, RN7SL5P	PTPRD, RN7SL5P
9	N/A	6	PTPRD, RN7SL5P	PTPRD, RN7SL5P
9	N/A	6	PTPRD, RN7SL5P	PTPRD, RN7SL5P

30927-005.R1 (paired) (Report)

Host chromosome	Unaligned ends virus	Broken reads host	Disrupted genes	Nearby genes
9	3,551	1	RMRP	RUSC2 (-96039), FAM166B (-94038), gene: ENSG00000288586 (-52798), TESK1 (-47896), MIR4667 (-49778), CD72 (-11127), gene: ENSG00000231393 (-14420), gene: ENSG00000227933 (-10838), SIT1 (-6987), RMRP, CCDC107 (+352), ARHGEF39 (+937), RN7SL22P (+13838), CA9 (+15980), TPM2 (+24054), TLN1 (+39010), MIR6852 (+52738), CREB3 (+74660), MIR6853 (+74984), GBA2 (+78928), RGP1 (+91349), MSMP (+95052), gene: ENSG00000228843 (+98774)
9	N/A	6	PTPRD, RN7SL5P	PTPRD, RN7SL5P
10	N/A	59		KNDC1 (-25073), UTF1 (-19927), VENTX (-9557), MIR202HG (-3594), MIR202 (-3865), gene: ENSG00000273980 (+5658), ADAM8 (+10934), TUBGCP2 (+27149), ZNF511 (+57428), ZNF511-PRAP1 (+57924), CALY (+72586), gene: ENSG00000226699 (+94268), PRAP1 (+95882)

30927-005.R1 (paired) (Report)

Host chromosome	Unaligned ends virus	Broken reads host	Disrupted genes	Nearby genes
10	N/A	41	TUBGCP2	KNDC1 (-58500), UTF1 (-53354), VENTX (-42984), MIR202HG (-37021), MIR202 (-37292), gene: ENSG00000273980 (-27361), ADAM8 (-8044), TUBGCP2, ZNF511 (+24001), ZNF511-PRAP1 (+24497), CALY (+39159), gene: ENSG00000226699 (+60841), PRAP1 (+62455), FUOM (+70245), ECHS1 (+77572), MIR3944 (+86643), gene: ENSG00000274685 (+89823), PAOX (+94348), gene: ENSG00000254536 (+95104)
10	N/A	64	RGS10	GRK5 (-66223), RGS10, TIAL1 (+45833)
10	8,627	144	RGS10	GRK5 (-67559), RGS10, TIAL1 (+44497)
10	2,822	50		KNDC1 (-24709), UTF1 (-19563), VENTX (-9193), MIR202HG (-3230), MIR202 (-3501), gene: ENSG00000273980 (+6022), ADAM8 (+11298), TUBGCP2 (+27513), ZNF511 (+57792), ZNF511-PRAP1 (+58288), CALY (+72950), gene: ENSG00000226699 (+94632), PRAP1 (+96246)
10	N/A	140	RGS10	GRK5 (-66158), RGS10, TIAL1 (+45898)
10	4,442	144	RGS10	GRK5 (-67682), RGS10, TIAL1 (+44374)

30927-005.R1 (paired) (Report)

Host chromosome	Unaligned ends virus	Broken reads host	Disrupted genes	Nearby genes
10	N/A	77	TUBGCP2	KNDC1 (-71256), UTF1 (-66110), VENTX (-55740), MIR202HG (-49777), MIR202 (-50048), gene: ENSG00000273980 (-40117), ADAM8 (-20800), TUBGCP2, ZNF511 (+11245), ZNF511-PRAP1 (+11741), CALY (+26403), gene: ENSG00000226699 (+48085), PRAP1 (+49699), FUOM (+57489), ECHS1 (+64816), MIR3944 (+73887), gene: ENSG00000274685 (+77067), PAOX (+81592), gene: ENSG00000254536 (+82348), MTG1 (+96425)
14	N/A	11	gene: ENSG00000259001, RPPH1	OR11H4 (-98740), TTC5 (-37129), gene: ENSG00000258768 (-78781), gene: ENSG00000258459 (-36278), CCNB1IP1 (-9777), gene: ENSG00000287735 (-23382), SNORA79B (-19763), SNORD126 (-16563), gene: ENSG00000259001, RPPH1, PARP2 (+525), gene: ENSG00000254846 (+1143), TEP1 (+22577), KLHL33 (+82762)
14	N/A	2	gene: ENSG00000282885, RN7SL2	KLHDC2 (-76255), NEMF (-9819), Y_RNA (-23147), RNU6-539P (-22813), RN7SL3 (-8726), gene: ENSG00000282885, RN7SL2, gene: ENSG00000278002 (+431), ARF6 (+30441), RNU6-189P (+39209), Metazoa_SRP (+50852), LINC01588 (+64930)

30927-005.R1 (paired) (Report)

Host chromosome	Unaligned ends virus	Broken reads host	Disrupted genes	Nearby genes
14	N/A	64	RPS29, RN7SL1	RPS29, RN7SL1, Y_RNA (+5656), LRR1 (+12181), RPL36AL (+31950), MGAT2 (+34219), gene: ENSG00000258377 (+34235), DNAAF2 (+38594), gene: ENSG00000258450 (+42711), POLE2 (+56975), RNU6ATAC30P (+97964)
14	3,001	52	RPS29, RN7SL1	RPS29, RN7SL1, Y_RNA (+5637), LRR1 (+12162), RPL36AL (+31931), MGAT2 (+34200), gene: ENSG00000258377 (+34216), DNAAF2 (+38575), gene: ENSG00000258450 (+42692), POLE2 (+56956), RNU6ATAC30P (+97945)
14	4,505	51	RPS29, RN7SL1	RPS29, RN7SL1, Y_RNA (+5635), LRR1 (+12160), RPL36AL (+31929), MGAT2 (+34198), gene: ENSG00000258377 (+34214), DNAAF2 (+38573), gene: ENSG00000258450 (+42690), POLE2 (+56954), RNU6ATAC30P (+97943)
14	N/A	38	RPS29, RN7SL1	RPS29, RN7SL1, Y_RNA (+5618), LRR1 (+12143), RPL36AL (+31912), MGAT2 (+34181), gene: ENSG00000258377 (+34197), DNAAF2 (+38556), gene: ENSG00000258450 (+42673), POLE2 (+56937), RNU6ATAC30P (+97926)

30927-005.R1 (paired) (Report)

Host chromosome	Unaligned ends virus	Broken reads host	Disrupted genes	Nearby genes
14	N/A	38	RPS29, RN7SL1	RPS29, RN7SL1, Y_RNA (+5613), LRR1 (+12138), RPL36AL (+31907), MGAT2 (+34176), gene: ENSG00000258377 (+34192), DNAAF2 (+38551), gene: ENSG00000258450 (+42668), POLE2 (+56932), RNU6ATAC30P (+97921)
14	N/A	35	RPS29, RN7SL1	RPS29, RN7SL1, Y_RNA (+5610), LRR1 (+12135), RPL36AL (+31904), MGAT2 (+34173), gene: ENSG00000258377 (+34189), DNAAF2 (+38548), gene: ENSG00000258450 (+42665), POLE2 (+56929), RNU6ATAC30P (+97918)
14	4,436	32	RPS29, RN7SL1	RPS29, RN7SL1, Y_RNA (+5601), LRR1 (+12126), RPL36AL (+31895), MGAT2 (+34164), gene: ENSG00000258377 (+34180), DNAAF2 (+38539), gene: ENSG00000258450 (+42656), POLE2 (+56920), RNU6ATAC30P (+97909)
14	3,489	32	RPS29, RN7SL1	RPS29, RN7SL1, Y_RNA (+5600), LRR1 (+12125), RPL36AL (+31894), MGAT2 (+34163), gene: ENSG00000258377 (+34179), DNAAF2 (+38538), gene: ENSG00000258450 (+42655), POLE2 (+56919), RNU6ATAC30P (+97908)

30927-005.R1 (paired) (Report)

Host chromosome	Unaligned ends virus	Broken reads host	Disrupted genes	Nearby genes
14	N/A	31	RPS29, RN7SL1	RPS29, RN7SL1, Y_RNA (+5599), LRR1 (+12124), RPL36AL (+31893), MGAT2 (+34162), gene: ENSG00000258377 (+34178), DNAAF2 (+38537), gene: ENSG00000258450 (+42654), POLE2 (+56918), RNU6ATAC30P (+97907)
14	N/A	20	RPS29, RN7SL1	RPS29, RN7SL1, Y_RNA (+5588), LRR1 (+12113), RPL36AL (+31882), MGAT2 (+34151), gene: ENSG00000258377 (+34167), DNAAF2 (+38526), gene: ENSG00000258450 (+42643), POLE2 (+56907), RNU6ATAC30P (+97896)
14	3,481	20	RPS29, RN7SL1	RPS29, RN7SL1, Y_RNA (+5586), LRR1 (+12111), RPL36AL (+31880), MGAT2 (+34149), gene: ENSG00000258377 (+34165), DNAAF2 (+38524), gene: ENSG00000258450 (+42641), POLE2 (+56905), RNU6ATAC30P (+97894)
14	N/A	15	RPS29, RN7SL1	RPS29, RN7SL1, Y_RNA (+5568), LRR1 (+12093), RPL36AL (+31862), MGAT2 (+34131), gene: ENSG00000258377 (+34147), DNAAF2 (+38506), gene: ENSG00000258450 (+42623), POLE2 (+56887), RNU6ATAC30P (+97876)

30927-005.R1 (paired) (Report)

Host chromosome	Unaligned ends virus	Broken reads host	Disrupted genes	Nearby genes
14	3,551	14	RPS29, RN7SL1	RPS29, RN7SL1, Y_RNA (+5560), LRR1 (+12085), RPL36AL (+31854), MGAT2 (+34123), gene: ENSG00000258377 (+34139), DNAAF2 (+38498), gene: ENSG00000258450 (+42615), POLE2 (+56879), RNU6ATAC30P (+97868)
14	N/A	3	RPS29, RN7SL1	RPS29, RN7SL1, Y_RNA (+5523), LRR1 (+12048), RPL36AL (+31817), MGAT2 (+34086), gene: ENSG00000258377 (+34102), DNAAF2 (+38461), gene: ENSG00000258450 (+42578), POLE2 (+56842), RNU6ATAC30P (+97831)
14	6,188	9	RPS29, RN7SL1	RPS29, RN7SL1, Y_RNA (+5547), LRR1 (+12072), RPL36AL (+31841), MGAT2 (+34110), gene: ENSG00000258377 (+34126), DNAAF2 (+38485), gene: ENSG00000258450 (+42602), POLE2 (+56866), RNU6ATAC30P (+97855)
14	N/A	9	gene: ENSG00000282885, RN7SL2	KLHDC2 (-76200), NEMF (-9764), Y_RNA (-23092), RNU6-539P (-22758), RN7SL3 (-8671), gene: ENSG00000282885, RN7SL2, gene: ENSG00000278002 (+486), ARF6 (+30496), RNU6-189P (+39264), Metazoa_SRP (+50907), LINC01588 (+64985)

30927-005.R1 (paired) (Report)

Host chromosome	Unaligned ends virus	Broken reads host	Disrupted genes	Nearby genes
14	N/A	52	RPS29, RN7SL1	RPS29, RN7SL1, Y_RNA (+5636), LRR1 (+12161), RPL36AL (+31930), MGAT2 (+34199), gene: ENSG00000258377 (+34215), DNAAF2 (+38574), gene: ENSG00000258450 (+42691), POLE2 (+56955), RNU6ATAC30P (+97944)
14	3,551	13	gene: ENSG00000282885, RN7SL2	KLHDC2 (-76183), NEMF (-9747), Y_RNA (-23075), RNU6-539P (-22741), RN7SL3 (-8654), gene: ENSG00000282885, RN7SL2, gene: ENSG00000278002 (+503), ARF6 (+30513), RNU6-189P (+39281), Metazoa_SRP (+50924), LINC01588 (+65002)
14	N/A	12	gene: ENSG00000282885, RN7SL2	KLHDC2 (-76186), NEMF (-9750), Y_RNA (-23078), RNU6-539P (-22744), RN7SL3 (-8657), gene: ENSG00000282885, RN7SL2, gene: ENSG00000278002 (+500), ARF6 (+30510), RNU6-189P (+39278), Metazoa_SRP (+50921), LINC01588 (+64999)
14	3,489	12	gene: ENSG00000282885, RN7SL2	KLHDC2 (-76188), NEMF (-9752), Y_RNA (-23080), RNU6-539P (-22746), RN7SL3 (-8659), gene: ENSG00000282885, RN7SL2, gene: ENSG00000278002 (+498), ARF6 (+30508), RNU6-189P (+39276), Metazoa_SRP (+50919), LINC01588 (+64997)

30927-005.R1 (paired) (Report)

Host chromosome	Unaligned ends virus	Broken reads host	Disrupted genes	Nearby genes
14	N/A	12	gene: ENSG00000282885, RN7SL2	KLHDC2 (-76189), NEMF (-9753), Y_RNA (-23081), RNU6-539P (-22747), RN7SL3 (-8660), gene: ENSG00000282885, RN7SL2, gene: ENSG00000278002 (+497), ARF6 (+30507), RNU6-189P (+39275), Metazoa_SRP (+50918), LINC01588 (+64996)
14	N/A	11	gene: ENSG00000282885, RN7SL2	KLHDC2 (-76192), NEMF (-9756), Y_RNA (-23084), RNU6-539P (-22750), RN7SL3 (-8663), gene: ENSG00000282885, RN7SL2, gene: ENSG00000278002 (+494), ARF6 (+30504), RNU6-189P (+39272), Metazoa_SRP (+50915), LINC01588 (+64993)
14	3,951	8	gene: ENSG00000282885, RN7SL2	KLHDC2 (-76205), NEMF (-9769), Y_RNA (-23097), RNU6-539P (-22763), RN7SL3 (-8676), gene: ENSG00000282885, RN7SL2, gene: ENSG00000278002 (+481), ARF6 (+30491), RNU6-189P (+39259), Metazoa_SRP (+50902), LINC01588 (+64980)

30927-005.R1 (paired) (Report)

Host chromosome	Unaligned ends virus	Broken reads host	Disrupted genes	Nearby genes
14	N/A	15	gene: ENSG00000259001	OR11H4 (-98723), TTC5 (-37112), gene: ENSG00000258768 (-78764), gene: ENSG00000258459 (-36261), CCNB1IP1 (-9760), gene: ENSG00000287735 (-23365), SNORA79B (-19746), SNORD126 (-16546), gene: ENSG00000259001, RPPH1 (+2), PARP2 (+542), gene: ENSG00000254846 (+1160), TEP1 (+22594), KLHL33 (+82779)
14	N/A	11	gene: ENSG00000259001, RPPH1	OR11H4 (-98739), TTC5 (-37128), gene: ENSG00000258768 (-78780), gene: ENSG00000258459 (-36277), CCNB1IP1 (-9776), gene: ENSG00000287735 (-23381), SNORA79B (-19762), SNORD126 (-16562), gene: ENSG00000259001, RPPH1, PARP2 (+526), gene: ENSG00000254846 (+1144), TEP1 (+22578), KLHL33 (+82763)
14	N/A	3	gene: ENSG00000259001, RPPH1	OR11H4 (-98811), TTC5 (-37200), gene: ENSG00000258768 (-78852), gene: ENSG00000258459 (-36349), CCNB1IP1 (-9848), gene: ENSG00000287735 (-23453), SNORA79B (-19834), SNORD126 (-16634), gene: ENSG00000259001, RPPH1, PARP2 (+454), gene: ENSG00000254846 (+1072), TEP1 (+22506), KLHL33 (+82691)

30927-005.R1 (paired) (Report)

Host chromosome	Unaligned ends virus	Broken reads host	Disrupted genes	Nearby genes
14	N/A	2	RPS29, RN7SL1	RPS29, RN7SL1, Y_RNA (+5506), LRR1 (+12031), RPL36AL (+31800), MGAT2 (+34069), gene: ENSG00000258377 (+34085), DNAAF2 (+38444), gene: ENSG00000258450 (+42561), POLE2 (+56825), RNU6ATAC30P (+97814)
14	N/A	2	RPS29, RN7SL1	RPS29, RN7SL1, Y_RNA (+5503), LRR1 (+12028), RPL36AL (+31797), MGAT2 (+34066), gene: ENSG00000258377 (+34082), DNAAF2 (+38441), gene: ENSG00000258450 (+42558), POLE2 (+56822), RNU6ATAC30P (+97811)
14	N/A	7	RPS29, RN7SL1	RPS29, RN7SL1, Y_RNA (+5491), LRR1 (+12016), RPL36AL (+31785), MGAT2 (+34054), gene: ENSG00000258377 (+34070), DNAAF2 (+38429), gene: ENSG00000258450 (+42546), POLE2 (+56810), RNU6ATAC30P (+97799)
14	5,000	12	RPS29, RN7SL1	RPS29, RN7SL1, Y_RNA (+5480), LRR1 (+12005), RPL36AL (+31774), MGAT2 (+34043), gene: ENSG00000258377 (+34059), DNAAF2 (+38418), gene: ENSG00000258450 (+42535), POLE2 (+56799), RNU6ATAC30P (+97788)

30927-005.R1 (paired) (Report)

Host chromosome	Unaligned ends virus	Broken reads host	Disrupted genes	Nearby genes
14	5,325	12	RPS29, RN7SL1	RPS29, RN7SL1, Y_RNA (+5479), LRR1 (+12004), RPL36AL (+31773), MGAT2 (+34042), gene: ENSG00000258377 (+34058), DNAAF2 (+38417), gene: ENSG00000258450 (+42534), POLE2 (+56798), RNU6ATAC30P (+97787)
14	2,822	12	RPS29, RN7SL1	RPS29, RN7SL1, Y_RNA (+5478), LRR1 (+12003), RPL36AL (+31772), MGAT2 (+34041), gene: ENSG00000258377 (+34057), DNAAF2 (+38416), gene: ENSG00000258450 (+42533), POLE2 (+56797), RNU6ATAC30P (+97786)
14	60,930	13	RPS29, RN7SL1	RPS29, RN7SL1, Y_RNA (+5477), LRR1 (+12002), RPL36AL (+31771), MGAT2 (+34040), gene: ENSG00000258377 (+34056), DNAAF2 (+38415), gene: ENSG00000258450 (+42532), POLE2 (+56796), RNU6ATAC30P (+97785)
14	N/A	14	RPS29, RN7SL1	RPS29, RN7SL1, Y_RNA (+5476), LRR1 (+12001), RPL36AL (+31770), MGAT2 (+34039), gene: ENSG00000258377 (+34055), DNAAF2 (+38414), gene: ENSG00000258450 (+42531), POLE2 (+56795), RNU6ATAC30P (+97784)

30927-005.R1 (paired) (Report)

Host chromosome	Unaligned ends virus	Broken reads host	Disrupted genes	Nearby genes
14	3,551	14	RPS29, RN7SL1	RPS29, RN7SL1, Y_RNA (+5475), LRR1 (+12000), RPL36AL (+31769), MGAT2 (+34038), gene: ENSG00000258377 (+34054), DNAAF2 (+38413), gene: ENSG00000258450 (+42530), POLE2 (+56794), RNU6ATAC30P (+97783)
14	3,156	15	RPS29, RN7SL1	RPS29, RN7SL1, Y_RNA (+5471), LRR1 (+11996), RPL36AL (+31765), MGAT2 (+34034), gene: ENSG00000258377 (+34050), DNAAF2 (+38409), gene: ENSG00000258450 (+42526), POLE2 (+56790), RNU6ATAC30P (+97779)
14	N/A	15	RPS29, RN7SL1	RPS29, RN7SL1, Y_RNA (+5468), LRR1 (+11993), RPL36AL (+31762), MGAT2 (+34031), gene: ENSG00000258377 (+34047), DNAAF2 (+38406), gene: ENSG00000258450 (+42523), POLE2 (+56787), RNU6ATAC30P (+97776)
14	3,001	24	RPS29, RN7SL1	RPS29, RN7SL1, Y_RNA (+5451), LRR1 (+11976), RPL36AL (+31745), MGAT2 (+34014), gene: ENSG00000258377 (+34030), DNAAF2 (+38389), gene: ENSG00000258450 (+42506), POLE2 (+56770), RNU6ATAC30P (+97759)

30927-005.R1 (paired) (Report)

Host chromosome	Unaligned ends virus	Broken reads host	Disrupted genes	Nearby genes
14	N/A	24	RPS29, RN7SL1	RPS29, RN7SL1, Y_RNA (+5450), LRR1 (+11975), RPL36AL (+31744), MGAT2 (+34013), gene: ENSG00000258377 (+34029), DNAAF2 (+38388), gene: ENSG00000258450 (+42505), POLE2 (+56769), RNU6ATAC30P (+97758)
14	N/A	28	RPS29, RN7SL1	RPS29, RN7SL1, Y_RNA (+5434), LRR1 (+11959), RPL36AL (+31728), MGAT2 (+33997), gene: ENSG00000258377 (+34013), DNAAF2 (+38372), gene: ENSG00000258450 (+42489), POLE2 (+56753), RNU6ATAC30P (+97742)
14	6,188	29	RPS29, RN7SL1	RPS29, RN7SL1, Y_RNA (+5418), LRR1 (+11943), RPL36AL (+31712), MGAT2 (+33981), gene: ENSG00000258377 (+33997), DNAAF2 (+38356), gene: ENSG00000258450 (+42473), POLE2 (+56737), RNU6ATAC30P (+97726)
14	4,126	37	RPS29, RN7SL1	RPS29, RN7SL1, Y_RNA (+5397), LRR1 (+11922), RPL36AL (+31691), MGAT2 (+33960), gene: ENSG00000258377 (+33976), DNAAF2 (+38335), gene: ENSG00000258450 (+42452), POLE2 (+56716), RNU6ATAC30P (+97705)

30927-005.R1 (paired) (Report)

Host chromosome	Unaligned ends virus	Broken reads host	Disrupted genes	Nearby genes
14	N/A	38	RPS29, RN7SL1	RPS29, RN7SL1, Y_RNA (+5396), LRR1 (+11921), RPL36AL (+31690), MGAT2 (+33959), gene: ENSG00000258377 (+33975), DNAAF2 (+38334), gene: ENSG00000258450 (+42451), POLE2 (+56715), RNU6ATAC30P (+97704)
14	5,000	38	RPS29, RN7SL1	RPS29, RN7SL1, Y_RNA (+5395), LRR1 (+11920), RPL36AL (+31689), MGAT2 (+33958), gene: ENSG00000258377 (+33974), DNAAF2 (+38333), gene: ENSG00000258450 (+42450), POLE2 (+56714), RNU6ATAC30P (+97703)
14	N/A	38	RPS29, RN7SL1	RPS29, RN7SL1, Y_RNA (+5392), LRR1 (+11917), RPL36AL (+31686), MGAT2 (+33955), gene: ENSG00000258377 (+33971), DNAAF2 (+38330), gene: ENSG00000258450 (+42447), POLE2 (+56711), RNU6ATAC30P (+97700)
14	4,442	52	RPS29, RN7SL1	RPS29, RN7SL1, Y_RNA (+5381), LRR1 (+11906), RPL36AL (+31675), MGAT2 (+33944), gene: ENSG00000258377 (+33960), DNAAF2 (+38319), gene: ENSG00000258450 (+42436), POLE2 (+56700), RNU6ATAC30P (+97689)

30927-005.R1 (paired) (Report)

Host chromosome	Unaligned ends virus	Broken reads host	Disrupted genes	Nearby genes
14	N/A	53	RPS29, RN7SL1	RPS29, RN7SL1, Y_RNA (+5380), LRR1 (+11905), RPL36AL (+31674), MGAT2 (+33943), gene: ENSG00000258377 (+33959), DNAAF2 (+38318), gene: ENSG00000258450 (+42435), POLE2 (+56699), RNU6ATAC30P (+97688)
14	N/A	53	RPS29, RN7SL1	RPS29, RN7SL1, Y_RNA (+5378), LRR1 (+11903), RPL36AL (+31672), MGAT2 (+33941), gene: ENSG00000258377 (+33957), DNAAF2 (+38316), gene: ENSG00000258450 (+42433), POLE2 (+56697), RNU6ATAC30P (+97686)
14	N/A	63	RPS29, RN7SL1	RPS29, RN7SL1, Y_RNA (+5367), LRR1 (+11892), RPL36AL (+31661), MGAT2 (+33930), gene: ENSG00000258377 (+33946), DNAAF2 (+38305), gene: ENSG00000258450 (+42422), POLE2 (+56686), RNU6ATAC30P (+97675)
14	3,156	63	RPS29, RN7SL1	RPS29, RN7SL1, Y_RNA (+5364), LRR1 (+11889), RPL36AL (+31658), MGAT2 (+33927), gene: ENSG00000258377 (+33943), DNAAF2 (+38302), gene: ENSG00000258450 (+42419), POLE2 (+56683), RNU6ATAC30P (+97672)

30927-005.R1 (paired) (Report)

Host chromosome	Unaligned ends virus	Broken reads host	Disrupted genes	Nearby genes
14	N/A	63	RPS29, RN7SL1	RPS29, RN7SL1, Y_RNA (+5363), LRR1 (+11888), RPL36AL (+31657), MGAT2 (+33926), gene: ENSG00000258377 (+33942), DNAAF2 (+38301), gene: ENSG00000258450 (+42418), POLE2 (+56682), RNU6ATAC30P (+97671)
14	53,392	6	gene: ENSG00000259001, RPPH1	OR11H4 (-98949), TTC5 (-37338), gene: ENSG00000258768 (-78990), gene: ENSG00000258459 (-36487), CCNB1IP1 (-9986), gene: ENSG00000287735 (-23591), SNORA79B (-19972), SNORD126 (-16772), gene: ENSG00000259001, RPPH1, PARP2 (+316), gene: ENSG00000254846 (+934), TEP1 (+22368), KLHL33 (+82553)
14	2,822	5	gene: ENSG00000282885, RN7SL2	KLHDC2 (-76353), NEMF (-9917), Y_RNA (-23245), RNU6-539P (-22911), RN7SL3 (-8824), gene: ENSG00000282885, RN7SL2, gene: ENSG00000278002 (+333), ARF6 (+30343), RNU6-189P (+39111), Metazoa_SRP (+50754), LINC01588 (+64832)

30927-005.R1 (paired) (Report)

Host chromosome	Unaligned ends virus	Broken reads host	Disrupted genes	Nearby genes
14	6,188	5	gene: ENSG00000282885, RN7SL2	KLHDC2 (-76355), NEMF (-9919), Y_RNA (-23247), RNU6-539P (-22913), RN7SL3 (-8826), gene: ENSG00000282885, RN7SL2, gene: ENSG00000278002 (+331), ARF6 (+30341), RNU6-189P (+39109), Metazoa_SRP (+50752), LINC01588 (+64830)
14	N/A	6	gene: ENSG00000282885, RN7SL2	KLHDC2 (-76374), NEMF (-9938), Y_RNA (-23266), RNU6-539P (-22932), RN7SL3 (-8845), gene: ENSG00000282885, RN7SL2, gene: ENSG00000278002 (+312), ARF6 (+30322), RNU6-189P (+39090), Metazoa_SRP (+50733), LINC01588 (+64811)
14	N/A	6	gene: ENSG00000282885, RN7SL2	KLHDC2 (-76376), NEMF (-9940), Y_RNA (-23268), RNU6-539P (-22934), RN7SL3 (-8847), gene: ENSG00000282885, RN7SL2, gene: ENSG00000278002 (+310), ARF6 (+30320), RNU6-189P (+39088), Metazoa_SRP (+50731), LINC01588 (+64809)
14	N/A	7	gene: ENSG00000282885, RN7SL2	KLHDC2 (-76398), NEMF (-9962), Y_RNA (-23290), RNU6-539P (-22956), RN7SL3 (-8869), gene: ENSG00000282885, RN7SL2, gene: ENSG00000278002 (+288), ARF6 (+30298), RNU6-189P (+39066), Metazoa_SRP (+50709), LINC01588 (+64787)

30927-005.R1 (paired) (Report)

Host chromosome	Unaligned ends virus	Broken reads host	Disrupted genes	Nearby genes
14	53,392	9	gene: ENSG00000282885, RN7SL2	KLHDC2 (-76415), NEMF (-9979), Y_RNA (-23307), RNU6-539P (-22973), RN7SL3 (-8886), gene: ENSG00000282885, RN7SL2, gene: ENSG00000278002 (+271), ARF6 (+30281), RNU6-189P (+39049), Metazoa_SRP (+50692), LINC01588 (+64770)
14	3,551	9	gene: ENSG00000282885, RN7SL2	KLHDC2 (-76419), NEMF (-9983), Y_RNA (-23311), RNU6-539P (-22977), RN7SL3 (-8890), gene: ENSG00000282885, RN7SL2, gene: ENSG00000278002 (+267), ARF6 (+30277), RNU6-189P (+39045), Metazoa_SRP (+50688), LINC01588 (+64766)
14	61,253	5	gene: ENSG00000259001, RPPH1	OR11H4 (-98943), TTC5 (-37332), gene: ENSG00000258768 (-78984), gene: ENSG00000258459 (-36481), CCNB1IP1 (-9980), gene: ENSG00000287735 (-23585), SNORA79B (-19966), SNORD126 (-16766), gene: ENSG00000259001, RPPH1, PARP2 (+322), gene: ENSG00000254846 (+940), TEP1 (+22374), KLHL33 (+82559)

30927-005.R1 (paired) (Report)

Host chromosome	Unaligned ends virus	Broken reads host	Disrupted genes	Nearby genes
14	4,505	18	RPS29, RN7SL1	RPS29, RN7SL1, Y_RNA (+5456), LRR1 (+11981), RPL36AL (+31750), MGAT2 (+34019), gene: ENSG00000258377 (+34035), DNAAF2 (+38394), gene: ENSG00000258450 (+42511), POLE2 (+56775), RNU6ATAC30P (+97764)
14	92	12	gene: ENSG00000259001, RPPH1	OR11H4 (-99038), TTC5 (-37427), gene: ENSG00000258768 (-79079), gene: ENSG00000258459 (-36576), CCNB1IP1 (-10075), gene: ENSG00000287735 (-23680), SNORA79B (-20061), SNORD126 (-16861), gene: ENSG00000259001, RPPH1, PARP2 (+227), gene: ENSG00000254846 (+845), TEP1 (+22279), KLHL33 (+82464)
14	N/A	6	gene: ENSG00000282885, RN7SL2	KLHDC2 (-76394), NEMF (-9958), Y_RNA (-23286), RNU6-539P (-22952), RN7SL3 (-8865), gene: ENSG00000282885, RN7SL2, gene: ENSG00000278002 (+292), ARF6 (+30302), RNU6-189P (+39070), Metazoa_SRP (+50713), LINC01588 (+64791)

30927-005.R1 (paired) (Report)

Host chromosome	Unaligned ends virus	Broken reads host	Disrupted genes	Nearby genes
16	N/A	3	METTL26	CAPN15 (-79799), MIR5587 (-99067), MIR3176 (-91069), gene: ENSG00000261691 (-80588), PRR35 (-68906), PIGQ (-50326), NHLRC4 (-64940), gene: ENSG00000282907 (-58178), RAB40C (-5163), WFIKKN1 (-318), METTL26, MCRIP2 (+2381), gene: ENSG00000228201 (+14037), WDR90 (+14875), gene: ENSG00000262528 (+20175), RHOT2 (+33669), RHBDL1 (+41235), LINC02867 (+44068), gene: ENSG00000279441 (+44209), STUB1 (+45788), JMJD8 (+47234), WDR24 (+50186), gene: ENSG00000261659 (+54565), FBXL16 (+58062), gene: ENSG00000279255 (+73214), gene: ENSG00000259840 (+76310), METRN (+80682), ANTKMT (+86145), CCDC78 (+88146), HAGHL (+92500), CIAO3 (+95324)

30927-005.R1 (paired) (Report)

Host chromosome	Unaligned ends virus	Broken reads host	Disrupted genes	Nearby genes
16	N/A	3	METTL26	CAPN15 (-79823), MIR5587 (-99091), MIR3176 (-91093), gene: ENSG00000261691 (-80612), PRR35 (-68930), PIGQ (-50350), NHLRC4 (-64964), gene: ENSG00000282907 (-58202), RAB40C (-5187), WFIKKN1 (-342), METTL26, MCRIP2 (+2357), gene: ENSG00000228201 (+14013), WDR90 (+14851), gene: ENSG00000262528 (+20151), RHOT2 (+33645), RHBDL1 (+41211), LINC02867 (+44044), gene: ENSG00000279441 (+44185), STUB1 (+45764), JMJD8 (+47210), WDR24 (+50162), gene: ENSG00000261659 (+54541), FBXL16 (+58038), gene: ENSG00000279255 (+73190), gene: ENSG00000259840 (+76286), METRN (+80658), ANTKMT (+86121), CCDC78 (+88122), HAGHL (+92476), CIAO3 (+95300)

30927-005.R1 (paired) (Report)

Host chromosome	Unaligned ends virus	Broken reads host	Disrupted genes	Nearby genes
16	N/A	3	METTL26	CAPN15 (-79828), MIR5587 (-99096), MIR3176 (-91098), gene: ENSG00000261691 (-80617), PRR35 (-68935), PIGQ (-50355), NHLRC4 (-64969), gene: ENSG00000282907 (-58207), RAB40C (-5192), WFIKKN1 (-347), METTL26, MCRIP2 (+2352), gene: ENSG00000228201 (+14008), WDR90 (+14846), gene: ENSG00000262528 (+20146), RHOT2 (+33640), RHBDL1 (+41206), LINC02867 (+44039), gene: ENSG00000279441 (+44180), STUB1 (+45759), JMJD8 (+47205), WDR24 (+50157), gene: ENSG00000261659 (+54536), FBXL16 (+58033), gene: ENSG00000279255 (+73185), gene: ENSG00000259840 (+76281), METRN (+80653), ANTKMT (+86116), CCDC78 (+88117), HAGHL (+92471), CIAO3 (+95295)

30927-005.R1 (paired) (Report)

Host chromosome	Unaligned ends virus	Broken reads host	Disrupted genes	Nearby genes
16	1,930	3	METTL26	CAPN15 (-79845), MIR5587 (-99113), MIR3176 (-91115), gene: ENSG00000261691 (-80634), PRR35 (-68952), PIGQ (-50372), NHLRC4 (-64986), gene: ENSG00000282907 (-58224), RAB40C (-5209), WFIKKN1 (-364), METTL26, MCRIP2 (+2335), gene: ENSG00000228201 (+13991), WDR90 (+14829), gene: ENSG00000262528 (+20129), RHOT2 (+33623), RHBDL1 (+41189), LINC02867 (+44022), gene: ENSG00000279441 (+44163), STUB1 (+45742), JMJD8 (+47188), WDR24 (+50140), gene: ENSG00000261659 (+54519), FBXL16 (+58016), gene: ENSG00000279255 (+73168), gene: ENSG00000259840 (+76264), METRN (+80636), ANTKMT (+86099), CCDC78 (+88100), HAGHL (+92454), CIAO3 (+95278)

30927-005.R1 (paired) (Report)

Host chromosome	Unaligned ends virus	Broken reads host	Disrupted genes	Nearby genes
16	N/A	3	METTL26	CAPN15 (-79851), MIR5587 (-99119), MIR3176 (-91121), gene: ENSG00000261691 (-80640), PRR35 (-68958), PIGQ (-50378), NHLRC4 (-64992), gene: ENSG00000282907 (-58230), RAB40C (-5215), WFIKKN1 (-370), METTL26, MCRIP2 (+2329), gene: ENSG00000228201 (+13985), WDR90 (+14823), gene: ENSG00000262528 (+20123), RHOT2 (+33617), RHBDL1 (+41183), LINC02867 (+44016), gene: ENSG00000279441 (+44157), STUB1 (+45736), JMJD8 (+47182), WDR24 (+50134), gene: ENSG00000261659 (+54513), FBXL16 (+58010), gene: ENSG00000279255 (+73162), gene: ENSG00000259840 (+76258), METRN (+80630), ANTKMT (+86093), CCDC78 (+88094), HAGHL (+92448), CIAO3 (+95272)

30927-005.R1 (paired) (Report)

Host chromosome	Unaligned ends virus	Broken reads host	Disrupted genes	Nearby genes
16	N/A	3	METTL26	CAPN15 (-79858), MIR5587 (-99126), MIR3176 (-91128), gene: ENSG00000261691 (-80647), PRR35 (-68965), PIGQ (-50385), NHLRC4 (-64999), gene: ENSG00000282907 (-58237), RAB40C (-5222), WFIKKN1 (-377), METTL26, MCRIP2 (+2322), gene: ENSG00000228201 (+13978), WDR90 (+14816), gene: ENSG00000262528 (+20116), RHOT2 (+33610), RHBDL1 (+41176), LINC02867 (+44009), gene: ENSG00000279441 (+44150), STUB1 (+45729), JMJD8 (+47175), WDR24 (+50127), gene: ENSG00000261659 (+54506), FBXL16 (+58003), gene: ENSG00000279255 (+73155), gene: ENSG00000259840 (+76251), METRN (+80623), ANTKMT (+86086), CCDC78 (+88087), HAGHL (+92441), CIAO3 (+95265)

30927-005.R1 (paired) (Report)

Host chromosome	Unaligned ends virus	Broken reads host	Disrupted genes	Nearby genes
16	N/A	3	METTL26	CAPN15 (-79861), MIR5587 (-99129), MIR3176 (-91131), gene: ENSG00000261691 (-80650), PRR35 (-68968), PIGQ (-50388), NHLRC4 (-65002), gene: ENSG00000282907 (-58240), RAB40C (-5225), WFIKKN1 (-380), METTL26, MCRIP2 (+2319), gene: ENSG00000228201 (+13975), WDR90 (+14813), gene: ENSG00000262528 (+20113), RHOT2 (+33607), RHBDL1 (+41173), LINC02867 (+44006), gene: ENSG00000279441 (+44147), STUB1 (+45726), JMJD8 (+47172), WDR24 (+50124), gene: ENSG00000261659 (+54503), FBXL16 (+58000), gene: ENSG00000279255 (+73152), gene: ENSG00000259840 (+76248), METRN (+80620), ANTKMT (+86083), CCDC78 (+88084), HAGHL (+92438), CIAO3 (+95262)

30927-005.R1 (paired) (Report)

Host chromosome	Unaligned ends virus	Broken reads host	Disrupted genes	Nearby genes
16	N/A	3	METTL26	CAPN15 (-79865), MIR5587 (-99133), MIR3176 (-91135), gene: ENSG00000261691 (-80654), PRR35 (-68972), PIGQ (-50392), NHLRC4 (-65006), gene: ENSG00000282907 (-58244), RAB40C (-5229), WFIKKN1 (-384), METTL26, MCRIP2 (+2315), gene: ENSG00000228201 (+13971), WDR90 (+14809), gene: ENSG00000262528 (+20109), RHOT2 (+33603), RHBDL1 (+41169), LINC02867 (+44002), gene: ENSG00000279441 (+44143), STUB1 (+45722), JMJD8 (+47168), WDR24 (+50120), gene: ENSG00000261659 (+54499), FBXL16 (+57996), gene: ENSG00000279255 (+73148), gene: ENSG00000259840 (+76244), METRN (+80616), ANTKMT (+86079), CCDC78 (+88080), HAGHL (+92434), CIAO3 (+95258)

Table S4 Viral integration sites reports

30927-005.R1 (paired) (Report)

Host chromosome	Unaligned ends virus	Broken reads host	Disrupted genes	Nearby genes
16	N/A	3	METTL26	CAPN15 (-79868), MIR5587 (-99136), MIR3176 (-91138), gene: ENSG00000261691 (-80657), PRR35 (-68975), PIGQ (-50395), NHLRC4 (-65009), gene: ENSG00000282907 (-58247), RAB40C (-5232), WFIKKN1 (-387), METTL26, MCRIP2 (+2312), gene: ENSG00000228201 (+13968), WDR90 (+14806), gene: ENSG00000262528 (+20106), RHOT2 (+33600), RHBDL1 (+41166), LINC02867 (+43999), gene: ENSG00000279441 (+44140), STUB1 (+45719), JMJD8 (+47165), WDR24 (+50117), gene: ENSG00000261659 (+54496), FBXL16 (+57993), gene: ENSG00000279255 (+73145), gene: ENSG00000259840 (+76241), METRN (+80613), ANTKMT (+86076), CCDC78 (+88077), HAGHL (+92431), CIAO3 (+95255)

30927-005.R1 (paired) (Report)

Host chromosome	Unaligned ends virus	Broken reads host	Disrupted genes	Nearby genes
16	3,426	2	METTL26	CAPN15 (-79874), MIR5587 (-99142), MIR3176 (-91144), gene: ENSG00000261691 (-80663), PRR35 (-68981), PIGQ (-50401), NHLRC4 (-65015), gene: ENSG00000282907 (-58253), RAB40C (-5238), WFIKKN1 (-393), METTL26, MCRIP2 (+2306), gene: ENSG00000228201 (+13962), WDR90 (+14800), gene: ENSG00000262528 (+20100), RHOT2 (+33594), RHBDL1 (+41160), LINC02867 (+43993), gene: ENSG00000279441 (+44134), STUB1 (+45713), JMJD8 (+47159), WDR24 (+50111), gene: ENSG00000261659 (+54490), FBXL16 (+57987), gene: ENSG00000279255 (+73139), gene: ENSG00000259840 (+76235), METRN (+80607), ANTKMT (+86070), CCDC78 (+88071), HAGHL (+92425), CIAO3 (+95249)

30927-005.R1 (paired) (Report)

Host chromosome	Unaligned ends virus	Broken reads host	Disrupted genes	Nearby genes
16	N/A	2	METTL26	CAPN15 (-79887), MIR5587 (-99155), MIR3176 (-91157), gene: ENSG00000261691 (-80676), PRR35 (-68994), PIGQ (-50414), NHLRC4 (-65028), gene: ENSG00000282907 (-58266), RAB40C (-5251), WFIKKN1 (-406), METTL26, MCRIP2 (+2293), gene: ENSG00000228201 (+13949), WDR90 (+14787), gene: ENSG00000262528 (+20087), RHOT2 (+33581), RHBDL1 (+41147), LINC02867 (+43980), gene: ENSG00000279441 (+44121), STUB1 (+45700), JMJD8 (+47146), WDR24 (+50098), gene: ENSG00000261659 (+54477), FBXL16 (+57974), gene: ENSG00000279255 (+73126), gene: ENSG00000259840 (+76222), METRN (+80594), ANTKMT (+86057), CCDC78 (+88058), HAGHL (+92412), CIAO3 (+95236)

30927-005.R1 (paired) (Report)

Host chromosome	Unaligned ends virus	Broken reads host	Disrupted genes	Nearby genes
16	6,702	2	METTL26	CAPN15 (-79888), MIR5587 (-99156), MIR3176 (-91158), gene: ENSG00000261691 (-80677), PRR35 (-68995), PIGQ (-50415), NHLRC4 (-65029), gene: ENSG00000282907 (-58267), RAB40C (-5252), WFIKKN1 (-407), METTL26, MCRIP2 (+2292), gene: ENSG00000228201 (+13948), WDR90 (+14786), gene: ENSG00000262528 (+20086), RHOT2 (+33580), RHBDL1 (+41146), LINC02867 (+43979), gene: ENSG00000279441 (+44120), STUB1 (+45699), JMJD8 (+47145), WDR24 (+50097), gene: ENSG00000261659 (+54476), FBXL16 (+57973), gene: ENSG00000279255 (+73125), gene: ENSG00000259840 (+76221), METRN (+80593), ANTKMT (+86056), CCDC78 (+88057), HAGHL (+92411), CIAO3 (+95235)

30927-005.R1 (paired) (Report)

Host chromosome	Unaligned ends virus	Broken reads host	Disrupted genes	Nearby genes
16	320	3	METTL26	CAPN15 (-79844), MIR5587 (-99112), MIR3176 (-91114), gene: ENSG00000261691 (-80633), PRR35 (-68951), PIGQ (-50371), NHLRC4 (-64985), gene: ENSG00000282907 (-58223), RAB40C (-5208), WFIKKN1 (-363), METTL26, MCRIP2 (+2336), gene: ENSG00000228201 (+13992), WDR90 (+14830), gene: ENSG00000262528 (+20130), RHOT2 (+33624), RHBDL1 (+41190), LINC02867 (+44023), gene: ENSG00000279441 (+44164), STUB1 (+45743), JMJD8 (+47189), WDR24 (+50141), gene: ENSG00000261659 (+54520), FBXL16 (+58017), gene: ENSG00000279255 (+73169), gene: ENSG00000259840 (+76265), METRN (+80637), ANTKMT (+86100), CCDC78 (+88101), HAGHL (+92455), CIAO3 (+95279)

30927-005.R1 (paired) (Report)

Host chromosome	Unaligned ends virus	Broken reads host	Disrupted genes	Nearby genes
21	N/A	15		gene: ENSG00000278996 (-30550), MIR6724-1 (-52790), gene: ENSG00000275664 (-52256), MIR3648-1 (-49544), gene: ENSG00000277437 (-49292), gene: ENSG00000280800 (-46890), RNA5-8SN2 (-45472), gene: ENSG00000286155 (-22364), gene: ENSG00000286252 (-20217), gene: ENSG00000286057 (-19104), gene: ENSG00000286012 (-17149), MIR6724-2 (-8600), gene: ENSG00000277671 (-8047), gene: ENSG00000281383 (-2682), 5_8S_rRNA (-1263)
21	3,276	12		gene: ENSG00000278996 (-30557), MIR6724-1 (-52797), gene: ENSG00000275664 (-52263), MIR3648-1 (-49551), gene: ENSG00000277437 (-49299), gene: ENSG00000280800 (-46897), RNA5-8SN2 (-45479), gene: ENSG00000286155 (-22371), gene: ENSG00000286252 (-20224), gene: ENSG00000286057 (-19111), gene: ENSG00000286012 (-17156), MIR6724-2 (-8607), gene: ENSG00000277671 (-8054), gene: ENSG00000281383 (-2689), 5_8S_rRNA (-1270)

30927-005.R1 (paired) (Report)

Host chromosome	Unaligned ends virus	Broken reads host	Disrupted genes	Nearby genes
21	N/A	7		gene: ENSG00000278996 (-30575), MIR6724-1 (-52815), gene: ENSG00000275664 (-52281), MIR3648-1 (-49569), gene: ENSG00000277437 (-49317), gene: ENSG00000280800 (-46915), RNA5-8SN2 (-45497), gene: ENSG00000286155 (-22389), gene: ENSG00000286252 (-20242), gene: ENSG00000286057 (-19129), gene: ENSG00000286012 (-17174), MIR6724-2 (-8625), gene: ENSG00000277671 (-8072), gene: ENSG00000281383 (-2707), 5_8S_rRNA (-1288)
21	4,496	44		gene: ENSG00000278996 (-31792), MIR6724-1 (-54032), gene: ENSG00000275664 (-53498), MIR3648-1 (-50786), gene: ENSG00000277437 (-50534), gene: ENSG00000280800 (-48132), RNA5-8SN2 (-46714), gene: ENSG00000286155 (-23606), gene: ENSG00000286252 (-21459), gene: ENSG00000286057 (-20346), gene: ENSG00000286012 (-18391), MIR6724-2 (-9842), gene: ENSG00000277671 (-9289), gene: ENSG00000281383 (-3924), 5_8S_rRNA (-2505)

30927-005.R1 (paired) (Report)

Host chromosome	Unaligned ends virus	Broken reads host	Disrupted genes	Nearby genes
21	N/A	45		gene: ENSG00000278996 (-31795), MIR6724-1 (-54035), gene: ENSG00000275664 (-53501), MIR3648-1 (-50789), gene: ENSG00000277437 (-50537), gene: ENSG00000280800 (-48135), RNA5-8SN2 (-46717), gene: ENSG00000286155 (-23609), gene: ENSG00000286252 (-21462), gene: ENSG00000286057 (-20349), gene: ENSG00000286012 (-18394), MIR6724-2 (-9845), gene: ENSG00000277671 (-9292), gene: ENSG00000281383 (-3927), 5_8S_rRNA (-2508)
21	2,821	42		gene: ENSG00000278996 (-31815), MIR6724-1 (-54055), gene: ENSG00000275664 (-53521), MIR3648-1 (-50809), gene: ENSG00000277437 (-50557), gene: ENSG00000280800 (-48155), RNA5-8SN2 (-46737), gene: ENSG00000286155 (-23629), gene: ENSG00000286252 (-21482), gene: ENSG00000286057 (-20369), gene: ENSG00000286012 (-18414), MIR6724-2 (-9865), gene: ENSG00000277671 (-9312), gene: ENSG00000281383 (-3947), 5_8S_rRNA (-2528)

30927-005.R1 (paired) (Report)

Host chromosome	Unaligned ends virus	Broken reads host	Disrupted genes	Nearby genes
21	3,156	42		gene: ENSG00000278996 (-31819), MIR6724-1 (-54059), gene: ENSG00000275664 (-53525), MIR3648-1 (-50813), gene: ENSG00000277437 (-50561), gene: ENSG00000280800 (-48159), RNA5-8SN2 (-46741), gene: ENSG00000286155 (-23633), gene: ENSG00000286252 (-21486), gene: ENSG00000286057 (-20373), gene: ENSG00000286012 (-18418), MIR6724-2 (-9869), gene: ENSG00000277671 (-9316), gene: ENSG00000281383 (-3951), 5_8S_rRNA (-2532)
21	N/A	38		gene: ENSG00000278996 (-31827), MIR6724-1 (-54067), gene: ENSG00000275664 (-53533), MIR3648-1 (-50821), gene: ENSG00000277437 (-50569), gene: ENSG00000280800 (-48167), RNA5-8SN2 (-46749), gene: ENSG00000286155 (-23641), gene: ENSG00000286252 (-21494), gene: ENSG00000286057 (-20381), gene: ENSG00000286012 (-18426), MIR6724-2 (-9877), gene: ENSG00000277671 (-9324), gene: ENSG00000281383 (-3959), 5_8S_rRNA (-2540)

Table S4 Viral integration sites reports

30927-005.R1 (paired) (Report)

Host chromosome	Unaligned ends virus	Broken reads host	Disrupted genes	Nearby genes
21	N/A	39		gene: ENSG00000278996 (-31829), MIR6724-1 (-54069), gene: ENSG00000275664 (-53535), MIR3648-1 (-50823), gene: ENSG00000277437 (-50571), gene: ENSG00000280800 (-48169), RNA5-8SN2 (-46751), gene: ENSG00000286155 (-23643), gene: ENSG00000286252 (-21496), gene: ENSG00000286057 (-20383), gene: ENSG00000286012 (-18428), MIR6724-2 (-9879), gene: ENSG00000277671 (-9326), gene: ENSG00000281383 (-3961), 5_8S_rRNA (-2542)
21	61,253	34		gene: ENSG00000278996 (-31845), MIR6724-1 (-54085), gene: ENSG00000275664 (-53551), MIR3648-1 (-50839), gene: ENSG00000277437 (-50587), gene: ENSG00000280800 (-48185), RNA5-8SN2 (-46767), gene: ENSG00000286155 (-23659), gene: ENSG00000286252 (-21512), gene: ENSG00000286057 (-20399), gene: ENSG00000286012 (-18444), MIR6724-2 (-9895), gene: ENSG00000277671 (-9342), gene: ENSG00000281383 (-3977), 5_8S_rRNA (-2558)

30927-005.R1 (paired) (Report)

Host chromosome	Unaligned ends virus	Broken reads host	Disrupted genes	Nearby genes
21	N/A	34		gene: ENSG00000278996 (-31847), MIR6724-1 (-54087), gene: ENSG00000275664 (-53553), MIR3648-1 (-50841), gene: ENSG00000277437 (-50589), gene: ENSG00000280800 (-48187), RNA5-8SN2 (-46769), gene: ENSG00000286155 (-23661), gene: ENSG00000286252 (-21514), gene: ENSG00000286057 (-20401), gene: ENSG00000286012 (-18446), MIR6724-2 (-9897), gene: ENSG00000277671 (-9344), gene: ENSG00000281383 (-3979), 5_8S_rRNA (-2560)
21	N/A	27		gene: ENSG00000278996 (-31857), MIR6724-1 (-54097), gene: ENSG00000275664 (-53563), MIR3648-1 (-50851), gene: ENSG00000277437 (-50599), gene: ENSG00000280800 (-48197), RNA5-8SN2 (-46779), gene: ENSG00000286155 (-23671), gene: ENSG00000286252 (-21524), gene: ENSG00000286057 (-20411), gene: ENSG00000286012 (-18456), MIR6724-2 (-9907), gene: ENSG00000277671 (-9354), gene: ENSG00000281383 (-3989), 5_8S_rRNA (-2570)

30927-005.R1 (paired) (Report)

Host chromosome	Unaligned ends virus	Broken reads host	Disrupted genes	Nearby genes
21	N/A	21		gene: ENSG00000278996 (-31940), MIR6724-1 (-54180), gene: ENSG00000275664 (-53646), MIR3648-1 (-50934), gene: ENSG00000277437 (-50682), gene: ENSG00000280800 (-48280), RNA5-8SN2 (-46862), gene: ENSG00000286155 (-23754), gene: ENSG00000286252 (-21607), gene: ENSG00000286057 (-20494), gene: ENSG00000286012 (-18539), MIR6724-2 (-9990), gene: ENSG00000277671 (-9437), gene: ENSG00000281383 (-4072), 5_8S_rRNA (-2653)
21	N/A	21		gene: ENSG00000278996 (-31948), MIR6724-1 (-54188), gene: ENSG00000275664 (-53654), MIR3648-1 (-50942), gene: ENSG00000277437 (-50690), gene: ENSG00000280800 (-48288), RNA5-8SN2 (-46870), gene: ENSG00000286155 (-23762), gene: ENSG00000286252 (-21615), gene: ENSG00000286057 (-20502), gene: ENSG00000286012 (-18547), MIR6724-2 (-9998), gene: ENSG00000277671 (-9445), gene: ENSG00000281383 (-4080), 5_8S_rRNA (-2661)

30927-005.R1 (paired) (Report)

Host chromosome	Unaligned ends virus	Broken reads host	Disrupted genes	Nearby genes
21	N/A	21		gene: ENSG00000278996 (-31958), MIR6724-1 (-54198), gene: ENSG00000275664 (-53664), MIR3648-1 (-50952), gene: ENSG00000277437 (-50700), gene: ENSG00000280800 (-48298), RNA5-8SN2 (-46880), gene: ENSG00000286155 (-23772), gene: ENSG00000286252 (-21625), gene: ENSG00000286057 (-20512), gene: ENSG00000286012 (-18557), MIR6724-2 (-10008), gene: ENSG00000277671 (-9455), gene: ENSG00000281383 (-4090), 5_8S_rRNA (-2671)
21	4,358	18		gene: ENSG00000278996 (-31984), MIR6724-1 (-54224), gene: ENSG00000275664 (-53690), MIR3648-1 (-50978), gene: ENSG00000277437 (-50726), gene: ENSG00000280800 (-48324), RNA5-8SN2 (-46906), gene: ENSG00000286155 (-23798), gene: ENSG00000286252 (-21651), gene: ENSG00000286057 (-20538), gene: ENSG00000286012 (-18583), MIR6724-2 (-10034), gene: ENSG00000277671 (-9481), gene: ENSG00000281383 (-4116), 5_8S_rRNA (-2697)

30927-005.R1 (paired) (Report)

Host chromosome	Unaligned ends virus	Broken reads host	Disrupted genes	Nearby genes
21	N/A	18		gene: ENSG00000278996 (-31992), MIR6724-1 (-54232), gene: ENSG00000275664 (-53698), MIR3648-1 (-50986), gene: ENSG00000277437 (-50734), gene: ENSG00000280800 (-48332), RNA5-8SN2 (-46914), gene: ENSG00000286155 (-23806), gene: ENSG00000286252 (-21659), gene: ENSG00000286057 (-20546), gene: ENSG00000286012 (-18591), MIR6724-2 (-10042), gene: ENSG00000277671 (-9489), gene: ENSG00000281383 (-4124), 5_8S_rRNA (-2705)
21	4,442	14		gene: ENSG00000278996 (-32060), MIR6724-1 (-54300), gene: ENSG00000275664 (-53766), MIR3648-1 (-51054), gene: ENSG00000277437 (-50802), gene: ENSG00000280800 (-48400), RNA5-8SN2 (-46982), gene: ENSG00000286155 (-23874), gene: ENSG00000286252 (-21727), gene: ENSG00000286057 (-20614), gene: ENSG00000286012 (-18659), MIR6724-2 (-10110), gene: ENSG00000277671 (-9557), gene: ENSG00000281383 (-4192), 5_8S_rRNA (-2773)

30927-005.R1 (paired) (Report)

Host chromosome	Unaligned ends virus	Broken reads host	Disrupted genes	Nearby genes
21	N/A	13		gene: ENSG00000278996 (-32258), MIR6724-1 (-54498), gene: ENSG00000275664 (-53964), MIR3648-1 (-51252), gene: ENSG00000277437 (-51000), gene: ENSG00000280800 (-48598), RNA5-8SN2 (-47180), gene: ENSG00000286155 (-24072), gene: ENSG00000286252 (-21925), gene: ENSG00000286057 (-20812), gene: ENSG00000286012 (-18857), MIR6724-2 (-10308), gene: ENSG00000277671 (-9755), gene: ENSG00000281383 (-4390), 5_8S_rRNA (-2971)
21	3,510	13		gene: ENSG00000278996 (-32362), MIR6724-1 (-54602), gene: ENSG00000275664 (-54068), MIR3648-1 (-51356), gene: ENSG00000277437 (-51104), gene: ENSG00000280800 (-48702), RNA5-8SN2 (-47284), gene: ENSG00000286155 (-24176), gene: ENSG00000286252 (-22029), gene: ENSG00000286057 (-20916), gene: ENSG00000286012 (-18961), MIR6724-2 (-10412), gene: ENSG00000277671 (-9859), gene: ENSG00000281383 (-4494), 5_8S_rRNA (-3075)

30927-005.R1 (paired) (Report)

Host chromosome	Unaligned ends virus	Broken reads host	Disrupted genes	Nearby genes
21	4,442	13		gene: ENSG00000278996 (-32438), MIR6724-1 (-54678), gene: ENSG00000275664 (-54144), MIR3648-1 (-51432), gene: ENSG00000277437 (-51180), gene: ENSG00000280800 (-48778), RNA5-8SN2 (-47360), gene: ENSG00000286155 (-24252), gene: ENSG00000286252 (-22105), gene: ENSG00000286057 (-20992), gene: ENSG00000286012 (-19037), MIR6724-2 (-10488), gene: ENSG00000277671 (-9935), gene: ENSG00000281383 (-4570), 5_8S_rRNA (-3151)
21	N/A	13		gene: ENSG00000278996 (-32440), MIR6724-1 (-54680), gene: ENSG00000275664 (-54146), MIR3648-1 (-51434), gene: ENSG00000277437 (-51182), gene: ENSG00000280800 (-48780), RNA5-8SN2 (-47362), gene: ENSG00000286155 (-24254), gene: ENSG00000286252 (-22107), gene: ENSG00000286057 (-20994), gene: ENSG00000286012 (-19039), MIR6724-2 (-10490), gene: ENSG00000277671 (-9937), gene: ENSG00000281383 (-4572), 5_8S_rRNA (-3153)

30927-005.R1 (paired) (Report)

Host chromosome	Unaligned ends virus	Broken reads host	Disrupted genes	Nearby genes
21	N/A	13		gene: ENSG00000278996 (-32897), MIR6724-1 (-55137), gene: ENSG00000275664 (-54603), MIR3648-1 (-51891), gene: ENSG00000277437 (-51639), gene: ENSG00000280800 (-49237), RNA5-8SN2 (-47819), gene: ENSG00000286155 (-24711), gene: ENSG00000286252 (-22564), gene: ENSG00000286057 (-21451), gene: ENSG00000286012 (-19496), MIR6724-2 (-10947), gene: ENSG00000277671 (-10394), gene: ENSG00000281383 (-5029), 5_8S_rRNA (-3610)
21	N/A	12		gene: ENSG00000278996 (-32904), MIR6724-1 (-55144), gene: ENSG00000275664 (-54610), MIR3648-1 (-51898), gene: ENSG00000277437 (-51646), gene: ENSG00000280800 (-49244), RNA5-8SN2 (-47826), gene: ENSG00000286155 (-24718), gene: ENSG00000286252 (-22571), gene: ENSG00000286057 (-21458), gene: ENSG00000286012 (-19503), MIR6724-2 (-10954), gene: ENSG00000277671 (-10401), gene: ENSG00000281383 (-5036), 5_8S_rRNA (-3617)

30927-005.R1 (paired) (Report)

Host chromosome	Unaligned ends virus	Broken reads host	Disrupted genes	Nearby genes
21	N/A	12		gene: ENSG00000278996 (-32912), MIR6724-1 (-55152), gene: ENSG00000275664 (-54618), MIR3648-1 (-51906), gene: ENSG00000277437 (-51654), gene: ENSG00000280800 (-49252), RNA5-8SN2 (-47834), gene: ENSG00000286155 (-24726), gene: ENSG00000286252 (-22579), gene: ENSG00000286057 (-21466), gene: ENSG00000286012 (-19511), MIR6724-2 (-10962), gene: ENSG00000277671 (-10409), gene: ENSG00000281383 (-5044), 5_8S_rRNA (-3625)
21	5,000	3		gene: ENSG00000278996 (-33012), MIR6724-1 (-55252), gene: ENSG00000275664 (-54718), MIR3648-1 (-52006), gene: ENSG00000277437 (-51754), gene: ENSG00000280800 (-49352), RNA5-8SN2 (-47934), gene: ENSG00000286155 (-24826), gene: ENSG00000286252 (-22679), gene: ENSG00000286057 (-21566), gene: ENSG00000286012 (-19611), MIR6724-2 (-11062), gene: ENSG00000277671 (-10509), gene: ENSG00000281383 (-5144), 5_8S_rRNA (-3725)

30927-005.R1 (paired) (Report)

Host chromosome	Unaligned ends virus	Broken reads host	Disrupted genes	Nearby genes
21	3,156	7	gene: ENSG00000280441	gene: ENSG00000280441, MIR6724-3 (-4405), gene: ENSG00000274868 (-3871), gene: ENSG00000280614 (+560), RNA5-8SN3 (+2748), gene: ENSG00000286267 (+24898), gene: ENSG00000286146 (+27816), gene: ENSG00000286054 (+29024), gene: ENSG00000286178 (+30901), MIR6724-4 (+39671), gene: ENSG00000278775 (+40226), gene: ENSG00000281181 (+44770), RNA5-8SN1 (+46964), gene: ENSG00000286149 (+69461), gene: ENSG00000286032 (+72395), gene: ENSG00000286091 (+73607), gene: ENSG00000286148 (+75461)

30927-005.R1 (paired) (Report)

Host chromosome	Unaligned ends virus	Broken reads host	Disrupted genes	Nearby genes
21	N/A	7	gene: ENSG00000280441	gene: ENSG00000280441, MIR6724-3 (-4426), gene: ENSG00000274868 (-3892), gene: ENSG00000280614 (+539), RNA5-8SN3 (+2727), gene: ENSG00000286267 (+24877), gene: ENSG00000286146 (+27795), gene: ENSG00000286054 (+29003), gene: ENSG00000286178 (+30880), MIR6724-4 (+39650), gene: ENSG00000278775 (+40205), gene: ENSG00000281181 (+44749), RNA5-8SN1 (+46943), gene: ENSG00000286149 (+69440), gene: ENSG00000286032 (+72374), gene: ENSG00000286091 (+73586), gene: ENSG00000286148 (+75440)

30927-005.R1 (paired) (Report)

Host chromosome	Unaligned ends virus	Broken reads host	Disrupted genes	Nearby genes
21	61,253	7	gene: ENSG00000280441	gene: ENSG00000280441, MIR6724-3 (-4451), gene: ENSG00000274868 (-3917), gene: ENSG00000280614 (+514), RNA5-8SN3 (+2702), gene: ENSG00000286267 (+24852), gene: ENSG00000286146 (+27770), gene: ENSG00000286054 (+28978), gene: ENSG00000286178 (+30855), MIR6724-4 (+39625), gene: ENSG00000278775 (+40180), gene: ENSG00000281181 (+44724), RNA5-8SN1 (+46918), gene: ENSG00000286149 (+69415), gene: ENSG00000286032 (+72349), gene: ENSG00000286091 (+73561), gene: ENSG00000286148 (+75415)

30927-005.R1 (paired) (Report)

Host chromosome	Unaligned ends virus	Broken reads host	Disrupted genes	Nearby genes
21	6,188	15	gene: ENSG00000280441, gene: ENSG00000280614	gene: ENSG00000280441, MIR6724-3 (-5601), gene: ENSG00000274868 (-5067), gene: ENSG00000280614, RNA5-8SN3 (+1552), gene: ENSG00000286267 (+23702), gene: ENSG00000286146 (+26620), gene: ENSG00000286054 (+27828), gene: ENSG00000286178 (+29705), MIR6724-4 (+38475), gene: ENSG00000278775 (+39030), gene: ENSG00000281181 (+43574), RNA5-8SN1 (+45768), gene: ENSG00000286149 (+68265), gene: ENSG00000286032 (+71199), gene: ENSG00000286091 (+72411), gene: ENSG00000286148 (+74265)

30927-005.R1 (paired) (Report)

Host chromosome	Unaligned ends virus	Broken reads host	Disrupted genes	Nearby genes
21	3,156	15	gene: ENSG00000280441, gene: ENSG00000280614	gene: ENSG00000280441, MIR6724-3 (-5607), gene: ENSG00000274868 (-5073), gene: ENSG00000280614, RNA5-8SN3 (+1546), gene: ENSG00000286267 (+23696), gene: ENSG00000286146 (+26614), gene: ENSG00000286054 (+27822), gene: ENSG00000286178 (+29699), MIR6724-4 (+38469), gene: ENSG00000278775 (+39024), gene: ENSG00000281181 (+43568), RNA5-8SN1 (+45762), gene: ENSG00000286149 (+68259), gene: ENSG00000286032 (+71193), gene: ENSG00000286091 (+72405), gene: ENSG00000286148 (+74259)

30927-005.R1 (paired) (Report)

Host chromosome	Unaligned ends virus	Broken reads host	Disrupted genes	Nearby genes
21	1,929	10	gene: ENSG00000280441, gene: ENSG00000280614	gene: ENSG00000280441, MIR6724-3 (-5660), gene: ENSG00000274868 (-5126), gene: ENSG00000280614, RNA5-8SN3 (+1493), gene: ENSG00000286267 (+23643), gene: ENSG00000286146 (+26561), gene: ENSG00000286054 (+27769), gene: ENSG00000286178 (+29646), MIR6724-4 (+38416), gene: ENSG00000278775 (+38971), gene: ENSG00000281181 (+43515), RNA5-8SN1 (+45709), gene: ENSG00000286149 (+68206), gene: ENSG00000286032 (+71140), gene: ENSG00000286091 (+72352), gene: ENSG00000286148 (+74206)

30927-005.R1 (paired) (Report)

Host chromosome	Unaligned ends virus	Broken reads host	Disrupted genes	Nearby genes
21	2,821	20	gene: ENSG00000280441	gene: ENSG00000280441, MIR6724-3 (-8560), gene: ENSG00000274868 (-8026), gene: ENSG00000280614 (-2672), RNA5-8SN3 (-1254), gene: ENSG00000286267 (+20743), gene: ENSG00000286146 (+23661), gene: ENSG00000286054 (+24869), gene: ENSG00000286178 (+26746), MIR6724-4 (+35516), gene: ENSG00000278775 (+36071), gene: ENSG00000281181 (+40615), RNA5-8SN1 (+42809), gene: ENSG00000286149 (+65306), gene: ENSG00000286032 (+68240), gene: ENSG00000286091 (+69452), gene: ENSG00000286148 (+71306)

30927-005.R1 (paired) (Report)

Host chromosome	Unaligned ends virus	Broken reads host	Disrupted genes	Nearby genes
21	N/A	20	gene: ENSG00000280441	gene: ENSG00000280441, MIR6724-3 (-8561), gene: ENSG00000274868 (-8027), gene: ENSG00000280614 (-2673), RNA5-8SN3 (-1255), gene: ENSG00000286267 (+20742), gene: ENSG00000286146 (+23660), gene: ENSG00000286054 (+24868), gene: ENSG00000286178 (+26745), MIR6724-4 (+35515), gene: ENSG00000278775 (+36070), gene: ENSG00000281181 (+40614), RNA5-8SN1 (+42808), gene: ENSG00000286149 (+65305), gene: ENSG00000286032 (+68239), gene: ENSG00000286091 (+69451), gene: ENSG00000286148 (+71305)

30927-005.R1 (paired) (Report)

Host chromosome	Unaligned ends virus	Broken reads host	Disrupted genes	Nearby genes
21	3,276	16	gene: ENSG00000280441	gene: ENSG00000280441, MIR6724-3 (-8567), gene: ENSG00000274868 (-8033), gene: ENSG00000280614 (-2679), RNA5-8SN3 (-1261), gene: ENSG00000286267 (+20736), gene: ENSG00000286146 (+23654), gene: ENSG00000286054 (+24862), gene: ENSG00000286178 (+26739), MIR6724-4 (+35509), gene: ENSG00000278775 (+36064), gene: ENSG00000281181 (+40608), RNA5-8SN1 (+42802), gene: ENSG00000286149 (+65299), gene: ENSG00000286032 (+68233), gene: ENSG00000286091 (+69445), gene: ENSG00000286148 (+71299)

30927-005.R1 (paired) (Report)

Host chromosome	Unaligned ends virus	Broken reads host	Disrupted genes	Nearby genes
21	92	14	gene: ENSG00000280441	gene: ENSG00000280441, MIR6724-3 (-8593), gene: ENSG00000274868 (-8059), gene: ENSG00000280614 (-2705), RNA5-8SN3 (-1287), gene: ENSG00000286267 (+20710), gene: ENSG00000286146 (+23628), gene: ENSG00000286054 (+24836), gene: ENSG00000286178 (+26713), MIR6724-4 (+35483), gene: ENSG00000278775 (+36038), gene: ENSG00000281181 (+40582), RNA5-8SN1 (+42776), gene: ENSG00000286149 (+65273), gene: ENSG00000286032 (+68207), gene: ENSG00000286091 (+69419), gene: ENSG00000286148 (+71273)

30927-005.R1 (paired) (Report)

Host chromosome	Unaligned ends virus	Broken reads host	Disrupted genes	Nearby genes
21	N/A	5	gene: ENSG00000280441	gene: ENSG00000280441, MIR6724-3 (-8905), gene: ENSG00000274868 (-8371), gene: ENSG00000280614 (-3017), RNA5-8SN3 (-1599), gene: ENSG00000286267 (+20398), gene: ENSG00000286146 (+23316), gene: ENSG00000286054 (+24524), gene: ENSG00000286178 (+26401), MIR6724-4 (+35171), gene: ENSG00000278775 (+35726), gene: ENSG00000281181 (+40270), RNA5-8SN1 (+42464), gene: ENSG00000286149 (+64961), gene: ENSG00000286032 (+67895), gene: ENSG00000286091 (+69107), gene: ENSG00000286148 (+70961)

30927-005.R1 (paired) (Report)

Host chromosome	Unaligned ends virus	Broken reads host	Disrupted genes	Nearby genes
21	N/A	28	gene: ENSG00000280441	gene: ENSG00000280441, MIR6724-3 (-9792), gene: ENSG00000274868 (-9258), gene: ENSG00000280614 (-3904), RNA5-8SN3 (-2486), gene: ENSG00000286267 (+19511), gene: ENSG00000286146 (+22429), gene: ENSG00000286054 (+23637), gene: ENSG00000286178 (+25514), MIR6724-4 (+34284), gene: ENSG00000278775 (+34839), gene: ENSG00000281181 (+39383), RNA5-8SN1 (+41577), gene: ENSG00000286149 (+64074), gene: ENSG00000286032 (+67008), gene: ENSG00000286091 (+68220), gene: ENSG00000286148 (+70074)

30927-005.R1 (paired) (Report)

Host chromosome	Unaligned ends virus	Broken reads host	Disrupted genes	Nearby genes
21	N/A	29	gene: ENSG00000280441	gene: ENSG00000280441, MIR6724-3 (-9800), gene: ENSG00000274868 (-9266), gene: ENSG00000280614 (-3912), RNA5-8SN3 (-2494), gene: ENSG00000286267 (+19503), gene: ENSG00000286146 (+22421), gene: ENSG00000286054 (+23629), gene: ENSG00000286178 (+25506), MIR6724-4 (+34276), gene: ENSG00000278775 (+34831), gene: ENSG00000281181 (+39375), RNA5-8SN1 (+41569), gene: ENSG00000286149 (+64066), gene: ENSG00000286032 (+67000), gene: ENSG00000286091 (+68212), gene: ENSG00000286148 (+70066)

30927-005.R1 (paired) (Report)

Host chromosome	Unaligned ends virus	Broken reads host	Disrupted genes	Nearby genes
21	N/A	29	gene: ENSG00000280441	gene: ENSG00000280441, MIR6724-3 (-9810), gene: ENSG00000274868 (-9276), gene: ENSG00000280614 (-3922), RNA5-8SN3 (-2504), gene: ENSG00000286267 (+19493), gene: ENSG00000286146 (+22411), gene: ENSG00000286054 (+23619), gene: ENSG00000286178 (+25496), MIR6724-4 (+34266), gene: ENSG00000278775 (+34821), gene: ENSG00000281181 (+39365), RNA5-8SN1 (+41559), gene: ENSG00000286149 (+64056), gene: ENSG00000286032 (+66990), gene: ENSG00000286091 (+68202), gene: ENSG00000286148 (+70056)

30927-005.R1 (paired) (Report)

Host chromosome	Unaligned ends virus	Broken reads host	Disrupted genes	Nearby genes
21	N/A	29	gene: ENSG00000280441	gene: ENSG00000280441, MIR6724-3 (-9812), gene: ENSG00000274868 (-9278), gene: ENSG00000280614 (-3924), RNA5-8SN3 (-2506), gene: ENSG00000286267 (+19491), gene: ENSG00000286146 (+22409), gene: ENSG00000286054 (+23617), gene: ENSG00000286178 (+25494), MIR6724-4 (+34264), gene: ENSG00000278775 (+34819), gene: ENSG00000281181 (+39363), RNA5-8SN1 (+41557), gene: ENSG00000286149 (+64054), gene: ENSG00000286032 (+66988), gene: ENSG00000286091 (+68200), gene: ENSG00000286148 (+70054)

30927-005.R1 (paired) (Report)

Host chromosome	Unaligned ends virus	Broken reads host	Disrupted genes	Nearby genes
21	8,627	29	gene: ENSG00000280441	gene: ENSG00000280441, MIR6724-3 (-9817), gene: ENSG00000274868 (-9283), gene: ENSG00000280614 (-3929), RNA5-8SN3 (-2511), gene: ENSG00000286267 (+19486), gene: ENSG00000286146 (+22404), gene: ENSG00000286054 (+23612), gene: ENSG00000286178 (+25489), MIR6724-4 (+34259), gene: ENSG00000278775 (+34814), gene: ENSG00000281181 (+39358), RNA5-8SN1 (+41552), gene: ENSG00000286149 (+64049), gene: ENSG00000286032 (+66983), gene: ENSG00000286091 (+68195), gene: ENSG00000286148 (+70049)

30927-005.R1 (paired) (Report)

Host chromosome	Unaligned ends virus	Broken reads host	Disrupted genes	Nearby genes
21	N/A	29	gene: ENSG00000280441	gene: ENSG00000280441, MIR6724-3 (-9820), gene: ENSG00000274868 (-9286), gene: ENSG00000280614 (-3932), RNA5-8SN3 (-2514), gene: ENSG00000286267 (+19483), gene: ENSG00000286146 (+22401), gene: ENSG00000286054 (+23609), gene: ENSG00000286178 (+25486), MIR6724-4 (+34256), gene: ENSG00000278775 (+34811), gene: ENSG00000281181 (+39355), RNA5-8SN1 (+41549), gene: ENSG00000286149 (+64046), gene: ENSG00000286032 (+66980), gene: ENSG00000286091 (+68192), gene: ENSG00000286148 (+70046)

30927-005.R1 (paired) (Report)

Host chromosome	Unaligned ends virus	Broken reads host	Disrupted genes	Nearby genes
21	N/A	28	gene: ENSG00000280441	gene: ENSG00000280441, MIR6724-3 (-9825), gene: ENSG00000274868 (-9291), gene: ENSG00000280614 (-3937), RNA5-8SN3 (-2519), gene: ENSG00000286267 (+19478), gene: ENSG00000286146 (+22396), gene: ENSG00000286054 (+23604), gene: ENSG00000286178 (+25481), MIR6724-4 (+34251), gene: ENSG00000278775 (+34806), gene: ENSG00000281181 (+39350), RNA5-8SN1 (+41544), gene: ENSG00000286149 (+64041), gene: ENSG00000286032 (+66975), gene: ENSG00000286091 (+68187), gene: ENSG00000286148 (+70041)

30927-005.R1 (paired) (Report)

Host chromosome	Unaligned ends virus	Broken reads host	Disrupted genes	Nearby genes
21	N/A	25	gene: ENSG00000280441	gene: ENSG00000280441, MIR6724-3 (-9843), gene: ENSG00000274868 (-9309), gene: ENSG00000280614 (-3955), RNA5-8SN3 (-2537), gene: ENSG00000286267 (+19460), gene: ENSG00000286146 (+22378), gene: ENSG00000286054 (+23586), gene: ENSG00000286178 (+25463), MIR6724-4 (+34233), gene: ENSG00000278775 (+34788), gene: ENSG00000281181 (+39332), RNA5-8SN1 (+41526), gene: ENSG00000286149 (+64023), gene: ENSG00000286032 (+66957), gene: ENSG00000286091 (+68169), gene: ENSG00000286148 (+70023)

30927-005.R1 (paired) (Report)

Host chromosome	Unaligned ends virus	Broken reads host	Disrupted genes	Nearby genes
21	3,426	24	gene: ENSG00000280441	gene: ENSG00000280441, MIR6724-3 (-9861), gene: ENSG00000274868 (-9327), gene: ENSG00000280614 (-3973), RNA5-8SN3 (-2555), gene: ENSG00000286267 (+19442), gene: ENSG00000286146 (+22360), gene: ENSG00000286054 (+23568), gene: ENSG00000286178 (+25445), MIR6724-4 (+34215), gene: ENSG00000278775 (+34770), gene: ENSG00000281181 (+39314), RNA5-8SN1 (+41508), gene: ENSG00000286149 (+64005), gene: ENSG00000286032 (+66939), gene: ENSG00000286091 (+68151), gene: ENSG00000286148 (+70005)

30927-005.R1 (paired) (Report)

Host chromosome	Unaligned ends virus	Broken reads host	Disrupted genes	Nearby genes
21	92	24	gene: ENSG00000280441	gene: ENSG00000280441, MIR6724-3 (-9884), gene: ENSG00000274868 (-9350), gene: ENSG00000280614 (-3996), RNA5-8SN3 (-2578), gene: ENSG00000286267 (+19419), gene: ENSG00000286146 (+22337), gene: ENSG00000286054 (+23545), gene: ENSG00000286178 (+25422), MIR6724-4 (+34192), gene: ENSG00000278775 (+34747), gene: ENSG00000281181 (+39291), RNA5-8SN1 (+41485), gene: ENSG00000286149 (+63982), gene: ENSG00000286032 (+66916), gene: ENSG00000286091 (+68128), gene: ENSG00000286148 (+69982)

30927-005.R1 (paired) (Report)

Host chromosome	Unaligned ends virus	Broken reads host	Disrupted genes	Nearby genes
21	N/A	23	gene: ENSG00000280441	gene: ENSG00000280441, MIR6724-3 (-9891), gene: ENSG00000274868 (-9357), gene: ENSG00000280614 (-4003), RNA5-8SN3 (-2585), gene: ENSG00000286267 (+19412), gene: ENSG00000286146 (+22330), gene: ENSG00000286054 (+23538), gene: ENSG00000286178 (+25415), MIR6724-4 (+34185), gene: ENSG00000278775 (+34740), gene: ENSG00000281181 (+39284), RNA5-8SN1 (+41478), gene: ENSG00000286149 (+63975), gene: ENSG00000286032 (+66909), gene: ENSG00000286091 (+68121), gene: ENSG00000286148 (+69975)

30927-005.R1 (paired) (Report)

Host chromosome	Unaligned ends virus	Broken reads host	Disrupted genes	Nearby genes
21	N/A	23	gene: ENSG00000280441	gene: ENSG00000280441, MIR6724-3 (-9910), gene: ENSG00000274868 (-9376), gene: ENSG00000280614 (-4022), RNA5-8SN3 (-2604), gene: ENSG00000286267 (+19393), gene: ENSG00000286146 (+22311), gene: ENSG00000286054 (+23519), gene: ENSG00000286178 (+25396), MIR6724-4 (+34166), gene: ENSG00000278775 (+34721), gene: ENSG00000281181 (+39265), RNA5-8SN1 (+41459), gene: ENSG00000286149 (+63956), gene: ENSG00000286032 (+66890), gene: ENSG00000286091 (+68102), gene: ENSG00000286148 (+69956)

30927-005.R1 (paired) (Report)

Host chromosome	Unaligned ends virus	Broken reads host	Disrupted genes	Nearby genes
21	N/A	23	gene: ENSG00000280441	gene: ENSG00000280441, MIR6724-3 (-9961), gene: ENSG00000274868 (-9427), gene: ENSG00000280614 (-4073), RNA5-8SN3 (-2655), gene: ENSG00000286267 (+19342), gene: ENSG00000286146 (+22260), gene: ENSG00000286054 (+23468), gene: ENSG00000286178 (+25345), MIR6724-4 (+34115), gene: ENSG00000278775 (+34670), gene: ENSG00000281181 (+39214), RNA5-8SN1 (+41408), gene: ENSG00000286149 (+63905), gene: ENSG00000286032 (+66839), gene: ENSG00000286091 (+68051), gene: ENSG00000286148 (+69905)

30927-005.R1 (paired) (Report)

Host chromosome	Unaligned ends virus	Broken reads host	Disrupted genes	Nearby genes
21	N/A	23	gene: ENSG00000280441	gene: ENSG00000280441, MIR6724-3 (-9967), gene: ENSG00000274868 (-9433), gene: ENSG00000280614 (-4079), RNA5-8SN3 (-2661), gene: ENSG00000286267 (+19336), gene: ENSG00000286146 (+22254), gene: ENSG00000286054 (+23462), gene: ENSG00000286178 (+25339), MIR6724-4 (+34109), gene: ENSG00000278775 (+34664), gene: ENSG00000281181 (+39208), RNA5-8SN1 (+41402), gene: ENSG00000286149 (+63899), gene: ENSG00000286032 (+66833), gene: ENSG00000286091 (+68045), gene: ENSG00000286148 (+69899)

30927-005.R1 (paired) (Report)

Host chromosome	Unaligned ends virus	Broken reads host	Disrupted genes	Nearby genes
21	3,156	20	gene: ENSG00000280441	gene: ENSG00000280441, MIR6724-3 (-9981), gene: ENSG00000274868 (-9447), gene: ENSG00000280614 (-4093), RNA5-8SN3 (-2675), gene: ENSG00000286267 (+19322), gene: ENSG00000286146 (+22240), gene: ENSG00000286054 (+23448), gene: ENSG00000286178 (+25325), MIR6724-4 (+34095), gene: ENSG00000278775 (+34650), gene: ENSG00000281181 (+39194), RNA5-8SN1 (+41388), gene: ENSG00000286149 (+63885), gene: ENSG00000286032 (+66819), gene: ENSG00000286091 (+68031), gene: ENSG00000286148 (+69885)

30927-005.R1 (paired) (Report)

Host chromosome	Unaligned ends virus	Broken reads host	Disrupted genes	Nearby genes
21	92	21	gene: ENSG00000280441	gene: ENSG00000280441, MIR6724-3 (-10011), gene: ENSG00000274868 (-9477), gene: ENSG00000280614 (-4123), RNA5-8SN3 (-2705), gene: ENSG00000286267 (+19292), gene: ENSG00000286146 (+22210), gene: ENSG00000286054 (+23418), gene: ENSG00000286178 (+25295), MIR6724-4 (+34065), gene: ENSG00000278775 (+34620), gene: ENSG00000281181 (+39164), RNA5-8SN1 (+41358), gene: ENSG00000286149 (+63855), gene: ENSG00000286032 (+66789), gene: ENSG00000286091 (+68001), gene: ENSG00000286148 (+69855)

30927-005.R1 (paired) (Report)

Host chromosome	Unaligned ends virus	Broken reads host	Disrupted genes	Nearby genes
21	6,188	17	gene: ENSG00000280441	gene: ENSG00000280441, MIR6724-3 (-10028), gene: ENSG00000274868 (-9494), gene: ENSG00000280614 (-4140), RNA5-8SN3 (-2722), gene: ENSG00000286267 (+19275), gene: ENSG00000286146 (+22193), gene: ENSG00000286054 (+23401), gene: ENSG00000286178 (+25278), MIR6724-4 (+34048), gene: ENSG00000278775 (+34603), gene: ENSG00000281181 (+39147), RNA5-8SN1 (+41341), gene: ENSG00000286149 (+63838), gene: ENSG00000286032 (+66772), gene: ENSG00000286091 (+67984), gene: ENSG00000286148 (+69838)

30927-005.R1 (paired) (Report)

Host chromosome	Unaligned ends virus	Broken reads host	Disrupted genes	Nearby genes
21	4,505	15	gene: ENSG00000280441	gene: ENSG00000280441, MIR6724-3 (-10055), gene: ENSG00000274868 (-9521), gene: ENSG00000280614 (-4167), RNA5-8SN3 (-2749), gene: ENSG00000286267 (+19248), gene: ENSG00000286146 (+22166), gene: ENSG00000286054 (+23374), gene: ENSG00000286178 (+25251), MIR6724-4 (+34021), gene: ENSG00000278775 (+34576), gene: ENSG00000281181 (+39120), RNA5-8SN1 (+41314), gene: ENSG00000286149 (+63811), gene: ENSG00000286032 (+66745), gene: ENSG00000286091 (+67957), gene: ENSG00000286148 (+69811)

30927-005.R1 (paired) (Report)

Host chromosome	Unaligned ends virus	Broken reads host	Disrupted genes	Nearby genes
21	N/A	12	gene: ENSG00000280441	gene: ENSG00000280441, MIR6724-3 (-10268), gene: ENSG00000274868 (-9734), gene: ENSG00000280614 (-4380), RNA5-8SN3 (-2962), gene: ENSG00000286267 (+19035), gene: ENSG00000286146 (+21953), gene: ENSG00000286054 (+23161), gene: ENSG00000286178 (+25038), MIR6724-4 (+33808), gene: ENSG00000278775 (+34363), gene: ENSG00000281181 (+38907), RNA5-8SN1 (+41101), gene: ENSG00000286149 (+63598), gene: ENSG00000286032 (+66532), gene: ENSG00000286091 (+67744), gene: ENSG00000286148 (+69598)

30927-005.R1 (paired) (Report)

Host chromosome	Unaligned ends virus	Broken reads host	Disrupted genes	Nearby genes
21	4,261	22	gene: ENSG00000280441	gene: ENSG00000280441, MIR6724-3 (-10441), gene: ENSG00000274868 (-9907), gene: ENSG00000280614 (-4553), RNA5-8SN3 (-3135), gene: ENSG00000286267 (+18862), gene: ENSG00000286146 (+21780), gene: ENSG00000286054 (+22988), gene: ENSG00000286178 (+24865), MIR6724-4 (+33635), gene: ENSG00000278775 (+34190), gene: ENSG00000281181 (+38734), RNA5-8SN1 (+40928), gene: ENSG00000286149 (+63425), gene: ENSG00000286032 (+66359), gene: ENSG00000286091 (+67571), gene: ENSG00000286148 (+69425)

30927-005.R1 (paired) (Report)

Host chromosome	Unaligned ends virus	Broken reads host	Disrupted genes	Nearby genes
21	3,951	22	gene: ENSG00000280441	gene: ENSG00000280441, MIR6724-3 (-10442), gene: ENSG00000274868 (-9908), gene: ENSG00000280614 (-4554), RNA5-8SN3 (-3136), gene: ENSG00000286267 (+18861), gene: ENSG00000286146 (+21779), gene: ENSG00000286054 (+22987), gene: ENSG00000286178 (+24864), MIR6724-4 (+33634), gene: ENSG00000278775 (+34189), gene: ENSG00000281181 (+38733), RNA5-8SN1 (+40927), gene: ENSG00000286149 (+63424), gene: ENSG00000286032 (+66358), gene: ENSG00000286091 (+67570), gene: ENSG00000286148 (+69424)

30927-005.R1 (paired) (Report)

Host chromosome	Unaligned ends virus	Broken reads host	Disrupted genes	Nearby genes
21	60,930	24	gene: ENSG00000280441	gene: ENSG00000280441, MIR6724-3 (-10614), gene: ENSG00000274868 (-10080), gene: ENSG00000280614 (-4726), RNA5-8SN3 (-3308), gene: ENSG00000286267 (+18689), gene: ENSG00000286146 (+21607), gene: ENSG00000286054 (+22815), gene: ENSG00000286178 (+24692), MIR6724-4 (+33462), gene: ENSG00000278775 (+34017), gene: ENSG00000281181 (+38561), RNA5-8SN1 (+40755), gene: ENSG00000286149 (+63252), gene: ENSG00000286032 (+66186), gene: ENSG00000286091 (+67398), gene: ENSG00000286148 (+69252)

30927-005.R1 (paired) (Report)

Host chromosome	Unaligned ends virus	Broken reads host	Disrupted genes	Nearby genes
21	N/A	26	gene: ENSG00000280441	gene: ENSG00000280441, MIR6724-3 (-10885), gene: ENSG00000274868 (-10351), gene: ENSG00000280614 (-4997), RNA5-8SN3 (-3579), gene: ENSG00000286267 (+18418), gene: ENSG00000286146 (+21336), gene: ENSG00000286054 (+22544), gene: ENSG00000286178 (+24421), MIR6724-4 (+33191), gene: ENSG00000278775 (+33746), gene: ENSG00000281181 (+38290), RNA5-8SN1 (+40484), gene: ENSG00000286149 (+62981), gene: ENSG00000286032 (+65915), gene: ENSG00000286091 (+67127), gene: ENSG00000286148 (+68981)

30927-005.R1 (paired) (Report)

Host chromosome	Unaligned ends virus	Broken reads host	Disrupted genes	Nearby genes
21	6,702	24	gene: ENSG00000280441	gene: ENSG00000280441, MIR6724-3 (-10899), gene: ENSG00000274868 (-10365), gene: ENSG00000280614 (-5011), RNA5-8SN3 (-3593), gene: ENSG00000286267 (+18404), gene: ENSG00000286146 (+21322), gene: ENSG00000286054 (+22530), gene: ENSG00000286178 (+24407), MIR6724-4 (+33177), gene: ENSG00000278775 (+33732), gene: ENSG00000281181 (+38276), RNA5-8SN1 (+40470), gene: ENSG00000286149 (+62967), gene: ENSG00000286032 (+65901), gene: ENSG00000286091 (+67113), gene: ENSG00000286148 (+68967)

Table S4 Viral integration sites reports

30927-005.R1 (paired) (Report)

Host chromosome	Unaligned ends virus	Broken reads host	Disrupted genes	Nearby genes
21	N/A	14	gene: ENSG00000280441	gene: ENSG00000280441, MIR6724-3 (-10988), gene: ENSG00000274868 (-10454), gene: ENSG00000280614 (-5100), RNA5-8SN3 (-3682), gene: ENSG00000286267 (+18315), gene: ENSG00000286146 (+21233), gene: ENSG00000286054 (+22441), gene: ENSG00000286178 (+24318), MIR6724-4 (+33088), gene: ENSG00000278775 (+33643), gene: ENSG00000281181 (+38187), RNA5-8SN1 (+40381), gene: ENSG00000286149 (+62878), gene: ENSG00000286032 (+65812), gene: ENSG00000286091 (+67024), gene: ENSG00000286148 (+68878)

30927-005.R1 (paired) (Report)

Host chromosome	Unaligned ends virus	Broken reads host	Disrupted genes	Nearby genes
21	N/A	14	gene: ENSG00000280441	gene: ENSG00000280441, MIR6724-3 (-11282), gene: ENSG00000274868 (-10748), gene: ENSG00000280614 (-5394), RNA5-8SN3 (-3976), gene: ENSG00000286267 (+18021), gene: ENSG00000286146 (+20939), gene: ENSG00000286054 (+22147), gene: ENSG00000286178 (+24024), MIR6724-4 (+32794), gene: ENSG00000278775 (+33349), gene: ENSG00000281181 (+37893), RNA5-8SN1 (+40087), gene: ENSG00000286149 (+62584), gene: ENSG00000286032 (+65518), gene: ENSG00000286091 (+66730), gene: ENSG00000286148 (+68584)

30927-005.R1 (paired) (Report)

Host chromosome	Unaligned ends virus	Broken reads host	Disrupted genes	Nearby genes
21	N/A	13	gene: ENSG00000280441	gene: ENSG00000280441, MIR6724-3 (-11352), gene: ENSG00000274868 (-10818), gene: ENSG00000280614 (-5464), RNA5-8SN3 (-4046), gene: ENSG00000286267 (+17951), gene: ENSG00000286146 (+20869), gene: ENSG00000286054 (+22077), gene: ENSG00000286178 (+23954), MIR6724-4 (+32724), gene: ENSG00000278775 (+33279), gene: ENSG00000281181 (+37823), RNA5-8SN1 (+40017), gene: ENSG00000286149 (+62514), gene: ENSG00000286032 (+65448), gene: ENSG00000286091 (+66660), gene: ENSG00000286148 (+68514)

30927-005.R1 (paired) (Report)

Host chromosome	Unaligned ends virus	Broken reads host	Disrupted genes	Nearby genes
21	3,489	10	gene: ENSG00000280441	gene: ENSG00000280441, MIR6724-3 (-12460), gene: ENSG00000274868 (-11926), gene: ENSG00000280614 (-6572), RNA5-8SN3 (-5154), gene: ENSG00000286267 (+16843), gene: ENSG00000286146 (+19761), gene: ENSG00000286054 (+20969), gene: ENSG00000286178 (+22846), MIR6724-4 (+31616), gene: ENSG00000278775 (+32171), gene: ENSG00000281181 (+36715), RNA5-8SN1 (+38909), gene: ENSG00000286149 (+61406), gene: ENSG00000286032 (+64340), gene: ENSG00000286091 (+65552), gene: ENSG00000286148 (+67406)

30927-005.R1 (paired) (Report)

Host chromosome	Unaligned ends virus	Broken reads host	Disrupted genes	Nearby genes
21	92	24	gene: ENSG00000280441	gene: ENSG00000280441, MIR6724-3 (-13101), gene: ENSG00000274868 (-12567), gene: ENSG00000280614 (-7213), RNA5-8SN3 (-5795), gene: ENSG00000286267 (+16202), gene: ENSG00000286146 (+19120), gene: ENSG00000286054 (+20328), gene: ENSG00000286178 (+22205), MIR6724-4 (+30975), gene: ENSG00000278775 (+31530), gene: ENSG00000281181 (+36074), RNA5-8SN1 (+38268), gene: ENSG00000286149 (+60765), gene: ENSG00000286032 (+63699), gene: ENSG00000286091 (+64911), gene: ENSG00000286148 (+66765)

30927-005.R1 (paired) (Report)

Host chromosome	Unaligned ends virus	Broken reads host	Disrupted genes	Nearby genes
21	N/A	22	gene: ENSG00000280441	gene: ENSG00000280441, MIR6724-3 (-13107), gene: ENSG00000274868 (-12573), gene: ENSG00000280614 (-7219), RNA5-8SN3 (-5801), gene: ENSG00000286267 (+16196), gene: ENSG00000286146 (+19114), gene: ENSG00000286054 (+20322), gene: ENSG00000286178 (+22199), MIR6724-4 (+30969), gene: ENSG00000278775 (+31524), gene: ENSG00000281181 (+36068), RNA5-8SN1 (+38262), gene: ENSG00000286149 (+60759), gene: ENSG00000286032 (+63693), gene: ENSG00000286091 (+64905), gene: ENSG00000286148 (+66759)

30927-005.R1 (paired) (Report)

Host chromosome	Unaligned ends virus	Broken reads host	Disrupted genes	Nearby genes
21	N/A	22	gene: ENSG00000280441	gene: ENSG00000280441, MIR6724-3 (-13134), gene: ENSG00000274868 (-12600), gene: ENSG00000280614 (-7246), RNA5-8SN3 (-5828), gene: ENSG00000286267 (+16169), gene: ENSG00000286146 (+19087), gene: ENSG00000286054 (+20295), gene: ENSG00000286178 (+22172), MIR6724-4 (+30942), gene: ENSG00000278775 (+31497), gene: ENSG00000281181 (+36041), RNA5-8SN1 (+38235), gene: ENSG00000286149 (+60732), gene: ENSG00000286032 (+63666), gene: ENSG00000286091 (+64878), gene: ENSG00000286148 (+66732)

30927-005.R1 (paired) (Report)

Host chromosome	Unaligned ends virus	Broken reads host	Disrupted genes	Nearby genes
21	N/A	20	gene: ENSG00000280441	gene: ENSG00000280441, MIR6724-3 (-13157), gene: ENSG00000274868 (-12623), gene: ENSG00000280614 (-7269), RNA5-8SN3 (-5851), gene: ENSG00000286267 (+16146), gene: ENSG00000286146 (+19064), gene: ENSG00000286054 (+20272), gene: ENSG00000286178 (+22149), MIR6724-4 (+30919), gene: ENSG00000278775 (+31474), gene: ENSG00000281181 (+36018), RNA5-8SN1 (+38212), gene: ENSG00000286149 (+60709), gene: ENSG00000286032 (+63643), gene: ENSG00000286091 (+64855), gene: ENSG00000286148 (+66709)

30927-005.R1 (paired) (Report)

Host chromosome	Unaligned ends virus	Broken reads host	Disrupted genes	Nearby genes
21	N/A	10	gene: ENSG00000280441	gene: ENSG00000280441, MIR6724-3 (-13216), gene: ENSG00000274868 (-12682), gene: ENSG00000280614 (-7328), RNA5-8SN3 (-5910), gene: ENSG00000286267 (+16087), gene: ENSG00000286146 (+19005), gene: ENSG00000286054 (+20213), gene: ENSG00000286178 (+22090), MIR6724-4 (+30860), gene: ENSG00000278775 (+31415), gene: ENSG00000281181 (+35959), RNA5-8SN1 (+38153), gene: ENSG00000286149 (+60650), gene: ENSG00000286032 (+63584), gene: ENSG00000286091 (+64796), gene: ENSG00000286148 (+66650)

30927-005.R1 (paired) (Report)

Host chromosome	Unaligned ends virus	Broken reads host	Disrupted genes	Nearby genes
21	N/A	10	gene: ENSG00000280441	gene: ENSG00000280441, MIR6724-3 (-13221), gene: ENSG00000274868 (-12687), gene: ENSG00000280614 (-7333), RNA5-8SN3 (-5915), gene: ENSG00000286267 (+16082), gene: ENSG00000286146 (+19000), gene: ENSG00000286054 (+20208), gene: ENSG00000286178 (+22085), MIR6724-4 (+30855), gene: ENSG00000278775 (+31410), gene: ENSG00000281181 (+35954), RNA5-8SN1 (+38148), gene: ENSG00000286149 (+60645), gene: ENSG00000286032 (+63579), gene: ENSG00000286091 (+64791), gene: ENSG00000286148 (+66645)

30927-005.R1 (paired) (Report)

Host chromosome	Unaligned ends virus	Broken reads host	Disrupted genes	Nearby genes
21	590	9	gene: ENSG00000280441	gene: ENSG00000280441, MIR6724-3 (-13222), gene: ENSG00000274868 (-12688), gene: ENSG00000280614 (-7334), RNA5-8SN3 (-5916), gene: ENSG00000286267 (+16081), gene: ENSG00000286146 (+18999), gene: ENSG00000286054 (+20207), gene: ENSG00000286178 (+22084), MIR6724-4 (+30854), gene: ENSG00000278775 (+31409), gene: ENSG00000281181 (+35953), RNA5-8SN1 (+38147), gene: ENSG00000286149 (+60644), gene: ENSG00000286032 (+63578), gene: ENSG00000286091 (+64790), gene: ENSG00000286148 (+66644)

30927-005.R1 (paired) (Report)

Host chromosome	Unaligned ends virus	Broken reads host	Disrupted genes	Nearby genes
21	3,489	6	gene: ENSG00000280441	gene: ENSG00000280441, MIR6724-3 (-13244), gene: ENSG00000274868 (-12710), gene: ENSG00000280614 (-7356), RNA5-8SN3 (-5938), gene: ENSG00000286267 (+16059), gene: ENSG00000286146 (+18977), gene: ENSG00000286054 (+20185), gene: ENSG00000286178 (+22062), MIR6724-4 (+30832), gene: ENSG00000278775 (+31387), gene: ENSG00000281181 (+35931), RNA5-8SN1 (+38125), gene: ENSG00000286149 (+60622), gene: ENSG00000286032 (+63556), gene: ENSG00000286091 (+64768), gene: ENSG00000286148 (+66622)

30927-005.R1 (paired) (Report)

Host chromosome	Unaligned ends virus	Broken reads host	Disrupted genes	Nearby genes
21	N/A	6	gene: ENSG00000280441	gene: ENSG00000280441, MIR6724-3 (-13246), gene: ENSG00000274868 (-12712), gene: ENSG00000280614 (-7358), RNA5-8SN3 (-5940), gene: ENSG00000286267 (+16057), gene: ENSG00000286146 (+18975), gene: ENSG00000286054 (+20183), gene: ENSG00000286178 (+22060), MIR6724-4 (+30830), gene: ENSG00000278775 (+31385), gene: ENSG00000281181 (+35929), RNA5-8SN1 (+38123), gene: ENSG00000286149 (+60620), gene: ENSG00000286032 (+63554), gene: ENSG00000286091 (+64766), gene: ENSG00000286148 (+66620)

30927-005.R1 (paired) (Report)

Host chromosome	Unaligned ends virus	Broken reads host	Disrupted genes	Nearby genes
21	7,291	3	gene: ENSG00000280441	gene: ENSG00000280441, MIR6724-3 (-13255), gene: ENSG00000274868 (-12721), gene: ENSG00000280614 (-7367), RNA5-8SN3 (-5949), gene: ENSG00000286267 (+16048), gene: ENSG00000286146 (+18966), gene: ENSG00000286054 (+20174), gene: ENSG00000286178 (+22051), MIR6724-4 (+30821), gene: ENSG00000278775 (+31376), gene: ENSG00000281181 (+35920), RNA5-8SN1 (+38114), gene: ENSG00000286149 (+60611), gene: ENSG00000286032 (+63545), gene: ENSG00000286091 (+64757), gene: ENSG00000286148 (+66611)

30927-005.R1 (paired) (Report)

Host chromosome	Unaligned ends virus	Broken reads host	Disrupted genes	Nearby genes
21	N/A	1	gene: ENSG00000280441	gene: ENSG00000280441, MIR6724-3 (-13265), gene: ENSG00000274868 (-12731), gene: ENSG00000280614 (-7377), RNA5-8SN3 (-5959), gene: ENSG00000286267 (+16038), gene: ENSG00000286146 (+18956), gene: ENSG00000286054 (+20164), gene: ENSG00000286178 (+22041), MIR6724-4 (+30811), gene: ENSG00000278775 (+31366), gene: ENSG00000281181 (+35910), RNA5-8SN1 (+38104), gene: ENSG00000286149 (+60601), gene: ENSG00000286032 (+63535), gene: ENSG00000286091 (+64747), gene: ENSG00000286148 (+66601)

30927-005.R1 (paired) (Report)

Host chromosome	Unaligned ends virus	Broken reads host	Disrupted genes	Nearby genes
21	N/A	1	gene: ENSG00000280441	gene: ENSG00000280441, MIR6724-3 (-13268), gene: ENSG00000274868 (-12734), gene: ENSG00000280614 (-7380), RNA5-8SN3 (-5962), gene: ENSG00000286267 (+16035), gene: ENSG00000286146 (+18953), gene: ENSG00000286054 (+20161), gene: ENSG00000286178 (+22038), MIR6724-4 (+30808), gene: ENSG00000278775 (+31363), gene: ENSG00000281181 (+35907), RNA5-8SN1 (+38101), gene: ENSG00000286149 (+60598), gene: ENSG00000286032 (+63532), gene: ENSG00000286091 (+64744), gene: ENSG00000286148 (+66598)

30927-005.R1 (paired) (Report)

Host chromosome	Unaligned ends virus	Broken reads host	Disrupted genes	Nearby genes
21	N/A	1	gene: ENSG00000280441	gene: ENSG00000280441, MIR6724-3 (-13272), gene: ENSG00000274868 (-12738), gene: ENSG00000280614 (-7384), RNA5-8SN3 (-5966), gene: ENSG00000286267 (+16031), gene: ENSG00000286146 (+18949), gene: ENSG00000286054 (+20157), gene: ENSG00000286178 (+22034), MIR6724-4 (+30804), gene: ENSG00000278775 (+31359), gene: ENSG00000281181 (+35903), RNA5-8SN1 (+38097), gene: ENSG00000286149 (+60594), gene: ENSG00000286032 (+63528), gene: ENSG00000286091 (+64740), gene: ENSG00000286148 (+66594)

30927-005.R1 (paired) (Report)

Host chromosome	Unaligned ends virus	Broken reads host	Disrupted genes	Nearby genes
21	N/A	1	gene: ENSG00000280441	gene: ENSG00000280441, MIR6724-3 (-13283), gene: ENSG00000274868 (-12749), gene: ENSG00000280614 (-7395), RNA5-8SN3 (-5977), gene: ENSG00000286267 (+16020), gene: ENSG00000286146 (+18938), gene: ENSG00000286054 (+20146), gene: ENSG00000286178 (+22023), MIR6724-4 (+30793), gene: ENSG00000278775 (+31348), gene: ENSG00000281181 (+35892), RNA5-8SN1 (+38086), gene: ENSG00000286149 (+60583), gene: ENSG00000286032 (+63517), gene: ENSG00000286091 (+64729), gene: ENSG00000286148 (+66583)

30927-005.R1 (paired) (Report)

Host chromosome	Unaligned ends virus	Broken reads host	Disrupted genes	Nearby genes
21	N/A	48	gene: ENSG00000280441	gene: ENSG00000280441, MIR6724-3 (-53846), gene: ENSG00000274868 (-53312), gene: ENSG00000280614 (-47958), RNA5-8SN3 (-46540), gene: ENSG00000286267 (-24064), gene: ENSG00000286146 (-21296), gene: ENSG00000286054 (-20183), gene: ENSG00000286178 (-18228), MIR6724-4 (-9678), gene: ENSG00000278775 (-9125), gene: ENSG00000281181 (-3748), RNA5-8SN1 (-2324), gene: ENSG00000286149 (+20020), gene: ENSG00000286032 (+22954), gene: ENSG00000286091 (+24166), gene: ENSG00000286148 (+26020)

30927-005.R1 (paired) (Report)

Host chromosome	Unaligned ends virus	Broken reads host	Disrupted genes	Nearby genes
21	N/A	7	gene: ENSG00000278996	gene: ENSG00000278996, MIR6724-1 (-4596), gene: ENSG00000275664 (-4062), MIR3648-1 (-1350), gene: ENSG00000277437 (-1098), gene: ENSG00000280800 (+381), RNA5-8SN2 (+2569), gene: ENSG00000286155 (+25351), gene: ENSG00000286252 (+27646), gene: ENSG00000286057 (+28856), gene: ENSG00000286012 (+30733), MIR6724-2 (+39502), gene: ENSG00000277671 (+40057), gene: ENSG00000281383 (+44589), 5_8S_rRNA (+46778)
21	92	19	gene: ENSG00000278996, gene: ENSG00000280800	gene: ENSG00000278996, MIR6724-1 (-5619), gene: ENSG00000275664 (-5085), MIR3648-1 (-2373), gene: ENSG00000277437 (-2121), gene: ENSG00000280800, RNA5-8SN2 (+1546), gene: ENSG00000286155 (+24328), gene: ENSG00000286252 (+26623), gene: ENSG00000286057 (+27833), gene: ENSG00000286012 (+29710), MIR6724-2 (+38479), gene: ENSG00000277671 (+39034), gene: ENSG00000281383 (+43566), 5_8S_rRNA (+45755)

30927-005.R1 (paired) (Report)

Host chromosome	Unaligned ends virus	Broken reads host	Disrupted genes	Nearby genes
21	N/A	13	gene: ENSG00000278996, gene: ENSG00000280800	gene: ENSG00000278996, MIR6724-1 (-5672), gene: ENSG00000275664 (-5138), MIR3648-1 (-2426), gene: ENSG00000277437 (-2174), gene: ENSG00000280800, RNA5-8SN2 (+1493), gene: ENSG00000286155 (+24275), gene: ENSG00000286252 (+26570), gene: ENSG00000286057 (+27780), gene: ENSG00000286012 (+29657), MIR6724-2 (+38426), gene: ENSG00000277671 (+38981), gene: ENSG00000281383 (+43513), 5_8S_rRNA (+45702)
21	4,496	9	gene: ENSG00000278996, gene: ENSG00000280800	gene: ENSG00000278996, MIR6724-1 (-5694), gene: ENSG00000275664 (-5160), MIR3648-1 (-2448), gene: ENSG00000277437 (-2196), gene: ENSG00000280800, RNA5-8SN2 (+1471), gene: ENSG00000286155 (+24253), gene: ENSG00000286252 (+26548), gene: ENSG00000286057 (+27758), gene: ENSG00000286012 (+29635), MIR6724-2 (+38404), gene: ENSG00000277671 (+38959), gene: ENSG00000281383 (+43491), 5_8S_rRNA (+45680)

30927-005.R1 (paired) (Report)

Host chromosome	Unaligned ends virus	Broken reads host	Disrupted genes	Nearby genes
21	N/A	28	gene: ENSG00000280441	gene: ENSG00000280441, MIR6724-3 (-54485), gene: ENSG00000274868 (-53951), gene: ENSG00000280614 (-48597), RNA5-8SN3 (-47179), gene: ENSG00000286267 (-24703), gene: ENSG00000286146 (-21935), gene: ENSG00000286054 (-20822), gene: ENSG00000286178 (-18867), MIR6724-4 (-10317), gene: ENSG00000278775 (-9764), gene: ENSG00000281181 (-4387), RNA5-8SN1 (-2963), gene: ENSG00000286149 (+19381), gene: ENSG00000286032 (+22315), gene: ENSG00000286091 (+23527), gene: ENSG00000286148 (+25381)

30927-005.R1 (paired) (Report)

Host chromosome	Unaligned ends virus	Broken reads host	Disrupted genes	Nearby genes
21	N/A	25	gene: ENSG00000278996	gene: ENSG00000278996, MIR6724-1 (-8576), gene: ENSG00000275664 (-8042), MIR3648-1 (-5330), gene: ENSG00000277437 (-5078), gene: ENSG00000280800 (-2676), RNA5-8SN2 (-1258), gene: ENSG00000286155 (+21371), gene: ENSG00000286252 (+23666), gene: ENSG00000286057 (+24876), gene: ENSG00000286012 (+26753), MIR6724-2 (+35522), gene: ENSG00000277671 (+36077), gene: ENSG00000281383 (+40609), 5_8S_rRNA (+42798)
21	N/A	18	gene: ENSG00000278996	gene: ENSG00000278996, MIR6724-1 (-8602), gene: ENSG00000275664 (-8068), MIR3648-1 (-5356), gene: ENSG00000277437 (-5104), gene: ENSG00000280800 (-2702), RNA5-8SN2 (-1284), gene: ENSG00000286155 (+21345), gene: ENSG00000286252 (+23640), gene: ENSG00000286057 (+24850), gene: ENSG00000286012 (+26727), MIR6724-2 (+35496), gene: ENSG00000277671 (+36051), gene: ENSG00000281383 (+40583), 5_8S_rRNA (+42772)

30927-005.R1 (paired) (Report)

Host chromosome	Unaligned ends virus	Broken reads host	Disrupted genes	Nearby genes
21	N/A	16	gene: ENSG00000278996	gene: ENSG00000278996, MIR6724-1 (-8903), gene: ENSG00000275664 (-8369), MIR3648-1 (-5657), gene: ENSG00000277437 (-5405), gene: ENSG00000280800 (-3003), RNA5-8SN2 (-1585), gene: ENSG00000286155 (+21044), gene: ENSG00000286252 (+23339), gene: ENSG00000286057 (+24549), gene: ENSG00000286012 (+26426), MIR6724-2 (+35195), gene: ENSG00000277671 (+35750), gene: ENSG00000281383 (+40282), 5_8S_rRNA (+42471)
21	5,000	14	gene: ENSG00000278996	gene: ENSG00000278996, MIR6724-1 (-8914), gene: ENSG00000275664 (-8380), MIR3648-1 (-5668), gene: ENSG00000277437 (-5416), gene: ENSG00000280800 (-3014), RNA5-8SN2 (-1596), gene: ENSG00000286155 (+21033), gene: ENSG00000286252 (+23328), gene: ENSG00000286057 (+24538), gene: ENSG00000286012 (+26415), MIR6724-2 (+35184), gene: ENSG00000277671 (+35739), gene: ENSG00000281383 (+40271), 5_8S_rRNA (+42460)

30927-005.R1 (paired) (Report)

Host chromosome	Unaligned ends virus	Broken reads host	Disrupted genes	Nearby genes
21	N/A	36	gene: ENSG00000278996	gene: ENSG00000278996, MIR6724-1 (-9803), gene: ENSG00000275664 (-9269), MIR3648-1 (-6557), gene: ENSG00000277437 (-6305), gene: ENSG00000280800 (-3903), RNA5-8SN2 (-2485), gene: ENSG00000286155 (+20144), gene: ENSG00000286252 (+22439), gene: ENSG00000286057 (+23649), gene: ENSG00000286012 (+25526), MIR6724-2 (+34295), gene: ENSG00000277671 (+34850), gene: ENSG00000281383 (+39382), 5_8S_rRNA (+41571)
21	3,510	36	gene: ENSG00000278996	gene: ENSG00000278996, MIR6724-1 (-9805), gene: ENSG00000275664 (-9271), MIR3648-1 (-6559), gene: ENSG00000277437 (-6307), gene: ENSG00000280800 (-3905), RNA5-8SN2 (-2487), gene: ENSG00000286155 (+20142), gene: ENSG00000286252 (+22437), gene: ENSG00000286057 (+23647), gene: ENSG00000286012 (+25524), MIR6724-2 (+34293), gene: ENSG00000277671 (+34848), gene: ENSG00000281383 (+39380), 5_8S_rRNA (+41569)

30927-005.R1 (paired) (Report)

Host chromosome	Unaligned ends virus	Broken reads host	Disrupted genes	Nearby genes
21	N/A	33	gene: ENSG00000278996	gene: ENSG00000278996, MIR6724-1 (-9814), gene: ENSG00000275664 (-9280), MIR3648-1 (-6568), gene: ENSG00000277437 (-6316), gene: ENSG00000280800 (-3914), RNA5-8SN2 (-2496), gene: ENSG00000286155 (+20133), gene: ENSG00000286252 (+22428), gene: ENSG00000286057 (+23638), gene: ENSG00000286012 (+25515), MIR6724-2 (+34284), gene: ENSG00000277671 (+34839), gene: ENSG00000281383 (+39371), 5_8S_rRNA (+41560)
21	2,822	32	gene: ENSG00000278996	gene: ENSG00000278996, MIR6724-1 (-9831), gene: ENSG00000275664 (-9297), MIR3648-1 (-6585), gene: ENSG00000277437 (-6333), gene: ENSG00000280800 (-3931), RNA5-8SN2 (-2513), gene: ENSG00000286155 (+20116), gene: ENSG00000286252 (+22411), gene: ENSG00000286057 (+23621), gene: ENSG00000286012 (+25498), MIR6724-2 (+34267), gene: ENSG00000277671 (+34822), gene: ENSG00000281383 (+39354), 5_8S_rRNA (+41543)

30927-005.R1 (paired) (Report)

Host chromosome	Unaligned ends virus	Broken reads host	Disrupted genes	Nearby genes
21	N/A	29	gene: ENSG00000278996	gene: ENSG00000278996, MIR6724-1 (-9838), gene: ENSG00000275664 (-9304), MIR3648-1 (-6592), gene: ENSG00000277437 (-6340), gene: ENSG00000280800 (-3938), RNA5-8SN2 (-2520), gene: ENSG00000286155 (+20109), gene: ENSG00000286252 (+22404), gene: ENSG00000286057 (+23614), gene: ENSG00000286012 (+25491), MIR6724-2 (+34260), gene: ENSG00000277671 (+34815), gene: ENSG00000281383 (+39347), 5_8S_rRNA (+41536)
21	N/A	27	gene: ENSG00000278996	gene: ENSG00000278996, MIR6724-1 (-9848), gene: ENSG00000275664 (-9314), MIR3648-1 (-6602), gene: ENSG00000277437 (-6350), gene: ENSG00000280800 (-3948), RNA5-8SN2 (-2530), gene: ENSG00000286155 (+20099), gene: ENSG00000286252 (+22394), gene: ENSG00000286057 (+23604), gene: ENSG00000286012 (+25481), MIR6724-2 (+34250), gene: ENSG00000277671 (+34805), gene: ENSG00000281383 (+39337), 5_8S_rRNA (+41526)

30927-005.R1 (paired) (Report)

Host chromosome	Unaligned ends virus	Broken reads host	Disrupted genes	Nearby genes
21	4,496	26	gene: ENSG00000278996	gene: ENSG00000278996, MIR6724-1 (-9849), gene: ENSG00000275664 (-9315), MIR3648-1 (-6603), gene: ENSG00000277437 (-6351), gene: ENSG00000280800 (-3949), RNA5-8SN2 (-2531), gene: ENSG00000286155 (+20098), gene: ENSG00000286252 (+22393), gene: ENSG00000286057 (+23603), gene: ENSG00000286012 (+25480), MIR6724-2 (+34249), gene: ENSG00000277671 (+34804), gene: ENSG00000281383 (+39336), 5_8S_rRNA (+41525)
21	N/A	25	gene: ENSG00000278996	gene: ENSG00000278996, MIR6724-1 (-9868), gene: ENSG00000275664 (-9334), MIR3648-1 (-6622), gene: ENSG00000277437 (-6370), gene: ENSG00000280800 (-3968), RNA5-8SN2 (-2550), gene: ENSG00000286155 (+20079), gene: ENSG00000286252 (+22374), gene: ENSG00000286057 (+23584), gene: ENSG00000286012 (+25461), MIR6724-2 (+34230), gene: ENSG00000277671 (+34785), gene: ENSG00000281383 (+39317), 5_8S_rRNA (+41506)

30927-005.R1 (paired) (Report)

Host chromosome	Unaligned ends virus	Broken reads host	Disrupted genes	Nearby genes
21	N/A	23	gene: ENSG00000278996	gene: ENSG00000278996, MIR6724-1 (-9874), gene: ENSG00000275664 (-9340), MIR3648-1 (-6628), gene: ENSG00000277437 (-6376), gene: ENSG00000280800 (-3974), RNA5-8SN2 (-2556), gene: ENSG00000286155 (+20073), gene: ENSG00000286252 (+22368), gene: ENSG00000286057 (+23578), gene: ENSG00000286012 (+25455), MIR6724-2 (+34224), gene: ENSG00000277671 (+34779), gene: ENSG00000281383 (+39311), 5_8S_rRNA (+41500)
21	60,930	17	gene: ENSG00000278996	gene: ENSG00000278996, MIR6724-1 (-9969), gene: ENSG00000275664 (-9435), MIR3648-1 (-6723), gene: ENSG00000277437 (-6471), gene: ENSG00000280800 (-4069), RNA5-8SN2 (-2651), gene: ENSG00000286155 (+19978), gene: ENSG00000286252 (+22273), gene: ENSG00000286057 (+23483), gene: ENSG00000286012 (+25360), MIR6724-2 (+34129), gene: ENSG00000277671 (+34684), gene: ENSG00000281383 (+39216), 5_8S_rRNA (+41405)

30927-005.R1 (paired) (Report)

Host chromosome	Unaligned ends virus	Broken reads host	Disrupted genes	Nearby genes
21	N/A	17	gene: ENSG00000278996	gene: ENSG00000278996, MIR6724-1 (-9974), gene: ENSG00000275664 (-9440), MIR3648-1 (-6728), gene: ENSG00000277437 (-6476), gene: ENSG00000280800 (-4074), RNA5-8SN2 (-2656), gene: ENSG00000286155 (+19973), gene: ENSG00000286252 (+22268), gene: ENSG00000286057 (+23478), gene: ENSG00000286012 (+25355), MIR6724-2 (+34124), gene: ENSG00000277671 (+34679), gene: ENSG00000281383 (+39211), 5_8S_rRNA (+41400)
21	N/A	15	gene: ENSG00000278996	gene: ENSG00000278996, MIR6724-1 (-9994), gene: ENSG00000275664 (-9460), MIR3648-1 (-6748), gene: ENSG00000277437 (-6496), gene: ENSG00000280800 (-4094), RNA5-8SN2 (-2676), gene: ENSG00000286155 (+19953), gene: ENSG00000286252 (+22248), gene: ENSG00000286057 (+23458), gene: ENSG00000286012 (+25335), MIR6724-2 (+34104), gene: ENSG00000277671 (+34659), gene: ENSG00000281383 (+39191), 5_8S_rRNA (+41380)

30927-005.R1 (paired) (Report)

Host chromosome	Unaligned ends virus	Broken reads host	Disrupted genes	Nearby genes
21	4,358	15	gene: ENSG00000278996	gene: ENSG00000278996, MIR6724-1 (-9995), gene: ENSG00000275664 (-9461), MIR3648-1 (-6749), gene: ENSG00000277437 (-6497), gene: ENSG00000280800 (-4095), RNA5-8SN2 (-2677), gene: ENSG00000286155 (+19952), gene: ENSG00000286252 (+22247), gene: ENSG00000286057 (+23457), gene: ENSG00000286012 (+25334), MIR6724-2 (+34103), gene: ENSG00000277671 (+34658), gene: ENSG00000281383 (+39190), 5_8S_rRNA (+41379)
21	N/A	14	gene: ENSG00000278996	gene: ENSG00000278996, MIR6724-1 (-9996), gene: ENSG00000275664 (-9462), MIR3648-1 (-6750), gene: ENSG00000277437 (-6498), gene: ENSG00000280800 (-4096), RNA5-8SN2 (-2678), gene: ENSG00000286155 (+19951), gene: ENSG00000286252 (+22246), gene: ENSG00000286057 (+23456), gene: ENSG00000286012 (+25333), MIR6724-2 (+34102), gene: ENSG00000277671 (+34657), gene: ENSG00000281383 (+39189), 5_8S_rRNA (+41378)

30927-005.R1 (paired) (Report)

Host chromosome	Unaligned ends virus	Broken reads host	Disrupted genes	Nearby genes
21	N/A	14	gene: ENSG00000278996	gene: ENSG00000278996, MIR6724-1 (-10003), gene: ENSG00000275664 (-9469), MIR3648-1 (-6757), gene: ENSG00000277437 (-6505), gene: ENSG00000280800 (-4103), RNA5-8SN2 (-2685), gene: ENSG00000286155 (+19944), gene: ENSG00000286252 (+22239), gene: ENSG00000286057 (+23449), gene: ENSG00000286012 (+25326), MIR6724-2 (+34095), gene: ENSG00000277671 (+34650), gene: ENSG00000281383 (+39182), 5_8S_rRNA (+41371)
21	N/A	8	gene: ENSG00000278996	gene: ENSG00000278996, MIR6724-1 (-10299), gene: ENSG00000275664 (-9765), MIR3648-1 (-7053), gene: ENSG00000277437 (-6801), gene: ENSG00000280800 (-4399), RNA5-8SN2 (-2981), gene: ENSG00000286155 (+19648), gene: ENSG00000286252 (+21943), gene: ENSG00000286057 (+23153), gene: ENSG00000286012 (+25030), MIR6724-2 (+33799), gene: ENSG00000277671 (+34354), gene: ENSG00000281383 (+38886), 5_8S_rRNA (+41075)

30927-005.R1 (paired) (Report)

Host chromosome	Unaligned ends virus	Broken reads host	Disrupted genes	Nearby genes
21	N/A	14	gene: ENSG00000278996	gene: ENSG00000278996, MIR6724-1 (-11001), gene: ENSG00000275664 (-10467), MIR3648-1 (-7755), gene: ENSG00000277437 (-7503), gene: ENSG00000280800 (-5101), RNA5-8SN2 (-3683), gene: ENSG00000286155 (+18946), gene: ENSG00000286252 (+21241), gene: ENSG00000286057 (+22451), gene: ENSG00000286012 (+24328), MIR6724-2 (+33097), gene: ENSG00000277671 (+33652), gene: ENSG00000281383 (+38184), 5_8S_rRNA (+40373)
21	4,358	49	gene: ENSG00000278996	gene: ENSG00000278996, MIR6724-1 (-13116), gene: ENSG00000275664 (-12582), MIR3648-1 (-9870), gene: ENSG00000277437 (-9618), gene: ENSG00000280800 (-7216), RNA5-8SN2 (-5798), gene: ENSG00000286155 (+16831), gene: ENSG00000286252 (+19126), gene: ENSG00000286057 (+20336), gene: ENSG00000286012 (+22213), MIR6724-2 (+30982), gene: ENSG00000277671 (+31537), gene: ENSG00000281383 (+36069), 5_8S_rRNA (+38258)

30927-005.R1 (paired) (Report)

Host chromosome	Unaligned ends virus	Broken reads host	Disrupted genes	Nearby genes
21	6,702	43	gene: ENSG00000278996	gene: ENSG00000278996, MIR6724-1 (-13143), gene: ENSG00000275664 (-12609), MIR3648-1 (-9897), gene: ENSG00000277437 (-9645), gene: ENSG00000280800 (-7243), RNA5-8SN2 (-5825), gene: ENSG00000286155 (+16804), gene: ENSG00000286252 (+19099), gene: ENSG00000286057 (+20309), gene: ENSG00000286012 (+22186), MIR6724-2 (+30955), gene: ENSG00000277671 (+31510), gene: ENSG00000281383 (+36042), 5_8S_rRNA (+38231)
21	3,551	40	gene: ENSG00000278996	gene: ENSG00000278996, MIR6724-1 (-13166), gene: ENSG00000275664 (-12632), MIR3648-1 (-9920), gene: ENSG00000277437 (-9668), gene: ENSG00000280800 (-7266), RNA5-8SN2 (-5848), gene: ENSG00000286155 (+16781), gene: ENSG00000286252 (+19076), gene: ENSG00000286057 (+20286), gene: ENSG00000286012 (+22163), MIR6724-2 (+30932), gene: ENSG00000277671 (+31487), gene: ENSG00000281383 (+36019), 5_8S_rRNA (+38208)

30927-005.R1 (paired) (Report)

Host chromosome	Unaligned ends virus	Broken reads host	Disrupted genes	Nearby genes
21	N/A	38	gene: ENSG00000278996	gene: ENSG00000278996, MIR6724-1 (-13172), gene: ENSG00000275664 (-12638), MIR3648-1 (-9926), gene: ENSG00000277437 (-9674), gene: ENSG00000280800 (-7272), RNA5-8SN2 (-5854), gene: ENSG00000286155 (+16775), gene: ENSG00000286252 (+19070), gene: ENSG00000286057 (+20280), gene: ENSG00000286012 (+22157), MIR6724-2 (+30926), gene: ENSG00000277671 (+31481), gene: ENSG00000281383 (+36013), 5_8S_rRNA (+38202)
21	N/A	37	gene: ENSG00000278996	gene: ENSG00000278996, MIR6724-1 (-13174), gene: ENSG00000275664 (-12640), MIR3648-1 (-9928), gene: ENSG00000277437 (-9676), gene: ENSG00000280800 (-7274), RNA5-8SN2 (-5856), gene: ENSG00000286155 (+16773), gene: ENSG00000286252 (+19068), gene: ENSG00000286057 (+20278), gene: ENSG00000286012 (+22155), MIR6724-2 (+30924), gene: ENSG00000277671 (+31479), gene: ENSG00000281383 (+36011), 5_8S_rRNA (+38200)

30927-005.R1 (paired) (Report)

Host chromosome	Unaligned ends virus	Broken reads host	Disrupted genes	Nearby genes
21	3,551	32	gene: ENSG00000278996	gene: ENSG00000278996, MIR6724-1 (-13184), gene: ENSG00000275664 (-12650), MIR3648-1 (-9938), gene: ENSG00000277437 (-9686), gene: ENSG00000280800 (-7284), RNA5-8SN2 (-5866), gene: ENSG00000286155 (+16763), gene: ENSG00000286252 (+19058), gene: ENSG00000286057 (+20268), gene: ENSG00000286012 (+22145), MIR6724-2 (+30914), gene: ENSG00000277671 (+31469), gene: ENSG00000281383 (+36001), 5_8S_rRNA (+38190)
21	1,930	32	gene: ENSG00000278996	gene: ENSG00000278996, MIR6724-1 (-13185), gene: ENSG00000275664 (-12651), MIR3648-1 (-9939), gene: ENSG00000277437 (-9687), gene: ENSG00000280800 (-7285), RNA5-8SN2 (-5867), gene: ENSG00000286155 (+16762), gene: ENSG00000286252 (+19057), gene: ENSG00000286057 (+20267), gene: ENSG00000286012 (+22144), MIR6724-2 (+30913), gene: ENSG00000277671 (+31468), gene: ENSG00000281383 (+36000), 5_8S_rRNA (+38189)

30927-005.R1 (paired) (Report)

Host chromosome	Unaligned ends virus	Broken reads host	Disrupted genes	Nearby genes
21	3,156	31	gene: ENSG00000278996	gene: ENSG00000278996, MIR6724-1 (-13186), gene: ENSG00000275664 (-12652), MIR3648-1 (-9940), gene: ENSG00000277437 (-9688), gene: ENSG00000280800 (-7286), RNA5-8SN2 (-5868), gene: ENSG00000286155 (+16761), gene: ENSG00000286252 (+19056), gene: ENSG00000286057 (+20266), gene: ENSG00000286012 (+22143), MIR6724-2 (+30912), gene: ENSG00000277671 (+31467), gene: ENSG00000281383 (+35999), 5_8S_rRNA (+38188)
21	4,427	21	gene: ENSG00000278996	gene: ENSG00000278996, MIR6724-1 (-13201), gene: ENSG00000275664 (-12667), MIR3648-1 (-9955), gene: ENSG00000277437 (-9703), gene: ENSG00000280800 (-7301), RNA5-8SN2 (-5883), gene: ENSG00000286155 (+16746), gene: ENSG00000286252 (+19041), gene: ENSG00000286057 (+20251), gene: ENSG00000286012 (+22128), MIR6724-2 (+30897), gene: ENSG00000277671 (+31452), gene: ENSG00000281383 (+35984), 5_8S_rRNA (+38173)

30927-005.R1 (paired) (Report)

Host chromosome	Unaligned ends virus	Broken reads host	Disrupted genes	Nearby genes
21	3,156	21	gene: ENSG00000278996	gene: ENSG00000278996, MIR6724-1 (-13202), gene: ENSG00000275664 (-12668), MIR3648-1 (-9956), gene: ENSG00000277437 (-9704), gene: ENSG00000280800 (-7302), RNA5-8SN2 (-5884), gene: ENSG00000286155 (+16745), gene: ENSG00000286252 (+19040), gene: ENSG00000286057 (+20250), gene: ENSG00000286012 (+22127), MIR6724-2 (+30896), gene: ENSG00000277671 (+31451), gene: ENSG00000281383 (+35983), 5_8S_rRNA (+38172)
21	N/A	21	gene: ENSG00000278996	gene: ENSG00000278996, MIR6724-1 (-13204), gene: ENSG00000275664 (-12670), MIR3648-1 (-9958), gene: ENSG00000277437 (-9706), gene: ENSG00000280800 (-7304), RNA5-8SN2 (-5886), gene: ENSG00000286155 (+16743), gene: ENSG00000286252 (+19038), gene: ENSG00000286057 (+20248), gene: ENSG00000286012 (+22125), MIR6724-2 (+30894), gene: ENSG00000277671 (+31449), gene: ENSG00000281383 (+35981), 5_8S_rRNA (+38170)

30927-005.R1 (paired) (Report)

Host chromosome	Unaligned ends virus	Broken reads host	Disrupted genes	Nearby genes
21	N/A	19	gene: ENSG00000278996	gene: ENSG00000278996, MIR6724-1 (-13211), gene: ENSG00000275664 (-12677), MIR3648-1 (-9965), gene: ENSG00000277437 (-9713), gene: ENSG00000280800 (-7311), RNA5-8SN2 (-5893), gene: ENSG00000286155 (+16736), gene: ENSG00000286252 (+19031), gene: ENSG00000286057 (+20241), gene: ENSG00000286012 (+22118), MIR6724-2 (+30887), gene: ENSG00000277671 (+31442), gene: ENSG00000281383 (+35974), 5_8S_rRNA (+38163)
21	N/A	15	gene: ENSG00000278996	gene: ENSG00000278996, MIR6724-1 (-13220), gene: ENSG00000275664 (-12686), MIR3648-1 (-9974), gene: ENSG00000277437 (-9722), gene: ENSG00000280800 (-7320), RNA5-8SN2 (-5902), gene: ENSG00000286155 (+16727), gene: ENSG00000286252 (+19022), gene: ENSG00000286057 (+20232), gene: ENSG00000286012 (+22109), MIR6724-2 (+30878), gene: ENSG00000277671 (+31433), gene: ENSG00000281383 (+35965), 5_8S_rRNA (+38154)

30927-005.R1 (paired) (Report)

Host chromosome	Unaligned ends virus	Broken reads host	Disrupted genes	Nearby genes
21	4,056	15	gene: ENSG00000278996	gene: ENSG00000278996, MIR6724-1 (-13221), gene: ENSG00000275664 (-12687), MIR3648-1 (-9975), gene: ENSG00000277437 (-9723), gene: ENSG00000280800 (-7321), RNA5-8SN2 (-5903), gene: ENSG00000286155 (+16726), gene: ENSG00000286252 (+19021), gene: ENSG00000286057 (+20231), gene: ENSG00000286012 (+22108), MIR6724-2 (+30877), gene: ENSG00000277671 (+31432), gene: ENSG00000281383 (+35964), 5_8S_rRNA (+38153)
21	N/A	13	gene: ENSG00000278996	gene: ENSG00000278996, MIR6724-1 (-13230), gene: ENSG00000275664 (-12696), MIR3648-1 (-9984), gene: ENSG00000277437 (-9732), gene: ENSG00000280800 (-7330), RNA5-8SN2 (-5912), gene: ENSG00000286155 (+16717), gene: ENSG00000286252 (+19012), gene: ENSG00000286057 (+20222), gene: ENSG00000286012 (+22099), MIR6724-2 (+30868), gene: ENSG00000277671 (+31423), gene: ENSG00000281383 (+35955), 5_8S_rRNA (+38144)

30927-005.R1 (paired) (Report)

Host chromosome	Unaligned ends virus	Broken reads host	Disrupted genes	Nearby genes
21	N/A	12	gene: ENSG00000278996	gene: ENSG00000278996, MIR6724-1 (-13231), gene: ENSG00000275664 (-12697), MIR3648-1 (-9985), gene: ENSG00000277437 (-9733), gene: ENSG00000280800 (-7331), RNA5-8SN2 (-5913), gene: ENSG00000286155 (+16716), gene: ENSG00000286252 (+19011), gene: ENSG00000286057 (+20221), gene: ENSG00000286012 (+22098), MIR6724-2 (+30867), gene: ENSG00000277671 (+31422), gene: ENSG00000281383 (+35954), 5_8S_rRNA (+38143)
21	N/A	12	gene: ENSG00000278996	gene: ENSG00000278996, MIR6724-1 (-13236), gene: ENSG00000275664 (-12702), MIR3648-1 (-9990), gene: ENSG00000277437 (-9738), gene: ENSG00000280800 (-7336), RNA5-8SN2 (-5918), gene: ENSG00000286155 (+16711), gene: ENSG00000286252 (+19006), gene: ENSG00000286057 (+20216), gene: ENSG00000286012 (+22093), MIR6724-2 (+30862), gene: ENSG00000277671 (+31417), gene: ENSG00000281383 (+35949), 5_8S_rRNA (+38138)

30927-005.R1 (paired) (Report)

Host chromosome	Unaligned ends virus	Broken reads host	Disrupted genes	Nearby genes
21	N/A	6	gene: ENSG00000278996	gene: ENSG00000278996, MIR6724-1 (-13246), gene: ENSG00000275664 (-12712), MIR3648-1 (-10000), gene: ENSG00000277437 (-9748), gene: ENSG00000280800 (-7346), RNA5-8SN2 (-5928), gene: ENSG00000286155 (+16701), gene: ENSG00000286252 (+18996), gene: ENSG00000286057 (+20206), gene: ENSG00000286012 (+22083), MIR6724-2 (+30852), gene: ENSG00000277671 (+31407), gene: ENSG00000281383 (+35939), 5_8S_rRNA (+38128)
21	N/A	5	gene: ENSG00000278996	gene: ENSG00000278996, MIR6724-1 (-13258), gene: ENSG00000275664 (-12724), MIR3648-1 (-10012), gene: ENSG00000277437 (-9760), gene: ENSG00000280800 (-7358), RNA5-8SN2 (-5940), gene: ENSG00000286155 (+16689), gene: ENSG00000286252 (+18984), gene: ENSG00000286057 (+20194), gene: ENSG00000286012 (+22071), MIR6724-2 (+30840), gene: ENSG00000277671 (+31395), gene: ENSG00000281383 (+35927), 5_8S_rRNA (+38116)

30927-005.R1 (paired) (Report)

Host chromosome	Unaligned ends virus	Broken reads host	Disrupted genes	Nearby genes
21	N/A	1	gene: ENSG00000278996	gene: ENSG00000278996, MIR6724-1 (-13280), gene: ENSG00000275664 (-12746), MIR3648-1 (-10034), gene: ENSG00000277437 (-9782), gene: ENSG00000280800 (-7380), RNA5-8SN2 (-5962), gene: ENSG00000286155 (+16667), gene: ENSG00000286252 (+18962), gene: ENSG00000286057 (+20172), gene: ENSG00000286012 (+22049), MIR6724-2 (+30818), gene: ENSG00000277671 (+31373), gene: ENSG00000281383 (+35905), 5_8S_rRNA (+38094)

30927-005.R1 (paired) (Report)

Host chromosome	Unaligned ends virus	Broken reads host	Disrupted genes	Nearby genes
21	N/A	34	gene: ENSG00000280441	gene: ENSG00000280441, MIR6724-3 (-54677), gene: ENSG00000274868 (-54143), gene: ENSG00000280614 (-48789), RNA5-8SN3 (-47371), gene: ENSG00000286267 (-24895), gene: ENSG00000286146 (-22127), gene: ENSG00000286054 (-21014), gene: ENSG00000286178 (-19059), MIR6724-4 (-10509), gene: ENSG00000278775 (-9956), gene: ENSG00000281181 (-4579), RNA5-8SN1 (-3155), gene: ENSG00000286149 (+19189), gene: ENSG00000286032 (+22123), gene: ENSG00000286091 (+23335), gene: ENSG00000286148 (+25189)

30927-005.R1 (paired) (Report)

Host chromosome	Unaligned ends virus	Broken reads host	Disrupted genes	Nearby genes
21	N/A	4	gene: ENSG00000280441	gene: ENSG00000280441, MIR6724-3 (-45602), gene: ENSG00000274868 (-45068), gene: ENSG00000280614 (-39714), RNA5-8SN3 (-38296), gene: ENSG00000286267 (-15820), gene: ENSG00000286146 (-13052), gene: ENSG00000286054 (-11939), gene: ENSG00000286178 (-9984), MIR6724-4 (-1434), gene: ENSG00000278775 (-881), gene: ENSG00000281181 (+3573), RNA5-8SN1 (+5767), gene: ENSG00000286149 (+28264), gene: ENSG00000286032 (+31198), gene: ENSG00000286091 (+32410), gene: ENSG00000286148 (+34264)

30927-005.R1 (paired) (Report)

Host chromosome	Unaligned ends virus	Broken reads host	Disrupted genes	Nearby genes
21	320	4	gene: ENSG00000280441	gene: ENSG00000280441, MIR6724-3 (-48661), gene: ENSG00000274868 (-48127), gene: ENSG00000280614 (-42773), RNA5-8SN3 (-41355), gene: ENSG00000286267 (-18879), gene: ENSG00000286146 (-16111), gene: ENSG00000286054 (-14998), gene: ENSG00000286178 (-13043), MIR6724-4 (-4493), gene: ENSG00000278775 (-3940), gene: ENSG00000281181 (+514), RNA5-8SN1 (+2708), gene: ENSG00000286149 (+25205), gene: ENSG00000286032 (+28139), gene: ENSG00000286091 (+29351), gene: ENSG00000286148 (+31205)

30927-005.R1 (paired) (Report)

Host chromosome	Unaligned ends virus	Broken reads host	Disrupted genes	Nearby genes
21	N/A	2	gene: ENSG00000280441	gene: ENSG00000280441, MIR6724-3 (-48725), gene: ENSG00000274868 (-48191), gene: ENSG00000280614 (-42837), RNA5-8SN3 (-41419), gene: ENSG00000286267 (-18943), gene: ENSG00000286146 (-16175), gene: ENSG00000286054 (-15062), gene: ENSG00000286178 (-13107), MIR6724-4 (-4557), gene: ENSG00000278775 (-4004), gene: ENSG00000281181 (+450), RNA5-8SN1 (+2644), gene: ENSG00000286149 (+25141), gene: ENSG00000286032 (+28075), gene: ENSG00000286091 (+29287), gene: ENSG00000286148 (+31141)

30927-005.R1 (paired) (Report)

Host chromosome	Unaligned ends virus	Broken reads host	Disrupted genes	Nearby genes
21	N/A	25	gene: ENSG00000280441, gene: ENSG00000281181	gene: ENSG00000280441, MIR6724-3 (-49783), gene: ENSG00000274868 (-49249), gene: ENSG00000280614 (-43895), RNA5-8SN3 (-42477), gene: ENSG00000286267 (-20001), gene: ENSG00000286146 (-17233), gene: ENSG00000286054 (-16120), gene: ENSG00000286178 (-14165), MIR6724-4 (-5615), gene: ENSG00000278775 (-5062), gene: ENSG00000281181, RNA5-8SN1 (+1586), gene: ENSG00000286149 (+24083), gene: ENSG00000286032 (+27017), gene: ENSG00000286091 (+28229), gene: ENSG00000286148 (+30083)

30927-005.R1 (paired) (Report)

Host chromosome	Unaligned ends virus	Broken reads host	Disrupted genes	Nearby genes
21	4,056	15	gene: ENSG00000280441, gene: ENSG00000281181	gene: ENSG00000280441, MIR6724-3 (-49845), gene: ENSG00000274868 (-49311), gene: ENSG00000280614 (-43957), RNA5-8SN3 (-42539), gene: ENSG00000286267 (-20063), gene: ENSG00000286146 (-17295), gene: ENSG00000286054 (-16182), gene: ENSG00000286178 (-14227), MIR6724-4 (-5677), gene: ENSG00000278775 (-5124), gene: ENSG00000281181, RNA5-8SN1 (+1524), gene: ENSG00000286149 (+24021), gene: ENSG00000286032 (+26955), gene: ENSG00000286091 (+28167), gene: ENSG00000286148 (+30021)

30927-005.R1 (paired) (Report)

Host chromosome	Unaligned ends virus	Broken reads host	Disrupted genes	Nearby genes
21	1,929	13	gene: ENSG00000280441, gene: ENSG00000281181	gene: ENSG00000280441, MIR6724-3 (-49870), gene: ENSG00000274868 (-49336), gene: ENSG00000280614 (-43982), RNA5-8SN3 (-42564), gene: ENSG00000286267 (-20088), gene: ENSG00000286146 (-17320), gene: ENSG00000286054 (-16207), gene: ENSG00000286178 (-14252), MIR6724-4 (-5702), gene: ENSG00000278775 (-5149), gene: ENSG00000281181, RNA5-8SN1 (+1499), gene: ENSG00000286149 (+23996), gene: ENSG00000286032 (+26930), gene: ENSG00000286091 (+28142), gene: ENSG00000286148 (+29996)

30927-005.R1 (paired) (Report)

Host chromosome	Unaligned ends virus	Broken reads host	Disrupted genes	Nearby genes
21	N/A	11	gene: ENSG00000280441, gene: ENSG00000281181	gene: ENSG00000280441, MIR6724-3 (-49892), gene: ENSG00000274868 (-49358), gene: ENSG00000280614 (-44004), RNA5-8SN3 (-42586), gene: ENSG00000286267 (-20110), gene: ENSG00000286146 (-17342), gene: ENSG00000286054 (-16229), gene: ENSG00000286178 (-14274), MIR6724-4 (-5724), gene: ENSG00000278775 (-5171), gene: ENSG00000281181, RNA5-8SN1 (+1477), gene: ENSG00000286149 (+23974), gene: ENSG00000286032 (+26908), gene: ENSG00000286091 (+28120), gene: ENSG00000286148 (+29974)

30927-005.R1 (paired) (Report)

Host chromosome	Unaligned ends virus	Broken reads host	Disrupted genes	Nearby genes
21	N/A	33	gene: ENSG00000280441	gene: ENSG00000280441, MIR6724-3 (-52702), gene: ENSG00000274868 (-52168), gene: ENSG00000280614 (-46814), RNA5-8SN3 (-45396), gene: ENSG00000286267 (-22920), gene: ENSG00000286146 (-20152), gene: ENSG00000286054 (-19039), gene: ENSG00000286178 (-17084), MIR6724-4 (-8534), gene: ENSG00000278775 (-7981), gene: ENSG00000281181 (-2604), RNA5-8SN1 (-1180), gene: ENSG00000286149 (+21164), gene: ENSG00000286032 (+24098), gene: ENSG00000286091 (+25310), gene: ENSG00000286148 (+27164)

30927-005.R1 (paired) (Report)

Host chromosome	Unaligned ends virus	Broken reads host	Disrupted genes	Nearby genes
21	N/A	32	gene: ENSG00000280441	gene: ENSG00000280441, MIR6724-3 (-52712), gene: ENSG00000274868 (-52178), gene: ENSG00000280614 (-46824), RNA5-8SN3 (-45406), gene: ENSG00000286267 (-22930), gene: ENSG00000286146 (-20162), gene: ENSG00000286054 (-19049), gene: ENSG00000286178 (-17094), MIR6724-4 (-8544), gene: ENSG00000278775 (-7991), gene: ENSG00000281181 (-2614), RNA5-8SN1 (-1190), gene: ENSG00000286149 (+21154), gene: ENSG00000286032 (+24088), gene: ENSG00000286091 (+25300), gene: ENSG00000286148 (+27154)

30927-005.R1 (paired) (Report)

Host chromosome	Unaligned ends virus	Broken reads host	Disrupted genes	Nearby genes
21	3,156	27	gene: ENSG00000280441	gene: ENSG00000280441, MIR6724-3 (-52743), gene: ENSG00000274868 (-52209), gene: ENSG00000280614 (-46855), RNA5-8SN3 (-45437), gene: ENSG00000286267 (-22961), gene: ENSG00000286146 (-20193), gene: ENSG00000286054 (-19080), gene: ENSG00000286178 (-17125), MIR6724-4 (-8575), gene: ENSG00000278775 (-8022), gene: ENSG00000281181 (-2645), RNA5-8SN1 (-1221), gene: ENSG00000286149 (+21123), gene: ENSG00000286032 (+24057), gene: ENSG00000286091 (+25269), gene: ENSG00000286148 (+27123)

30927-005.R1 (paired) (Report)

Host chromosome	Unaligned ends virus	Broken reads host	Disrupted genes	Nearby genes
21	N/A	27	gene: ENSG00000280441	gene: ENSG00000280441, MIR6724-3 (-52745), gene: ENSG00000274868 (-52211), gene: ENSG00000280614 (-46857), RNA5-8SN3 (-45439), gene: ENSG00000286267 (-22963), gene: ENSG00000286146 (-20195), gene: ENSG00000286054 (-19082), gene: ENSG00000286178 (-17127), MIR6724-4 (-8577), gene: ENSG00000278775 (-8024), gene: ENSG00000281181 (-2647), RNA5-8SN1 (-1223), gene: ENSG00000286149 (+21121), gene: ENSG00000286032 (+24055), gene: ENSG00000286091 (+25267), gene: ENSG00000286148 (+27121)

30927-005.R1 (paired) (Report)

Host chromosome	Unaligned ends virus	Broken reads host	Disrupted genes	Nearby genes
21	3,855	27	gene: ENSG00000280441	gene: ENSG00000280441, MIR6724-3 (-52748), gene: ENSG00000274868 (-52214), gene: ENSG00000280614 (-46860), RNA5-8SN3 (-45442), gene: ENSG00000286267 (-22966), gene: ENSG00000286146 (-20198), gene: ENSG00000286054 (-19085), gene: ENSG00000286178 (-17130), MIR6724-4 (-8580), gene: ENSG00000278775 (-8027), gene: ENSG00000281181 (-2650), RNA5-8SN1 (-1226), gene: ENSG00000286149 (+21118), gene: ENSG00000286032 (+24052), gene: ENSG00000286091 (+25264), gene: ENSG00000286148 (+27118)

30927-005.R1 (paired) (Report)

Host chromosome	Unaligned ends virus	Broken reads host	Disrupted genes	Nearby genes
21	N/A	21	gene: ENSG00000280441	gene: ENSG00000280441, MIR6724-3 (-52781), gene: ENSG00000274868 (-52247), gene: ENSG00000280614 (-46893), RNA5-8SN3 (-45475), gene: ENSG00000286267 (-22999), gene: ENSG00000286146 (-20231), gene: ENSG00000286054 (-19118), gene: ENSG00000286178 (-17163), MIR6724-4 (-8613), gene: ENSG00000278775 (-8060), gene: ENSG00000281181 (-2683), RNA5-8SN1 (-1259), gene: ENSG00000286149 (+21085), gene: ENSG00000286032 (+24019), gene: ENSG00000286091 (+25231), gene: ENSG00000286148 (+27085)

30927-005.R1 (paired) (Report)

Host chromosome	Unaligned ends virus	Broken reads host	Disrupted genes	Nearby genes
21	N/A	20	gene: ENSG00000280441	gene: ENSG00000280441, MIR6724-3 (-52787), gene: ENSG00000274868 (-52253), gene: ENSG00000280614 (-46899), RNA5-8SN3 (-45481), gene: ENSG00000286267 (-23005), gene: ENSG00000286146 (-20237), gene: ENSG00000286054 (-19124), gene: ENSG00000286178 (-17169), MIR6724-4 (-8619), gene: ENSG00000278775 (-8066), gene: ENSG00000281181 (-2689), RNA5-8SN1 (-1265), gene: ENSG00000286149 (+21079), gene: ENSG00000286032 (+24013), gene: ENSG00000286091 (+25225), gene: ENSG00000286148 (+27079)

30927-005.R1 (paired) (Report)

Host chromosome	Unaligned ends virus	Broken reads host	Disrupted genes	Nearby genes
21	85	13	gene: ENSG00000280441	gene: ENSG00000280441, MIR6724-3 (-53112), gene: ENSG00000274868 (-52578), gene: ENSG00000280614 (-47224), RNA5-8SN3 (-45806), gene: ENSG00000286267 (-23330), gene: ENSG00000286146 (-20562), gene: ENSG00000286054 (-19449), gene: ENSG00000286178 (-17494), MIR6724-4 (-8944), gene: ENSG00000278775 (-8391), gene: ENSG00000281181 (-3014), RNA5-8SN1 (-1590), gene: ENSG00000286149 (+20754), gene: ENSG00000286032 (+23688), gene: ENSG00000286091 (+24900), gene: ENSG00000286148 (+26754)

30927-005.R1 (paired) (Report)

Host chromosome	Unaligned ends virus	Broken reads host	Disrupted genes	Nearby genes
21	4,496	11	gene: ENSG00000280441	gene: ENSG00000280441, MIR6724-3 (-53127), gene: ENSG00000274868 (-52593), gene: ENSG00000280614 (-47239), RNA5-8SN3 (-45821), gene: ENSG00000286267 (-23345), gene: ENSG00000286146 (-20577), gene: ENSG00000286054 (-19464), gene: ENSG00000286178 (-17509), MIR6724-4 (-8959), gene: ENSG00000278775 (-8406), gene: ENSG00000281181 (-3029), RNA5-8SN1 (-1605), gene: ENSG00000286149 (+20739), gene: ENSG00000286032 (+23673), gene: ENSG00000286091 (+24885), gene: ENSG00000286148 (+26739)

30927-005.R1 (paired) (Report)

Host chromosome	Unaligned ends virus	Broken reads host	Disrupted genes	Nearby genes
21	4,427	64	gene: ENSG00000280441	gene: ENSG00000280441, MIR6724-3 (-53638), gene: ENSG00000274868 (-53104), gene: ENSG00000280614 (-47750), RNA5-8SN3 (-46332), gene: ENSG00000286267 (-23856), gene: ENSG00000286146 (-21088), gene: ENSG00000286054 (-19975), gene: ENSG00000286178 (-18020), MIR6724-4 (-9470), gene: ENSG00000278775 (-8917), gene: ENSG00000281181 (-3540), RNA5-8SN1 (-2116), gene: ENSG00000286149 (+20228), gene: ENSG00000286032 (+23162), gene: ENSG00000286091 (+24374), gene: ENSG00000286148 (+26228)

30927-005.R1 (paired) (Report)

Host chromosome	Unaligned ends virus	Broken reads host	Disrupted genes	Nearby genes
21	N/A	61	gene: ENSG00000280441	gene: ENSG00000280441, MIR6724-3 (-53668), gene: ENSG00000274868 (-53134), gene: ENSG00000280614 (-47780), RNA5-8SN3 (-46362), gene: ENSG00000286267 (-23886), gene: ENSG00000286146 (-21118), gene: ENSG00000286054 (-20005), gene: ENSG00000286178 (-18050), MIR6724-4 (-9500), gene: ENSG00000278775 (-8947), gene: ENSG00000281181 (-3570), RNA5-8SN1 (-2146), gene: ENSG00000286149 (+20198), gene: ENSG00000286032 (+23132), gene: ENSG00000286091 (+24344), gene: ENSG00000286148 (+26198)

30927-005.R1 (paired) (Report)

Host chromosome	Unaligned ends virus	Broken reads host	Disrupted genes	Nearby genes
21	N/A	65	gene: ENSG00000280441	gene: ENSG00000280441, MIR6724-3 (-53699), gene: ENSG00000274868 (-53165), gene: ENSG00000280614 (-47811), RNA5-8SN3 (-46393), gene: ENSG00000286267 (-23917), gene: ENSG00000286146 (-21149), gene: ENSG00000286054 (-20036), gene: ENSG00000286178 (-18081), MIR6724-4 (-9531), gene: ENSG00000278775 (-8978), gene: ENSG00000281181 (-3601), RNA5-8SN1 (-2177), gene: ENSG00000286149 (+20167), gene: ENSG00000286032 (+23101), gene: ENSG00000286091 (+24313), gene: ENSG00000286148 (+26167)

30927-005.R1 (paired) (Report)

Host chromosome	Unaligned ends virus	Broken reads host	Disrupted genes	Nearby genes
21	4,442	66	gene: ENSG00000280441	gene: ENSG00000280441, MIR6724-3 (-53704), gene: ENSG00000274868 (-53170), gene: ENSG00000280614 (-47816), RNA5-8SN3 (-46398), gene: ENSG00000286267 (-23922), gene: ENSG00000286146 (-21154), gene: ENSG00000286054 (-20041), gene: ENSG00000286178 (-18086), MIR6724-4 (-9536), gene: ENSG00000278775 (-8983), gene: ENSG00000281181 (-3606), RNA5-8SN1 (-2182), gene: ENSG00000286149 (+20162), gene: ENSG00000286032 (+23096), gene: ENSG00000286091 (+24308), gene: ENSG00000286148 (+26162)

30927-005.R1 (paired) (Report)

Host chromosome	Unaligned ends virus	Broken reads host	Disrupted genes	Nearby genes
21	N/A	64	gene: ENSG00000280441	gene: ENSG00000280441, MIR6724-3 (-53738), gene: ENSG00000274868 (-53204), gene: ENSG00000280614 (-47850), RNA5-8SN3 (-46432), gene: ENSG00000286267 (-23956), gene: ENSG00000286146 (-21188), gene: ENSG00000286054 (-20075), gene: ENSG00000286178 (-18120), MIR6724-4 (-9570), gene: ENSG00000278775 (-9017), gene: ENSG00000281181 (-3640), RNA5-8SN1 (-2216), gene: ENSG00000286149 (+20128), gene: ENSG00000286032 (+23062), gene: ENSG00000286091 (+24274), gene: ENSG00000286148 (+26128)

30927-005.R1 (paired) (Report)

Host chromosome	Unaligned ends virus	Broken reads host	Disrupted genes	Nearby genes
21	N/A	65	gene: ENSG00000280441	gene: ENSG00000280441, MIR6724-3 (-53754), gene: ENSG00000274868 (-53220), gene: ENSG00000280614 (-47866), RNA5-8SN3 (-46448), gene: ENSG00000286267 (-23972), gene: ENSG00000286146 (-21204), gene: ENSG00000286054 (-20091), gene: ENSG00000286178 (-18136), MIR6724-4 (-9586), gene: ENSG00000278775 (-9033), gene: ENSG00000281181 (-3656), RNA5-8SN1 (-2232), gene: ENSG00000286149 (+20112), gene: ENSG00000286032 (+23046), gene: ENSG00000286091 (+24258), gene: ENSG00000286148 (+26112)

30927-005.R1 (paired) (Report)

Host chromosome	Unaligned ends virus	Broken reads host	Disrupted genes	Nearby genes
21	N/A	64	gene: ENSG00000280441	gene: ENSG00000280441, MIR6724-3 (-53761), gene: ENSG00000274868 (-53227), gene: ENSG00000280614 (-47873), RNA5-8SN3 (-46455), gene: ENSG00000286267 (-23979), gene: ENSG00000286146 (-21211), gene: ENSG00000286054 (-20098), gene: ENSG00000286178 (-18143), MIR6724-4 (-9593), gene: ENSG00000278775 (-9040), gene: ENSG00000281181 (-3663), RNA5-8SN1 (-2239), gene: ENSG00000286149 (+20105), gene: ENSG00000286032 (+23039), gene: ENSG00000286091 (+24251), gene: ENSG00000286148 (+26105)

30927-005.R1 (paired) (Report)

Host chromosome	Unaligned ends virus	Broken reads host	Disrupted genes	Nearby genes
21	N/A	62	gene: ENSG00000280441	gene: ENSG00000280441, MIR6724-3 (-53764), gene: ENSG00000274868 (-53230), gene: ENSG00000280614 (-47876), RNA5-8SN3 (-46458), gene: ENSG00000286267 (-23982), gene: ENSG00000286146 (-21214), gene: ENSG00000286054 (-20101), gene: ENSG00000286178 (-18146), MIR6724-4 (-9596), gene: ENSG00000278775 (-9043), gene: ENSG00000281181 (-3666), RNA5-8SN1 (-2242), gene: ENSG00000286149 (+20102), gene: ENSG00000286032 (+23036), gene: ENSG00000286091 (+24248), gene: ENSG00000286148 (+26102)

30927-005.R1 (paired) (Report)

Host chromosome	Unaligned ends virus	Broken reads host	Disrupted genes	Nearby genes
21	N/A	60	gene: ENSG00000280441	gene: ENSG00000280441, MIR6724-3 (-53770), gene: ENSG00000274868 (-53236), gene: ENSG00000280614 (-47882), RNA5-8SN3 (-46464), gene: ENSG00000286267 (-23988), gene: ENSG00000286146 (-21220), gene: ENSG00000286054 (-20107), gene: ENSG00000286178 (-18152), MIR6724-4 (-9602), gene: ENSG00000278775 (-9049), gene: ENSG00000281181 (-3672), RNA5-8SN1 (-2248), gene: ENSG00000286149 (+20096), gene: ENSG00000286032 (+23030), gene: ENSG00000286091 (+24242), gene: ENSG00000286148 (+26096)

30927-005.R1 (paired) (Report)

Host chromosome	Unaligned ends virus	Broken reads host	Disrupted genes	Nearby genes
21	4,442	60	gene: ENSG00000280441	gene: ENSG00000280441, MIR6724-3 (-53773), gene: ENSG00000274868 (-53239), gene: ENSG00000280614 (-47885), RNA5-8SN3 (-46467), gene: ENSG00000286267 (-23991), gene: ENSG00000286146 (-21223), gene: ENSG00000286054 (-20110), gene: ENSG00000286178 (-18155), MIR6724-4 (-9605), gene: ENSG00000278775 (-9052), gene: ENSG00000281181 (-3675), RNA5-8SN1 (-2251), gene: ENSG00000286149 (+20093), gene: ENSG00000286032 (+23027), gene: ENSG00000286091 (+24239), gene: ENSG00000286148 (+26093)

30927-005.R1 (paired) (Report)

Host chromosome	Unaligned ends virus	Broken reads host	Disrupted genes	Nearby genes
21	N/A	54	gene: ENSG00000280441	gene: ENSG00000280441, MIR6724-3 (-53783), gene: ENSG00000274868 (-53249), gene: ENSG00000280614 (-47895), RNA5-8SN3 (-46477), gene: ENSG00000286267 (-24001), gene: ENSG00000286146 (-21233), gene: ENSG00000286054 (-20120), gene: ENSG00000286178 (-18165), MIR6724-4 (-9615), gene: ENSG00000278775 (-9062), gene: ENSG00000281181 (-3685), RNA5-8SN1 (-2261), gene: ENSG00000286149 (+20083), gene: ENSG00000286032 (+23017), gene: ENSG00000286091 (+24229), gene: ENSG00000286148 (+26083)

30927-005.R1 (paired) (Report)

Host chromosome	Unaligned ends virus	Broken reads host	Disrupted genes	Nearby genes
21	N/A	52	gene: ENSG00000280441	gene: ENSG00000280441, MIR6724-3 (-53794), gene: ENSG00000274868 (-53260), gene: ENSG00000280614 (-47906), RNA5-8SN3 (-46488), gene: ENSG00000286267 (-24012), gene: ENSG00000286146 (-21244), gene: ENSG00000286054 (-20131), gene: ENSG00000286178 (-18176), MIR6724-4 (-9626), gene: ENSG00000278775 (-9073), gene: ENSG00000281181 (-3696), RNA5-8SN1 (-2272), gene: ENSG00000286149 (+20072), gene: ENSG00000286032 (+23006), gene: ENSG00000286091 (+24218), gene: ENSG00000286148 (+26072)

30927-005.R1 (paired) (Report)

Host chromosome	Unaligned ends virus	Broken reads host	Disrupted genes	Nearby genes
21	5,000	51	gene: ENSG00000280441	gene: ENSG00000280441, MIR6724-3 (-53799), gene: ENSG00000274868 (-53265), gene: ENSG00000280614 (-47911), RNA5-8SN3 (-46493), gene: ENSG00000286267 (-24017), gene: ENSG00000286146 (-21249), gene: ENSG00000286054 (-20136), gene: ENSG00000286178 (-18181), MIR6724-4 (-9631), gene: ENSG00000278775 (-9078), gene: ENSG00000281181 (-3701), RNA5-8SN1 (-2277), gene: ENSG00000286149 (+20067), gene: ENSG00000286032 (+23001), gene: ENSG00000286091 (+24213), gene: ENSG00000286148 (+26067)

30927-005.R1 (paired) (Report)

Host chromosome	Unaligned ends virus	Broken reads host	Disrupted genes	Nearby genes
21	320	49	gene: ENSG00000280441	gene: ENSG00000280441, MIR6724-3 (-53813), gene: ENSG00000274868 (-53279), gene: ENSG00000280614 (-47925), RNA5-8SN3 (-46507), gene: ENSG00000286267 (-24031), gene: ENSG00000286146 (-21263), gene: ENSG00000286054 (-20150), gene: ENSG00000286178 (-18195), MIR6724-4 (-9645), gene: ENSG00000278775 (-9092), gene: ENSG00000281181 (-3715), RNA5-8SN1 (-2291), gene: ENSG00000286149 (+20053), gene: ENSG00000286032 (+22987), gene: ENSG00000286091 (+24199), gene: ENSG00000286148 (+26053)

30927-005.R1 (paired) (Report)

Host chromosome	Unaligned ends virus	Broken reads host	Disrupted genes	Nearby genes
21	N/A	47	gene: ENSG00000280441	gene: ENSG00000280441, MIR6724-3 (-53857), gene: ENSG00000274868 (-53323), gene: ENSG00000280614 (-47969), RNA5-8SN3 (-46551), gene: ENSG00000286267 (-24075), gene: ENSG00000286146 (-21307), gene: ENSG00000286054 (-20194), gene: ENSG00000286178 (-18239), MIR6724-4 (-9689), gene: ENSG00000278775 (-9136), gene: ENSG00000281181 (-3759), RNA5-8SN1 (-2335), gene: ENSG00000286149 (+20009), gene: ENSG00000286032 (+22943), gene: ENSG00000286091 (+24155), gene: ENSG00000286148 (+26009)

30927-005.R1 (paired) (Report)

Host chromosome	Unaligned ends virus	Broken reads host	Disrupted genes	Nearby genes
21	3,551	47	gene: ENSG00000280441	gene: ENSG00000280441, MIR6724-3 (-53859), gene: ENSG00000274868 (-53325), gene: ENSG00000280614 (-47971), RNA5-8SN3 (-46553), gene: ENSG00000286267 (-24077), gene: ENSG00000286146 (-21309), gene: ENSG00000286054 (-20196), gene: ENSG00000286178 (-18241), MIR6724-4 (-9691), gene: ENSG00000278775 (-9138), gene: ENSG00000281181 (-3761), RNA5-8SN1 (-2337), gene: ENSG00000286149 (+20007), gene: ENSG00000286032 (+22941), gene: ENSG00000286091 (+24153), gene: ENSG00000286148 (+26007)

30927-005.R1 (paired) (Report)

Host chromosome	Unaligned ends virus	Broken reads host	Disrupted genes	Nearby genes
21	N/A	47	gene: ENSG00000280441	gene: ENSG00000280441, MIR6724-3 (-53860), gene: ENSG00000274868 (-53326), gene: ENSG00000280614 (-47972), RNA5-8SN3 (-46554), gene: ENSG00000286267 (-24078), gene: ENSG00000286146 (-21310), gene: ENSG00000286054 (-20197), gene: ENSG00000286178 (-18242), MIR6724-4 (-9692), gene: ENSG00000278775 (-9139), gene: ENSG00000281181 (-3762), RNA5-8SN1 (-2338), gene: ENSG00000286149 (+20006), gene: ENSG00000286032 (+22940), gene: ENSG00000286091 (+24152), gene: ENSG00000286148 (+26006)

30927-005.R1 (paired) (Report)

Host chromosome	Unaligned ends virus	Broken reads host	Disrupted genes	Nearby genes
21	N/A	45	gene: ENSG00000280441	gene: ENSG00000280441, MIR6724-3 (-53867), gene: ENSG00000274868 (-53333), gene: ENSG00000280614 (-47979), RNA5-8SN3 (-46561), gene: ENSG00000286267 (-24085), gene: ENSG00000286146 (-21317), gene: ENSG00000286054 (-20204), gene: ENSG00000286178 (-18249), MIR6724-4 (-9699), gene: ENSG00000278775 (-9146), gene: ENSG00000281181 (-3769), RNA5-8SN1 (-2345), gene: ENSG00000286149 (+19999), gene: ENSG00000286032 (+22933), gene: ENSG00000286091 (+24145), gene: ENSG00000286148 (+25999)

30927-005.R1 (paired) (Report)

Host chromosome	Unaligned ends virus	Broken reads host	Disrupted genes	Nearby genes
21	N/A	45	gene: ENSG00000280441	gene: ENSG00000280441, MIR6724-3 (-53870), gene: ENSG00000274868 (-53336), gene: ENSG00000280614 (-47982), RNA5-8SN3 (-46564), gene: ENSG00000286267 (-24088), gene: ENSG00000286146 (-21320), gene: ENSG00000286054 (-20207), gene: ENSG00000286178 (-18252), MIR6724-4 (-9702), gene: ENSG00000278775 (-9149), gene: ENSG00000281181 (-3772), RNA5-8SN1 (-2348), gene: ENSG00000286149 (+19996), gene: ENSG00000286032 (+22930), gene: ENSG00000286091 (+24142), gene: ENSG00000286148 (+25996)

30927-005.R1 (paired) (Report)

Host chromosome	Unaligned ends virus	Broken reads host	Disrupted genes	Nearby genes
21	60,930	45	gene: ENSG00000280441	gene: ENSG00000280441, MIR6724-3 (-53871), gene: ENSG00000274868 (-53337), gene: ENSG00000280614 (-47983), RNA5-8SN3 (-46565), gene: ENSG00000286267 (-24089), gene: ENSG00000286146 (-21321), gene: ENSG00000286054 (-20208), gene: ENSG00000286178 (-18253), MIR6724-4 (-9703), gene: ENSG00000278775 (-9150), gene: ENSG00000281181 (-3773), RNA5-8SN1 (-2349), gene: ENSG00000286149 (+19995), gene: ENSG00000286032 (+22929), gene: ENSG00000286091 (+24141), gene: ENSG00000286148 (+25995)

30927-005.R1 (paired) (Report)

Host chromosome	Unaligned ends virus	Broken reads host	Disrupted genes	Nearby genes
21	N/A	46	gene: ENSG00000280441	gene: ENSG00000280441, MIR6724-3 (-53878), gene: ENSG00000274868 (-53344), gene: ENSG00000280614 (-47990), RNA5-8SN3 (-46572), gene: ENSG00000286267 (-24096), gene: ENSG00000286146 (-21328), gene: ENSG00000286054 (-20215), gene: ENSG00000286178 (-18260), MIR6724-4 (-9710), gene: ENSG00000278775 (-9157), gene: ENSG00000281181 (-3780), RNA5-8SN1 (-2356), gene: ENSG00000286149 (+19988), gene: ENSG00000286032 (+22922), gene: ENSG00000286091 (+24134), gene: ENSG00000286148 (+25988)

30927-005.R1 (paired) (Report)

Host chromosome	Unaligned ends virus	Broken reads host	Disrupted genes	Nearby genes
21	N/A	46	gene: ENSG00000280441	gene: ENSG00000280441, MIR6724-3 (-53885), gene: ENSG00000274868 (-53351), gene: ENSG00000280614 (-47997), RNA5-8SN3 (-46579), gene: ENSG00000286267 (-24103), gene: ENSG00000286146 (-21335), gene: ENSG00000286054 (-20222), gene: ENSG00000286178 (-18267), MIR6724-4 (-9717), gene: ENSG00000278775 (-9164), gene: ENSG00000281181 (-3787), RNA5-8SN1 (-2363), gene: ENSG00000286149 (+19981), gene: ENSG00000286032 (+22915), gene: ENSG00000286091 (+24127), gene: ENSG00000286148 (+25981)

30927-005.R1 (paired) (Report)

Host chromosome	Unaligned ends virus	Broken reads host	Disrupted genes	Nearby genes
21	4,126	46	gene: ENSG00000280441	gene: ENSG00000280441, MIR6724-3 (-53890), gene: ENSG00000274868 (-53356), gene: ENSG00000280614 (-48002), RNA5-8SN3 (-46584), gene: ENSG00000286267 (-24108), gene: ENSG00000286146 (-21340), gene: ENSG00000286054 (-20227), gene: ENSG00000286178 (-18272), MIR6724-4 (-9722), gene: ENSG00000278775 (-9169), gene: ENSG00000281181 (-3792), RNA5-8SN1 (-2368), gene: ENSG00000286149 (+19976), gene: ENSG00000286032 (+22910), gene: ENSG00000286091 (+24122), gene: ENSG00000286148 (+25976)

30927-005.R1 (paired) (Report)

Host chromosome	Unaligned ends virus	Broken reads host	Disrupted genes	Nearby genes
21	4,126	45	gene: ENSG00000280441	gene: ENSG00000280441, MIR6724-3 (-53894), gene: ENSG00000274868 (-53360), gene: ENSG00000280614 (-48006), RNA5-8SN3 (-46588), gene: ENSG00000286267 (-24112), gene: ENSG00000286146 (-21344), gene: ENSG00000286054 (-20231), gene: ENSG00000286178 (-18276), MIR6724-4 (-9726), gene: ENSG00000278775 (-9173), gene: ENSG00000281181 (-3796), RNA5-8SN1 (-2372), gene: ENSG00000286149 (+19972), gene: ENSG00000286032 (+22906), gene: ENSG00000286091 (+24118), gene: ENSG00000286148 (+25972)

30927-005.R1 (paired) (Report)

Host chromosome	Unaligned ends virus	Broken reads host	Disrupted genes	Nearby genes
21	3,510	46	gene: ENSG00000280441	gene: ENSG00000280441, MIR6724-3 (-54017), gene: ENSG00000274868 (-53483), gene: ENSG00000280614 (-48129), RNA5-8SN3 (-46711), gene: ENSG00000286267 (-24235), gene: ENSG00000286146 (-21467), gene: ENSG00000286054 (-20354), gene: ENSG00000286178 (-18399), MIR6724-4 (-9849), gene: ENSG00000278775 (-9296), gene: ENSG00000281181 (-3919), RNA5-8SN1 (-2495), gene: ENSG00000286149 (+19849), gene: ENSG00000286032 (+22783), gene: ENSG00000286091 (+23995), gene: ENSG00000286148 (+25849)

30927-005.R1 (paired) (Report)

Host chromosome	Unaligned ends virus	Broken reads host	Disrupted genes	Nearby genes
21	N/A	38	gene: ENSG00000280441	gene: ENSG00000280441, MIR6724-3 (-54039), gene: ENSG00000274868 (-53505), gene: ENSG00000280614 (-48151), RNA5-8SN3 (-46733), gene: ENSG00000286267 (-24257), gene: ENSG00000286146 (-21489), gene: ENSG00000286054 (-20376), gene: ENSG00000286178 (-18421), MIR6724-4 (-9871), gene: ENSG00000278775 (-9318), gene: ENSG00000281181 (-3941), RNA5-8SN1 (-2517), gene: ENSG00000286149 (+19827), gene: ENSG00000286032 (+22761), gene: ENSG00000286091 (+23973), gene: ENSG00000286148 (+25827)

30927-005.R1 (paired) (Report)

Host chromosome	Unaligned ends virus	Broken reads host	Disrupted genes	Nearby genes
21	N/A	38	gene: ENSG00000280441	gene: ENSG00000280441, MIR6724-3 (-54041), gene: ENSG00000274868 (-53507), gene: ENSG00000280614 (-48153), RNA5-8SN3 (-46735), gene: ENSG00000286267 (-24259), gene: ENSG00000286146 (-21491), gene: ENSG00000286054 (-20378), gene: ENSG00000286178 (-18423), MIR6724-4 (-9873), gene: ENSG00000278775 (-9320), gene: ENSG00000281181 (-3943), RNA5-8SN1 (-2519), gene: ENSG00000286149 (+19825), gene: ENSG00000286032 (+22759), gene: ENSG00000286091 (+23971), gene: ENSG00000286148 (+25825)

30927-005.R1 (paired) (Report)

Host chromosome	Unaligned ends virus	Broken reads host	Disrupted genes	Nearby genes
21	8,627	36	gene: ENSG00000280441	gene: ENSG00000280441, MIR6724-3 (-54048), gene: ENSG00000274868 (-53514), gene: ENSG00000280614 (-48160), RNA5-8SN3 (-46742), gene: ENSG00000286267 (-24266), gene: ENSG00000286146 (-21498), gene: ENSG00000286054 (-20385), gene: ENSG00000286178 (-18430), MIR6724-4 (-9880), gene: ENSG00000278775 (-9327), gene: ENSG00000281181 (-3950), RNA5-8SN1 (-2526), gene: ENSG00000286149 (+19818), gene: ENSG00000286032 (+22752), gene: ENSG00000286091 (+23964), gene: ENSG00000286148 (+25818)

30927-005.R1 (paired) (Report)

Host chromosome	Unaligned ends virus	Broken reads host	Disrupted genes	Nearby genes
21	590	35	gene: ENSG00000280441	gene: ENSG00000280441, MIR6724-3 (-54054), gene: ENSG00000274868 (-53520), gene: ENSG00000280614 (-48166), RNA5-8SN3 (-46748), gene: ENSG00000286267 (-24272), gene: ENSG00000286146 (-21504), gene: ENSG00000286054 (-20391), gene: ENSG00000286178 (-18436), MIR6724-4 (-9886), gene: ENSG00000278775 (-9333), gene: ENSG00000281181 (-3956), RNA5-8SN1 (-2532), gene: ENSG00000286149 (+19812), gene: ENSG00000286032 (+22746), gene: ENSG00000286091 (+23958), gene: ENSG00000286148 (+25812)

30927-005.R1 (paired) (Report)

Host chromosome	Unaligned ends virus	Broken reads host	Disrupted genes	Nearby genes
21	N/A	33	gene: ENSG00000280441	gene: ENSG00000280441, MIR6724-3 (-54072), gene: ENSG00000274868 (-53538), gene: ENSG00000280614 (-48184), RNA5-8SN3 (-46766), gene: ENSG00000286267 (-24290), gene: ENSG00000286146 (-21522), gene: ENSG00000286054 (-20409), gene: ENSG00000286178 (-18454), MIR6724-4 (-9904), gene: ENSG00000278775 (-9351), gene: ENSG00000281181 (-3974), RNA5-8SN1 (-2550), gene: ENSG00000286149 (+19794), gene: ENSG00000286032 (+22728), gene: ENSG00000286091 (+23940), gene: ENSG00000286148 (+25794)

30927-005.R1 (paired) (Report)

Host chromosome	Unaligned ends virus	Broken reads host	Disrupted genes	Nearby genes
21	N/A	33	gene: ENSG00000280441	gene: ENSG00000280441, MIR6724-3 (-54074), gene: ENSG00000274868 (-53540), gene: ENSG00000280614 (-48186), RNA5-8SN3 (-46768), gene: ENSG00000286267 (-24292), gene: ENSG00000286146 (-21524), gene: ENSG00000286054 (-20411), gene: ENSG00000286178 (-18456), MIR6724-4 (-9906), gene: ENSG00000278775 (-9353), gene: ENSG00000281181 (-3976), RNA5-8SN1 (-2552), gene: ENSG00000286149 (+19792), gene: ENSG00000286032 (+22726), gene: ENSG00000286091 (+23938), gene: ENSG00000286148 (+25792)

30927-005.R1 (paired) (Report)

Host chromosome	Unaligned ends virus	Broken reads host	Disrupted genes	Nearby genes
21	N/A	27	gene: ENSG00000280441	gene: ENSG00000280441, MIR6724-3 (-54120), gene: ENSG00000274868 (-53586), gene: ENSG00000280614 (-48232), RNA5-8SN3 (-46814), gene: ENSG00000286267 (-24338), gene: ENSG00000286146 (-21570), gene: ENSG00000286054 (-20457), gene: ENSG00000286178 (-18502), MIR6724-4 (-9952), gene: ENSG00000278775 (-9399), gene: ENSG00000281181 (-4022), RNA5-8SN1 (-2598), gene: ENSG00000286149 (+19746), gene: ENSG00000286032 (+22680), gene: ENSG00000286091 (+23892), gene: ENSG00000286148 (+25746)

30927-005.R1 (paired) (Report)

Host chromosome	Unaligned ends virus	Broken reads host	Disrupted genes	Nearby genes
21	3,426	27	gene: ENSG00000280441	gene: ENSG00000280441, MIR6724-3 (-54125), gene: ENSG00000274868 (-53591), gene: ENSG00000280614 (-48237), RNA5-8SN3 (-46819), gene: ENSG00000286267 (-24343), gene: ENSG00000286146 (-21575), gene: ENSG00000286054 (-20462), gene: ENSG00000286178 (-18507), MIR6724-4 (-9957), gene: ENSG00000278775 (-9404), gene: ENSG00000281181 (-4027), RNA5-8SN1 (-2603), gene: ENSG00000286149 (+19741), gene: ENSG00000286032 (+22675), gene: ENSG00000286091 (+23887), gene: ENSG00000286148 (+25741)

30927-005.R1 (paired) (Report)

Host chromosome	Unaligned ends virus	Broken reads host	Disrupted genes	Nearby genes
21	4,358	27	gene: ENSG00000280441	gene: ENSG00000280441, MIR6724-3 (-54156), gene: ENSG00000274868 (-53622), gene: ENSG00000280614 (-48268), RNA5-8SN3 (-46850), gene: ENSG00000286267 (-24374), gene: ENSG00000286146 (-21606), gene: ENSG00000286054 (-20493), gene: ENSG00000286178 (-18538), MIR6724-4 (-9988), gene: ENSG00000278775 (-9435), gene: ENSG00000281181 (-4058), RNA5-8SN1 (-2634), gene: ENSG00000286149 (+19710), gene: ENSG00000286032 (+22644), gene: ENSG00000286091 (+23856), gene: ENSG00000286148 (+25710)

30927-005.R1 (paired) (Report)

Host chromosome	Unaligned ends virus	Broken reads host	Disrupted genes	Nearby genes
21	4,126	26	gene: ENSG00000280441	gene: ENSG00000280441, MIR6724-3 (-54167), gene: ENSG00000274868 (-53633), gene: ENSG00000280614 (-48279), RNA5-8SN3 (-46861), gene: ENSG00000286267 (-24385), gene: ENSG00000286146 (-21617), gene: ENSG00000286054 (-20504), gene: ENSG00000286178 (-18549), MIR6724-4 (-9999), gene: ENSG00000278775 (-9446), gene: ENSG00000281181 (-4069), RNA5-8SN1 (-2645), gene: ENSG00000286149 (+19699), gene: ENSG00000286032 (+22633), gene: ENSG00000286091 (+23845), gene: ENSG00000286148 (+25699)

30927-005.R1 (paired) (Report)

Host chromosome	Unaligned ends virus	Broken reads host	Disrupted genes	Nearby genes
21	N/A	26	gene: ENSG00000280441	gene: ENSG00000280441, MIR6724-3 (-54175), gene: ENSG00000274868 (-53641), gene: ENSG00000280614 (-48287), RNA5-8SN3 (-46869), gene: ENSG00000286267 (-24393), gene: ENSG00000286146 (-21625), gene: ENSG00000286054 (-20512), gene: ENSG00000286178 (-18557), MIR6724-4 (-10007), gene: ENSG00000278775 (-9454), gene: ENSG00000281181 (-4077), RNA5-8SN1 (-2653), gene: ENSG00000286149 (+19691), gene: ENSG00000286032 (+22625), gene: ENSG00000286091 (+23837), gene: ENSG00000286148 (+25691)

30927-005.R1 (paired) (Report)

Host chromosome	Unaligned ends virus	Broken reads host	Disrupted genes	Nearby genes
21	4,496	25	gene: ENSG00000280441	gene: ENSG00000280441, MIR6724-3 (-54195), gene: ENSG00000274868 (-53661), gene: ENSG00000280614 (-48307), RNA5-8SN3 (-46889), gene: ENSG00000286267 (-24413), gene: ENSG00000286146 (-21645), gene: ENSG00000286054 (-20532), gene: ENSG00000286178 (-18577), MIR6724-4 (-10027), gene: ENSG00000278775 (-9474), gene: ENSG00000281181 (-4097), RNA5-8SN1 (-2673), gene: ENSG00000286149 (+19671), gene: ENSG00000286032 (+22605), gene: ENSG00000286091 (+23817), gene: ENSG00000286148 (+25671)

30927-005.R1 (paired) (Report)

Host chromosome	Unaligned ends virus	Broken reads host	Disrupted genes	Nearby genes
21	N/A	25	gene: ENSG00000280441	gene: ENSG00000280441, MIR6724-3 (-54219), gene: ENSG00000274868 (-53685), gene: ENSG00000280614 (-48331), RNA5-8SN3 (-46913), gene: ENSG00000286267 (-24437), gene: ENSG00000286146 (-21669), gene: ENSG00000286054 (-20556), gene: ENSG00000286178 (-18601), MIR6724-4 (-10051), gene: ENSG00000278775 (-9498), gene: ENSG00000281181 (-4121), RNA5-8SN1 (-2697), gene: ENSG00000286149 (+19647), gene: ENSG00000286032 (+22581), gene: ENSG00000286091 (+23793), gene: ENSG00000286148 (+25647)

30927-005.R1 (paired) (Report)

Host chromosome	Unaligned ends virus	Broken reads host	Disrupted genes	Nearby genes
21	60,930	26	gene: ENSG00000280441	gene: ENSG00000280441, MIR6724-3 (-54284), gene: ENSG00000274868 (-53750), gene: ENSG00000280614 (-48396), RNA5-8SN3 (-46978), gene: ENSG00000286267 (-24502), gene: ENSG00000286146 (-21734), gene: ENSG00000286054 (-20621), gene: ENSG00000286178 (-18666), MIR6724-4 (-10116), gene: ENSG00000278775 (-9563), gene: ENSG00000281181 (-4186), RNA5-8SN1 (-2762), gene: ENSG00000286149 (+19582), gene: ENSG00000286032 (+22516), gene: ENSG00000286091 (+23728), gene: ENSG00000286148 (+25582)

30927-005.R1 (paired) (Report)

Host chromosome	Unaligned ends virus	Broken reads host	Disrupted genes	Nearby genes
21	N/A	27	gene: ENSG00000280441	gene: ENSG00000280441, MIR6724-3 (-54515), gene: ENSG00000274868 (-53981), gene: ENSG00000280614 (-48627), RNA5-8SN3 (-47209), gene: ENSG00000286267 (-24733), gene: ENSG00000286146 (-21965), gene: ENSG00000286054 (-20852), gene: ENSG00000286178 (-18897), MIR6724-4 (-10347), gene: ENSG00000278775 (-9794), gene: ENSG00000281181 (-4417), RNA5-8SN1 (-2993), gene: ENSG00000286149 (+19351), gene: ENSG00000286032 (+22285), gene: ENSG00000286091 (+23497), gene: ENSG00000286148 (+25351)

30927-005.R1 (paired) (Report)

Host chromosome	Unaligned ends virus	Broken reads host	Disrupted genes	Nearby genes
21	N/A	35	gene: ENSG00000280441	gene: ENSG00000280441, MIR6724-3 (-54665), gene: ENSG00000274868 (-54131), gene: ENSG00000280614 (-48777), RNA5-8SN3 (-47359), gene: ENSG00000286267 (-24883), gene: ENSG00000286146 (-22115), gene: ENSG00000286054 (-21002), gene: ENSG00000286178 (-19047), MIR6724-4 (-10497), gene: ENSG00000278775 (-9944), gene: ENSG00000281181 (-4567), RNA5-8SN1 (-3143), gene: ENSG00000286149 (+19201), gene: ENSG00000286032 (+22135), gene: ENSG00000286091 (+23347), gene: ENSG00000286148 (+25201)

30927-005.R1 (paired) (Report)

Host chromosome	Unaligned ends virus	Broken reads host	Disrupted genes	Nearby genes
21	61,253	34	gene: ENSG00000280441	gene: ENSG00000280441, MIR6724-3 (-54667), gene: ENSG00000274868 (-54133), gene: ENSG00000280614 (-48779), RNA5-8SN3 (-47361), gene: ENSG00000286267 (-24885), gene: ENSG00000286146 (-22117), gene: ENSG00000286054 (-21004), gene: ENSG00000286178 (-19049), MIR6724-4 (-10499), gene: ENSG00000278775 (-9946), gene: ENSG00000281181 (-4569), RNA5-8SN1 (-3145), gene: ENSG00000286149 (+19199), gene: ENSG00000286032 (+22133), gene: ENSG00000286091 (+23345), gene: ENSG00000286148 (+25199)

30927-005.R1 (paired) (Report)

Host chromosome	Unaligned ends virus	Broken reads host	Disrupted genes	Nearby genes
21	N/A	34	gene: ENSG00000280441	gene: ENSG00000280441, MIR6724-3 (-54669), gene: ENSG00000274868 (-54135), gene: ENSG00000280614 (-48781), RNA5-8SN3 (-47363), gene: ENSG00000286267 (-24887), gene: ENSG00000286146 (-22119), gene: ENSG00000286054 (-21006), gene: ENSG00000286178 (-19051), MIR6724-4 (-10501), gene: ENSG00000278775 (-9948), gene: ENSG00000281181 (-4571), RNA5-8SN1 (-3147), gene: ENSG00000286149 (+19197), gene: ENSG00000286032 (+22131), gene: ENSG00000286091 (+23343), gene: ENSG00000286148 (+25197)

30927-005.R1 (paired) (Report)

Host chromosome	Unaligned ends virus	Broken reads host	Disrupted genes	Nearby genes
21	N/A	33	gene: ENSG00000280441	gene: ENSG00000280441, MIR6724-3 (-54696), gene: ENSG00000274868 (-54162), gene: ENSG00000280614 (-48808), RNA5-8SN3 (-47390), gene: ENSG00000286267 (-24914), gene: ENSG00000286146 (-22146), gene: ENSG00000286054 (-21033), gene: ENSG00000286178 (-19078), MIR6724-4 (-10528), gene: ENSG00000278775 (-9975), gene: ENSG00000281181 (-4598), RNA5-8SN1 (-3174), gene: ENSG00000286149 (+19170), gene: ENSG00000286032 (+22104), gene: ENSG00000286091 (+23316), gene: ENSG00000286148 (+25170)

30927-005.R1 (paired) (Report)

Host chromosome	Unaligned ends virus	Broken reads host	Disrupted genes	Nearby genes
21	N/A	33	gene: ENSG00000280441	gene: ENSG00000280441, MIR6724-3 (-54697), gene: ENSG00000274868 (-54163), gene: ENSG00000280614 (-48809), RNA5-8SN3 (-47391), gene: ENSG00000286267 (-24915), gene: ENSG00000286146 (-22147), gene: ENSG00000286054 (-21034), gene: ENSG00000286178 (-19079), MIR6724-4 (-10529), gene: ENSG00000278775 (-9976), gene: ENSG00000281181 (-4599), RNA5-8SN1 (-3175), gene: ENSG00000286149 (+19169), gene: ENSG00000286032 (+22103), gene: ENSG00000286091 (+23315), gene: ENSG00000286148 (+25169)

30927-005.R1 (paired) (Report)

Host chromosome	Unaligned ends virus	Broken reads host	Disrupted genes	Nearby genes
21	N/A	32	gene: ENSG00000280441	gene: ENSG00000280441, MIR6724-3 (-54722), gene: ENSG00000274868 (-54188), gene: ENSG00000280614 (-48834), RNA5-8SN3 (-47416), gene: ENSG00000286267 (-24940), gene: ENSG00000286146 (-22172), gene: ENSG00000286054 (-21059), gene: ENSG00000286178 (-19104), MIR6724-4 (-10554), gene: ENSG00000278775 (-10001), gene: ENSG00000281181 (-4624), RNA5-8SN1 (-3200), gene: ENSG00000286149 (+19144), gene: ENSG00000286032 (+22078), gene: ENSG00000286091 (+23290), gene: ENSG00000286148 (+25144)

30927-005.R1 (paired) (Report)

Host chromosome	Unaligned ends virus	Broken reads host	Disrupted genes	Nearby genes
21	N/A	34	gene: ENSG00000280441	gene: ENSG00000280441, MIR6724-3 (-54752), gene: ENSG00000274868 (-54218), gene: ENSG00000280614 (-48864), RNA5-8SN3 (-47446), gene: ENSG00000286267 (-24970), gene: ENSG00000286146 (-22202), gene: ENSG00000286054 (-21089), gene: ENSG00000286178 (-19134), MIR6724-4 (-10584), gene: ENSG00000278775 (-10031), gene: ENSG00000281181 (-4654), RNA5-8SN1 (-3230), gene: ENSG00000286149 (+19114), gene: ENSG00000286032 (+22048), gene: ENSG00000286091 (+23260), gene: ENSG00000286148 (+25114)

30927-005.R1 (paired) (Report)

Host chromosome	Unaligned ends virus	Broken reads host	Disrupted genes	Nearby genes
21	N/A	34	gene: ENSG00000280441	gene: ENSG00000280441, MIR6724-3 (-54753), gene: ENSG00000274868 (-54219), gene: ENSG00000280614 (-48865), RNA5-8SN3 (-47447), gene: ENSG00000286267 (-24971), gene: ENSG00000286146 (-22203), gene: ENSG00000286054 (-21090), gene: ENSG00000286178 (-19135), MIR6724-4 (-10585), gene: ENSG00000278775 (-10032), gene: ENSG00000281181 (-4655), RNA5-8SN1 (-3231), gene: ENSG00000286149 (+19113), gene: ENSG00000286032 (+22047), gene: ENSG00000286091 (+23259), gene: ENSG00000286148 (+25113)

30927-005.R1 (paired) (Report)

Host chromosome	Unaligned ends virus	Broken reads host	Disrupted genes	Nearby genes
21	N/A	34	gene: ENSG00000280441	gene: ENSG00000280441, MIR6724-3 (-54755), gene: ENSG00000274868 (-54221), gene: ENSG00000280614 (-48867), RNA5-8SN3 (-47449), gene: ENSG00000286267 (-24973), gene: ENSG00000286146 (-22205), gene: ENSG00000286054 (-21092), gene: ENSG00000286178 (-19137), MIR6724-4 (-10587), gene: ENSG00000278775 (-10034), gene: ENSG00000281181 (-4657), RNA5-8SN1 (-3233), gene: ENSG00000286149 (+19111), gene: ENSG00000286032 (+22045), gene: ENSG00000286091 (+23257), gene: ENSG00000286148 (+25111)

30927-005.R1 (paired) (Report)

Host chromosome	Unaligned ends virus	Broken reads host	Disrupted genes	Nearby genes
21	6,188	29	gene: ENSG00000280441	gene: ENSG00000280441, MIR6724-3 (-54776), gene: ENSG00000274868 (-54242), gene: ENSG00000280614 (-48888), RNA5-8SN3 (-47470), gene: ENSG00000286267 (-24994), gene: ENSG00000286146 (-22226), gene: ENSG00000286054 (-21113), gene: ENSG00000286178 (-19158), MIR6724-4 (-10608), gene: ENSG00000278775 (-10055), gene: ENSG00000281181 (-4678), RNA5-8SN1 (-3254), gene: ENSG00000286149 (+19090), gene: ENSG00000286032 (+22024), gene: ENSG00000286091 (+23236), gene: ENSG00000286148 (+25090)

30927-005.R1 (paired) (Report)

Host chromosome	Unaligned ends virus	Broken reads host	Disrupted genes	Nearby genes
21	N/A	26	gene: ENSG00000280441	gene: ENSG00000280441, MIR6724-3 (-54787), gene: ENSG00000274868 (-54253), gene: ENSG00000280614 (-48899), RNA5-8SN3 (-47481), gene: ENSG00000286267 (-25005), gene: ENSG00000286146 (-22237), gene: ENSG00000286054 (-21124), gene: ENSG00000286178 (-19169), MIR6724-4 (-10619), gene: ENSG00000278775 (-10066), gene: ENSG00000281181 (-4689), RNA5-8SN1 (-3265), gene: ENSG00000286149 (+19079), gene: ENSG00000286032 (+22013), gene: ENSG00000286091 (+23225), gene: ENSG00000286148 (+25079)

30927-005.R1 (paired) (Report)

Host chromosome	Unaligned ends virus	Broken reads host	Disrupted genes	Nearby genes
21	N/A	26	gene: ENSG00000280441	gene: ENSG00000280441, MIR6724-3 (-54788), gene: ENSG00000274868 (-54254), gene: ENSG00000280614 (-48900), RNA5-8SN3 (-47482), gene: ENSG00000286267 (-25006), gene: ENSG00000286146 (-22238), gene: ENSG00000286054 (-21125), gene: ENSG00000286178 (-19170), MIR6724-4 (-10620), gene: ENSG00000278775 (-10067), gene: ENSG00000281181 (-4690), RNA5-8SN1 (-3266), gene: ENSG00000286149 (+19078), gene: ENSG00000286032 (+22012), gene: ENSG00000286091 (+23224), gene: ENSG00000286148 (+25078)

30927-005.R1 (paired) (Report)

Host chromosome	Unaligned ends virus	Broken reads host	Disrupted genes	Nearby genes
21	N/A	25	gene: ENSG00000280441	gene: ENSG00000280441, MIR6724-3 (-54793), gene: ENSG00000274868 (-54259), gene: ENSG00000280614 (-48905), RNA5-8SN3 (-47487), gene: ENSG00000286267 (-25011), gene: ENSG00000286146 (-22243), gene: ENSG00000286054 (-21130), gene: ENSG00000286178 (-19175), MIR6724-4 (-10625), gene: ENSG00000278775 (-10072), gene: ENSG00000281181 (-4695), RNA5-8SN1 (-3271), gene: ENSG00000286149 (+19073), gene: ENSG00000286032 (+22007), gene: ENSG00000286091 (+23219), gene: ENSG00000286148 (+25073)

30927-005.R1 (paired) (Report)

Host chromosome	Unaligned ends virus	Broken reads host	Disrupted genes	Nearby genes
21	N/A	24	gene: ENSG00000280441	gene: ENSG00000280441, MIR6724-3 (-54838), gene: ENSG00000274868 (-54304), gene: ENSG00000280614 (-48950), RNA5-8SN3 (-47532), gene: ENSG00000286267 (-25056), gene: ENSG00000286146 (-22288), gene: ENSG00000286054 (-21175), gene: ENSG00000286178 (-19220), MIR6724-4 (-10670), gene: ENSG00000278775 (-10117), gene: ENSG00000281181 (-4740), RNA5-8SN1 (-3316), gene: ENSG00000286149 (+19028), gene: ENSG00000286032 (+21962), gene: ENSG00000286091 (+23174), gene: ENSG00000286148 (+25028)

30927-005.R1 (paired) (Report)

Host chromosome	Unaligned ends virus	Broken reads host	Disrupted genes	Nearby genes
21	6,702	18	gene: ENSG00000280441	gene: ENSG00000280441, MIR6724-3 (-55134), gene: ENSG00000274868 (-54600), gene: ENSG00000280614 (-49246), RNA5-8SN3 (-47828), gene: ENSG00000286267 (-25352), gene: ENSG00000286146 (-22584), gene: ENSG00000286054 (-21471), gene: ENSG00000286178 (-19516), MIR6724-4 (-10966), gene: ENSG00000278775 (-10413), gene: ENSG00000281181 (-5036), RNA5-8SN1 (-3612), gene: ENSG00000286149 (+18732), gene: ENSG00000286032 (+21666), gene: ENSG00000286091 (+22878), gene: ENSG00000286148 (+24732)

30927-005.R1 (paired) (Report)

Host chromosome	Unaligned ends virus	Broken reads host	Disrupted genes	Nearby genes
21	N/A	15	gene: ENSG00000280441	gene: ENSG00000280441, MIR6724-3 (-55145), gene: ENSG00000274868 (-54611), gene: ENSG00000280614 (-49257), RNA5-8SN3 (-47839), gene: ENSG00000286267 (-25363), gene: ENSG00000286146 (-22595), gene: ENSG00000286054 (-21482), gene: ENSG00000286178 (-19527), MIR6724-4 (-10977), gene: ENSG00000278775 (-10424), gene: ENSG00000281181 (-5047), RNA5-8SN1 (-3623), gene: ENSG00000286149 (+18721), gene: ENSG00000286032 (+21655), gene: ENSG00000286091 (+22867), gene: ENSG00000286148 (+24721)

30927-005.R1 (paired) (Report)

Host chromosome	Unaligned ends virus	Broken reads host	Disrupted genes	Nearby genes
21	N/A	12	gene: ENSG00000280441	gene: ENSG00000280441, MIR6724-3 (-55223), gene: ENSG00000274868 (-54689), gene: ENSG00000280614 (-49335), RNA5-8SN3 (-47917), gene: ENSG00000286267 (-25441), gene: ENSG00000286146 (-22673), gene: ENSG00000286054 (-21560), gene: ENSG00000286178 (-19605), MIR6724-4 (-11055), gene: ENSG00000278775 (-10502), gene: ENSG00000281181 (-5125), RNA5-8SN1 (-3701), gene: ENSG00000286149 (+18643), gene: ENSG00000286032 (+21577), gene: ENSG00000286091 (+22789), gene: ENSG00000286148 (+24643)

30927-005.R1 (paired) (Report)

Host chromosome	Unaligned ends virus	Broken reads host	Disrupted genes	Nearby genes
21	1,930	9	gene: ENSG00000280441	gene: ENSG00000280441, MIR6724-3 (-55272), gene: ENSG00000274868 (-54738), gene: ENSG00000280614 (-49384), RNA5-8SN3 (-47966), gene: ENSG00000286267 (-25490), gene: ENSG00000286146 (-22722), gene: ENSG00000286054 (-21609), gene: ENSG00000286178 (-19654), MIR6724-4 (-11104), gene: ENSG00000278775 (-10551), gene: ENSG00000281181 (-5174), RNA5-8SN1 (-3750), gene: ENSG00000286149 (+18594), gene: ENSG00000286032 (+21528), gene: ENSG00000286091 (+22740), gene: ENSG00000286148 (+24594)

30927-005.R1 (paired) (Report)

Host chromosome	Unaligned ends virus	Broken reads host	Disrupted genes	Nearby genes
21	N/A	8	gene: ENSG00000280441	gene: ENSG00000280441, MIR6724-3 (-55517), gene: ENSG00000274868 (-54983), gene: ENSG00000280614 (-49629), RNA5-8SN3 (-48211), gene: ENSG00000286267 (-25735), gene: ENSG00000286146 (-22967), gene: ENSG00000286054 (-21854), gene: ENSG00000286178 (-19899), MIR6724-4 (-11349), gene: ENSG00000278775 (-10796), gene: ENSG00000281181 (-5419), RNA5-8SN1 (-3995), gene: ENSG00000286149 (+18349), gene: ENSG00000286032 (+21283), gene: ENSG00000286091 (+22495), gene: ENSG00000286148 (+24349)

30927-005.R1 (paired) (Report)

Host chromosome	Unaligned ends virus	Broken reads host	Disrupted genes	Nearby genes
21	N/A	39	gene: ENSG00000280441	gene: ENSG00000280441, MIR6724-3 (-57355), gene: ENSG00000274868 (-56821), gene: ENSG00000280614 (-51467), RNA5-8SN3 (-50049), gene: ENSG00000286267 (-27573), gene: ENSG00000286146 (-24805), gene: ENSG00000286054 (-23692), gene: ENSG00000286178 (-21737), MIR6724-4 (-13187), gene: ENSG00000278775 (-12634), gene: ENSG00000281181 (-7257), RNA5-8SN1 (-5833), gene: ENSG00000286149 (+16511), gene: ENSG00000286032 (+19445), gene: ENSG00000286091 (+20657), gene: ENSG00000286148 (+22511)

30927-005.R1 (paired) (Report)

Host chromosome	Unaligned ends virus	Broken reads host	Disrupted genes	Nearby genes
21	61,253	39	gene: ENSG00000280441	gene: ENSG00000280441, MIR6724-3 (-57366), gene: ENSG00000274868 (-56832), gene: ENSG00000280614 (-51478), RNA5-8SN3 (-50060), gene: ENSG00000286267 (-27584), gene: ENSG00000286146 (-24816), gene: ENSG00000286054 (-23703), gene: ENSG00000286178 (-21748), MIR6724-4 (-13198), gene: ENSG00000278775 (-12645), gene: ENSG00000281181 (-7268), RNA5-8SN1 (-5844), gene: ENSG00000286149 (+16500), gene: ENSG00000286032 (+19434), gene: ENSG00000286091 (+20646), gene: ENSG00000286148 (+22500)

30927-005.R1 (paired) (Report)

Host chromosome	Unaligned ends virus	Broken reads host	Disrupted genes	Nearby genes
21	4,126	34	gene: ENSG00000280441	gene: ENSG00000280441, MIR6724-3 (-57372), gene: ENSG00000274868 (-56838), gene: ENSG00000280614 (-51484), RNA5-8SN3 (-50066), gene: ENSG00000286267 (-27590), gene: ENSG00000286146 (-24822), gene: ENSG00000286054 (-23709), gene: ENSG00000286178 (-21754), MIR6724-4 (-13204), gene: ENSG00000278775 (-12651), gene: ENSG00000281181 (-7274), RNA5-8SN1 (-5850), gene: ENSG00000286149 (+16494), gene: ENSG00000286032 (+19428), gene: ENSG00000286091 (+20640), gene: ENSG00000286148 (+22494)

30927-005.R1 (paired) (Report)

Host chromosome	Unaligned ends virus	Broken reads host	Disrupted genes	Nearby genes
21	N/A	33	gene: ENSG00000280441	gene: ENSG00000280441, MIR6724-3 (-57373), gene: ENSG00000274868 (-56839), gene: ENSG00000280614 (-51485), RNA5-8SN3 (-50067), gene: ENSG00000286267 (-27591), gene: ENSG00000286146 (-24823), gene: ENSG00000286054 (-23710), gene: ENSG00000286178 (-21755), MIR6724-4 (-13205), gene: ENSG00000278775 (-12652), gene: ENSG00000281181 (-7275), RNA5-8SN1 (-5851), gene: ENSG00000286149 (+16493), gene: ENSG00000286032 (+19427), gene: ENSG00000286091 (+20639), gene: ENSG00000286148 (+22493)

30927-005.R1 (paired) (Report)

Host chromosome	Unaligned ends virus	Broken reads host	Disrupted genes	Nearby genes
21	2,822	32	gene: ENSG00000280441	gene: ENSG00000280441, MIR6724-3 (-57389), gene: ENSG00000274868 (-56855), gene: ENSG00000280614 (-51501), RNA5-8SN3 (-50083), gene: ENSG00000286267 (-27607), gene: ENSG00000286146 (-24839), gene: ENSG00000286054 (-23726), gene: ENSG00000286178 (-21771), MIR6724-4 (-13221), gene: ENSG00000278775 (-12668), gene: ENSG00000281181 (-7291), RNA5-8SN1 (-5867), gene: ENSG00000286149 (+16477), gene: ENSG00000286032 (+19411), gene: ENSG00000286091 (+20623), gene: ENSG00000286148 (+22477)

30927-005.R1 (paired) (Report)

Host chromosome	Unaligned ends virus	Broken reads host	Disrupted genes	Nearby genes
21	N/A	26	gene: ENSG00000280441	gene: ENSG00000280441, MIR6724-3 (-57403), gene: ENSG00000274868 (-56869), gene: ENSG00000280614 (-51515), RNA5-8SN3 (-50097), gene: ENSG00000286267 (-27621), gene: ENSG00000286146 (-24853), gene: ENSG00000286054 (-23740), gene: ENSG00000286178 (-21785), MIR6724-4 (-13235), gene: ENSG00000278775 (-12682), gene: ENSG00000281181 (-7305), RNA5-8SN1 (-5881), gene: ENSG00000286149 (+16463), gene: ENSG00000286032 (+19397), gene: ENSG00000286091 (+20609), gene: ENSG00000286148 (+22463)

30927-005.R1 (paired) (Report)

Host chromosome	Unaligned ends virus	Broken reads host	Disrupted genes	Nearby genes
21	N/A	21	gene: ENSG00000280441	gene: ENSG00000280441, MIR6724-3 (-57422), gene: ENSG00000274868 (-56888), gene: ENSG00000280614 (-51534), RNA5-8SN3 (-50116), gene: ENSG00000286267 (-27640), gene: ENSG00000286146 (-24872), gene: ENSG00000286054 (-23759), gene: ENSG00000286178 (-21804), MIR6724-4 (-13254), gene: ENSG00000278775 (-12701), gene: ENSG00000281181 (-7324), RNA5-8SN1 (-5900), gene: ENSG00000286149 (+16444), gene: ENSG00000286032 (+19378), gene: ENSG00000286091 (+20590), gene: ENSG00000286148 (+22444)

30927-005.R1 (paired) (Report)

Host chromosome	Unaligned ends virus	Broken reads host	Disrupted genes	Nearby genes
21	4,126	21	gene: ENSG00000280441	gene: ENSG00000280441, MIR6724-3 (-57424), gene: ENSG00000274868 (-56890), gene: ENSG00000280614 (-51536), RNA5-8SN3 (-50118), gene: ENSG00000286267 (-27642), gene: ENSG00000286146 (-24874), gene: ENSG00000286054 (-23761), gene: ENSG00000286178 (-21806), MIR6724-4 (-13256), gene: ENSG00000278775 (-12703), gene: ENSG00000281181 (-7326), RNA5-8SN1 (-5902), gene: ENSG00000286149 (+16442), gene: ENSG00000286032 (+19376), gene: ENSG00000286091 (+20588), gene: ENSG00000286148 (+22442)

30927-005.R1 (paired) (Report)

Host chromosome	Unaligned ends virus	Broken reads host	Disrupted genes	Nearby genes
21	5,000	17	gene: ENSG00000280441	gene: ENSG00000280441, MIR6724-3 (-57444), gene: ENSG00000274868 (-56910), gene: ENSG00000280614 (-51556), RNA5-8SN3 (-50138), gene: ENSG00000286267 (-27662), gene: ENSG00000286146 (-24894), gene: ENSG00000286054 (-23781), gene: ENSG00000286178 (-21826), MIR6724-4 (-13276), gene: ENSG00000278775 (-12723), gene: ENSG00000281181 (-7346), RNA5-8SN1 (-5922), gene: ENSG00000286149 (+16422), gene: ENSG00000286032 (+19356), gene: ENSG00000286091 (+20568), gene: ENSG00000286148 (+22422)

30927-005.R1 (paired) (Report)

Host chromosome	Unaligned ends virus	Broken reads host	Disrupted genes	Nearby genes
21	N/A	16	gene: ENSG00000280441	gene: ENSG00000280441, MIR6724-3 (-57459), gene: ENSG00000274868 (-56925), gene: ENSG00000280614 (-51571), RNA5-8SN3 (-50153), gene: ENSG00000286267 (-27677), gene: ENSG00000286146 (-24909), gene: ENSG00000286054 (-23796), gene: ENSG00000286178 (-21841), MIR6724-4 (-13291), gene: ENSG00000278775 (-12738), gene: ENSG00000281181 (-7361), RNA5-8SN1 (-5937), gene: ENSG00000286149 (+16407), gene: ENSG00000286032 (+19341), gene: ENSG00000286091 (+20553), gene: ENSG00000286148 (+22407)

30927-005.R1 (paired) (Report)

Host chromosome	Unaligned ends virus	Broken reads host	Disrupted genes	Nearby genes
21	N/A	16	gene: ENSG00000280441	gene: ENSG00000280441, MIR6724-3 (-57460), gene: ENSG00000274868 (-56926), gene: ENSG00000280614 (-51572), RNA5-8SN3 (-50154), gene: ENSG00000286267 (-27678), gene: ENSG00000286146 (-24910), gene: ENSG00000286054 (-23797), gene: ENSG00000286178 (-21842), MIR6724-4 (-13292), gene: ENSG00000278775 (-12739), gene: ENSG00000281181 (-7362), RNA5-8SN1 (-5938), gene: ENSG00000286149 (+16406), gene: ENSG00000286032 (+19340), gene: ENSG00000286091 (+20552), gene: ENSG00000286148 (+22406)

30927-005.R1 (paired) (Report)

Host chromosome	Unaligned ends virus	Broken reads host	Disrupted genes	Nearby genes
21	61,253	15	gene: ENSG00000280441	gene: ENSG00000280441, MIR6724-3 (-57464), gene: ENSG00000274868 (-56930), gene: ENSG00000280614 (-51576), RNA5-8SN3 (-50158), gene: ENSG00000286267 (-27682), gene: ENSG00000286146 (-24914), gene: ENSG00000286054 (-23801), gene: ENSG00000286178 (-21846), MIR6724-4 (-13296), gene: ENSG00000278775 (-12743), gene: ENSG00000281181 (-7366), RNA5-8SN1 (-5942), gene: ENSG00000286149 (+16402), gene: ENSG00000286032 (+19336), gene: ENSG00000286091 (+20548), gene: ENSG00000286148 (+22402)

30927-005.R1 (paired) (Report)

Host chromosome	Unaligned ends virus	Broken reads host	Disrupted genes	Nearby genes
21	7,291	14	gene: ENSG00000280441	gene: ENSG00000280441, MIR6724-3 (-57466), gene: ENSG00000274868 (-56932), gene: ENSG00000280614 (-51578), RNA5-8SN3 (-50160), gene: ENSG00000286267 (-27684), gene: ENSG00000286146 (-24916), gene: ENSG00000286054 (-23803), gene: ENSG00000286178 (-21848), MIR6724-4 (-13298), gene: ENSG00000278775 (-12745), gene: ENSG00000281181 (-7368), RNA5-8SN1 (-5944), gene: ENSG00000286149 (+16400), gene: ENSG00000286032 (+19334), gene: ENSG00000286091 (+20546), gene: ENSG00000286148 (+22400)

30927-005.R1 (paired) (Report)

Host chromosome	Unaligned ends virus	Broken reads host	Disrupted genes	Nearby genes
21	N/A	8	gene: ENSG00000280441	gene: ENSG00000280441, MIR6724-3 (-57503), gene: ENSG00000274868 (-56969), gene: ENSG00000280614 (-51615), RNA5-8SN3 (-50197), gene: ENSG00000286267 (-27721), gene: ENSG00000286146 (-24953), gene: ENSG00000286054 (-23840), gene: ENSG00000286178 (-21885), MIR6724-4 (-13335), gene: ENSG00000278775 (-12782), gene: ENSG00000281181 (-7405), RNA5-8SN1 (-5981), gene: ENSG00000286149 (+16363), gene: ENSG00000286032 (+19297), gene: ENSG00000286091 (+20509), gene: ENSG00000286148 (+22363)

30927-005.R1 (paired) (Report)

Host chromosome	Unaligned ends virus	Broken reads host	Disrupted genes	Nearby genes
21	N/A	5	gene: ENSG00000280441	gene: ENSG00000280441, MIR6724-3 (-57535), gene: ENSG00000274868 (-57001), gene: ENSG00000280614 (-51647), RNA5-8SN3 (-50229), gene: ENSG00000286267 (-27753), gene: ENSG00000286146 (-24985), gene: ENSG00000286054 (-23872), gene: ENSG00000286178 (-21917), MIR6724-4 (-13367), gene: ENSG00000278775 (-12814), gene: ENSG00000281181 (-7437), RNA5-8SN1 (-6013), gene: ENSG00000286149 (+16331), gene: ENSG00000286032 (+19265), gene: ENSG00000286091 (+20477), gene: ENSG00000286148 (+22331)

30927-005.R1 (paired) (Report)

Host chromosome	Unaligned ends virus	Broken reads host	Disrupted genes	Nearby genes
21	N/A	3	gene: ENSG00000280441	gene: ENSG00000280441, MIR6724-3 (-57542), gene: ENSG00000274868 (-57008), gene: ENSG00000280614 (-51654), RNA5-8SN3 (-50236), gene: ENSG00000286267 (-27760), gene: ENSG00000286146 (-24992), gene: ENSG00000286054 (-23879), gene: ENSG00000286178 (-21924), MIR6724-4 (-13374), gene: ENSG00000278775 (-12821), gene: ENSG00000281181 (-7444), RNA5-8SN1 (-6020), gene: ENSG00000286149 (+16324), gene: ENSG00000286032 (+19258), gene: ENSG00000286091 (+20470), gene: ENSG00000286148 (+22324)

30927-005.R1 (paired) (Report)

Host chromosome	Unaligned ends virus	Broken reads host	Disrupted genes	Nearby genes
21	N/A	16		gene: ENSG00000278996 (-31994), MIR6724-1 (-54234), gene: ENSG00000275664 (-53700), MIR3648-1 (-50988), gene: ENSG00000277437 (-50736), gene: ENSG00000280800 (-48334), RNA5-8SN2 (-46916), gene: ENSG00000286155 (-23808), gene: ENSG00000286252 (-21661), gene: ENSG00000286057 (-20548), gene: ENSG00000286012 (-18593), MIR6724-2 (-10044), gene: ENSG00000277671 (-9491), gene: ENSG00000281383 (-4126), 5_8S_rRNA (-2707)
21	N/A	21		gene: ENSG00000278996 (-32049), MIR6724-1 (-54289), gene: ENSG00000275664 (-53755), MIR3648-1 (-51043), gene: ENSG00000277437 (-50791), gene: ENSG00000280800 (-48389), RNA5-8SN2 (-46971), gene: ENSG00000286155 (-23863), gene: ENSG00000286252 (-21716), gene: ENSG00000286057 (-20603), gene: ENSG00000286012 (-18648), MIR6724-2 (-10099), gene: ENSG00000277671 (-9546), gene: ENSG00000281383 (-4181), 5_8S_rRNA (-2762)

30927-005.R1 (paired) (Report)

Host chromosome	Unaligned ends virus	Broken reads host	Disrupted genes	Nearby genes
21	6,702	24		gene: ENSG00000278996 (-32059), MIR6724-1 (-54299), gene: ENSG00000275664 (-53765), MIR3648-1 (-51053), gene: ENSG00000277437 (-50801), gene: ENSG00000280800 (-48399), RNA5-8SN2 (-46981), gene: ENSG00000286155 (-23873), gene: ENSG00000286252 (-21726), gene: ENSG00000286057 (-20613), gene: ENSG00000286012 (-18658), MIR6724-2 (-10109), gene: ENSG00000277671 (-9556), gene: ENSG00000281383 (-4191), 5_8S_rRNA (-2772)
21	4,505	42		gene: ENSG00000278996 (-32433), MIR6724-1 (-54673), gene: ENSG00000275664 (-54139), MIR3648-1 (-51427), gene: ENSG00000277437 (-51175), gene: ENSG00000280800 (-48773), RNA5-8SN2 (-47355), gene: ENSG00000286155 (-24247), gene: ENSG00000286252 (-22100), gene: ENSG00000286057 (-20987), gene: ENSG00000286012 (-19032), MIR6724-2 (-10483), gene: ENSG00000277671 (-9930), gene: ENSG00000281383 (-4565), 5_8S_rRNA (-3146)

30927-005.R1 (paired) (Report)

Host chromosome	Unaligned ends virus	Broken reads host	Disrupted genes	Nearby genes
21	3,951	6	gene: ENSG00000280441	gene: ENSG00000280441, MIR6724-3 (-9992), gene: ENSG00000274868 (-9458), gene: ENSG00000280614 (-4104), RNA5-8SN3 (-2686), gene: ENSG00000286267 (+19311), gene: ENSG00000286146 (+22229), gene: ENSG00000286054 (+23437), gene: ENSG00000286178 (+25314), MIR6724-4 (+34084), gene: ENSG00000278775 (+34639), gene: ENSG00000281181 (+39183), RNA5-8SN1 (+41377), gene: ENSG00000286149 (+63874), gene: ENSG00000286032 (+66808), gene: ENSG00000286091 (+68020), gene: ENSG00000286148 (+69874)

30927-005.R1 (paired) (Report)

Host chromosome	Unaligned ends virus	Broken reads host	Disrupted genes	Nearby genes
21	N/A	8	gene: ENSG00000280441	gene: ENSG00000280441, MIR6724-3 (-10047), gene: ENSG00000274868 (-9513), gene: ENSG00000280614 (-4159), RNA5-8SN3 (-2741), gene: ENSG00000286267 (+19256), gene: ENSG00000286146 (+22174), gene: ENSG00000286054 (+23382), gene: ENSG00000286178 (+25259), MIR6724-4 (+34029), gene: ENSG00000278775 (+34584), gene: ENSG00000281181 (+39128), RNA5-8SN1 (+41322), gene: ENSG00000286149 (+63819), gene: ENSG00000286032 (+66753), gene: ENSG00000286091 (+67965), gene: ENSG00000286148 (+69819)

30927-005.R1 (paired) (Report)

Host chromosome	Unaligned ends virus	Broken reads host	Disrupted genes	Nearby genes
21	N/A	22	gene: ENSG00000280441	gene: ENSG00000280441, MIR6724-3 (-10207), gene: ENSG00000274868 (-9673), gene: ENSG00000280614 (-4319), RNA5-8SN3 (-2901), gene: ENSG00000286267 (+19096), gene: ENSG00000286146 (+22014), gene: ENSG00000286054 (+23222), gene: ENSG00000286178 (+25099), MIR6724-4 (+33869), gene: ENSG00000278775 (+34424), gene: ENSG00000281181 (+38968), RNA5-8SN1 (+41162), gene: ENSG00000286149 (+63659), gene: ENSG00000286032 (+66593), gene: ENSG00000286091 (+67805), gene: ENSG00000286148 (+69659)

30927-005.R1 (paired) (Report)

Host chromosome	Unaligned ends virus	Broken reads host	Disrupted genes	Nearby genes
21	N/A	29	gene: ENSG00000280441	gene: ENSG00000280441, MIR6724-3 (-10431), gene: ENSG00000274868 (-9897), gene: ENSG00000280614 (-4543), RNA5-8SN3 (-3125), gene: ENSG00000286267 (+18872), gene: ENSG00000286146 (+21790), gene: ENSG00000286054 (+22998), gene: ENSG00000286178 (+24875), MIR6724-4 (+33645), gene: ENSG00000278775 (+34200), gene: ENSG00000281181 (+38744), RNA5-8SN1 (+40938), gene: ENSG00000286149 (+63435), gene: ENSG00000286032 (+66369), gene: ENSG00000286091 (+67581), gene: ENSG00000286148 (+69435)

30927-005.R1 (paired) (Report)

Host chromosome	Unaligned ends virus	Broken reads host	Disrupted genes	Nearby genes
21	N/A	13	gene: ENSG00000280441	gene: ENSG00000280441, MIR6724-3 (-11057), gene: ENSG00000274868 (-10523), gene: ENSG00000280614 (-5169), RNA5-8SN3 (-3751), gene: ENSG00000286267 (+18246), gene: ENSG00000286146 (+21164), gene: ENSG00000286054 (+22372), gene: ENSG00000286178 (+24249), MIR6724-4 (+33019), gene: ENSG00000278775 (+33574), gene: ENSG00000281181 (+38118), RNA5-8SN1 (+40312), gene: ENSG00000286149 (+62809), gene: ENSG00000286032 (+65743), gene: ENSG00000286091 (+66955), gene: ENSG00000286148 (+68809)

30927-005.R1 (paired) (Report)

Host chromosome	Unaligned ends virus	Broken reads host	Disrupted genes	Nearby genes
21	4,442	21	gene: ENSG00000280441	gene: ENSG00000280441, MIR6724-3 (-11207), gene: ENSG00000274868 (-10673), gene: ENSG00000280614 (-5319), RNA5-8SN3 (-3901), gene: ENSG00000286267 (+18096), gene: ENSG00000286146 (+21014), gene: ENSG00000286054 (+22222), gene: ENSG00000286178 (+24099), MIR6724-4 (+32869), gene: ENSG00000278775 (+33424), gene: ENSG00000281181 (+37968), RNA5-8SN1 (+40162), gene: ENSG00000286149 (+62659), gene: ENSG00000286032 (+65593), gene: ENSG00000286091 (+66805), gene: ENSG00000286148 (+68659)

30927-005.R1 (paired) (Report)

Host chromosome	Unaligned ends virus	Broken reads host	Disrupted genes	Nearby genes
21	N/A	19	gene: ENSG00000278996, gene: ENSG00000280800	gene: ENSG00000278996, MIR6724-1 (-5872), gene: ENSG00000275664 (-5338), MIR3648-1 (-2626), gene: ENSG00000277437 (-2374), gene: ENSG00000280800, RNA5-8SN2 (+1293), gene: ENSG00000286155 (+24075), gene: ENSG00000286252 (+26370), gene: ENSG00000286057 (+27580), gene: ENSG00000286012 (+29457), MIR6724-2 (+38226), gene: ENSG00000277671 (+38781), gene: ENSG00000281383 (+43313), 5_8S_rRNA (+45502)
21	4,442	22	gene: ENSG00000278996	gene: ENSG00000278996, MIR6724-1 (-9064), gene: ENSG00000275664 (-8530), MIR3648-1 (-5818), gene: ENSG00000277437 (-5566), gene: ENSG00000280800 (-3164), RNA5-8SN2 (-1746), gene: ENSG00000286155 (+20883), gene: ENSG00000286252 (+23178), gene: ENSG00000286057 (+24388), gene: ENSG00000286012 (+26265), MIR6724-2 (+35034), gene: ENSG00000277671 (+35589), gene: ENSG00000281383 (+40121), 5_8S_rRNA (+42310)

30927-005.R1 (paired) (Report)

Host chromosome	Unaligned ends virus	Broken reads host	Disrupted genes	Nearby genes
21	3,551	28	gene: ENSG00000278996	gene: ENSG00000278996, MIR6724-1 (-10057), gene: ENSG00000275664 (-9523), MIR3648-1 (-6811), gene: ENSG00000277437 (-6559), gene: ENSG00000280800 (-4157), RNA5-8SN2 (-2739), gene: ENSG00000286155 (+19890), gene: ENSG00000286252 (+22185), gene: ENSG00000286057 (+23395), gene: ENSG00000286012 (+25272), MIR6724-2 (+34041), gene: ENSG00000277671 (+34596), gene: ENSG00000281383 (+39128), 5_8S_rRNA (+41317)
21	N/A	29	gene: ENSG00000278996	gene: ENSG00000278996, MIR6724-1 (-10060), gene: ENSG00000275664 (-9526), MIR3648-1 (-6814), gene: ENSG00000277437 (-6562), gene: ENSG00000280800 (-4160), RNA5-8SN2 (-2742), gene: ENSG00000286155 (+19887), gene: ENSG00000286252 (+22182), gene: ENSG00000286057 (+23392), gene: ENSG00000286012 (+25269), MIR6724-2 (+34038), gene: ENSG00000277671 (+34593), gene: ENSG00000281383 (+39125), 5_8S_rRNA (+41314)

30927-005.R1 (paired) (Report)

Host chromosome	Unaligned ends virus	Broken reads host	Disrupted genes	Nearby genes
21	N/A	36	gene: ENSG00000278996	gene: ENSG00000278996, MIR6724-1 (-10444), gene: ENSG00000275664 (-9910), MIR3648-1 (-7198), gene: ENSG00000277437 (-6946), gene: ENSG00000280800 (-4544), RNA5-8SN2 (-3126), gene: ENSG00000286155 (+19503), gene: ENSG00000286252 (+21798), gene: ENSG00000286057 (+23008), gene: ENSG00000286012 (+24885), MIR6724-2 (+33654), gene: ENSG00000277671 (+34209), gene: ENSG00000281383 (+38741), 5_8S_rRNA (+40930)
21	2,101	27	gene: ENSG00000278996	gene: ENSG00000278996, MIR6724-1 (-13396), gene: ENSG00000275664 (-12862), MIR3648-1 (-10150), gene: ENSG00000277437 (-9898), gene: ENSG00000280800 (-7496), RNA5-8SN2 (-6078), gene: ENSG00000286155 (+16551), gene: ENSG00000286252 (+18846), gene: ENSG00000286057 (+20056), gene: ENSG00000286012 (+21933), MIR6724-2 (+30702), gene: ENSG00000277671 (+31257), gene: ENSG00000281383 (+35789), 5_8S_rRNA (+37978)

30927-005.R1 (paired) (Report)

Host chromosome	Unaligned ends virus	Broken reads host	Disrupted genes	Nearby genes
21	2,101	32	gene: ENSG00000278996	gene: ENSG00000278996, MIR6724-1 (-13403), gene: ENSG00000275664 (-12869), MIR3648-1 (-10157), gene: ENSG00000277437 (-9905), gene: ENSG00000280800 (-7503), RNA5-8SN2 (-6085), gene: ENSG00000286155 (+16544), gene: ENSG00000286252 (+18839), gene: ENSG00000286057 (+20049), gene: ENSG00000286012 (+21926), MIR6724-2 (+30695), gene: ENSG00000277671 (+31250), gene: ENSG00000281383 (+35782), 5_8S_rRNA (+37971)
21	2,822	34	gene: ENSG00000278996	gene: ENSG00000278996, MIR6724-1 (-13405), gene: ENSG00000275664 (-12871), MIR3648-1 (-10159), gene: ENSG00000277437 (-9907), gene: ENSG00000280800 (-7505), RNA5-8SN2 (-6087), gene: ENSG00000286155 (+16542), gene: ENSG00000286252 (+18837), gene: ENSG00000286057 (+20047), gene: ENSG00000286012 (+21924), MIR6724-2 (+30693), gene: ENSG00000277671 (+31248), gene: ENSG00000281383 (+35780), 5_8S_rRNA (+37969)

30927-005.R1 (paired) (Report)

Host chromosome	Unaligned ends virus	Broken reads host	Disrupted genes	Nearby genes
21	N/A	36	gene: ENSG00000278996	gene: ENSG00000278996, MIR6724-1 (-13408), gene: ENSG00000275664 (-12874), MIR3648-1 (-10162), gene: ENSG00000277437 (-9910), gene: ENSG00000280800 (-7508), RNA5-8SN2 (-6090), gene: ENSG00000286155 (+16539), gene: ENSG00000286252 (+18834), gene: ENSG00000286057 (+20044), gene: ENSG00000286012 (+21921), MIR6724-2 (+30690), gene: ENSG00000277671 (+31245), gene: ENSG00000281383 (+35777), 5_8S_rRNA (+37966)
21	N/A	45	gene: ENSG00000278996	gene: ENSG00000278996, MIR6724-1 (-13414), gene: ENSG00000275664 (-12880), MIR3648-1 (-10168), gene: ENSG00000277437 (-9916), gene: ENSG00000280800 (-7514), RNA5-8SN2 (-6096), gene: ENSG00000286155 (+16533), gene: ENSG00000286252 (+18828), gene: ENSG00000286057 (+20038), gene: ENSG00000286012 (+21915), MIR6724-2 (+30684), gene: ENSG00000277671 (+31239), gene: ENSG00000281383 (+35771), 5_8S_rRNA (+37960)

30927-005.R1 (paired) (Report)

Host chromosome	Unaligned ends virus	Broken reads host	Disrupted genes	Nearby genes
21	N/A	52	gene: ENSG00000278996	gene: ENSG00000278996, MIR6724-1 (-13445), gene: ENSG00000275664 (-12911), MIR3648-1 (-10199), gene: ENSG00000277437 (-9947), gene: ENSG00000280800 (-7545), RNA5-8SN2 (-6127), gene: ENSG00000286155 (+16502), gene: ENSG00000286252 (+18797), gene: ENSG00000286057 (+20007), gene: ENSG00000286012 (+21884), MIR6724-2 (+30653), gene: ENSG00000277671 (+31208), gene: ENSG00000281383 (+35740), 5_8S_rRNA (+37929)
21	N/A	55	gene: ENSG00000278996	gene: ENSG00000278996, MIR6724-1 (-13465), gene: ENSG00000275664 (-12931), MIR3648-1 (-10219), gene: ENSG00000277437 (-9967), gene: ENSG00000280800 (-7565), RNA5-8SN2 (-6147), gene: ENSG00000286155 (+16482), gene: ENSG00000286252 (+18777), gene: ENSG00000286057 (+19987), gene: ENSG00000286012 (+21864), MIR6724-2 (+30633), gene: ENSG00000277671 (+31188), gene: ENSG00000281383 (+35720), 5_8S_rRNA (+37909)

30927-005.R1 (paired) (Report)

Host chromosome	Unaligned ends virus	Broken reads host	Disrupted genes	Nearby genes
21	N/A	19	gene: ENSG00000280441, gene: ENSG00000281181	gene: ENSG00000280441, MIR6724-3 (-50070), gene: ENSG00000274868 (-49536), gene: ENSG00000280614 (-44182), RNA5-8SN3 (-42764), gene: ENSG00000286267 (-20288), gene: ENSG00000286146 (-17520), gene: ENSG00000286054 (-16407), gene: ENSG00000286178 (-14452), MIR6724-4 (-5902), gene: ENSG00000278775 (-5349), gene: ENSG00000281181, RNA5-8SN1 (+1299), gene: ENSG00000286149 (+23796), gene: ENSG00000286032 (+26730), gene: ENSG00000286091 (+27942), gene: ENSG00000286148 (+29796)

30927-005.R1 (paired) (Report)

Host chromosome	Unaligned ends virus	Broken reads host	Disrupted genes	Nearby genes
21	4,442	20	gene: ENSG00000280441	gene: ENSG00000280441, MIR6724-3 (-53944), gene: ENSG00000274868 (-53410), gene: ENSG00000280614 (-48056), RNA5-8SN3 (-46638), gene: ENSG00000286267 (-24162), gene: ENSG00000286146 (-21394), gene: ENSG00000286054 (-20281), gene: ENSG00000286178 (-18326), MIR6724-4 (-9776), gene: ENSG00000278775 (-9223), gene: ENSG00000281181 (-3846), RNA5-8SN1 (-2422), gene: ENSG00000286149 (+19922), gene: ENSG00000286032 (+22856), gene: ENSG00000286091 (+24068), gene: ENSG00000286148 (+25922)

30927-005.R1 (paired) (Report)

Host chromosome	Unaligned ends virus	Broken reads host	Disrupted genes	Nearby genes
21	N/A	36	gene: ENSG00000280441	gene: ENSG00000280441, MIR6724-3 (-54002), gene: ENSG00000274868 (-53468), gene: ENSG00000280614 (-48114), RNA5-8SN3 (-46696), gene: ENSG00000286267 (-24220), gene: ENSG00000286146 (-21452), gene: ENSG00000286054 (-20339), gene: ENSG00000286178 (-18384), MIR6724-4 (-9834), gene: ENSG00000278775 (-9281), gene: ENSG00000281181 (-3904), RNA5-8SN1 (-2480), gene: ENSG00000286149 (+19864), gene: ENSG00000286032 (+22798), gene: ENSG00000286091 (+24010), gene: ENSG00000286148 (+25864)

30927-005.R1 (paired) (Report)

Host chromosome	Unaligned ends virus	Broken reads host	Disrupted genes	Nearby genes
21	N/A	37	gene: ENSG00000280441	gene: ENSG00000280441, MIR6724-3 (-54003), gene: ENSG00000274868 (-53469), gene: ENSG00000280614 (-48115), RNA5-8SN3 (-46697), gene: ENSG00000286267 (-24221), gene: ENSG00000286146 (-21453), gene: ENSG00000286054 (-20340), gene: ENSG00000286178 (-18385), MIR6724-4 (-9835), gene: ENSG00000278775 (-9282), gene: ENSG00000281181 (-3905), RNA5-8SN1 (-2481), gene: ENSG00000286149 (+19863), gene: ENSG00000286032 (+22797), gene: ENSG00000286091 (+24009), gene: ENSG00000286148 (+25863)

30927-005.R1 (paired) (Report)

Host chromosome	Unaligned ends virus	Broken reads host	Disrupted genes	Nearby genes
21	6,188	37	gene: ENSG00000280441	gene: ENSG00000280441, MIR6724-3 (-54004), gene: ENSG00000274868 (-53470), gene: ENSG00000280614 (-48116), RNA5-8SN3 (-46698), gene: ENSG00000286267 (-24222), gene: ENSG00000286146 (-21454), gene: ENSG00000286054 (-20341), gene: ENSG00000286178 (-18386), MIR6724-4 (-9836), gene: ENSG00000278775 (-9283), gene: ENSG00000281181 (-3906), RNA5-8SN1 (-2482), gene: ENSG00000286149 (+19862), gene: ENSG00000286032 (+22796), gene: ENSG00000286091 (+24008), gene: ENSG00000286148 (+25862)

30927-005.R1 (paired) (Report)

Host chromosome	Unaligned ends virus	Broken reads host	Disrupted genes	Nearby genes
21	N/A	37	gene: ENSG00000280441	gene: ENSG00000280441, MIR6724-3 (-54005), gene: ENSG00000274868 (-53471), gene: ENSG00000280614 (-48117), RNA5-8SN3 (-46699), gene: ENSG00000286267 (-24223), gene: ENSG00000286146 (-21455), gene: ENSG00000286054 (-20342), gene: ENSG00000286178 (-18387), MIR6724-4 (-9837), gene: ENSG00000278775 (-9284), gene: ENSG00000281181 (-3907), RNA5-8SN1 (-2483), gene: ENSG00000286149 (+19861), gene: ENSG00000286032 (+22795), gene: ENSG00000286091 (+24007), gene: ENSG00000286148 (+25861)

30927-005.R1 (paired) (Report)

Host chromosome	Unaligned ends virus	Broken reads host	Disrupted genes	Nearby genes
21	3,510	37	gene: ENSG00000280441	gene: ENSG00000280441, MIR6724-3 (-54007), gene: ENSG00000274868 (-53473), gene: ENSG00000280614 (-48119), RNA5-8SN3 (-46701), gene: ENSG00000286267 (-24225), gene: ENSG00000286146 (-21457), gene: ENSG00000286054 (-20344), gene: ENSG00000286178 (-18389), MIR6724-4 (-9839), gene: ENSG00000278775 (-9286), gene: ENSG00000281181 (-3909), RNA5-8SN1 (-2485), gene: ENSG00000286149 (+19859), gene: ENSG00000286032 (+22793), gene: ENSG00000286091 (+24005), gene: ENSG00000286148 (+25859)

30927-005.R1 (paired) (Report)

Host chromosome	Unaligned ends virus	Broken reads host	Disrupted genes	Nearby genes
21	4,442	37	gene: ENSG00000280441	gene: ENSG00000280441, MIR6724-3 (-54006), gene: ENSG00000274868 (-53472), gene: ENSG00000280614 (-48118), RNA5-8SN3 (-46700), gene: ENSG00000286267 (-24224), gene: ENSG00000286146 (-21456), gene: ENSG00000286054 (-20343), gene: ENSG00000286178 (-18388), MIR6724-4 (-9838), gene: ENSG00000278775 (-9285), gene: ENSG00000281181 (-3908), RNA5-8SN1 (-2484), gene: ENSG00000286149 (+19860), gene: ENSG00000286032 (+22794), gene: ENSG00000286091 (+24006), gene: ENSG00000286148 (+25860)

30927-005.R1 (paired) (Report)

Host chromosome	Unaligned ends virus	Broken reads host	Disrupted genes	Nearby genes
21	N/A	37	gene: ENSG00000280441	gene: ENSG00000280441, MIR6724-3 (-54008), gene: ENSG00000274868 (-53474), gene: ENSG00000280614 (-48120), RNA5-8SN3 (-46702), gene: ENSG00000286267 (-24226), gene: ENSG00000286146 (-21458), gene: ENSG00000286054 (-20345), gene: ENSG00000286178 (-18390), MIR6724-4 (-9840), gene: ENSG00000278775 (-9287), gene: ENSG00000281181 (-3910), RNA5-8SN1 (-2486), gene: ENSG00000286149 (+19858), gene: ENSG00000286032 (+22792), gene: ENSG00000286091 (+24004), gene: ENSG00000286148 (+25858)

30927-005.R1 (paired) (Report)

Host chromosome	Unaligned ends virus	Broken reads host	Disrupted genes	Nearby genes
21	N/A	51	gene: ENSG00000280441	gene: ENSG00000280441, MIR6724-3 (-54221), gene: ENSG00000274868 (-53687), gene: ENSG00000280614 (-48333), RNA5-8SN3 (-46915), gene: ENSG00000286267 (-24439), gene: ENSG00000286146 (-21671), gene: ENSG00000286054 (-20558), gene: ENSG00000286178 (-18603), MIR6724-4 (-10053), gene: ENSG00000278775 (-9500), gene: ENSG00000281181 (-4123), RNA5-8SN1 (-2699), gene: ENSG00000286149 (+19645), gene: ENSG00000286032 (+22579), gene: ENSG00000286091 (+23791), gene: ENSG00000286148 (+25645)

30927-005.R1 (paired) (Report)

Host chromosome	Unaligned ends virus	Broken reads host	Disrupted genes	Nearby genes
21	4,126	60	gene: ENSG00000280441	gene: ENSG00000280441, MIR6724-3 (-54273), gene: ENSG00000274868 (-53739), gene: ENSG00000280614 (-48385), RNA5-8SN3 (-46967), gene: ENSG00000286267 (-24491), gene: ENSG00000286146 (-21723), gene: ENSG00000286054 (-20610), gene: ENSG00000286178 (-18655), MIR6724-4 (-10105), gene: ENSG00000278775 (-9552), gene: ENSG00000281181 (-4175), RNA5-8SN1 (-2751), gene: ENSG00000286149 (+19593), gene: ENSG00000286032 (+22527), gene: ENSG00000286091 (+23739), gene: ENSG00000286148 (+25593)

30927-005.R1 (paired) (Report)

Host chromosome	Unaligned ends virus	Broken reads host	Disrupted genes	Nearby genes
21	N/A	62	gene: ENSG00000280441	gene: ENSG00000280441, MIR6724-3 (-54286), gene: ENSG00000274868 (-53752), gene: ENSG00000280614 (-48398), RNA5-8SN3 (-46980), gene: ENSG00000286267 (-24504), gene: ENSG00000286146 (-21736), gene: ENSG00000286054 (-20623), gene: ENSG00000286178 (-18668), MIR6724-4 (-10118), gene: ENSG00000278775 (-9565), gene: ENSG00000281181 (-4188), RNA5-8SN1 (-2764), gene: ENSG00000286149 (+19580), gene: ENSG00000286032 (+22514), gene: ENSG00000286091 (+23726), gene: ENSG00000286148 (+25580)

30927-005.R1 (paired) (Report)

Host chromosome	Unaligned ends virus	Broken reads host	Disrupted genes	Nearby genes
21	N/A	26	gene: ENSG00000280441	gene: ENSG00000280441, MIR6724-3 (-54873), gene: ENSG00000274868 (-54339), gene: ENSG00000280614 (-48985), RNA5-8SN3 (-47567), gene: ENSG00000286267 (-25091), gene: ENSG00000286146 (-22323), gene: ENSG00000286054 (-21210), gene: ENSG00000286178 (-19255), MIR6724-4 (-10705), gene: ENSG00000278775 (-10152), gene: ENSG00000281181 (-4775), RNA5-8SN1 (-3351), gene: ENSG00000286149 (+18993), gene: ENSG00000286032 (+21927), gene: ENSG00000286091 (+23139), gene: ENSG00000286148 (+24993)

30927-005.R1 (paired) (Report)

Host chromosome	Unaligned ends virus	Broken reads host	Disrupted genes	Nearby genes
21	4,442	26	gene: ENSG00000280441	gene: ENSG00000280441, MIR6724-3 (-54943), gene: ENSG00000274868 (-54409), gene: ENSG00000280614 (-49055), RNA5-8SN3 (-47637), gene: ENSG00000286267 (-25161), gene: ENSG00000286146 (-22393), gene: ENSG00000286054 (-21280), gene: ENSG00000286178 (-19325), MIR6724-4 (-10775), gene: ENSG00000278775 (-10222), gene: ENSG00000281181 (-4845), RNA5-8SN1 (-3421), gene: ENSG00000286149 (+18923), gene: ENSG00000286032 (+21857), gene: ENSG00000286091 (+23069), gene: ENSG00000286148 (+24923)

30927-005.R1 (paired) (Report)

Host chromosome	Unaligned ends virus	Broken reads host	Disrupted genes	Nearby genes
21	2,822	18	gene: ENSG00000280441	gene: ENSG00000280441, MIR6724-3 (-57628), gene: ENSG00000274868 (-57094), gene: ENSG00000280614 (-51740), RNA5-8SN3 (-50322), gene: ENSG00000286267 (-27846), gene: ENSG00000286146 (-25078), gene: ENSG00000286054 (-23965), gene: ENSG00000286178 (-22010), MIR6724-4 (-13460), gene: ENSG00000278775 (-12907), gene: ENSG00000281181 (-7530), RNA5-8SN1 (-6106), gene: ENSG00000286149 (+16238), gene: ENSG00000286032 (+19172), gene: ENSG00000286091 (+20384), gene: ENSG00000286148 (+22238)

30927-005.R1 (paired) (Report)

Host chromosome	Unaligned ends virus	Broken reads host	Disrupted genes	Nearby genes
21	N/A	24	gene: ENSG00000280441	gene: ENSG00000280441, MIR6724-3 (-57637), gene: ENSG00000274868 (-57103), gene: ENSG00000280614 (-51749), RNA5-8SN3 (-50331), gene: ENSG00000286267 (-27855), gene: ENSG00000286146 (-25087), gene: ENSG00000286054 (-23974), gene: ENSG00000286178 (-22019), MIR6724-4 (-13469), gene: ENSG00000278775 (-12916), gene: ENSG00000281181 (-7539), RNA5-8SN1 (-6115), gene: ENSG00000286149 (+16229), gene: ENSG00000286032 (+19163), gene: ENSG00000286091 (+20375), gene: ENSG00000286148 (+22229)

30927-005.R1 (paired) (Report)

Host chromosome	Unaligned ends virus	Broken reads host	Disrupted genes	Nearby genes
21	3,951	24	gene: ENSG00000280441	gene: ENSG00000280441, MIR6724-3 (-57639), gene: ENSG00000274868 (-57105), gene: ENSG00000280614 (-51751), RNA5-8SN3 (-50333), gene: ENSG00000286267 (-27857), gene: ENSG00000286146 (-25089), gene: ENSG00000286054 (-23976), gene: ENSG00000286178 (-22021), MIR6724-4 (-13471), gene: ENSG00000278775 (-12918), gene: ENSG00000281181 (-7541), RNA5-8SN1 (-6117), gene: ENSG00000286149 (+16227), gene: ENSG00000286032 (+19161), gene: ENSG00000286091 (+20373), gene: ENSG00000286148 (+22227)
22	N/A	1	PPIL2	UBE2L3 (-71678), YDJC (-65648), CCDC116 (-58383), gene: ENSG00000273342 (-54428), SDF2L1 (-51413), gene: ENSG00000207751 (-25475), MIR301B (-42654), MIR130B (-42327), gene: ENSG00000272954 (-33349), PPIL2, YPEL1 (+1823), gene: ENSG00000286365 (+17048), RN7SL280P (+27182), gene: ENSG00000286127 (+41735), MAPK1 (+63944)

30927-005.R1 (paired) (Report)

Host chromosome	Unaligned ends virus	Broken reads host	Disrupted genes	Nearby genes
22	3,426	1	PPIL2	UBE2L3 (-71713), YDJC (-65683), CCDC116 (-58418), gene: ENSG00000273342 (-54463), SDF2L1 (-51448), gene: ENSG00000207751 (-25510), MIR301B (-42689), MIR130B (-42362), gene: ENSG00000272954 (-33384), PPIL2, YPEL1 (+1788), gene: ENSG00000286365 (+17013), RN7SL280P (+27147), gene: ENSG00000286127 (+41700), MAPK1 (+63909)
22	N/A	1	PPIL2	UBE2L3 (-71781), YDJC (-65751), CCDC116 (-58486), gene: ENSG00000273342 (-54531), SDF2L1 (-51516), gene: ENSG00000207751 (-25578), MIR301B (-42757), MIR130B (-42430), gene: ENSG00000272954 (-33452), PPIL2, YPEL1 (+1720), gene: ENSG00000286365 (+16945), RN7SL280P (+27079), gene: ENSG00000286127 (+41632), MAPK1 (+63841)

30927-005.R1 (paired) (Report)

Host chromosome	Unaligned ends virus	Broken reads host	Disrupted genes	Nearby genes
22	N/A	1	PPIL2	UBE2L3 (-71802), YDJC (-65772), CCDC116 (-58507), gene: ENSG00000273342 (-54552), SDF2L1 (-51537), gene: ENSG00000207751 (-25599), MIR301B (-42778), MIR130B (-42451), gene: ENSG00000272954 (-33473), PPIL2, YPEL1 (+1699), gene: ENSG00000286365 (+16924), RN7SL280P (+27058), gene: ENSG00000286127 (+41611), MAPK1 (+63820)
X	N/A	62	DACH2	DACH2
X	N/A	50	DACH2	DACH2

30927-006.R1 (paired) (Report)

1. Identify Viral Integration Sites summary

Input reads	2,750,036
Host reads	1,944,776
Virus reads	55,090
Unmapped reads	750,170
Host reads (%)	70.72
Virus reads (%)	2.00
Unmapped reads (%)	27.28
Breakpoints identified	75
Viruses identified	1

2. Identify Viral Integration Sites virus content

Virus	Reads mapped
HPV16REF	28,140

3. Identify Viral Integration Sites breakpoint summary

Host chromosome	Host region	Virus	Virus region	Unaligned ends host
12	complement (132571066.. 132571067)	HPV16REF	7192..7193	1,329
12	132571231.. 132571232	HPV16REF	complement(523..524)	1,471
12	132571271.. 132571272	HPV16REF	2608..2609	76
16	complement (634428..634429)	HPV16REF	1808..1809	1,227
16	complement (634487..634488)	HPV16REF	3136..3137	245
16	634645..634646	HPV16REF	1806..1807	29,885
20	complement (37184177..37184178)	HPV16REF	226..227	27
21	complement (8259491..8259492)	HPV16REF	2975..2976	927
21	complement (8259602..8259603)	HPV16REF	276..277	290

30927-006.R1 (paired) (Report)

Host chromosome	Host region	Virus	Virus region	Unaligned ends host
21	complement (8260084..8260085)	HPV16REF	2946..2947	200
21	complement (8399441..8399442)	HPV16REF	1609..1610	464
21	complement (8401586..8401587)	HPV16REF	2544..2545	214
21	complement (8401597..8401598)	HPV16REF	5293..5294	365
21	complement (8401610..8401611)	HPV16REF	4701..4702	3,454
21	complement (8401623..8401624)	HPV16REF	2241..2242	336
21	complement (8401627..8401628)	HPV16REF	3259..3260	539
21	complement (8401629..8401630)	HPV16REF	1486..1487	237
21	complement (8401640..8401641)	HPV16REF	2746..2747	600
21	complement (8401648..8401649)	HPV16REF	3533..3534	1,782
21	complement (8401649..8401650)	HPV16REF	3781..3782	366
21	complement (8401665..8401666)	HPV16REF	1502..1503	296
21	complement (8401674..8401675)	HPV16REF	2374..2375	601
21	complement (8401675..8401676)	HPV16REF	complement(523..524)	640
21	complement (8401680..8401681)	HPV16REF	5226..5227	335
21	complement (8401681..8401682)	HPV16REF	607..608	201
21	complement (8401693..8401694)	HPV16REF	3618..3619	441
21	complement (8401697..8401698)	HPV16REF	6548..6549	1,133
21	complement (8401702..8401703)	HPV16REF	3262..3263	308
21	complement (8401703..8401704)	HPV16REF	1042..1043	843
21	complement (8215280..8215281)	HPV16REF	1881..1882	233
21	complement (8215409..8215410)	HPV16REF	2089..2090	736
21	complement (8215642..8215643)	HPV16REF	3785..3786	246

30927-006.R1 (paired) (Report)

Host chromosome	Host region	Virus	Virus region	Unaligned ends host
21	complement (8216032..8216033)	HPV16REF	3676..3677	252
21	complement (8216033..8216034)	HPV16REF	1451..1452	535
21	complement (8401666..8401667)	HPV16REF	3031..3032	346
21	complement (8441234..8441235)	HPV16REF	7456..7457	213
21	complement (8442212..8442213)	HPV16REF	4409..4410	400
21	complement (8442214..8442215)	HPV16REF	2758..2759	228
21	complement (8442223..8442224)	HPV16REF	3848..3849	524
21	complement (8442224..8442225)	HPV16REF	620..621	270
21	complement (8442238..8442239)	HPV16REF	622..623	181
21	complement (8442240..8442241)	HPV16REF	3127..3128	629
21	complement (8442241..8442242)	HPV16REF	1262..1263	225
21	complement (8442257..8442258)	HPV16REF	2611..2612	192
21	complement (8442260..8442261)	HPV16REF	553..554	203
21	complement (8442300..8442301)	HPV16REF	3250..3251	243
21	complement (8442311..8442312)	HPV16REF	1628..1629	289
21	complement (8442312..8442313)	HPV16REF	801..802	220
21	complement (8442313..8442314)	HPV16REF	6686..6687	195
21	complement (8442357..8442358)	HPV16REF	6551..6552	305
21	complement (8442509..8442510)	HPV16REF	553..554	227
21	complement (8442905..8442906)	HPV16REF	7483..7484	267
21	complement (8443174..8443175)	HPV16REF	7724..7725	224
21	complement (8443202..8443203)	HPV16REF	2229..2230	229
21	complement (8443245..8443246)	HPV16REF	5178..5179	447

30927-006.R1 (paired) (Report)

Host chromosome	Host region	Virus	Virus region	Unaligned ends host
21	complement (8443252..8443253)	HPV16REF	7418..7419	242
21	complement (8443175..8443176)	HPV16REF	2080..2081	211
21	8259695..8259696	HPV16REF	complement(406..407)	502
21	8401797..8401798	HPV16REF	complement(523..524)	708
21	8401800..8401801	HPV16REF	232..233	484
21	8401851..8401852	HPV16REF	5469..5470	674
21	8401854..8401855	HPV16REF	complement(406..407)	747
21	8401858..8401859	HPV16REF	4803..4804	503
21	8401872..8401873	HPV16REF	2799..2800	553
21	8215850..8215851	HPV16REF	1715..1716	472
21	8442379..8442380	HPV16REF	3279..3280	749
21	8442421..8442422	HPV16REF	276..277	570
21	8442424..8442425	HPV16REF	3710..3711	490
21	8442455..8442456	HPV16REF	7362..7363	713
21	8442457..8442458	HPV16REF	complement(56..57)	956
21	8442458..8442459	HPV16REF	3159..3160	933
21	8442459..8442460	HPV16REF	4668..4669	1,003
21	8442460..8442461	HPV16REF	complement(406..407)	1,386
21	8442729..8442730	HPV16REF	3306..3307	500
22	21695775..21695776	HPV16REF	6170..6171	24

Host chromosome	Unaligned ends virus	Broken reads host	Disrupted genes	Nearby genes
12	N/A	2	FBRSL1	FBRSL1, gene: ENSG00000280287 (-16119), gene: ENSG00000279700 (-4641), MIR6763 (+10930), gene: ENSG00000277186 (+21964), LRCOL1 (+32083), P2RX2 (+47709), gene: ENSG00000280311 (+50932), POLE (+52686)

30927-006.R1 (paired) (Report)

Host chromosome	Unaligned ends virus	Broken reads host	Disrupted genes	Nearby genes
12	498	2	FBRSL1	FBRSL1, gene: ENSG00000280287 (-16284), gene: ENSG00000279700 (-4806), MIR6763 (+10765), gene: ENSG00000277186 (+21799), LRCOL1 (+31918), P2RX2 (+47544), gene: ENSG00000280311 (+50767), POLE (+52521)
12	N/A	2	FBRSL1	FBRSL1, gene: ENSG00000280287 (-16324), gene: ENSG00000279700 (-4846), MIR6763 (+10725), gene: ENSG00000277186 (+21759), LRCOL1 (+31878), P2RX2 (+47504), gene: ENSG00000280311 (+50727), POLE (+52481)

30927-006.R1 (paired) (Report)

Host chromosome	Unaligned ends virus	Broken reads host	Disrupted genes	Nearby genes
16	N/A	1	METTL26	CAPN15 (-79792), MIR5587 (-99060), MIR3176 (-91062), gene: ENSG00000261691 (-80581), PRR35 (-68899), PIGQ (-50319), NHLRC4 (-64933), gene: ENSG00000282907 (-58171), RAB40C (-5156), WFIKKN1 (-311), METTL26, MCRIP2 (+2388), gene: ENSG00000228201 (+14044), WDR90 (+14882), gene: ENSG00000262528 (+20182), RHOT2 (+33676), RHBDL1 (+41242), LINC02867 (+44075), gene: ENSG00000279441 (+44216), STUB1 (+45795), JMJD8 (+47241), WDR24 (+50193), gene: ENSG00000261659 (+54572), FBXL16 (+58069), gene: ENSG00000279255 (+73221), gene: ENSG00000259840 (+76317), METRN (+80689), ANTKMT (+86152), CCDC78 (+88153), HAGHL (+92507), CIAO3 (+95331)

30927-006.R1 (paired) (Report)

Host chromosome	Unaligned ends virus	Broken reads host	Disrupted genes	Nearby genes
16	N/A	1	METTL26	CAPN15 (-79851), MIR5587 (-99119), MIR3176 (-91121), gene: ENSG00000261691 (-80640), PRR35 (-68958), PIGQ (-50378), NHLRC4 (-64992), gene: ENSG00000282907 (-58230), RAB40C (-5215), WFIKKN1 (-370), METTL26, MCRIP2 (+2329), gene: ENSG00000228201 (+13985), WDR90 (+14823), gene: ENSG00000262528 (+20123), RHOT2 (+33617), RHBDL1 (+41183), LINC02867 (+44016), gene: ENSG00000279441 (+44157), STUB1 (+45736), JMJD8 (+47182), WDR24 (+50134), gene: ENSG00000261659 (+54513), FBXL16 (+58010), gene: ENSG00000279255 (+73162), gene: ENSG00000259840 (+76258), METRN (+80630), ANTKMT (+86093), CCDC78 (+88094), HAGHL (+92448), CIAO3 (+95272)

30927-006.R1 (paired) (Report)

Host chromosome	Unaligned ends virus	Broken reads host	Disrupted genes	Nearby genes
16	N/A	1	METTL26	CAPN15 (-80009), MIR5587 (-99277), MIR3176 (-91279), gene: ENSG00000261691 (-80798), PRR35 (-69116), PIGQ (-50536), NHLRC4 (-65150), gene: ENSG00000282907 (-58388), RAB40C (-5373), WFIKKN1 (-528), METTL26, MCRIP2 (+2171), gene: ENSG00000228201 (+13827), WDR90 (+14665), gene: ENSG00000262528 (+19965), RHOT2 (+33459), RHBDL1 (+41025), LINC02867 (+43858), gene: ENSG00000279441 (+43999), STUB1 (+45578), JMJD8 (+47024), WDR24 (+49976), gene: ENSG00000261659 (+54355), FBXL16 (+57852), gene: ENSG00000279255 (+73004), gene: ENSG00000259840 (+76100), METRN (+80472), ANTKMT (+85935), CCDC78 (+87936), HAGHL (+92290), CIAO3 (+95114)
20	202	2	RPN2	RBL1 (-88180), gene: ENSG00000269846 (-86999), MROH8 (-4589), RPN2, GHRH (+66908)

30927-006.R1 (paired) (Report)

Host chromosome	Unaligned ends virus	Broken reads host	Disrupted genes	Nearby genes
21	N/A	2		gene: ENSG00000278996 (-31845), MIR6724-1 (-54085), gene: ENSG00000275664 (-53551), MIR3648-1 (-50839), gene: ENSG00000277437 (-50587), gene: ENSG00000280800 (-48185), RNA5-8SN2 (-46767), gene: ENSG00000286155 (-23659), gene: ENSG00000286252 (-21512), gene: ENSG00000286057 (-20399), gene: ENSG00000286012 (-18444), MIR6724-2 (-9895), gene: ENSG00000277671 (-9342), gene: ENSG00000281383 (-3977), 5_8S_rRNA (-2558)
21	27	2		gene: ENSG00000278996 (-31956), MIR6724-1 (-54196), gene: ENSG00000275664 (-53662), MIR3648-1 (-50950), gene: ENSG00000277437 (-50698), gene: ENSG00000280800 (-48296), RNA5-8SN2 (-46878), gene: ENSG00000286155 (-23770), gene: ENSG00000286252 (-21623), gene: ENSG00000286057 (-20510), gene: ENSG00000286012 (-18555), MIR6724-2 (-10006), gene: ENSG00000277671 (-9453), gene: ENSG00000281383 (-4088), 5_8S_rRNA (-2669)

30927-006.R1 (paired) (Report)

Host chromosome	Unaligned ends virus	Broken reads host	Disrupted genes	Nearby genes
21	N/A	2		gene: ENSG00000278996 (-32438), MIR6724-1 (-54678), gene: ENSG00000275664 (-54144), MIR3648-1 (-51432), gene: ENSG00000277437 (-51180), gene: ENSG00000280800 (-48778), RNA5-8SN2 (-47360), gene: ENSG00000286155 (-24252), gene: ENSG00000286252 (-22105), gene: ENSG00000286057 (-20992), gene: ENSG00000286012 (-19037), MIR6724-2 (-10488), gene: ENSG00000277671 (-9935), gene: ENSG00000281383 (-4570), 5_8S_rRNA (-3151)

30927-006.R1 (paired) (Report)

Host chromosome	Unaligned ends virus	Broken reads host	Disrupted genes	Nearby genes
21	N/A	1	gene: ENSG00000280441	gene: ENSG00000280441, MIR6724-3 (-10988), gene: ENSG00000274868 (-10454), gene: ENSG00000280614 (-5100), RNA5-8SN3 (-3682), gene: ENSG00000286267 (+18315), gene: ENSG00000286146 (+21233), gene: ENSG00000286054 (+22441), gene: ENSG00000286178 (+24318), MIR6724-4 (+33088), gene: ENSG00000278775 (+33643), gene: ENSG00000281181 (+38187), RNA5-8SN1 (+40381), gene: ENSG00000286149 (+62878), gene: ENSG00000286032 (+65812), gene: ENSG00000286091 (+67024), gene: ENSG00000286148 (+68878)

30927-006.R1 (paired) (Report)

Host chromosome	Unaligned ends virus	Broken reads host	Disrupted genes	Nearby genes
21	N/A	6	gene: ENSG00000280441	gene: ENSG00000280441, MIR6724-3 (-13133), gene: ENSG00000274868 (-12599), gene: ENSG00000280614 (-7245), RNA5-8SN3 (-5827), gene: ENSG00000286267 (+16170), gene: ENSG00000286146 (+19088), gene: ENSG00000286054 (+20296), gene: ENSG00000286178 (+22173), MIR6724-4 (+30943), gene: ENSG00000278775 (+31498), gene: ENSG00000281181 (+36042), RNA5-8SN1 (+38236), gene: ENSG00000286149 (+60733), gene: ENSG00000286032 (+63667), gene: ENSG00000286091 (+64879), gene: ENSG00000286148 (+66733)

30927-006.R1 (paired) (Report)

Host chromosome	Unaligned ends virus	Broken reads host	Disrupted genes	Nearby genes
21	N/A	6	gene: ENSG00000280441	gene: ENSG00000280441, MIR6724-3 (-13144), gene: ENSG00000274868 (-12610), gene: ENSG00000280614 (-7256), RNA5-8SN3 (-5838), gene: ENSG00000286267 (+16159), gene: ENSG00000286146 (+19077), gene: ENSG00000286054 (+20285), gene: ENSG00000286178 (+22162), MIR6724-4 (+30932), gene: ENSG00000278775 (+31487), gene: ENSG00000281181 (+36031), RNA5-8SN1 (+38225), gene: ENSG00000286149 (+60722), gene: ENSG00000286032 (+63656), gene: ENSG00000286091 (+64868), gene: ENSG00000286148 (+66722)

30927-006.R1 (paired) (Report)

Host chromosome	Unaligned ends virus	Broken reads host	Disrupted genes	Nearby genes
21	N/A	6	gene: ENSG00000280441	gene: ENSG00000280441, MIR6724-3 (-13157), gene: ENSG00000274868 (-12623), gene: ENSG00000280614 (-7269), RNA5-8SN3 (-5851), gene: ENSG00000286267 (+16146), gene: ENSG00000286146 (+19064), gene: ENSG00000286054 (+20272), gene: ENSG00000286178 (+22149), MIR6724-4 (+30919), gene: ENSG00000278775 (+31474), gene: ENSG00000281181 (+36018), RNA5-8SN1 (+38212), gene: ENSG00000286149 (+60709), gene: ENSG00000286032 (+63643), gene: ENSG00000286091 (+64855), gene: ENSG00000286148 (+66709)

30927-006.R1 (paired) (Report)

Host chromosome	Unaligned ends virus	Broken reads host	Disrupted genes	Nearby genes
21	N/A	6	gene: ENSG00000280441	gene: ENSG00000280441, MIR6724-3 (-13170), gene: ENSG00000274868 (-12636), gene: ENSG00000280614 (-7282), RNA5-8SN3 (-5864), gene: ENSG00000286267 (+16133), gene: ENSG00000286146 (+19051), gene: ENSG00000286054 (+20259), gene: ENSG00000286178 (+22136), MIR6724-4 (+30906), gene: ENSG00000278775 (+31461), gene: ENSG00000281181 (+36005), RNA5-8SN1 (+38199), gene: ENSG00000286149 (+60696), gene: ENSG00000286032 (+63630), gene: ENSG00000286091 (+64842), gene: ENSG00000286148 (+66696)

30927-006.R1 (paired) (Report)

Host chromosome	Unaligned ends virus	Broken reads host	Disrupted genes	Nearby genes
21	N/A	6	gene: ENSG00000280441	gene: ENSG00000280441, MIR6724-3 (-13174), gene: ENSG00000274868 (-12640), gene: ENSG00000280614 (-7286), RNA5-8SN3 (-5868), gene: ENSG00000286267 (+16129), gene: ENSG00000286146 (+19047), gene: ENSG00000286054 (+20255), gene: ENSG00000286178 (+22132), MIR6724-4 (+30902), gene: ENSG00000278775 (+31457), gene: ENSG00000281181 (+36001), RNA5-8SN1 (+38195), gene: ENSG00000286149 (+60692), gene: ENSG00000286032 (+63626), gene: ENSG00000286091 (+64838), gene: ENSG00000286148 (+66692)

30927-006.R1 (paired) (Report)

Host chromosome	Unaligned ends virus	Broken reads host	Disrupted genes	Nearby genes
21	N/A	6	gene: ENSG00000280441	gene: ENSG00000280441, MIR6724-3 (-13176), gene: ENSG00000274868 (-12642), gene: ENSG00000280614 (-7288), RNA5-8SN3 (-5870), gene: ENSG00000286267 (+16127), gene: ENSG00000286146 (+19045), gene: ENSG00000286054 (+20253), gene: ENSG00000286178 (+22130), MIR6724-4 (+30900), gene: ENSG00000278775 (+31455), gene: ENSG00000281181 (+35999), RNA5-8SN1 (+38193), gene: ENSG00000286149 (+60690), gene: ENSG00000286032 (+63624), gene: ENSG00000286091 (+64836), gene: ENSG00000286148 (+66690)

30927-006.R1 (paired) (Report)

Host chromosome	Unaligned ends virus	Broken reads host	Disrupted genes	Nearby genes
21	N/A	6	gene: ENSG00000280441	gene: ENSG00000280441, MIR6724-3 (-13187), gene: ENSG00000274868 (-12653), gene: ENSG00000280614 (-7299), RNA5-8SN3 (-5881), gene: ENSG00000286267 (+16116), gene: ENSG00000286146 (+19034), gene: ENSG00000286054 (+20242), gene: ENSG00000286178 (+22119), MIR6724-4 (+30889), gene: ENSG00000278775 (+31444), gene: ENSG00000281181 (+35988), RNA5-8SN1 (+38182), gene: ENSG00000286149 (+60679), gene: ENSG00000286032 (+63613), gene: ENSG00000286091 (+64825), gene: ENSG00000286148 (+66679)

30927-006.R1 (paired) (Report)

Host chromosome	Unaligned ends virus	Broken reads host	Disrupted genes	Nearby genes
21	N/A	6	gene: ENSG00000280441	gene: ENSG00000280441, MIR6724-3 (-13195), gene: ENSG00000274868 (-12661), gene: ENSG00000280614 (-7307), RNA5-8SN3 (-5889), gene: ENSG00000286267 (+16108), gene: ENSG00000286146 (+19026), gene: ENSG00000286054 (+20234), gene: ENSG00000286178 (+22111), MIR6724-4 (+30881), gene: ENSG00000278775 (+31436), gene: ENSG00000281181 (+35980), RNA5-8SN1 (+38174), gene: ENSG00000286149 (+60671), gene: ENSG00000286032 (+63605), gene: ENSG00000286091 (+64817), gene: ENSG00000286148 (+66671)

30927-006.R1 (paired) (Report)

Host chromosome	Unaligned ends virus	Broken reads host	Disrupted genes	Nearby genes
21	N/A	6	gene: ENSG00000280441	gene: ENSG00000280441, MIR6724-3 (-13196), gene: ENSG00000274868 (-12662), gene: ENSG00000280614 (-7308), RNA5-8SN3 (-5890), gene: ENSG00000286267 (+16107), gene: ENSG00000286146 (+19025), gene: ENSG00000286054 (+20233), gene: ENSG00000286178 (+22110), MIR6724-4 (+30880), gene: ENSG00000278775 (+31435), gene: ENSG00000281181 (+35979), RNA5-8SN1 (+38173), gene: ENSG00000286149 (+60670), gene: ENSG00000286032 (+63604), gene: ENSG00000286091 (+64816), gene: ENSG00000286148 (+66670)

30927-006.R1 (paired) (Report)

Host chromosome	Unaligned ends virus	Broken reads host	Disrupted genes	Nearby genes
21	N/A	6	gene: ENSG00000280441	gene: ENSG00000280441, MIR6724-3 (-13212), gene: ENSG00000274868 (-12678), gene: ENSG00000280614 (-7324), RNA5-8SN3 (-5906), gene: ENSG00000286267 (+16091), gene: ENSG00000286146 (+19009), gene: ENSG00000286054 (+20217), gene: ENSG00000286178 (+22094), MIR6724-4 (+30864), gene: ENSG00000278775 (+31419), gene: ENSG00000281181 (+35963), RNA5-8SN1 (+38157), gene: ENSG00000286149 (+60654), gene: ENSG00000286032 (+63588), gene: ENSG00000286091 (+64800), gene: ENSG00000286148 (+66654)

30927-006.R1 (paired) (Report)

Host chromosome	Unaligned ends virus	Broken reads host	Disrupted genes	Nearby genes
21	N/A	6	gene: ENSG00000280441	gene: ENSG00000280441, MIR6724-3 (-13221), gene: ENSG00000274868 (-12687), gene: ENSG00000280614 (-7333), RNA5-8SN3 (-5915), gene: ENSG00000286267 (+16082), gene: ENSG00000286146 (+19000), gene: ENSG00000286054 (+20208), gene: ENSG00000286178 (+22085), MIR6724-4 (+30855), gene: ENSG00000278775 (+31410), gene: ENSG00000281181 (+35954), RNA5-8SN1 (+38148), gene: ENSG00000286149 (+60645), gene: ENSG00000286032 (+63579), gene: ENSG00000286091 (+64791), gene: ENSG00000286148 (+66645)

30927-006.R1 (paired) (Report)

Host chromosome	Unaligned ends virus	Broken reads host	Disrupted genes	Nearby genes
21	498	6	gene: ENSG00000280441	gene: ENSG00000280441, MIR6724-3 (-13222), gene: ENSG00000274868 (-12688), gene: ENSG00000280614 (-7334), RNA5-8SN3 (-5916), gene: ENSG00000286267 (+16081), gene: ENSG00000286146 (+18999), gene: ENSG00000286054 (+20207), gene: ENSG00000286178 (+22084), MIR6724-4 (+30854), gene: ENSG00000278775 (+31409), gene: ENSG00000281181 (+35953), RNA5-8SN1 (+38147), gene: ENSG00000286149 (+60644), gene: ENSG00000286032 (+63578), gene: ENSG00000286091 (+64790), gene: ENSG00000286148 (+66644)

30927-006.R1 (paired) (Report)

Host chromosome	Unaligned ends virus	Broken reads host	Disrupted genes	Nearby genes
21	N/A	6	gene: ENSG00000280441	gene: ENSG00000280441, MIR6724-3 (-13227), gene: ENSG00000274868 (-12693), gene: ENSG00000280614 (-7339), RNA5-8SN3 (-5921), gene: ENSG00000286267 (+16076), gene: ENSG00000286146 (+18994), gene: ENSG00000286054 (+20202), gene: ENSG00000286178 (+22079), MIR6724-4 (+30849), gene: ENSG00000278775 (+31404), gene: ENSG00000281181 (+35948), RNA5-8SN1 (+38142), gene: ENSG00000286149 (+60639), gene: ENSG00000286032 (+63573), gene: ENSG00000286091 (+64785), gene: ENSG00000286148 (+66639)

30927-006.R1 (paired) (Report)

Host chromosome	Unaligned ends virus	Broken reads host	Disrupted genes	Nearby genes
21	N/A	1	gene: ENSG00000280441	gene: ENSG00000280441, MIR6724-3 (-13228), gene: ENSG00000274868 (-12694), gene: ENSG00000280614 (-7340), RNA5-8SN3 (-5922), gene: ENSG00000286267 (+16075), gene: ENSG00000286146 (+18993), gene: ENSG00000286054 (+20201), gene: ENSG00000286178 (+22078), MIR6724-4 (+30848), gene: ENSG00000278775 (+31403), gene: ENSG00000281181 (+35947), RNA5-8SN1 (+38141), gene: ENSG00000286149 (+60638), gene: ENSG00000286032 (+63572), gene: ENSG00000286091 (+64784), gene: ENSG00000286148 (+66638)

30927-006.R1 (paired) (Report)

Host chromosome	Unaligned ends virus	Broken reads host	Disrupted genes	Nearby genes
21	N/A	1	gene: ENSG00000280441	gene: ENSG00000280441, MIR6724-3 (-13240), gene: ENSG00000274868 (-12706), gene: ENSG00000280614 (-7352), RNA5-8SN3 (-5934), gene: ENSG00000286267 (+16063), gene: ENSG00000286146 (+18981), gene: ENSG00000286054 (+20189), gene: ENSG00000286178 (+22066), MIR6724-4 (+30836), gene: ENSG00000278775 (+31391), gene: ENSG00000281181 (+35935), RNA5-8SN1 (+38129), gene: ENSG00000286149 (+60626), gene: ENSG00000286032 (+63560), gene: ENSG00000286091 (+64772), gene: ENSG00000286148 (+66626)

30927-006.R1 (paired) (Report)

Host chromosome	Unaligned ends virus	Broken reads host	Disrupted genes	Nearby genes
21	N/A	1	gene: ENSG00000280441	gene: ENSG00000280441, MIR6724-3 (-13244), gene: ENSG00000274868 (-12710), gene: ENSG00000280614 (-7356), RNA5-8SN3 (-5938), gene: ENSG00000286267 (+16059), gene: ENSG00000286146 (+18977), gene: ENSG00000286054 (+20185), gene: ENSG00000286178 (+22062), MIR6724-4 (+30832), gene: ENSG00000278775 (+31387), gene: ENSG00000281181 (+35931), RNA5-8SN1 (+38125), gene: ENSG00000286149 (+60622), gene: ENSG00000286032 (+63556), gene: ENSG00000286091 (+64768), gene: ENSG00000286148 (+66622)

30927-006.R1 (paired) (Report)

Host chromosome	Unaligned ends virus	Broken reads host	Disrupted genes	Nearby genes
21	N/A	1	gene: ENSG00000280441	gene: ENSG00000280441, MIR6724-3 (-13249), gene: ENSG00000274868 (-12715), gene: ENSG00000280614 (-7361), RNA5-8SN3 (-5943), gene: ENSG00000286267 (+16054), gene: ENSG00000286146 (+18972), gene: ENSG00000286054 (+20180), gene: ENSG00000286178 (+22057), MIR6724-4 (+30827), gene: ENSG00000278775 (+31382), gene: ENSG00000281181 (+35926), RNA5-8SN1 (+38120), gene: ENSG00000286149 (+60617), gene: ENSG00000286032 (+63551), gene: ENSG00000286091 (+64763), gene: ENSG00000286148 (+66617)

30927-006.R1 (paired) (Report)

Host chromosome	Unaligned ends virus	Broken reads host	Disrupted genes	Nearby genes
21	N/A	1	gene: ENSG00000280441	gene: ENSG00000280441, MIR6724-3 (-13250), gene: ENSG00000274868 (-12716), gene: ENSG00000280614 (-7362), RNA5-8SN3 (-5944), gene: ENSG00000286267 (+16053), gene: ENSG00000286146 (+18971), gene: ENSG00000286054 (+20179), gene: ENSG00000286178 (+22056), MIR6724-4 (+30826), gene: ENSG00000278775 (+31381), gene: ENSG00000281181 (+35925), RNA5-8SN1 (+38119), gene: ENSG00000286149 (+60616), gene: ENSG00000286032 (+63550), gene: ENSG00000286091 (+64762), gene: ENSG00000286148 (+66616)

30927-006.R1 (paired) (Report)

Host chromosome	Unaligned ends virus	Broken reads host	Disrupted genes	Nearby genes
21	N/A	1	gene: ENSG00000278996	gene: ENSG00000278996, MIR6724-1 (-9874), gene: ENSG00000275664 (-9340), MIR3648-1 (-6628), gene: ENSG00000277437 (-6376), gene: ENSG00000280800 (-3974), RNA5-8SN2 (-2556), gene: ENSG00000286155 (+20073), gene: ENSG00000286252 (+22368), gene: ENSG00000286057 (+23578), gene: ENSG00000286012 (+25455), MIR6724-2 (+34224), gene: ENSG00000277671 (+34779), gene: ENSG00000281383 (+39311), 5_8S_rRNA (+41500)
21	N/A	1	gene: ENSG00000278996	gene: ENSG00000278996, MIR6724-1 (-10003), gene: ENSG00000275664 (-9469), MIR3648-1 (-6757), gene: ENSG00000277437 (-6505), gene: ENSG00000280800 (-4103), RNA5-8SN2 (-2685), gene: ENSG00000286155 (+19944), gene: ENSG00000286252 (+22239), gene: ENSG00000286057 (+23449), gene: ENSG00000286012 (+25326), MIR6724-2 (+34095), gene: ENSG00000277671 (+34650), gene: ENSG00000281383 (+39182), 5_8S_rRNA (+41371)

Table S4 Viral integration sites reports

30927-006.R1 (paired) (Report)

Host chromosome	Unaligned ends virus	Broken reads host	Disrupted genes	Nearby genes
21	N/A	2	gene: ENSG00000278996	gene: ENSG00000278996, MIR6724-1 (-10236), gene: ENSG00000275664 (-9702), MIR3648-1 (-6990), gene: ENSG00000277437 (-6738), gene: ENSG00000280800 (-4336), RNA5-8SN2 (-2918), gene: ENSG00000286155 (+19711), gene: ENSG00000286252 (+22006), gene: ENSG00000286057 (+23216), gene: ENSG00000286012 (+25093), MIR6724-2 (+33862), gene: ENSG00000277671 (+34417), gene: ENSG00000281383 (+38949), 5_8S_rRNA (+41138)
21	N/A	2	gene: ENSG00000278996	gene: ENSG00000278996, MIR6724-1 (-10626), gene: ENSG00000275664 (-10092), MIR3648-1 (-7380), gene: ENSG00000277437 (-7128), gene: ENSG00000280800 (-4726), RNA5-8SN2 (-3308), gene: ENSG00000286155 (+19321), gene: ENSG00000286252 (+21616), gene: ENSG00000286057 (+22826), gene: ENSG00000286012 (+24703), MIR6724-2 (+33472), gene: ENSG00000277671 (+34027), gene: ENSG00000281383 (+38559), 5_8S_rRNA (+40748)

30927-006.R1 (paired) (Report)

Host chromosome	Unaligned ends virus	Broken reads host	Disrupted genes	Nearby genes
21	N/A	2	gene: ENSG00000278996	gene: ENSG00000278996, MIR6724-1 (-10627), gene: ENSG00000275664 (-10093), MIR3648-1 (-7381), gene: ENSG00000277437 (-7129), gene: ENSG00000280800 (-4727), RNA5-8SN2 (-3309), gene: ENSG00000286155 (+19320), gene: ENSG00000286252 (+21615), gene: ENSG00000286057 (+22825), gene: ENSG00000286012 (+24702), MIR6724-2 (+33471), gene: ENSG00000277671 (+34026), gene: ENSG00000281383 (+38558), 5_8S_rRNA (+40747)

30927-006.R1 (paired) (Report)

Host chromosome	Unaligned ends virus	Broken reads host	Disrupted genes	Nearby genes
21	N/A	6	gene: ENSG00000280441	gene: ENSG00000280441, MIR6724-3 (-13213), gene: ENSG00000274868 (-12679), gene: ENSG00000280614 (-7325), RNA5-8SN3 (-5907), gene: ENSG00000286267 (+16090), gene: ENSG00000286146 (+19008), gene: ENSG00000286054 (+20216), gene: ENSG00000286178 (+22093), MIR6724-4 (+30863), gene: ENSG00000278775 (+31418), gene: ENSG00000281181 (+35962), RNA5-8SN1 (+38156), gene: ENSG00000286149 (+60653), gene: ENSG00000286032 (+63587), gene: ENSG00000286091 (+64799), gene: ENSG00000286148 (+66653)

30927-006.R1 (paired) (Report)

Host chromosome	Unaligned ends virus	Broken reads host	Disrupted genes	Nearby genes
21	N/A	1	gene: ENSG00000280441	gene: ENSG00000280441, MIR6724-3 (-52781), gene: ENSG00000274868 (-52247), gene: ENSG00000280614 (-46893), RNA5-8SN3 (-45475), gene: ENSG00000286267 (-22999), gene: ENSG00000286146 (-20231), gene: ENSG00000286054 (-19118), gene: ENSG00000286178 (-17163), MIR6724-4 (-8613), gene: ENSG00000278775 (-8060), gene: ENSG00000281181 (-2683), RNA5-8SN1 (-1259), gene: ENSG00000286149 (+21085), gene: ENSG00000286032 (+24019), gene: ENSG00000286091 (+25231), gene: ENSG00000286148 (+27085)

30927-006.R1 (paired) (Report)

Host chromosome	Unaligned ends virus	Broken reads host	Disrupted genes	Nearby genes
21	N/A	5	gene: ENSG00000280441	gene: ENSG00000280441, MIR6724-3 (-53759), gene: ENSG00000274868 (-53225), gene: ENSG00000280614 (-47871), RNA5-8SN3 (-46453), gene: ENSG00000286267 (-23977), gene: ENSG00000286146 (-21209), gene: ENSG00000286054 (-20096), gene: ENSG00000286178 (-18141), MIR6724-4 (-9591), gene: ENSG00000278775 (-9038), gene: ENSG00000281181 (-3661), RNA5-8SN1 (-2237), gene: ENSG00000286149 (+20107), gene: ENSG00000286032 (+23041), gene: ENSG00000286091 (+24253), gene: ENSG00000286148 (+26107)

30927-006.R1 (paired) (Report)

Host chromosome	Unaligned ends virus	Broken reads host	Disrupted genes	Nearby genes
21	N/A	5	gene: ENSG00000280441	gene: ENSG00000280441, MIR6724-3 (-53761), gene: ENSG00000274868 (-53227), gene: ENSG00000280614 (-47873), RNA5-8SN3 (-46455), gene: ENSG00000286267 (-23979), gene: ENSG00000286146 (-21211), gene: ENSG00000286054 (-20098), gene: ENSG00000286178 (-18143), MIR6724-4 (-9593), gene: ENSG00000278775 (-9040), gene: ENSG00000281181 (-3663), RNA5-8SN1 (-2239), gene: ENSG00000286149 (+20105), gene: ENSG00000286032 (+23039), gene: ENSG00000286091 (+24251), gene: ENSG00000286148 (+26105)

30927-006.R1 (paired) (Report)

Host chromosome	Unaligned ends virus	Broken reads host	Disrupted genes	Nearby genes
21	N/A	5	gene: ENSG00000280441	gene: ENSG00000280441, MIR6724-3 (-53770), gene: ENSG00000274868 (-53236), gene: ENSG00000280614 (-47882), RNA5-8SN3 (-46464), gene: ENSG00000286267 (-23988), gene: ENSG00000286146 (-21220), gene: ENSG00000286054 (-20107), gene: ENSG00000286178 (-18152), MIR6724-4 (-9602), gene: ENSG00000278775 (-9049), gene: ENSG00000281181 (-3672), RNA5-8SN1 (-2248), gene: ENSG00000286149 (+20096), gene: ENSG00000286032 (+23030), gene: ENSG00000286091 (+24242), gene: ENSG00000286148 (+26096)

30927-006.R1 (paired) (Report)

Host chromosome	Unaligned ends virus	Broken reads host	Disrupted genes	Nearby genes
21	N/A	5	gene: ENSG00000280441	gene: ENSG00000280441, MIR6724-3 (-53771), gene: ENSG00000274868 (-53237), gene: ENSG00000280614 (-47883), RNA5-8SN3 (-46465), gene: ENSG00000286267 (-23989), gene: ENSG00000286146 (-21221), gene: ENSG00000286054 (-20108), gene: ENSG00000286178 (-18153), MIR6724-4 (-9603), gene: ENSG00000278775 (-9050), gene: ENSG00000281181 (-3673), RNA5-8SN1 (-2249), gene: ENSG00000286149 (+20095), gene: ENSG00000286032 (+23029), gene: ENSG00000286091 (+24241), gene: ENSG00000286148 (+26095)

30927-006.R1 (paired) (Report)

Host chromosome	Unaligned ends virus	Broken reads host	Disrupted genes	Nearby genes
21	N/A	4	gene: ENSG00000280441	gene: ENSG00000280441, MIR6724-3 (-53785), gene: ENSG00000274868 (-53251), gene: ENSG00000280614 (-47897), RNA5-8SN3 (-46479), gene: ENSG00000286267 (-24003), gene: ENSG00000286146 (-21235), gene: ENSG00000286054 (-20122), gene: ENSG00000286178 (-18167), MIR6724-4 (-9617), gene: ENSG00000278775 (-9064), gene: ENSG00000281181 (-3687), RNA5-8SN1 (-2263), gene: ENSG00000286149 (+20081), gene: ENSG00000286032 (+23015), gene: ENSG00000286091 (+24227), gene: ENSG00000286148 (+26081)

30927-006.R1 (paired) (Report)

Host chromosome	Unaligned ends virus	Broken reads host	Disrupted genes	Nearby genes
21	N/A	4	gene: ENSG00000280441	gene: ENSG00000280441, MIR6724-3 (-53787), gene: ENSG00000274868 (-53253), gene: ENSG00000280614 (-47899), RNA5-8SN3 (-46481), gene: ENSG00000286267 (-24005), gene: ENSG00000286146 (-21237), gene: ENSG00000286054 (-20124), gene: ENSG00000286178 (-18169), MIR6724-4 (-9619), gene: ENSG00000278775 (-9066), gene: ENSG00000281181 (-3689), RNA5-8SN1 (-2265), gene: ENSG00000286149 (+20079), gene: ENSG00000286032 (+23013), gene: ENSG00000286091 (+24225), gene: ENSG00000286148 (+26079)

30927-006.R1 (paired) (Report)

Host chromosome	Unaligned ends virus	Broken reads host	Disrupted genes	Nearby genes
21	N/A	4	gene: ENSG00000280441	gene: ENSG00000280441, MIR6724-3 (-53788), gene: ENSG00000274868 (-53254), gene: ENSG00000280614 (-47900), RNA5-8SN3 (-46482), gene: ENSG00000286267 (-24006), gene: ENSG00000286146 (-21238), gene: ENSG00000286054 (-20125), gene: ENSG00000286178 (-18170), MIR6724-4 (-9620), gene: ENSG00000278775 (-9067), gene: ENSG00000281181 (-3690), RNA5-8SN1 (-2266), gene: ENSG00000286149 (+20078), gene: ENSG00000286032 (+23012), gene: ENSG00000286091 (+24224), gene: ENSG00000286148 (+26078)

30927-006.R1 (paired) (Report)

Host chromosome	Unaligned ends virus	Broken reads host	Disrupted genes	Nearby genes
21	N/A	4	gene: ENSG00000280441	gene: ENSG00000280441, MIR6724-3 (-53804), gene: ENSG00000274868 (-53270), gene: ENSG00000280614 (-47916), RNA5-8SN3 (-46498), gene: ENSG00000286267 (-24022), gene: ENSG00000286146 (-21254), gene: ENSG00000286054 (-20141), gene: ENSG00000286178 (-18186), MIR6724-4 (-9636), gene: ENSG00000278775 (-9083), gene: ENSG00000281181 (-3706), RNA5-8SN1 (-2282), gene: ENSG00000286149 (+20062), gene: ENSG00000286032 (+22996), gene: ENSG00000286091 (+24208), gene: ENSG00000286148 (+26062)

30927-006.R1 (paired) (Report)

Host chromosome	Unaligned ends virus	Broken reads host	Disrupted genes	Nearby genes
21	316	4	gene: ENSG00000280441	gene: ENSG00000280441, MIR6724-3 (-53807), gene: ENSG00000274868 (-53273), gene: ENSG00000280614 (-47919), RNA5-8SN3 (-46501), gene: ENSG00000286267 (-24025), gene: ENSG00000286146 (-21257), gene: ENSG00000286054 (-20144), gene: ENSG00000286178 (-18189), MIR6724-4 (-9639), gene: ENSG00000278775 (-9086), gene: ENSG00000281181 (-3709), RNA5-8SN1 (-2285), gene: ENSG00000286149 (+20059), gene: ENSG00000286032 (+22993), gene: ENSG00000286091 (+24205), gene: ENSG00000286148 (+26059)

30927-006.R1 (paired) (Report)

Host chromosome	Unaligned ends virus	Broken reads host	Disrupted genes	Nearby genes
21	N/A	3	gene: ENSG00000280441	gene: ENSG00000280441, MIR6724-3 (-53847), gene: ENSG00000274868 (-53313), gene: ENSG00000280614 (-47959), RNA5-8SN3 (-46541), gene: ENSG00000286267 (-24065), gene: ENSG00000286146 (-21297), gene: ENSG00000286054 (-20184), gene: ENSG00000286178 (-18229), MIR6724-4 (-9679), gene: ENSG00000278775 (-9126), gene: ENSG00000281181 (-3749), RNA5-8SN1 (-2325), gene: ENSG00000286149 (+20019), gene: ENSG00000286032 (+22953), gene: ENSG00000286091 (+24165), gene: ENSG00000286148 (+26019)

30927-006.R1 (paired) (Report)

Host chromosome	Unaligned ends virus	Broken reads host	Disrupted genes	Nearby genes
21	N/A	3	gene: ENSG00000280441	gene: ENSG00000280441, MIR6724-3 (-53858), gene: ENSG00000274868 (-53324), gene: ENSG00000280614 (-47970), RNA5-8SN3 (-46552), gene: ENSG00000286267 (-24076), gene: ENSG00000286146 (-21308), gene: ENSG00000286054 (-20195), gene: ENSG00000286178 (-18240), MIR6724-4 (-9690), gene: ENSG00000278775 (-9137), gene: ENSG00000281181 (-3760), RNA5-8SN1 (-2336), gene: ENSG00000286149 (+20008), gene: ENSG00000286032 (+22942), gene: ENSG00000286091 (+24154), gene: ENSG00000286148 (+26008)

30927-006.R1 (paired) (Report)

Host chromosome	Unaligned ends virus	Broken reads host	Disrupted genes	Nearby genes
21	220	3	gene: ENSG00000280441	gene: ENSG00000280441, MIR6724-3 (-53859), gene: ENSG00000274868 (-53325), gene: ENSG00000280614 (-47971), RNA5-8SN3 (-46553), gene: ENSG00000286267 (-24077), gene: ENSG00000286146 (-21309), gene: ENSG00000286054 (-20196), gene: ENSG00000286178 (-18241), MIR6724-4 (-9691), gene: ENSG00000278775 (-9138), gene: ENSG00000281181 (-3761), RNA5-8SN1 (-2337), gene: ENSG00000286149 (+20007), gene: ENSG00000286032 (+22941), gene: ENSG00000286091 (+24153), gene: ENSG00000286148 (+26007)

30927-006.R1 (paired) (Report)

Host chromosome	Unaligned ends virus	Broken reads host	Disrupted genes	Nearby genes
21	N/A	3	gene: ENSG00000280441	gene: ENSG00000280441, MIR6724-3 (-53860), gene: ENSG00000274868 (-53326), gene: ENSG00000280614 (-47972), RNA5-8SN3 (-46554), gene: ENSG00000286267 (-24078), gene: ENSG00000286146 (-21310), gene: ENSG00000286054 (-20197), gene: ENSG00000286178 (-18242), MIR6724-4 (-9692), gene: ENSG00000278775 (-9139), gene: ENSG00000281181 (-3762), RNA5-8SN1 (-2338), gene: ENSG00000286149 (+20006), gene: ENSG00000286032 (+22940), gene: ENSG00000286091 (+24152), gene: ENSG00000286148 (+26006)

30927-006.R1 (paired) (Report)

Host chromosome	Unaligned ends virus	Broken reads host	Disrupted genes	Nearby genes
21	N/A	3	gene: ENSG00000280441	gene: ENSG00000280441, MIR6724-3 (-53904), gene: ENSG00000274868 (-53370), gene: ENSG00000280614 (-48016), RNA5-8SN3 (-46598), gene: ENSG00000286267 (-24122), gene: ENSG00000286146 (-21354), gene: ENSG00000286054 (-20241), gene: ENSG00000286178 (-18286), MIR6724-4 (-9736), gene: ENSG00000278775 (-9183), gene: ENSG00000281181 (-3806), RNA5-8SN1 (-2382), gene: ENSG00000286149 (+19962), gene: ENSG00000286032 (+22896), gene: ENSG00000286091 (+24108), gene: ENSG00000286148 (+25962)

30927-006.R1 (paired) (Report)

Host chromosome	Unaligned ends virus	Broken reads host	Disrupted genes	Nearby genes
21	316	1	gene: ENSG00000280441	gene: ENSG00000280441, MIR6724-3 (-54056), gene: ENSG00000274868 (-53522), gene: ENSG00000280614 (-48168), RNA5-8SN3 (-46750), gene: ENSG00000286267 (-24274), gene: ENSG00000286146 (-21506), gene: ENSG00000286054 (-20393), gene: ENSG00000286178 (-18438), MIR6724-4 (-9888), gene: ENSG00000278775 (-9335), gene: ENSG00000281181 (-3958), RNA5-8SN1 (-2534), gene: ENSG00000286149 (+19810), gene: ENSG00000286032 (+22744), gene: ENSG00000286091 (+23956), gene: ENSG00000286148 (+25810)

30927-006.R1 (paired) (Report)

Host chromosome	Unaligned ends virus	Broken reads host	Disrupted genes	Nearby genes
21	N/A	3	gene: ENSG00000280441	gene: ENSG00000280441, MIR6724-3 (-54452), gene: ENSG00000274868 (-53918), gene: ENSG00000280614 (-48564), RNA5-8SN3 (-47146), gene: ENSG00000286267 (-24670), gene: ENSG00000286146 (-21902), gene: ENSG00000286054 (-20789), gene: ENSG00000286178 (-18834), MIR6724-4 (-10284), gene: ENSG00000278775 (-9731), gene: ENSG00000281181 (-4354), RNA5-8SN1 (-2930), gene: ENSG00000286149 (+19414), gene: ENSG00000286032 (+22348), gene: ENSG00000286091 (+23560), gene: ENSG00000286148 (+25414)

30927-006.R1 (paired) (Report)

Host chromosome	Unaligned ends virus	Broken reads host	Disrupted genes	Nearby genes
21	N/A	4	gene: ENSG00000280441	gene: ENSG00000280441, MIR6724-3 (-54721), gene: ENSG00000274868 (-54187), gene: ENSG00000280614 (-48833), RNA5-8SN3 (-47415), gene: ENSG00000286267 (-24939), gene: ENSG00000286146 (-22171), gene: ENSG00000286054 (-21058), gene: ENSG00000286178 (-19103), MIR6724-4 (-10553), gene: ENSG00000278775 (-10000), gene: ENSG00000281181 (-4623), RNA5-8SN1 (-3199), gene: ENSG00000286149 (+19145), gene: ENSG00000286032 (+22079), gene: ENSG00000286091 (+23291), gene: ENSG00000286148 (+25145)

30927-006.R1 (paired) (Report)

Host chromosome	Unaligned ends virus	Broken reads host	Disrupted genes	Nearby genes
21	N/A	4	gene: ENSG00000280441	gene: ENSG00000280441, MIR6724-3 (-54749), gene: ENSG00000274868 (-54215), gene: ENSG00000280614 (-48861), RNA5-8SN3 (-47443), gene: ENSG00000286267 (-24967), gene: ENSG00000286146 (-22199), gene: ENSG00000286054 (-21086), gene: ENSG00000286178 (-19131), MIR6724-4 (-10581), gene: ENSG00000278775 (-10028), gene: ENSG00000281181 (-4651), RNA5-8SN1 (-3227), gene: ENSG00000286149 (+19117), gene: ENSG00000286032 (+22051), gene: ENSG00000286091 (+23263), gene: ENSG00000286148 (+25117)

30927-006.R1 (paired) (Report)

Host chromosome	Unaligned ends virus	Broken reads host	Disrupted genes	Nearby genes
21	N/A	4	gene: ENSG00000280441	gene: ENSG00000280441, MIR6724-3 (-54792), gene: ENSG00000274868 (-54258), gene: ENSG00000280614 (-48904), RNA5-8SN3 (-47486), gene: ENSG00000286267 (-25010), gene: ENSG00000286146 (-22242), gene: ENSG00000286054 (-21129), gene: ENSG00000286178 (-19174), MIR6724-4 (-10624), gene: ENSG00000278775 (-10071), gene: ENSG00000281181 (-4694), RNA5-8SN1 (-3270), gene: ENSG00000286149 (+19074), gene: ENSG00000286032 (+22008), gene: ENSG00000286091 (+23220), gene: ENSG00000286148 (+25074)

30927-006.R1 (paired) (Report)

Host chromosome	Unaligned ends virus	Broken reads host	Disrupted genes	Nearby genes
21	N/A	4	gene: ENSG00000280441	gene: ENSG00000280441, MIR6724-3 (-54799), gene: ENSG00000274868 (-54265), gene: ENSG00000280614 (-48911), RNA5-8SN3 (-47493), gene: ENSG00000286267 (-25017), gene: ENSG00000286146 (-22249), gene: ENSG00000286054 (-21136), gene: ENSG00000286178 (-19181), MIR6724-4 (-10631), gene: ENSG00000278775 (-10078), gene: ENSG00000281181 (-4701), RNA5-8SN1 (-3277), gene: ENSG00000286149 (+19067), gene: ENSG00000286032 (+22001), gene: ENSG00000286091 (+23213), gene: ENSG00000286148 (+25067)

30927-006.R1 (paired) (Report)

Host chromosome	Unaligned ends virus	Broken reads host	Disrupted genes	Nearby genes
21	N/A	4	gene: ENSG00000280441	gene: ENSG00000280441, MIR6724-3 (-54722), gene: ENSG00000274868 (-54188), gene: ENSG00000280614 (-48834), RNA5-8SN3 (-47416), gene: ENSG00000286267 (-24940), gene: ENSG00000286146 (-22172), gene: ENSG00000286054 (-21059), gene: ENSG00000286178 (-19104), MIR6724-4 (-10554), gene: ENSG00000278775 (-10001), gene: ENSG00000281181 (-4624), RNA5-8SN1 (-3200), gene: ENSG00000286149 (+19144), gene: ENSG00000286032 (+22078), gene: ENSG00000286091 (+23290), gene: ENSG00000286148 (+25144)

30927-006.R1 (paired) (Report)

Host chromosome	Unaligned ends virus	Broken reads host	Disrupted genes	Nearby genes
21	758	1		gene: ENSG00000278996 (-32049), MIR6724-1 (-54289), gene: ENSG00000275664 (-53755), MIR3648-1 (-51043), gene: ENSG00000277437 (-50791), gene: ENSG00000280800 (-48389), RNA5-8SN2 (-46971), gene: ENSG00000286155 (-23863), gene: ENSG00000286252 (-21716), gene: ENSG00000286057 (-20603), gene: ENSG00000286012 (-18648), MIR6724-2 (-10099), gene: ENSG00000277671 (-9546), gene: ENSG00000281383 (-4181), 5_8S_rRNA (-2762)

30927-006.R1 (paired) (Report)

Host chromosome	Unaligned ends virus	Broken reads host	Disrupted genes	Nearby genes
21	498	3	gene: ENSG00000280441	gene: ENSG00000280441, MIR6724-3 (-13344), gene: ENSG00000274868 (-12810), gene: ENSG00000280614 (-7456), RNA5-8SN3 (-6038), gene: ENSG00000286267 (+15959), gene: ENSG00000286146 (+18877), gene: ENSG00000286054 (+20085), gene: ENSG00000286178 (+21962), MIR6724-4 (+30732), gene: ENSG00000278775 (+31287), gene: ENSG00000281181 (+35831), RNA5-8SN1 (+38025), gene: ENSG00000286149 (+60522), gene: ENSG00000286032 (+63456), gene: ENSG00000286091 (+64668), gene: ENSG00000286148 (+66522)

30927-006.R1 (paired) (Report)

Host chromosome	Unaligned ends virus	Broken reads host	Disrupted genes	Nearby genes
21	259	3	gene: ENSG00000280441	gene: ENSG00000280441, MIR6724-3 (-13347), gene: ENSG00000274868 (-12813), gene: ENSG00000280614 (-7459), RNA5-8SN3 (-6041), gene: ENSG00000286267 (+15956), gene: ENSG00000286146 (+18874), gene: ENSG00000286054 (+20082), gene: ENSG00000286178 (+21959), MIR6724-4 (+30729), gene: ENSG00000278775 (+31284), gene: ENSG00000281181 (+35828), RNA5-8SN1 (+38022), gene: ENSG00000286149 (+60519), gene: ENSG00000286032 (+63453), gene: ENSG00000286091 (+64665), gene: ENSG00000286148 (+66519)

30927-006.R1 (paired) (Report)

Host chromosome	Unaligned ends virus	Broken reads host	Disrupted genes	Nearby genes
21	N/A	6	gene: ENSG00000280441	gene: ENSG00000280441, MIR6724-3 (-13398), gene: ENSG00000274868 (-12864), gene: ENSG00000280614 (-7510), RNA5-8SN3 (-6092), gene: ENSG00000286267 (+15905), gene: ENSG00000286146 (+18823), gene: ENSG00000286054 (+20031), gene: ENSG00000286178 (+21908), MIR6724-4 (+30678), gene: ENSG00000278775 (+31233), gene: ENSG00000281181 (+35777), RNA5-8SN1 (+37971), gene: ENSG00000286149 (+60468), gene: ENSG00000286032 (+63402), gene: ENSG00000286091 (+64614), gene: ENSG00000286148 (+66468)

30927-006.R1 (paired) (Report)

Host chromosome	Unaligned ends virus	Broken reads host	Disrupted genes	Nearby genes
21	758	6	gene: ENSG00000280441	gene: ENSG00000280441, MIR6724-3 (-13401), gene: ENSG00000274868 (-12867), gene: ENSG00000280614 (-7513), RNA5-8SN3 (-6095), gene: ENSG00000286267 (+15902), gene: ENSG00000286146 (+18820), gene: ENSG00000286054 (+20028), gene: ENSG00000286178 (+21905), MIR6724-4 (+30675), gene: ENSG00000278775 (+31230), gene: ENSG00000281181 (+35774), RNA5-8SN1 (+37968), gene: ENSG00000286149 (+60465), gene: ENSG00000286032 (+63399), gene: ENSG00000286091 (+64611), gene: ENSG00000286148 (+66465)

30927-006.R1 (paired) (Report)

Host chromosome	Unaligned ends virus	Broken reads host	Disrupted genes	Nearby genes
21	N/A	6	gene: ENSG00000280441	gene: ENSG00000280441, MIR6724-3 (-13405), gene: ENSG00000274868 (-12871), gene: ENSG00000280614 (-7517), RNA5-8SN3 (-6099), gene: ENSG00000286267 (+15898), gene: ENSG00000286146 (+18816), gene: ENSG00000286054 (+20024), gene: ENSG00000286178 (+21901), MIR6724-4 (+30671), gene: ENSG00000278775 (+31226), gene: ENSG00000281181 (+35770), RNA5-8SN1 (+37964), gene: ENSG00000286149 (+60461), gene: ENSG00000286032 (+63395), gene: ENSG00000286091 (+64607), gene: ENSG00000286148 (+66461)

30927-006.R1 (paired) (Report)

Host chromosome	Unaligned ends virus	Broken reads host	Disrupted genes	Nearby genes
21	N/A	6	gene: ENSG00000280441	gene: ENSG00000280441, MIR6724-3 (-13419), gene: ENSG00000274868 (-12885), gene: ENSG00000280614 (-7531), RNA5-8SN3 (-6113), gene: ENSG00000286267 (+15884), gene: ENSG00000286146 (+18802), gene: ENSG00000286054 (+20010), gene: ENSG00000286178 (+21887), MIR6724-4 (+30657), gene: ENSG00000278775 (+31212), gene: ENSG00000281181 (+35756), RNA5-8SN1 (+37950), gene: ENSG00000286149 (+60447), gene: ENSG00000286032 (+63381), gene: ENSG00000286091 (+64593), gene: ENSG00000286148 (+66447)

30927-006.R1 (paired) (Report)

Host chromosome	Unaligned ends virus	Broken reads host	Disrupted genes	Nearby genes
21	N/A	1	gene: ENSG00000278996	gene: ENSG00000278996, MIR6724-1 (-10444), gene: ENSG00000275664 (-9910), MIR3648-1 (-7198), gene: ENSG00000277437 (-6946), gene: ENSG00000280800 (-4544), RNA5-8SN2 (-3126), gene: ENSG00000286155 (+19503), gene: ENSG00000286252 (+21798), gene: ENSG00000286057 (+23008), gene: ENSG00000286012 (+24885), MIR6724-2 (+33654), gene: ENSG00000277671 (+34209), gene: ENSG00000281383 (+38741), 5_8S_rRNA (+40930)

30927-006.R1 (paired) (Report)

Host chromosome	Unaligned ends virus	Broken reads host	Disrupted genes	Nearby genes
21	N/A	1	gene: ENSG00000280441	gene: ENSG00000280441, MIR6724-3 (-53926), gene: ENSG00000274868 (-53392), gene: ENSG00000280614 (-48038), RNA5-8SN3 (-46620), gene: ENSG00000286267 (-24144), gene: ENSG00000286146 (-21376), gene: ENSG00000286054 (-20263), gene: ENSG00000286178 (-18308), MIR6724-4 (-9758), gene: ENSG00000278775 (-9205), gene: ENSG00000281181 (-3828), RNA5-8SN1 (-2404), gene: ENSG00000286149 (+19940), gene: ENSG00000286032 (+22874), gene: ENSG00000286091 (+24086), gene: ENSG00000286148 (+25940)

30927-006.R1 (paired) (Report)

Host chromosome	Unaligned ends virus	Broken reads host	Disrupted genes	Nearby genes
21	27	2	gene: ENSG00000280441	gene: ENSG00000280441, MIR6724-3 (-53968), gene: ENSG00000274868 (-53434), gene: ENSG00000280614 (-48080), RNA5-8SN3 (-46662), gene: ENSG00000286267 (-24186), gene: ENSG00000286146 (-21418), gene: ENSG00000286054 (-20305), gene: ENSG00000286178 (-18350), MIR6724-4 (-9800), gene: ENSG00000278775 (-9247), gene: ENSG00000281181 (-3870), RNA5-8SN1 (-2446), gene: ENSG00000286149 (+19898), gene: ENSG00000286032 (+22832), gene: ENSG00000286091 (+24044), gene: ENSG00000286148 (+25898)

30927-006.R1 (paired) (Report)

Host chromosome	Unaligned ends virus	Broken reads host	Disrupted genes	Nearby genes
21	N/A	2	gene: ENSG00000280441	gene: ENSG00000280441, MIR6724-3 (-53971), gene: ENSG00000274868 (-53437), gene: ENSG00000280614 (-48083), RNA5-8SN3 (-46665), gene: ENSG00000286267 (-24189), gene: ENSG00000286146 (-21421), gene: ENSG00000286054 (-20308), gene: ENSG00000286178 (-18353), MIR6724-4 (-9803), gene: ENSG00000278775 (-9250), gene: ENSG00000281181 (-3873), RNA5-8SN1 (-2449), gene: ENSG00000286149 (+19895), gene: ENSG00000286032 (+22829), gene: ENSG00000286091 (+24041), gene: ENSG00000286148 (+25895)

30927-006.R1 (paired) (Report)

Host chromosome	Unaligned ends virus	Broken reads host	Disrupted genes	Nearby genes
21	N/A	2	gene: ENSG00000280441	gene: ENSG00000280441, MIR6724-3 (-54002), gene: ENSG00000274868 (-53468), gene: ENSG00000280614 (-48114), RNA5-8SN3 (-46696), gene: ENSG00000286267 (-24220), gene: ENSG00000286146 (-21452), gene: ENSG00000286054 (-20339), gene: ENSG00000286178 (-18384), MIR6724-4 (-9834), gene: ENSG00000278775 (-9281), gene: ENSG00000281181 (-3904), RNA5-8SN1 (-2480), gene: ENSG00000286149 (+19864), gene: ENSG00000286032 (+22798), gene: ENSG00000286091 (+24010), gene: ENSG00000286148 (+25864)

30927-006.R1 (paired) (Report)

Host chromosome	Unaligned ends virus	Broken reads host	Disrupted genes	Nearby genes
21	343	2	gene: ENSG00000280441	gene: ENSG00000280441, MIR6724-3 (-54004), gene: ENSG00000274868 (-53470), gene: ENSG00000280614 (-48116), RNA5-8SN3 (-46698), gene: ENSG00000286267 (-24222), gene: ENSG00000286146 (-21454), gene: ENSG00000286054 (-20341), gene: ENSG00000286178 (-18386), MIR6724-4 (-9836), gene: ENSG00000278775 (-9283), gene: ENSG00000281181 (-3906), RNA5-8SN1 (-2482), gene: ENSG00000286149 (+19862), gene: ENSG00000286032 (+22796), gene: ENSG00000286091 (+24008), gene: ENSG00000286148 (+25862)

30927-006.R1 (paired) (Report)

Host chromosome	Unaligned ends virus	Broken reads host	Disrupted genes	Nearby genes
21	N/A	2	gene: ENSG00000280441	gene: ENSG00000280441, MIR6724-3 (-54005), gene: ENSG00000274868 (-53471), gene: ENSG00000280614 (-48117), RNA5-8SN3 (-46699), gene: ENSG00000286267 (-24223), gene: ENSG00000286146 (-21455), gene: ENSG00000286054 (-20342), gene: ENSG00000286178 (-18387), MIR6724-4 (-9837), gene: ENSG00000278775 (-9284), gene: ENSG00000281181 (-3907), RNA5-8SN1 (-2483), gene: ENSG00000286149 (+19861), gene: ENSG00000286032 (+22795), gene: ENSG00000286091 (+24007), gene: ENSG00000286148 (+25861)

30927-006.R1 (paired) (Report)

Host chromosome	Unaligned ends virus	Broken reads host	Disrupted genes	Nearby genes
21	N/A	2	gene: ENSG00000280441	gene: ENSG00000280441, MIR6724-3 (-54006), gene: ENSG00000274868 (-53472), gene: ENSG00000280614 (-48118), RNA5-8SN3 (-46700), gene: ENSG00000286267 (-24224), gene: ENSG00000286146 (-21456), gene: ENSG00000286054 (-20343), gene: ENSG00000286178 (-18388), MIR6724-4 (-9838), gene: ENSG00000278775 (-9285), gene: ENSG00000281181 (-3908), RNA5-8SN1 (-2484), gene: ENSG00000286149 (+19860), gene: ENSG00000286032 (+22794), gene: ENSG00000286091 (+24006), gene: ENSG00000286148 (+25860)

30927-006.R1 (paired) (Report)

Host chromosome	Unaligned ends virus	Broken reads host	Disrupted genes	Nearby genes
21	758	2	gene: ENSG00000280441	gene: ENSG00000280441, MIR6724-3 (-54007), gene: ENSG00000274868 (-53473), gene: ENSG00000280614 (-48119), RNA5-8SN3 (-46701), gene: ENSG00000286267 (-24225), gene: ENSG00000286146 (-21457), gene: ENSG00000286054 (-20344), gene: ENSG00000286178 (-18389), MIR6724-4 (-9839), gene: ENSG00000278775 (-9286), gene: ENSG00000281181 (-3909), RNA5-8SN1 (-2485), gene: ENSG00000286149 (+19859), gene: ENSG00000286032 (+22793), gene: ENSG00000286091 (+24005), gene: ENSG00000286148 (+25859)

30927-006.R1 (paired) (Report)

Host chromosome	Unaligned ends virus	Broken reads host	Disrupted genes	Nearby genes
21	N/A	5	gene: ENSG00000280441	gene: ENSG00000280441, MIR6724-3 (-54276), gene: ENSG00000274868 (-53742), gene: ENSG00000280614 (-48388), RNA5-8SN3 (-46970), gene: ENSG00000286267 (-24494), gene: ENSG00000286146 (-21726), gene: ENSG00000286054 (-20613), gene: ENSG00000286178 (-18658), MIR6724-4 (-10108), gene: ENSG00000278775 (-9555), gene: ENSG00000281181 (-4178), RNA5-8SN1 (-2754), gene: ENSG00000286149 (+19590), gene: ENSG00000286032 (+22524), gene: ENSG00000286091 (+23736), gene: ENSG00000286148 (+25590)
22	N/A	1	PPIL2	UBE2L3 (-71741), YDJC (-65711), CCDC116 (-58446), gene: ENSG00000273342 (-54491), SDF2L1 (-51476), gene: ENSG00000207751 (-25538), MIR301B (-42717), MIR130B (-42390), gene: ENSG00000272954 (-33412), PPIL2, YPEL1 (+1760), gene: ENSG00000286365 (+16985), RN7SL280P (+27119), gene: ENSG00000286127 (+41672), MAPK1 (+63881)

1. Identify Viral Integration Sites summary

Input reads	4,011,050
Host reads	2,301,829
Virus reads	704,442
Unmapped reads	1,004,779
Host reads (%)	57.39
Virus reads (%)	17.56
Unmapped reads (%)	25.05
Breakpoints identified	182
Viruses identified	1

2. Identify Viral Integration Sites virus content

Virus	Reads mapped
HPV16REF	358,455

3. Identify Viral Integration Sites breakpoint summary

Host chromosome	Host region	Virus	Virus region	Unaligned ends host
6	52995915..52995916	HPV16REF	5759..5760	74
8	complement (144139926.. 144139927)	HPV16REF	3096..3097	134
8	complement (144139965.. 144139966)	HPV16REF	3773..3774	194
8	complement (144139973.. 144139974)	HPV16REF	7261..7262	214
8	complement (144139975.. 144139976)	HPV16REF	3559..3560	405
8	complement (144139978.. 144139979)	HPV16REF	6650..6651	122
8	complement (144139981.. 144139982)	HPV16REF	4724..4725	190

30927-007.R1 (paired) (Report)

Host chromosome	Host region	Virus	Virus region	Unaligned ends host
8	144140118..<br>144140119	HPV16REF	3394..3395	251
8	144140119..<br>144140120	HPV16REF	4623..4624	220
8	144140120..<br>144140121	HPV16REF	1803..1804	368
8	144140130..<br>144140131	HPV16REF	1051..1052	230
8	144140143..<br>144140144	HPV16REF	3868..3869	197
8	144140159..<br>144140160	HPV16REF	5344..5345	832
8	144140175..<br>144140176	HPV16REF	2640..2641	189
8	144140223..<br>144140224	HPV16REF	3233..3234	89
8	144140227..<br>144140228	HPV16REF	1036..1037	283
8	144140208..<br>144140209	HPV16REF	5836..5837	105
14	complement<br>(49862565..49862566)	HPV16REF	complement(665..666)	35
14	complement<br>(49862573..49862574)	HPV16REF	3313..3314	78
14	49862804..49862805	HPV16REF	880..881	44
21	complement<br>(8258196..8258197)	HPV16REF	3576..3577	181
21	complement<br>(8258197..8258198)	HPV16REF	1535..1536	203
21	complement<br>(8258203..8258204)	HPV16REF	complement(131..132)	98
21	complement<br>(8259473..8259474)	HPV16REF	1613..1614	542
21	complement<br>(8259493..8259494)	HPV16REF	1582..1583	185
21	complement<br>(8259509..8259510)	HPV16REF	3713..3714	246
21	complement<br>(8259538..8259539)	HPV16REF	232..233	164
21	complement<br>(8259575..8259576)	HPV16REF	complement(406..407)	146
21	complement<br>(8259615..8259616)	HPV16REF	2170..2171	233
21	complement<br>(8259871..8259872)	HPV16REF	1530..1531	321

30927-007.R1 (paired) (Report)

Host chromosome	Host region	Virus	Virus region	Unaligned ends host
21	complement (8260081..8260082)	HPV16REF	4101..4102	212
21	complement (8260084..8260085)	HPV16REF	1454..1455	246
21	complement (8260544..8260545)	HPV16REF	5503..5504	95
21	complement (8394061..8394062)	HPV16REF	4686..4687	166
21	complement (8394120..8394121)	HPV16REF	7298..7299	95
21	complement (8397020..8397021)	HPV16REF	3787..3788	91
21	complement (8398296..8398297)	HPV16REF	2382..2383	1,103
21	complement (8398298..8398299)	HPV16REF	1647..1648	193
21	complement (8398314..8398315)	HPV16REF	1453..1454	283
21	complement (8398352..8398353)	HPV16REF	4752..4753	114
21	complement (8398379..8398380)	HPV16REF	complement(583..584)	121
21	complement (8398380..8398381)	HPV16REF	3800..3801	122
21	complement (8398391..8398392)	HPV16REF	4416..4417	300
21	complement (8398435..8398436)	HPV16REF	3247..3248	258
21	complement (8398445..8398446)	HPV16REF	4963..4964	445
21	complement (8398464..8398465)	HPV16REF	complement(583..584)	285
21	complement (8398891..8398892)	HPV16REF	3068..3069	185
21	complement (8398894..8398895)	HPV16REF	complement(713..714)	103
21	complement (8401560..8401561)	HPV16REF	3363..3364	209
21	complement (8401569..8401570)	HPV16REF	3468..3469	95
21	complement (8401587..8401588)	HPV16REF	complement(713..714)	571
21	complement (8401634..8401635)	HPV16REF	1989..1990	91
21	complement (8401635..8401636)	HPV16REF	880..881	104

30927-007.R1 (paired) (Report)

Host chromosome	Host region	Virus	Virus region	Unaligned ends host
21	complement (8401645..8401646)	HPV16REF	1395..1396	136
21	complement (8438270..8438271)	HPV16REF	complement(108..109)	179
21	complement (8441174..8441175)	HPV16REF	1852..1853	256
21	complement (8441193..8441194)	HPV16REF	complement(665..666)	213
21	complement (8441201..8441202)	HPV16REF	1768..1769	92
21	complement (8441234..8441235)	HPV16REF	2389..2390	170
21	complement (8441578..8441579)	HPV16REF	3755..3756	328
21	complement (8442121..8442122)	HPV16REF	2510..2511	395
21	complement (8442179..8442180)	HPV16REF	6365..6366	118
21	complement (8442209..8442210)	HPV16REF	2473..2474	156
21	complement (8442217..8442218)	HPV16REF	6338..6339	175
21	complement (8442254..8442255)	HPV16REF	7247..7248	124
21	complement (8442257..8442258)	HPV16REF	6378..6379	193
21	complement (8442260..8442261)	HPV16REF	5473..5474	167
21	complement (8442268..8442269)	HPV16REF	1449..1450	313
21	complement (8442292..8442293)	HPV16REF	2742..2743	157
21	complement (8442300..8442301)	HPV16REF	4003..4004	265
21	complement (8442301..8442302)	HPV16REF	complement(523..524)	181
21	complement (8442305..8442306)	HPV16REF	1890..1891	388
21	complement (8442306..8442307)	HPV16REF	6055..6056	193
21	complement (8442307..8442308)	HPV16REF	7418..7419	163
21	complement (8442312..8442313)	HPV16REF	2572..2573	196
21	complement (8442313..8442314)	HPV16REF	2825..2826	204

30927-007.R1 (paired) (Report)

Host chromosome	Host region	Virus	Virus region	Unaligned ends host
21	complement (8442317..8442318)	HPV16REF	880..881	192
21	complement (8442320..8442321)	HPV16REF	complement(623..624)	269
21	complement (8442323..8442324)	HPV16REF	complement(665..666)	336
21	complement (8442324..8442325)	HPV16REF	complement(665..666)	202
21	complement (8442334..8442335)	HPV16REF	3409..3410	178
21	complement (8442352..8442353)	HPV16REF	2204..2205	123
21	complement (8442356..8442357)	HPV16REF	1318..1319	227
21	complement (8442471..8442472)	HPV16REF	4053..4054	127
21	complement (8442475..8442476)	HPV16REF	2954..2955	178
21	complement (8442500..8442501)	HPV16REF	3545..3546	301
21	complement (8442539..8442540)	HPV16REF	3033..3034	308
21	complement (8442590..8442591)	HPV16REF	2201..2202	166
21	complement (8442598..8442599)	HPV16REF	1330..1331	225
21	complement (8442643..8442644)	HPV16REF	7857..7858	237
21	complement (8442663..8442664)	HPV16REF	7204..7205	264
21	complement (8442710..8442711)	HPV16REF	7894..7895	363
21	complement (8442904..8442905)	HPV16REF	5481..5482	130
21	complement (8442905..8442906)	HPV16REF	3252..3253	298
21	complement (8442950..8442951)	HPV16REF	702..703	117
21	complement (8442951..8442952)	HPV16REF	2716..2717	97
21	complement (8442968..8442969)	HPV16REF	complement(623..624)	430
21	complement (8443122..8443123)	HPV16REF	3960..3961	369
21	complement (8443123..8443124)	HPV16REF	complement(527..528)	245

30927-007.R1 (paired) (Report)

Host chromosome	Host region	Virus	Virus region	Unaligned ends host
21	complement (8443129..8443130)	HPV16REF	3812..3813	501
21	complement (8443130..8443131)	HPV16REF	1565..1566	170
21	complement (8443144..8443145)	HPV16REF	6990..6991	118
21	complement (8443150..8443151)	HPV16REF	6299..6300	254
21	complement (8443174..8443175)	HPV16REF	2463..2464	234
21	complement (8443181..8443182)	HPV16REF	7876..7877	314
21	complement (8443192..8443193)	HPV16REF	865..866	235
21	complement (8443195..8443196)	HPV16REF	865..866	103
21	complement (8443196..8443197)	HPV16REF	7827..7828	164
21	complement (8443202..8443203)	HPV16REF	7661..7662	242
21	complement (8443204..8443205)	HPV16REF	4419..4420	337
21	complement (8443245..8443246)	HPV16REF	5912..5913	598
21	complement (8443251..8443252)	HPV16REF	5790..5791	335
21	complement (8443297..8443298)	HPV16REF	7432..7433	129
21	complement (8445110..8445111)	HPV16REF	865..866	110
21	complement (8445808..8445809)	HPV16REF	865..866	130
21	complement (8445819..8445820)	HPV16REF	complement(406..407)	1,200
21	complement (8445829..8445830)	HPV16REF	2528..2529	183
21	complement (8445842..8445843)	HPV16REF	2258..2259	1,625
21	complement (8445849..8445850)	HPV16REF	3964..3965	129
21	complement (8445860..8445861)	HPV16REF	3256..3257	136
21	complement (8445879..8445880)	HPV16REF	6445..6446	135
21	complement (8445912..8445913)	HPV16REF	2402..2403	131

30927-007.R1 (paired) (Report)

Host chromosome	Host region	Virus	Virus region	Unaligned ends host
21	complement (8445931..8445932)	HPV16REF	880..881	894
21	complement (8445956..8445957)	HPV16REF	232..233	497
21	complement (8445980..8445981)	HPV16REF	5632..5633	167
21	complement (8445981..8445982)	HPV16REF	6740..6741	236
21	complement (15263528..15263529)	HPV16REF	865..866	142
21	complement (15263533..15263534)	HPV16REF	complement(108..109)	110
21	complement (15263538..15263539)	HPV16REF	232..233	114
21	complement (8988563..8988564)	HPV16REF	3388..3389	118
21	complement (8211100..8211101)	HPV16REF	4493..4494	319
21	complement (8211138..8211139)	HPV16REF	4213..4214	160
21	complement (8215207..8215208)	HPV16REF	1560..1561	103
21	complement (8215232..8215233)	HPV16REF	5492..5493	140
21	complement (8215236..8215237)	HPV16REF	1989..1990	309
21	complement (8215255..8215256)	HPV16REF	2077..2078	120
21	complement (8215275..8215276)	HPV16REF	2668..2669	159
21	complement (8215280..8215281)	HPV16REF	2333..2334	268
21	complement (8215304..8215305)	HPV16REF	5486..5487	172
21	complement (8215307..8215308)	HPV16REF	6688..6689	179
21	complement (8215309..8215310)	HPV16REF	2993..2994	150
21	complement (8215335..8215336)	HPV16REF	3780..3781	191
21	complement (8215386..8215387)	HPV16REF	3126..3127	236
21	complement (8215401..8215402)	HPV16REF	1546..1547	243
21	complement (8215402..8215403)	HPV16REF	4724..4725	259

30927-007.R1 (paired) (Report)

Host chromosome	Host region	Virus	Virus region	Unaligned ends host
21	complement (8215411..8215412)	HPV16REF	2289..2290	465
21	complement (8215447..8215448)	HPV16REF	2425..2426	350
21	complement (8215485..8215486)	HPV16REF	5909..5910	135
21	complement (8215705..8215706)	HPV16REF	complement(713..714)	391
21	complement (8215860..8215861)	HPV16REF	complement(713..714)	105
21	complement (8215859..8215860)	HPV16REF	2946..2947	190
21	complement (8215861..8215862)	HPV16REF	1054..1055	150
21	complement (8216407..8216408)	HPV16REF	7687..7688	555
21	complement (8216781..8216782)	HPV16REF	865..866	101
21	complement (8216785..8216786)	HPV16REF	1007..1008	138
21	complement (8218516..8218517)	HPV16REF	5588..5589	343
21	complement (8218530..8218531)	HPV16REF	3605..3606	104
21	complement (8218559..8218560)	HPV16REF	5786..5787	195
21	complement (8218585..8218586)	HPV16REF	2569..2570	139
21	complement (8218588..8218589)	HPV16REF	865..866	94
21	complement (8218608..8218609)	HPV16REF	4032..4033	195
21	complement (8218610..8218611)	HPV16REF	6674..6675	757
21	complement (8218616..8218617)	HPV16REF	3111..3112	302
21	complement (8218628..8218629)	HPV16REF	3218..3219	177
21	complement (8218638..8218639)	HPV16REF	5821..5822	98
21	complement (8218657..8218658)	HPV16REF	3421..3422	138
21	complement (8218659..8218660)	HPV16REF	7869..7870	470
21	complement (8218661..8218662)	HPV16REF	880..881	893

30927-007.R1 (paired) (Report)

Host chromosome	Host region	Virus	Virus region	Unaligned ends host
21	complement (15315692..15315693)	HPV16REF	1989..1990	261
21	complement (8254051..8254052)	HPV16REF	6091..6092	91
21	8438523..8438524	HPV16REF	1896..1897	1,027
21	15256382..15256383	HPV16REF	880..881	1,115
21	8442379..8442380	HPV16REF	3844..3845	820
21	8442421..8442422	HPV16REF	4656..4657	689
21	8442457..8442458	HPV16REF	1503..1504	863
21	8442458..8442459	HPV16REF	1282..1283	719
21	8442460..8442461	HPV16REF	1457..1458	1,031
21	8442461..8442462	HPV16REF	5943..5944	793
21	8443395..8443396	HPV16REF	2269..2270	7,770
21	15263668..15263669	HPV16REF	232..233	2,462
21	15263669..15263670	HPV16REF	880..881	12,549
21	15273220..15273221	HPV16REF	1989..1990	1,105

Host chromosome	Unaligned ends virus	Broken reads host	Disrupted genes	Nearby genes
6	N/A	1	7SK, RN7SK	GSTA3 (-86217), GSTA4 (-611), 7SK, RN7SK, CILK1 (+5363), gene: ENSG00000288614 (+45350), FBXO9 (+56075), gene: ENSG00000288646 (+69686), RN7SL244P (+95045)
8	N/A	1	HGH1	SPATC1 (-92812), OPLAH (-75961), MIR6846 (-82546), gene: ENSG00000255224 (-60661), EXOSC4 (-59278), MIR6847 (-59984), GPAA1 (-53710), CYC1 (-42401), SHARPIN (-31802), MAF1 (-32315), WDR97 (-21598), HGH1, MROH1 (+8089)

30927-007.R1 (paired) (Report)

Host chromosome	Unaligned ends virus	Broken reads host	Disrupted genes	Nearby genes
8	N/A	1	HGH1	SPATC1 (-92851), OPLAH (-76000), MIR6846 (-82585), gene: ENSG00000255224 (-60700), EXOSC4 (-59317), MIR6847 (-60023), GPAA1 (-53749), CYC1 (-42440), SHARPIN (-31841), MAF1 (-32354), WDR97 (-21637), HGH1, MROH1 (+8050)
8	N/A	1	HGH1	SPATC1 (-92859), OPLAH (-76008), MIR6846 (-82593), gene: ENSG00000255224 (-60708), EXOSC4 (-59325), MIR6847 (-60031), GPAA1 (-53757), CYC1 (-42448), SHARPIN (-31849), MAF1 (-32362), WDR97 (-21645), HGH1, MROH1 (+8042)
8	N/A	1	HGH1	SPATC1 (-92861), OPLAH (-76010), MIR6846 (-82595), gene: ENSG00000255224 (-60710), EXOSC4 (-59327), MIR6847 (-60033), GPAA1 (-53759), CYC1 (-42450), SHARPIN (-31851), MAF1 (-32364), WDR97 (-21647), HGH1, MROH1 (+8040)

30927-007.R1 (paired) (Report)

Host chromosome	Unaligned ends virus	Broken reads host	Disrupted genes	Nearby genes
8	N/A	1	HGH1	SPATC1 (-92864), OPLAH (-76013), MIR6846 (-82598), gene: ENSG00000255224 (-60713), EXOSC4 (-59330), MIR6847 (-60036), GPAA1 (-53762), CYC1 (-42453), SHARPIN (-31854), MAF1 (-32367), WDR97 (-21650), HGH1, MROH1 (+8037)
8	N/A	1	HGH1	SPATC1 (-92867), OPLAH (-76016), MIR6846 (-82601), gene: ENSG00000255224 (-60716), EXOSC4 (-59333), MIR6847 (-60039), GPAA1 (-53765), CYC1 (-42456), SHARPIN (-31857), MAF1 (-32370), WDR97 (-21653), HGH1, MROH1 (+8034)
8	N/A	1	HGH1	SPATC1 (-93004), OPLAH (-76153), MIR6846 (-82738), gene: ENSG00000255224 (-60853), EXOSC4 (-59470), MIR6847 (-60176), GPAA1 (-53902), CYC1 (-42593), SHARPIN (-31994), MAF1 (-32507), WDR97 (-21790), HGH1, MROH1 (+7897)

30927-007.R1 (paired) (Report)

Host chromosome	Unaligned ends virus	Broken reads host	Disrupted genes	Nearby genes
8	N/A	1	HGH1	SPATC1 (-93005), OPLAH (-76154), MIR6846 (-82739), gene: ENSG00000255224 (-60854), EXOSC4 (-59471), MIR6847 (-60177), GPAA1 (-53903), CYC1 (-42594), SHARPIN (-31995), MAF1 (-32508), WDR97 (-21791), HGH1, MROH1 (+7896)
8	N/A	1	HGH1	SPATC1 (-93006), OPLAH (-76155), MIR6846 (-82740), gene: ENSG00000255224 (-60855), EXOSC4 (-59472), MIR6847 (-60178), GPAA1 (-53904), CYC1 (-42595), SHARPIN (-31996), MAF1 (-32509), WDR97 (-21792), HGH1, MROH1 (+7895)
8	N/A	1	HGH1	SPATC1 (-93016), OPLAH (-76165), MIR6846 (-82750), gene: ENSG00000255224 (-60865), EXOSC4 (-59482), MIR6847 (-60188), GPAA1 (-53914), CYC1 (-42605), SHARPIN (-32006), MAF1 (-32519), WDR97 (-21802), HGH1, MROH1 (+7885)

30927-007.R1 (paired) (Report)

Host chromosome	Unaligned ends virus	Broken reads host	Disrupted genes	Nearby genes
8	N/A	1	HGH1	SPATC1 (-93029), OPLAH (-76178), MIR6846 (-82763), gene: ENSG00000255224 (-60878), EXOSC4 (-59495), MIR6847 (-60201), GPAA1 (-53927), CYC1 (-42618), SHARPIN (-32019), MAF1 (-32532), WDR97 (-21815), HGH1, MROH1 (+7872)
8	N/A	1	HGH1	SPATC1 (-93045), OPLAH (-76194), MIR6846 (-82779), gene: ENSG00000255224 (-60894), EXOSC4 (-59511), MIR6847 (-60217), GPAA1 (-53943), CYC1 (-42634), SHARPIN (-32035), MAF1 (-32548), WDR97 (-21831), HGH1, MROH1 (+7856)
8	N/A	1	HGH1	SPATC1 (-93061), OPLAH (-76210), MIR6846 (-82795), gene: ENSG00000255224 (-60910), EXOSC4 (-59527), MIR6847 (-60233), GPAA1 (-53959), CYC1 (-42650), SHARPIN (-32051), MAF1 (-32564), WDR97 (-21847), HGH1, MROH1 (+7840)

30927-007.R1 (paired) (Report)

Host chromosome	Unaligned ends virus	Broken reads host	Disrupted genes	Nearby genes
8	N/A	1	HGH1	SPATC1 (-93109), OPLAH (-76258), MIR6846 (-82843), gene: ENSG00000255224 (-60958), EXOSC4 (-59575), MIR6847 (-60281), GPAA1 (-54007), CYC1 (-42698), SHARPIN (-32099), MAF1 (-32612), WDR97 (-21895), HGH1, MROH1 (+7792)
8	N/A	1	HGH1	SPATC1 (-93113), OPLAH (-76262), MIR6846 (-82847), gene: ENSG00000255224 (-60962), EXOSC4 (-59579), MIR6847 (-60285), GPAA1 (-54011), CYC1 (-42702), SHARPIN (-32103), MAF1 (-32616), WDR97 (-21899), HGH1, MROH1 (+7788)
8	N/A	1	HGH1	SPATC1 (-93094), OPLAH (-76243), MIR6846 (-82828), gene: ENSG00000255224 (-60943), EXOSC4 (-59560), MIR6847 (-60266), GPAA1 (-53992), CYC1 (-42683), SHARPIN (-32084), MAF1 (-32597), WDR97 (-21880), HGH1, MROH1 (+7807)
14	2,332	2	gene: ENSG00000282885, RN7SL2	KLHDC2 (-76180), NEMF (-9744), Y_RNA (-23072), RNU6-539P (-22738), RN7SL3 (-8651), gene: ENSG00000282885, RN7SL2, gene: ENSG00000278002 (+506), ARF6 (+30516), RNU6-189P (+39284), Metazoa_SRP (+50927), LINC01588 (+65005)

30927-007.R1 (paired) (Report)

Host chromosome	Unaligned ends virus	Broken reads host	Disrupted genes	Nearby genes
14	N/A	2	gene: ENSG00000282885, RN7SL2	KLHDC2 (-76188), NEMF (-9752), Y_RNA (-23080), RNU6-539P (-22746), RN7SL3 (-8659), gene: ENSG00000282885, RN7SL2, gene: ENSG00000278002 (+498), ARF6 (+30508), RNU6-189P (+39276), Metazoa_SRP (+50919), LINC01588 (+64997)
14	41,362	2	gene: ENSG00000282885, RN7SL2	KLHDC2 (-76419), NEMF (-9983), Y_RNA (-23311), RNU6-539P (-22977), RN7SL3 (-8890), gene: ENSG00000282885, RN7SL2, gene: ENSG00000278002 (+267), ARF6 (+30277), RNU6-189P (+39045), Metazoa_SRP (+50688), LINC01588 (+64766)
21	N/A	1		gene: ENSG00000278996 (-30550), MIR6724-1 (-52790), gene: ENSG00000275664 (-52256), MIR3648-1 (-49544), gene: ENSG00000277437 (-49292), gene: ENSG00000280800 (-46890), RNA5-8SN2 (-45472), gene: ENSG00000286155 (-22364), gene: ENSG00000286252 (-20217), gene: ENSG00000286057 (-19104), gene: ENSG00000286012 (-17149), MIR6724-2 (-8600), gene: ENSG00000277671 (-8047), gene: ENSG00000281383 (-2682), 5_8S_rRNA (-1263)

30927-007.R1 (paired) (Report)

Host chromosome	Unaligned ends virus	Broken reads host	Disrupted genes	Nearby genes
21	N/A	1		gene: ENSG00000278996 (-30551), MIR6724-1 (-52791), gene: ENSG00000275664 (-52257), MIR3648-1 (-49545), gene: ENSG00000277437 (-49293), gene: ENSG00000280800 (-46891), RNA5-8SN2 (-45473), gene: ENSG00000286155 (-22365), gene: ENSG00000286252 (-20218), gene: ENSG00000286057 (-19105), gene: ENSG00000286012 (-17150), MIR6724-2 (-8601), gene: ENSG00000277671 (-8048), gene: ENSG00000281383 (-2683), 5_8S_rRNA (-1264)
21	1,213	1		gene: ENSG00000278996 (-30557), MIR6724-1 (-52797), gene: ENSG00000275664 (-52263), MIR3648-1 (-49551), gene: ENSG00000277437 (-49299), gene: ENSG00000280800 (-46897), RNA5-8SN2 (-45479), gene: ENSG00000286155 (-22371), gene: ENSG00000286252 (-20224), gene: ENSG00000286057 (-19111), gene: ENSG00000286012 (-17156), MIR6724-2 (-8607), gene: ENSG00000277671 (-8054), gene: ENSG00000281383 (-2689), 5_8S_rRNA (-1270)

30927-007.R1 (paired) (Report)

Host chromosome	Unaligned ends virus	Broken reads host	Disrupted genes	Nearby genes
21	N/A	7		gene: ENSG00000278996 (-31827), MIR6724-1 (-54067), gene: ENSG00000275664 (-53533), MIR3648-1 (-50821), gene: ENSG00000277437 (-50569), gene: ENSG00000280800 (-48167), RNA5-8SN2 (-46749), gene: ENSG00000286155 (-23641), gene: ENSG00000286252 (-21494), gene: ENSG00000286057 (-20381), gene: ENSG00000286012 (-18426), MIR6724-2 (-9877), gene: ENSG00000277671 (-9324), gene: ENSG00000281383 (-3959), 5_8S_rRNA (-2540)
21	N/A	6		gene: ENSG00000278996 (-31847), MIR6724-1 (-54087), gene: ENSG00000275664 (-53553), MIR3648-1 (-50841), gene: ENSG00000277437 (-50589), gene: ENSG00000280800 (-48187), RNA5-8SN2 (-46769), gene: ENSG00000286155 (-23661), gene: ENSG00000286252 (-21514), gene: ENSG00000286057 (-20401), gene: ENSG00000286012 (-18446), MIR6724-2 (-9897), gene: ENSG00000277671 (-9344), gene: ENSG00000281383 (-3979), 5_8S_rRNA (-2560)

30927-007.R1 (paired) (Report)

Host chromosome	Unaligned ends virus	Broken reads host	Disrupted genes	Nearby genes
21	N/A	5		gene: ENSG00000278996 (-31863), MIR6724-1 (-54103), gene: ENSG00000275664 (-53569), MIR3648-1 (-50857), gene: ENSG00000277437 (-50605), gene: ENSG00000280800 (-48203), RNA5-8SN2 (-46785), gene: ENSG00000286155 (-23677), gene: ENSG00000286252 (-21530), gene: ENSG00000286057 (-20417), gene: ENSG00000286012 (-18462), MIR6724-2 (-9913), gene: ENSG00000277671 (-9360), gene: ENSG00000281383 (-3995), 5_8S_rRNA (-2576)
21	2,762	6		gene: ENSG00000278996 (-31892), MIR6724-1 (-54132), gene: ENSG00000275664 (-53598), MIR3648-1 (-50886), gene: ENSG00000277437 (-50634), gene: ENSG00000280800 (-48232), RNA5-8SN2 (-46814), gene: ENSG00000286155 (-23706), gene: ENSG00000286252 (-21559), gene: ENSG00000286057 (-20446), gene: ENSG00000286012 (-18491), MIR6724-2 (-9942), gene: ENSG00000277671 (-9389), gene: ENSG00000281383 (-4024), 5_8S_rRNA (-2605)

30927-007.R1 (paired) (Report)

Host chromosome	Unaligned ends virus	Broken reads host	Disrupted genes	Nearby genes
21	38,981	4		gene: ENSG00000278996 (-31929), MIR6724-1 (-54169), gene: ENSG00000275664 (-53635), MIR3648-1 (-50923), gene: ENSG00000277437 (-50671), gene: ENSG00000280800 (-48269), RNA5-8SN2 (-46851), gene: ENSG00000286155 (-23743), gene: ENSG00000286252 (-21596), gene: ENSG00000286057 (-20483), gene: ENSG00000286012 (-18528), MIR6724-2 (-9979), gene: ENSG00000277671 (-9426), gene: ENSG00000281383 (-4061), 5_8S_rRNA (-2642)
21	N/A	3		gene: ENSG00000278996 (-31969), MIR6724-1 (-54209), gene: ENSG00000275664 (-53675), MIR3648-1 (-50963), gene: ENSG00000277437 (-50711), gene: ENSG00000280800 (-48309), RNA5-8SN2 (-46891), gene: ENSG00000286155 (-23783), gene: ENSG00000286252 (-21636), gene: ENSG00000286057 (-20523), gene: ENSG00000286012 (-18568), MIR6724-2 (-10019), gene: ENSG00000277671 (-9466), gene: ENSG00000281383 (-4101), 5_8S_rRNA (-2682)

30927-007.R1 (paired) (Report)

Host chromosome	Unaligned ends virus	Broken reads host	Disrupted genes	Nearby genes
21	N/A	4		gene: ENSG00000278996 (-32225), MIR6724-1 (-54465), gene: ENSG00000275664 (-53931), MIR3648-1 (-51219), gene: ENSG00000277437 (-50967), gene: ENSG00000280800 (-48565), RNA5-8SN2 (-47147), gene: ENSG00000286155 (-24039), gene: ENSG00000286252 (-21892), gene: ENSG00000286057 (-20779), gene: ENSG00000286012 (-18824), MIR6724-2 (-10275), gene: ENSG00000277671 (-9722), gene: ENSG00000281383 (-4357), 5_8S_rRNA (-2938)
21	N/A	3		gene: ENSG00000278996 (-32435), MIR6724-1 (-54675), gene: ENSG00000275664 (-54141), MIR3648-1 (-51429), gene: ENSG00000277437 (-51177), gene: ENSG00000280800 (-48775), RNA5-8SN2 (-47357), gene: ENSG00000286155 (-24249), gene: ENSG00000286252 (-22102), gene: ENSG00000286057 (-20989), gene: ENSG00000286012 (-19034), MIR6724-2 (-10485), gene: ENSG00000277671 (-9932), gene: ENSG00000281383 (-4567), 5_8S_rRNA (-3148)

30927-007.R1 (paired) (Report)

Host chromosome	Unaligned ends virus	Broken reads host	Disrupted genes	Nearby genes
21	N/A	3		gene: ENSG00000278996 (-32438), MIR6724-1 (-54678), gene: ENSG00000275664 (-54144), MIR3648-1 (-51432), gene: ENSG00000277437 (-51180), gene: ENSG00000280800 (-48778), RNA5-8SN2 (-47360), gene: ENSG00000286155 (-24252), gene: ENSG00000286252 (-22105), gene: ENSG00000286057 (-20992), gene: ENSG00000286012 (-19037), MIR6724-2 (-10488), gene: ENSG00000277671 (-9935), gene: ENSG00000281383 (-4570), 5_8S_rRNA (-3151)
21	N/A	1		gene: ENSG00000278996 (-32898), MIR6724-1 (-55138), gene: ENSG00000275664 (-54604), MIR3648-1 (-51892), gene: ENSG00000277437 (-51640), gene: ENSG00000280800 (-49238), RNA5-8SN2 (-47820), gene: ENSG00000286155 (-24712), gene: ENSG00000286252 (-22565), gene: ENSG00000286057 (-21452), gene: ENSG00000286012 (-19497), MIR6724-2 (-10948), gene: ENSG00000277671 (-10395), gene: ENSG00000281383 (-5030), 5_8S_rRNA (-3611)

30927-007.R1 (paired) (Report)

Host chromosome	Unaligned ends virus	Broken reads host	Disrupted genes	Nearby genes
21	N/A	3	gene: ENSG00000280441, gene: ENSG00000280614	gene: ENSG00000280441, MIR6724-3 (-5608), gene: ENSG00000274868 (-5074), gene: ENSG00000280614, RNA5-8SN3 (+1545), gene: ENSG00000286267 (+23695), gene: ENSG00000286146 (+26613), gene: ENSG00000286054 (+27821), gene: ENSG00000286178 (+29698), MIR6724-4 (+38468), gene: ENSG00000278775 (+39023), gene: ENSG00000281181 (+43567), RNA5-8SN1 (+45761), gene: ENSG00000286149 (+68258), gene: ENSG00000286032 (+71192), gene: ENSG00000286091 (+72404), gene: ENSG00000286148 (+74258)

30927-007.R1 (paired) (Report)

Host chromosome	Unaligned ends virus	Broken reads host	Disrupted genes	Nearby genes
21	N/A	2	gene: ENSG00000280441, gene: ENSG00000280614	gene: ENSG00000280441, MIR6724-3 (-5667), gene: ENSG00000274868 (-5133), gene: ENSG00000280614, RNA5-8SN3 (+1486), gene: ENSG00000286267 (+23636), gene: ENSG00000286146 (+26554), gene: ENSG00000286054 (+27762), gene: ENSG00000286178 (+29639), MIR6724-4 (+38409), gene: ENSG00000278775 (+38964), gene: ENSG00000281181 (+43508), RNA5-8SN1 (+45702), gene: ENSG00000286149 (+68199), gene: ENSG00000286032 (+71133), gene: ENSG00000286091 (+72345), gene: ENSG00000286148 (+74199)

30927-007.R1 (paired) (Report)

Host chromosome	Unaligned ends virus	Broken reads host	Disrupted genes	Nearby genes
21	N/A	3	gene: ENSG00000280441	gene: ENSG00000280441, MIR6724-3 (-8567), gene: ENSG00000274868 (-8033), gene: ENSG00000280614 (-2679), RNA5-8SN3 (-1261), gene: ENSG00000286267 (+20736), gene: ENSG00000286146 (+23654), gene: ENSG00000286054 (+24862), gene: ENSG00000286178 (+26739), MIR6724-4 (+35509), gene: ENSG00000278775 (+36064), gene: ENSG00000281181 (+40608), RNA5-8SN1 (+42802), gene: ENSG00000286149 (+65299), gene: ENSG00000286032 (+68233), gene: ENSG00000286091 (+69445), gene: ENSG00000286148 (+71299)

30927-007.R1 (paired) (Report)

Host chromosome	Unaligned ends virus	Broken reads host	Disrupted genes	Nearby genes
21	N/A	8	gene: ENSG00000280441	gene: ENSG00000280441, MIR6724-3 (-9843), gene: ENSG00000274868 (-9309), gene: ENSG00000280614 (-3955), RNA5-8SN3 (-2537), gene: ENSG00000286267 (+19460), gene: ENSG00000286146 (+22378), gene: ENSG00000286054 (+23586), gene: ENSG00000286178 (+25463), MIR6724-4 (+34233), gene: ENSG00000278775 (+34788), gene: ENSG00000281181 (+39332), RNA5-8SN1 (+41526), gene: ENSG00000286149 (+64023), gene: ENSG00000286032 (+66957), gene: ENSG00000286091 (+68169), gene: ENSG00000286148 (+70023)

30927-007.R1 (paired) (Report)

Host chromosome	Unaligned ends virus	Broken reads host	Disrupted genes	Nearby genes
21	N/A	8	gene: ENSG00000280441	gene: ENSG00000280441, MIR6724-3 (-9845), gene: ENSG00000274868 (-9311), gene: ENSG00000280614 (-3957), RNA5-8SN3 (-2539), gene: ENSG00000286267 (+19458), gene: ENSG00000286146 (+22376), gene: ENSG00000286054 (+23584), gene: ENSG00000286178 (+25461), MIR6724-4 (+34231), gene: ENSG00000278775 (+34786), gene: ENSG00000281181 (+39330), RNA5-8SN1 (+41524), gene: ENSG00000286149 (+64021), gene: ENSG00000286032 (+66955), gene: ENSG00000286091 (+68167), gene: ENSG00000286148 (+70021)

30927-007.R1 (paired) (Report)

Host chromosome	Unaligned ends virus	Broken reads host	Disrupted genes	Nearby genes
21	N/A	7	gene: ENSG00000280441	gene: ENSG00000280441, MIR6724-3 (-9861), gene: ENSG00000274868 (-9327), gene: ENSG00000280614 (-3973), RNA5-8SN3 (-2555), gene: ENSG00000286267 (+19442), gene: ENSG00000286146 (+22360), gene: ENSG00000286054 (+23568), gene: ENSG00000286178 (+25445), MIR6724-4 (+34215), gene: ENSG00000278775 (+34770), gene: ENSG00000281181 (+39314), RNA5-8SN1 (+41508), gene: ENSG00000286149 (+64005), gene: ENSG00000286032 (+66939), gene: ENSG00000286091 (+68151), gene: ENSG00000286148 (+70005)

30927-007.R1 (paired) (Report)

Host chromosome	Unaligned ends virus	Broken reads host	Disrupted genes	Nearby genes
21	N/A	8	gene: ENSG00000280441	gene: ENSG00000280441, MIR6724-3 (-9899), gene: ENSG00000274868 (-9365), gene: ENSG00000280614 (-4011), RNA5-8SN3 (-2593), gene: ENSG00000286267 (+19404), gene: ENSG00000286146 (+22322), gene: ENSG00000286054 (+23530), gene: ENSG00000286178 (+25407), MIR6724-4 (+34177), gene: ENSG00000278775 (+34732), gene: ENSG00000281181 (+39276), RNA5-8SN1 (+41470), gene: ENSG00000286149 (+63967), gene: ENSG00000286032 (+66901), gene: ENSG00000286091 (+68113), gene: ENSG00000286148 (+69967)

30927-007.R1 (paired) (Report)

Host chromosome	Unaligned ends virus	Broken reads host	Disrupted genes	Nearby genes
21	3,064	7	gene: ENSG00000280441	gene: ENSG00000280441, MIR6724-3 (-9926), gene: ENSG00000274868 (-9392), gene: ENSG00000280614 (-4038), RNA5-8SN3 (-2620), gene: ENSG00000286267 (+19377), gene: ENSG00000286146 (+22295), gene: ENSG00000286054 (+23503), gene: ENSG00000286178 (+25380), MIR6724-4 (+34150), gene: ENSG00000278775 (+34705), gene: ENSG00000281181 (+39249), RNA5-8SN1 (+41443), gene: ENSG00000286149 (+63940), gene: ENSG00000286032 (+66874), gene: ENSG00000286091 (+68086), gene: ENSG00000286148 (+69940)

30927-007.R1 (paired) (Report)

Host chromosome	Unaligned ends virus	Broken reads host	Disrupted genes	Nearby genes
21	N/A	7	gene: ENSG00000280441	gene: ENSG00000280441, MIR6724-3 (-9927), gene: ENSG00000274868 (-9393), gene: ENSG00000280614 (-4039), RNA5-8SN3 (-2621), gene: ENSG00000286267 (+19376), gene: ENSG00000286146 (+22294), gene: ENSG00000286054 (+23502), gene: ENSG00000286178 (+25379), MIR6724-4 (+34149), gene: ENSG00000278775 (+34704), gene: ENSG00000281181 (+39248), RNA5-8SN1 (+41442), gene: ENSG00000286149 (+63939), gene: ENSG00000286032 (+66873), gene: ENSG00000286091 (+68085), gene: ENSG00000286148 (+69939)

30927-007.R1 (paired) (Report)

Host chromosome	Unaligned ends virus	Broken reads host	Disrupted genes	Nearby genes
21	N/A	7	gene: ENSG00000280441	gene: ENSG00000280441, MIR6724-3 (-9938), gene: ENSG00000274868 (-9404), gene: ENSG00000280614 (-4050), RNA5-8SN3 (-2632), gene: ENSG00000286267 (+19365), gene: ENSG00000286146 (+22283), gene: ENSG00000286054 (+23491), gene: ENSG00000286178 (+25368), MIR6724-4 (+34138), gene: ENSG00000278775 (+34693), gene: ENSG00000281181 (+39237), RNA5-8SN1 (+41431), gene: ENSG00000286149 (+63928), gene: ENSG00000286032 (+66862), gene: ENSG00000286091 (+68074), gene: ENSG00000286148 (+69928)

30927-007.R1 (paired) (Report)

Host chromosome	Unaligned ends virus	Broken reads host	Disrupted genes	Nearby genes
21	N/A	6	gene: ENSG00000280441	gene: ENSG00000280441, MIR6724-3 (-9982), gene: ENSG00000274868 (-9448), gene: ENSG00000280614 (-4094), RNA5-8SN3 (-2676), gene: ENSG00000286267 (+19321), gene: ENSG00000286146 (+22239), gene: ENSG00000286054 (+23447), gene: ENSG00000286178 (+25324), MIR6724-4 (+34094), gene: ENSG00000278775 (+34649), gene: ENSG00000281181 (+39193), RNA5-8SN1 (+41387), gene: ENSG00000286149 (+63884), gene: ENSG00000286032 (+66818), gene: ENSG00000286091 (+68030), gene: ENSG00000286148 (+69884)

30927-007.R1 (paired) (Report)

Host chromosome	Unaligned ends virus	Broken reads host	Disrupted genes	Nearby genes
21	N/A	6	gene: ENSG00000280441	gene: ENSG00000280441, MIR6724-3 (-9992), gene: ENSG00000274868 (-9458), gene: ENSG00000280614 (-4104), RNA5-8SN3 (-2686), gene: ENSG00000286267 (+19311), gene: ENSG00000286146 (+22229), gene: ENSG00000286054 (+23437), gene: ENSG00000286178 (+25314), MIR6724-4 (+34084), gene: ENSG00000278775 (+34639), gene: ENSG00000281181 (+39183), RNA5-8SN1 (+41377), gene: ENSG00000286149 (+63874), gene: ENSG00000286032 (+66808), gene: ENSG00000286091 (+68020), gene: ENSG00000286148 (+69874)

30927-007.R1 (paired) (Report)

Host chromosome	Unaligned ends virus	Broken reads host	Disrupted genes	Nearby genes
21	3,064	5	gene: ENSG00000280441	gene: ENSG00000280441, MIR6724-3 (-10011), gene: ENSG00000274868 (-9477), gene: ENSG00000280614 (-4123), RNA5-8SN3 (-2705), gene: ENSG00000286267 (+19292), gene: ENSG00000286146 (+22210), gene: ENSG00000286054 (+23418), gene: ENSG00000286178 (+25295), MIR6724-4 (+34065), gene: ENSG00000278775 (+34620), gene: ENSG00000281181 (+39164), RNA5-8SN1 (+41358), gene: ENSG00000286149 (+63855), gene: ENSG00000286032 (+66789), gene: ENSG00000286091 (+68001), gene: ENSG00000286148 (+69855)

30927-007.R1 (paired) (Report)

Host chromosome	Unaligned ends virus	Broken reads host	Disrupted genes	Nearby genes
21	N/A	2	gene: ENSG00000280441	gene: ENSG00000280441, MIR6724-3 (-10438), gene: ENSG00000274868 (-9904), gene: ENSG00000280614 (-4550), RNA5-8SN3 (-3132), gene: ENSG00000286267 (+18865), gene: ENSG00000286146 (+21783), gene: ENSG00000286054 (+22991), gene: ENSG00000286178 (+24868), MIR6724-4 (+33638), gene: ENSG00000278775 (+34193), gene: ENSG00000281181 (+38737), RNA5-8SN1 (+40931), gene: ENSG00000286149 (+63428), gene: ENSG00000286032 (+66362), gene: ENSG00000286091 (+67574), gene: ENSG00000286148 (+69428)

30927-007.R1 (paired) (Report)

Host chromosome	Unaligned ends virus	Broken reads host	Disrupted genes	Nearby genes
21	4,090	2	gene: ENSG00000280441	gene: ENSG00000280441, MIR6724-3 (-10441), gene: ENSG00000274868 (-9907), gene: ENSG00000280614 (-4553), RNA5-8SN3 (-3135), gene: ENSG00000286267 (+18862), gene: ENSG00000286146 (+21780), gene: ENSG00000286054 (+22988), gene: ENSG00000286178 (+24865), MIR6724-4 (+33635), gene: ENSG00000278775 (+34190), gene: ENSG00000281181 (+38734), RNA5-8SN1 (+40928), gene: ENSG00000286149 (+63425), gene: ENSG00000286032 (+66359), gene: ENSG00000286091 (+67571), gene: ENSG00000286148 (+69425)

30927-007.R1 (paired) (Report)

Host chromosome	Unaligned ends virus	Broken reads host	Disrupted genes	Nearby genes
21	N/A	2	gene: ENSG00000280441	gene: ENSG00000280441, MIR6724-3 (-13107), gene: ENSG00000274868 (-12573), gene: ENSG00000280614 (-7219), RNA5-8SN3 (-5801), gene: ENSG00000286267 (+16196), gene: ENSG00000286146 (+19114), gene: ENSG00000286054 (+20322), gene: ENSG00000286178 (+22199), MIR6724-4 (+30969), gene: ENSG00000278775 (+31524), gene: ENSG00000281181 (+36068), RNA5-8SN1 (+38262), gene: ENSG00000286149 (+60759), gene: ENSG00000286032 (+63693), gene: ENSG00000286091 (+64905), gene: ENSG00000286148 (+66759)

30927-007.R1 (paired) (Report)

Host chromosome	Unaligned ends virus	Broken reads host	Disrupted genes	Nearby genes
21	N/A	2	gene: ENSG00000280441	gene: ENSG00000280441, MIR6724-3 (-13116), gene: ENSG00000274868 (-12582), gene: ENSG00000280614 (-7228), RNA5-8SN3 (-5810), gene: ENSG00000286267 (+16187), gene: ENSG00000286146 (+19105), gene: ENSG00000286054 (+20313), gene: ENSG00000286178 (+22190), MIR6724-4 (+30960), gene: ENSG00000278775 (+31515), gene: ENSG00000281181 (+36059), RNA5-8SN1 (+38253), gene: ENSG00000286149 (+60750), gene: ENSG00000286032 (+63684), gene: ENSG00000286091 (+64896), gene: ENSG00000286148 (+66750)

30927-007.R1 (paired) (Report)

Host chromosome	Unaligned ends virus	Broken reads host	Disrupted genes	Nearby genes
21	4,090	2	gene: ENSG00000280441	gene: ENSG00000280441, MIR6724-3 (-13134), gene: ENSG00000274868 (-12600), gene: ENSG00000280614 (-7246), RNA5-8SN3 (-5828), gene: ENSG00000286267 (+16169), gene: ENSG00000286146 (+19087), gene: ENSG00000286054 (+20295), gene: ENSG00000286178 (+22172), MIR6724-4 (+30942), gene: ENSG00000278775 (+31497), gene: ENSG00000281181 (+36041), RNA5-8SN1 (+38235), gene: ENSG00000286149 (+60732), gene: ENSG00000286032 (+63666), gene: ENSG00000286091 (+64878), gene: ENSG00000286148 (+66732)

30927-007.R1 (paired) (Report)

Host chromosome	Unaligned ends virus	Broken reads host	Disrupted genes	Nearby genes
21	3,061	1	gene: ENSG00000280441	gene: ENSG00000280441, MIR6724-3 (-13181), gene: ENSG00000274868 (-12647), gene: ENSG00000280614 (-7293), RNA5-8SN3 (-5875), gene: ENSG00000286267 (+16122), gene: ENSG00000286146 (+19040), gene: ENSG00000286054 (+20248), gene: ENSG00000286178 (+22125), MIR6724-4 (+30895), gene: ENSG00000278775 (+31450), gene: ENSG00000281181 (+35994), RNA5-8SN1 (+38188), gene: ENSG00000286149 (+60685), gene: ENSG00000286032 (+63619), gene: ENSG00000286091 (+64831), gene: ENSG00000286148 (+66685)

30927-007.R1 (paired) (Report)

Host chromosome	Unaligned ends virus	Broken reads host	Disrupted genes	Nearby genes
21	41,362	1	gene: ENSG00000280441	gene: ENSG00000280441, MIR6724-3 (-13182), gene: ENSG00000274868 (-12648), gene: ENSG00000280614 (-7294), RNA5-8SN3 (-5876), gene: ENSG00000286267 (+16121), gene: ENSG00000286146 (+19039), gene: ENSG00000286054 (+20247), gene: ENSG00000286178 (+22124), MIR6724-4 (+30894), gene: ENSG00000278775 (+31449), gene: ENSG00000281181 (+35993), RNA5-8SN1 (+38187), gene: ENSG00000286149 (+60684), gene: ENSG00000286032 (+63618), gene: ENSG00000286091 (+64830), gene: ENSG00000286148 (+66684)

30927-007.R1 (paired) (Report)

Host chromosome	Unaligned ends virus	Broken reads host	Disrupted genes	Nearby genes
21	N/A	1	gene: ENSG00000280441	gene: ENSG00000280441, MIR6724-3 (-13192), gene: ENSG00000274868 (-12658), gene: ENSG00000280614 (-7304), RNA5-8SN3 (-5886), gene: ENSG00000286267 (+16111), gene: ENSG00000286146 (+19029), gene: ENSG00000286054 (+20237), gene: ENSG00000286178 (+22114), MIR6724-4 (+30884), gene: ENSG00000278775 (+31439), gene: ENSG00000281181 (+35983), RNA5-8SN1 (+38177), gene: ENSG00000286149 (+60674), gene: ENSG00000286032 (+63608), gene: ENSG00000286091 (+64820), gene: ENSG00000286148 (+66674)

30927-007.R1 (paired) (Report)

Host chromosome	Unaligned ends virus	Broken reads host	Disrupted genes	Nearby genes
21	428	1	gene: ENSG00000280441, gene: ENSG00000281181	gene: ENSG00000280441, MIR6724-3 (-49817), gene: ENSG00000274868 (-49283), gene: ENSG00000280614 (-43929), RNA5-8SN3 (-42511), gene: ENSG00000286267 (-20035), gene: ENSG00000286146 (-17267), gene: ENSG00000286054 (-16154), gene: ENSG00000286178 (-14199), MIR6724-4 (-5649), gene: ENSG00000278775 (-5096), gene: ENSG00000281181, RNA5-8SN1 (+1552), gene: ENSG00000286149 (+24049), gene: ENSG00000286032 (+26983), gene: ENSG00000286091 (+28195), gene: ENSG00000286148 (+30049)

30927-007.R1 (paired) (Report)

Host chromosome	Unaligned ends virus	Broken reads host	Disrupted genes	Nearby genes
21	N/A	3	gene: ENSG00000280441	gene: ENSG00000280441, MIR6724-3 (-52721), gene: ENSG00000274868 (-52187), gene: ENSG00000280614 (-46833), RNA5-8SN3 (-45415), gene: ENSG00000286267 (-22939), gene: ENSG00000286146 (-20171), gene: ENSG00000286054 (-19058), gene: ENSG00000286178 (-17103), MIR6724-4 (-8553), gene: ENSG00000278775 (-8000), gene: ENSG00000281181 (-2623), RNA5-8SN1 (-1199), gene: ENSG00000286149 (+21145), gene: ENSG00000286032 (+24079), gene: ENSG00000286091 (+25291), gene: ENSG00000286148 (+27145)

30927-007.R1 (paired) (Report)

Host chromosome	Unaligned ends virus	Broken reads host	Disrupted genes	Nearby genes
21	2,332	2	gene: ENSG00000280441	gene: ENSG00000280441, MIR6724-3 (-52740), gene: ENSG00000274868 (-52206), gene: ENSG00000280614 (-46852), RNA5-8SN3 (-45434), gene: ENSG00000286267 (-22958), gene: ENSG00000286146 (-20190), gene: ENSG00000286054 (-19077), gene: ENSG00000286178 (-17122), MIR6724-4 (-8572), gene: ENSG00000278775 (-8019), gene: ENSG00000281181 (-2642), RNA5-8SN1 (-1218), gene: ENSG00000286149 (+21126), gene: ENSG00000286032 (+24060), gene: ENSG00000286091 (+25272), gene: ENSG00000286148 (+27126)

30927-007.R1 (paired) (Report)

Host chromosome	Unaligned ends virus	Broken reads host	Disrupted genes	Nearby genes
21	N/A	2	gene: ENSG00000280441	gene: ENSG00000280441, MIR6724-3 (-52748), gene: ENSG00000274868 (-52214), gene: ENSG00000280614 (-46860), RNA5-8SN3 (-45442), gene: ENSG00000286267 (-22966), gene: ENSG00000286146 (-20198), gene: ENSG00000286054 (-19085), gene: ENSG00000286178 (-17130), MIR6724-4 (-8580), gene: ENSG00000278775 (-8027), gene: ENSG00000281181 (-2650), RNA5-8SN1 (-1226), gene: ENSG00000286149 (+21118), gene: ENSG00000286032 (+24052), gene: ENSG00000286091 (+25264), gene: ENSG00000286148 (+27118)

30927-007.R1 (paired) (Report)

Host chromosome	Unaligned ends virus	Broken reads host	Disrupted genes	Nearby genes
21	N/A	2	gene: ENSG00000280441	gene: ENSG00000280441, MIR6724-3 (-52781), gene: ENSG00000274868 (-52247), gene: ENSG00000280614 (-46893), RNA5-8SN3 (-45475), gene: ENSG00000286267 (-22999), gene: ENSG00000286146 (-20231), gene: ENSG00000286054 (-19118), gene: ENSG00000286178 (-17163), MIR6724-4 (-8613), gene: ENSG00000278775 (-8060), gene: ENSG00000281181 (-2683), RNA5-8SN1 (-1259), gene: ENSG00000286149 (+21085), gene: ENSG00000286032 (+24019), gene: ENSG00000286091 (+25231), gene: ENSG00000286148 (+27085)

30927-007.R1 (paired) (Report)

Host chromosome	Unaligned ends virus	Broken reads host	Disrupted genes	Nearby genes
21	N/A	2	gene: ENSG00000280441	gene: ENSG00000280441, MIR6724-3 (-53125), gene: ENSG00000274868 (-52591), gene: ENSG00000280614 (-47237), RNA5-8SN3 (-45819), gene: ENSG00000286267 (-23343), gene: ENSG00000286146 (-20575), gene: ENSG00000286054 (-19462), gene: ENSG00000286178 (-17507), MIR6724-4 (-8957), gene: ENSG00000278775 (-8404), gene: ENSG00000281181 (-3027), RNA5-8SN1 (-1603), gene: ENSG00000286149 (+20741), gene: ENSG00000286032 (+23675), gene: ENSG00000286091 (+24887), gene: ENSG00000286148 (+26741)

30927-007.R1 (paired) (Report)

Host chromosome	Unaligned ends virus	Broken reads host	Disrupted genes	Nearby genes
21	N/A	11	gene: ENSG00000280441	gene: ENSG00000280441, MIR6724-3 (-53668), gene: ENSG00000274868 (-53134), gene: ENSG00000280614 (-47780), RNA5-8SN3 (-46362), gene: ENSG00000286267 (-23886), gene: ENSG00000286146 (-21118), gene: ENSG00000286054 (-20005), gene: ENSG00000286178 (-18050), MIR6724-4 (-9500), gene: ENSG00000278775 (-8947), gene: ENSG00000281181 (-3570), RNA5-8SN1 (-2146), gene: ENSG00000286149 (+20198), gene: ENSG00000286032 (+23132), gene: ENSG00000286091 (+24344), gene: ENSG00000286148 (+26198)

30927-007.R1 (paired) (Report)

Host chromosome	Unaligned ends virus	Broken reads host	Disrupted genes	Nearby genes
21	N/A	11	gene: ENSG00000280441	gene: ENSG00000280441, MIR6724-3 (-53726), gene: ENSG00000274868 (-53192), gene: ENSG00000280614 (-47838), RNA5-8SN3 (-46420), gene: ENSG00000286267 (-23944), gene: ENSG00000286146 (-21176), gene: ENSG00000286054 (-20063), gene: ENSG00000286178 (-18108), MIR6724-4 (-9558), gene: ENSG00000278775 (-9005), gene: ENSG00000281181 (-3628), RNA5-8SN1 (-2204), gene: ENSG00000286149 (+20140), gene: ENSG00000286032 (+23074), gene: ENSG00000286091 (+24286), gene: ENSG00000286148 (+26140)

30927-007.R1 (paired) (Report)

Host chromosome	Unaligned ends virus	Broken reads host	Disrupted genes	Nearby genes
21	N/A	11	gene: ENSG00000280441	gene: ENSG00000280441, MIR6724-3 (-53756), gene: ENSG00000274868 (-53222), gene: ENSG00000280614 (-47868), RNA5-8SN3 (-46450), gene: ENSG00000286267 (-23974), gene: ENSG00000286146 (-21206), gene: ENSG00000286054 (-20093), gene: ENSG00000286178 (-18138), MIR6724-4 (-9588), gene: ENSG00000278775 (-9035), gene: ENSG00000281181 (-3658), RNA5-8SN1 (-2234), gene: ENSG00000286149 (+20110), gene: ENSG00000286032 (+23044), gene: ENSG00000286091 (+24256), gene: ENSG00000286148 (+26110)

30927-007.R1 (paired) (Report)

Host chromosome	Unaligned ends virus	Broken reads host	Disrupted genes	Nearby genes
21	N/A	10	gene: ENSG00000280441	gene: ENSG00000280441, MIR6724-3 (-53764), gene: ENSG00000274868 (-53230), gene: ENSG00000280614 (-47876), RNA5-8SN3 (-46458), gene: ENSG00000286267 (-23982), gene: ENSG00000286146 (-21214), gene: ENSG00000286054 (-20101), gene: ENSG00000286178 (-18146), MIR6724-4 (-9596), gene: ENSG00000278775 (-9043), gene: ENSG00000281181 (-3666), RNA5-8SN1 (-2242), gene: ENSG00000286149 (+20102), gene: ENSG00000286032 (+23036), gene: ENSG00000286091 (+24248), gene: ENSG00000286148 (+26102)

30927-007.R1 (paired) (Report)

Host chromosome	Unaligned ends virus	Broken reads host	Disrupted genes	Nearby genes
21	N/A	10	gene: ENSG00000280441	gene: ENSG00000280441, MIR6724-3 (-53801), gene: ENSG00000274868 (-53267), gene: ENSG00000280614 (-47913), RNA5-8SN3 (-46495), gene: ENSG00000286267 (-24019), gene: ENSG00000286146 (-21251), gene: ENSG00000286054 (-20138), gene: ENSG00000286178 (-18183), MIR6724-4 (-9633), gene: ENSG00000278775 (-9080), gene: ENSG00000281181 (-3703), RNA5-8SN1 (-2279), gene: ENSG00000286149 (+20065), gene: ENSG00000286032 (+22999), gene: ENSG00000286091 (+24211), gene: ENSG00000286148 (+26065)

30927-007.R1 (paired) (Report)

Host chromosome	Unaligned ends virus	Broken reads host	Disrupted genes	Nearby genes
21	N/A	10	gene: ENSG00000280441	gene: ENSG00000280441, MIR6724-3 (-53804), gene: ENSG00000274868 (-53270), gene: ENSG00000280614 (-47916), RNA5-8SN3 (-46498), gene: ENSG00000286267 (-24022), gene: ENSG00000286146 (-21254), gene: ENSG00000286054 (-20141), gene: ENSG00000286178 (-18186), MIR6724-4 (-9636), gene: ENSG00000278775 (-9083), gene: ENSG00000281181 (-3706), RNA5-8SN1 (-2282), gene: ENSG00000286149 (+20062), gene: ENSG00000286032 (+22996), gene: ENSG00000286091 (+24208), gene: ENSG00000286148 (+26062)

30927-007.R1 (paired) (Report)

Host chromosome	Unaligned ends virus	Broken reads host	Disrupted genes	Nearby genes
21	N/A	10	gene: ENSG00000280441	gene: ENSG00000280441, MIR6724-3 (-53807), gene: ENSG00000274868 (-53273), gene: ENSG00000280614 (-47919), RNA5-8SN3 (-46501), gene: ENSG00000286267 (-24025), gene: ENSG00000286146 (-21257), gene: ENSG00000286054 (-20144), gene: ENSG00000286178 (-18189), MIR6724-4 (-9639), gene: ENSG00000278775 (-9086), gene: ENSG00000281181 (-3709), RNA5-8SN1 (-2285), gene: ENSG00000286149 (+20059), gene: ENSG00000286032 (+22993), gene: ENSG00000286091 (+24205), gene: ENSG00000286148 (+26059)

30927-007.R1 (paired) (Report)

Host chromosome	Unaligned ends virus	Broken reads host	Disrupted genes	Nearby genes
21	N/A	10	gene: ENSG00000280441	gene: ENSG00000280441, MIR6724-3 (-53815), gene: ENSG00000274868 (-53281), gene: ENSG00000280614 (-47927), RNA5-8SN3 (-46509), gene: ENSG00000286267 (-24033), gene: ENSG00000286146 (-21265), gene: ENSG00000286054 (-20152), gene: ENSG00000286178 (-18197), MIR6724-4 (-9647), gene: ENSG00000278775 (-9094), gene: ENSG00000281181 (-3717), RNA5-8SN1 (-2293), gene: ENSG00000286149 (+20051), gene: ENSG00000286032 (+22985), gene: ENSG00000286091 (+24197), gene: ENSG00000286148 (+26051)

30927-007.R1 (paired) (Report)

Host chromosome	Unaligned ends virus	Broken reads host	Disrupted genes	Nearby genes
21	N/A	10	gene: ENSG00000280441	gene: ENSG00000280441, MIR6724-3 (-53839), gene: ENSG00000274868 (-53305), gene: ENSG00000280614 (-47951), RNA5-8SN3 (-46533), gene: ENSG00000286267 (-24057), gene: ENSG00000286146 (-21289), gene: ENSG00000286054 (-20176), gene: ENSG00000286178 (-18221), MIR6724-4 (-9671), gene: ENSG00000278775 (-9118), gene: ENSG00000281181 (-3741), RNA5-8SN1 (-2317), gene: ENSG00000286149 (+20027), gene: ENSG00000286032 (+22961), gene: ENSG00000286091 (+24173), gene: ENSG00000286148 (+26027)

30927-007.R1 (paired) (Report)

Host chromosome	Unaligned ends virus	Broken reads host	Disrupted genes	Nearby genes
21	N/A	10	gene: ENSG00000280441	gene: ENSG00000280441, MIR6724-3 (-53847), gene: ENSG00000274868 (-53313), gene: ENSG00000280614 (-47959), RNA5-8SN3 (-46541), gene: ENSG00000286267 (-24065), gene: ENSG00000286146 (-21297), gene: ENSG00000286054 (-20184), gene: ENSG00000286178 (-18229), MIR6724-4 (-9679), gene: ENSG00000278775 (-9126), gene: ENSG00000281181 (-3749), RNA5-8SN1 (-2325), gene: ENSG00000286149 (+20019), gene: ENSG00000286032 (+22953), gene: ENSG00000286091 (+24165), gene: ENSG00000286148 (+26019)

30927-007.R1 (paired) (Report)

Host chromosome	Unaligned ends virus	Broken reads host	Disrupted genes	Nearby genes
21	3,206	10	gene: ENSG00000280441	gene: ENSG00000280441, MIR6724-3 (-53848), gene: ENSG00000274868 (-53314), gene: ENSG00000280614 (-47960), RNA5-8SN3 (-46542), gene: ENSG00000286267 (-24066), gene: ENSG00000286146 (-21298), gene: ENSG00000286054 (-20185), gene: ENSG00000286178 (-18230), MIR6724-4 (-9680), gene: ENSG00000278775 (-9127), gene: ENSG00000281181 (-3750), RNA5-8SN1 (-2326), gene: ENSG00000286149 (+20018), gene: ENSG00000286032 (+22952), gene: ENSG00000286091 (+24164), gene: ENSG00000286148 (+26018)

30927-007.R1 (paired) (Report)

Host chromosome	Unaligned ends virus	Broken reads host	Disrupted genes	Nearby genes
21	N/A	10	gene: ENSG00000280441	gene: ENSG00000280441, MIR6724-3 (-53852), gene: ENSG00000274868 (-53318), gene: ENSG00000280614 (-47964), RNA5-8SN3 (-46546), gene: ENSG00000286267 (-24070), gene: ENSG00000286146 (-21302), gene: ENSG00000286054 (-20189), gene: ENSG00000286178 (-18234), MIR6724-4 (-9684), gene: ENSG00000278775 (-9131), gene: ENSG00000281181 (-3754), RNA5-8SN1 (-2330), gene: ENSG00000286149 (+20014), gene: ENSG00000286032 (+22948), gene: ENSG00000286091 (+24160), gene: ENSG00000286148 (+26014)

30927-007.R1 (paired) (Report)

Host chromosome	Unaligned ends virus	Broken reads host	Disrupted genes	Nearby genes
21	N/A	10	gene: ENSG00000280441	gene: ENSG00000280441, MIR6724-3 (-53853), gene: ENSG00000274868 (-53319), gene: ENSG00000280614 (-47965), RNA5-8SN3 (-46547), gene: ENSG00000286267 (-24071), gene: ENSG00000286146 (-21303), gene: ENSG00000286054 (-20190), gene: ENSG00000286178 (-18235), MIR6724-4 (-9685), gene: ENSG00000278775 (-9132), gene: ENSG00000281181 (-3755), RNA5-8SN1 (-2331), gene: ENSG00000286149 (+20013), gene: ENSG00000286032 (+22947), gene: ENSG00000286091 (+24159), gene: ENSG00000286148 (+26013)

30927-007.R1 (paired) (Report)

Host chromosome	Unaligned ends virus	Broken reads host	Disrupted genes	Nearby genes
21	N/A	10	gene: ENSG00000280441	gene: ENSG00000280441, MIR6724-3 (-53854), gene: ENSG00000274868 (-53320), gene: ENSG00000280614 (-47966), RNA5-8SN3 (-46548), gene: ENSG00000286267 (-24072), gene: ENSG00000286146 (-21304), gene: ENSG00000286054 (-20191), gene: ENSG00000286178 (-18236), MIR6724-4 (-9686), gene: ENSG00000278775 (-9133), gene: ENSG00000281181 (-3756), RNA5-8SN1 (-2332), gene: ENSG00000286149 (+20012), gene: ENSG00000286032 (+22946), gene: ENSG00000286091 (+24158), gene: ENSG00000286148 (+26012)

30927-007.R1 (paired) (Report)

Host chromosome	Unaligned ends virus	Broken reads host	Disrupted genes	Nearby genes
21	N/A	10	gene: ENSG00000280441	gene: ENSG00000280441, MIR6724-3 (-53859), gene: ENSG00000274868 (-53325), gene: ENSG00000280614 (-47971), RNA5-8SN3 (-46553), gene: ENSG00000286267 (-24077), gene: ENSG00000286146 (-21309), gene: ENSG00000286054 (-20196), gene: ENSG00000286178 (-18241), MIR6724-4 (-9691), gene: ENSG00000278775 (-9138), gene: ENSG00000281181 (-3761), RNA5-8SN1 (-2337), gene: ENSG00000286149 (+20007), gene: ENSG00000286032 (+22941), gene: ENSG00000286091 (+24153), gene: ENSG00000286148 (+26007)

Table S4 Viral integration sites reports

30927-007.R1 (paired) (Report)

Host chromosome	Unaligned ends virus	Broken reads host	Disrupted genes	Nearby genes
21	N/A	10	gene: ENSG00000280441	gene: ENSG00000280441, MIR6724-3 (-53860), gene: ENSG00000274868 (-53326), gene: ENSG00000280614 (-47972), RNA5-8SN3 (-46554), gene: ENSG00000286267 (-24078), gene: ENSG00000286146 (-21310), gene: ENSG00000286054 (-20197), gene: ENSG00000286178 (-18242), MIR6724-4 (-9692), gene: ENSG00000278775 (-9139), gene: ENSG00000281181 (-3762), RNA5-8SN1 (-2338), gene: ENSG00000286149 (+20006), gene: ENSG00000286032 (+22940), gene: ENSG00000286091 (+24152), gene: ENSG00000286148 (+26006)

30927-007.R1 (paired) (Report)

Host chromosome	Unaligned ends virus	Broken reads host	Disrupted genes	Nearby genes
21	41,362	8	gene: ENSG00000280441	gene: ENSG00000280441, MIR6724-3 (-53864), gene: ENSG00000274868 (-53330), gene: ENSG00000280614 (-47976), RNA5-8SN3 (-46558), gene: ENSG00000286267 (-24082), gene: ENSG00000286146 (-21314), gene: ENSG00000286054 (-20201), gene: ENSG00000286178 (-18246), MIR6724-4 (-9696), gene: ENSG00000278775 (-9143), gene: ENSG00000281181 (-3766), RNA5-8SN1 (-2342), gene: ENSG00000286149 (+20002), gene: ENSG00000286032 (+22936), gene: ENSG00000286091 (+24148), gene: ENSG00000286148 (+26002)

30927-007.R1 (paired) (Report)

Host chromosome	Unaligned ends virus	Broken reads host	Disrupted genes	Nearby genes
21	3,418	8	gene: ENSG00000280441	gene: ENSG00000280441, MIR6724-3 (-53867), gene: ENSG00000274868 (-53333), gene: ENSG00000280614 (-47979), RNA5-8SN3 (-46561), gene: ENSG00000286267 (-24085), gene: ENSG00000286146 (-21317), gene: ENSG00000286054 (-20204), gene: ENSG00000286178 (-18249), MIR6724-4 (-9699), gene: ENSG00000278775 (-9146), gene: ENSG00000281181 (-3769), RNA5-8SN1 (-2345), gene: ENSG00000286149 (+19999), gene: ENSG00000286032 (+22933), gene: ENSG00000286091 (+24145), gene: ENSG00000286148 (+25999)

30927-007.R1 (paired) (Report)

Host chromosome	Unaligned ends virus	Broken reads host	Disrupted genes	Nearby genes
21	2,332	7	gene: ENSG00000280441	gene: ENSG00000280441, MIR6724-3 (-53870), gene: ENSG00000274868 (-53336), gene: ENSG00000280614 (-47982), RNA5-8SN3 (-46564), gene: ENSG00000286267 (-24088), gene: ENSG00000286146 (-21320), gene: ENSG00000286054 (-20207), gene: ENSG00000286178 (-18252), MIR6724-4 (-9702), gene: ENSG00000278775 (-9149), gene: ENSG00000281181 (-3772), RNA5-8SN1 (-2348), gene: ENSG00000286149 (+19996), gene: ENSG00000286032 (+22930), gene: ENSG00000286091 (+24142), gene: ENSG00000286148 (+25996)

30927-007.R1 (paired) (Report)

Host chromosome	Unaligned ends virus	Broken reads host	Disrupted genes	Nearby genes
21	2,332	7	gene: ENSG00000280441	gene: ENSG00000280441, MIR6724-3 (-53871), gene: ENSG00000274868 (-53337), gene: ENSG00000280614 (-47983), RNA5-8SN3 (-46565), gene: ENSG00000286267 (-24089), gene: ENSG00000286146 (-21321), gene: ENSG00000286054 (-20208), gene: ENSG00000286178 (-18253), MIR6724-4 (-9703), gene: ENSG00000278775 (-9150), gene: ENSG00000281181 (-3773), RNA5-8SN1 (-2349), gene: ENSG00000286149 (+19995), gene: ENSG00000286032 (+22929), gene: ENSG00000286091 (+24141), gene: ENSG00000286148 (+25995)

30927-007.R1 (paired) (Report)

Host chromosome	Unaligned ends virus	Broken reads host	Disrupted genes	Nearby genes
21	N/A	7	gene: ENSG00000280441	gene: ENSG00000280441, MIR6724-3 (-53881), gene: ENSG00000274868 (-53347), gene: ENSG00000280614 (-47993), RNA5-8SN3 (-46575), gene: ENSG00000286267 (-24099), gene: ENSG00000286146 (-21331), gene: ENSG00000286054 (-20218), gene: ENSG00000286178 (-18263), MIR6724-4 (-9713), gene: ENSG00000278775 (-9160), gene: ENSG00000281181 (-3783), RNA5-8SN1 (-2359), gene: ENSG00000286149 (+19985), gene: ENSG00000286032 (+22919), gene: ENSG00000286091 (+24131), gene: ENSG00000286148 (+25985)

30927-007.R1 (paired) (Report)

Host chromosome	Unaligned ends virus	Broken reads host	Disrupted genes	Nearby genes
21	N/A	8	gene: ENSG00000280441	gene: ENSG00000280441, MIR6724-3 (-53899), gene: ENSG00000274868 (-53365), gene: ENSG00000280614 (-48011), RNA5-8SN3 (-46593), gene: ENSG00000286267 (-24117), gene: ENSG00000286146 (-21349), gene: ENSG00000286054 (-20236), gene: ENSG00000286178 (-18281), MIR6724-4 (-9731), gene: ENSG00000278775 (-9178), gene: ENSG00000281181 (-3801), RNA5-8SN1 (-2377), gene: ENSG00000286149 (+19967), gene: ENSG00000286032 (+22901), gene: ENSG00000286091 (+24113), gene: ENSG00000286148 (+25967)

30927-007.R1 (paired) (Report)

Host chromosome	Unaligned ends virus	Broken reads host	Disrupted genes	Nearby genes
21	N/A	8	gene: ENSG00000280441	gene: ENSG00000280441, MIR6724-3 (-53903), gene: ENSG00000274868 (-53369), gene: ENSG00000280614 (-48015), RNA5-8SN3 (-46597), gene: ENSG00000286267 (-24121), gene: ENSG00000286146 (-21353), gene: ENSG00000286054 (-20240), gene: ENSG00000286178 (-18285), MIR6724-4 (-9735), gene: ENSG00000278775 (-9182), gene: ENSG00000281181 (-3805), RNA5-8SN1 (-2381), gene: ENSG00000286149 (+19963), gene: ENSG00000286032 (+22897), gene: ENSG00000286091 (+24109), gene: ENSG00000286148 (+25963)

30927-007.R1 (paired) (Report)

Host chromosome	Unaligned ends virus	Broken reads host	Disrupted genes	Nearby genes
21	N/A	8	gene: ENSG00000280441	gene: ENSG00000280441, MIR6724-3 (-54018), gene: ENSG00000274868 (-53484), gene: ENSG00000280614 (-48130), RNA5-8SN3 (-46712), gene: ENSG00000286267 (-24236), gene: ENSG00000286146 (-21468), gene: ENSG00000286054 (-20355), gene: ENSG00000286178 (-18400), MIR6724-4 (-9850), gene: ENSG00000278775 (-9297), gene: ENSG00000281181 (-3920), RNA5-8SN1 (-2496), gene: ENSG00000286149 (+19848), gene: ENSG00000286032 (+22782), gene: ENSG00000286091 (+23994), gene: ENSG00000286148 (+25848)

30927-007.R1 (paired) (Report)

Host chromosome	Unaligned ends virus	Broken reads host	Disrupted genes	Nearby genes
21	N/A	8	gene: ENSG00000280441	gene: ENSG00000280441, MIR6724-3 (-54022), gene: ENSG00000274868 (-53488), gene: ENSG00000280614 (-48134), RNA5-8SN3 (-46716), gene: ENSG00000286267 (-24240), gene: ENSG00000286146 (-21472), gene: ENSG00000286054 (-20359), gene: ENSG00000286178 (-18404), MIR6724-4 (-9854), gene: ENSG00000278775 (-9301), gene: ENSG00000281181 (-3924), RNA5-8SN1 (-2500), gene: ENSG00000286149 (+19844), gene: ENSG00000286032 (+22778), gene: ENSG00000286091 (+23990), gene: ENSG00000286148 (+25844)

30927-007.R1 (paired) (Report)

Host chromosome	Unaligned ends virus	Broken reads host	Disrupted genes	Nearby genes
21	N/A	7	gene: ENSG00000280441	gene: ENSG00000280441, MIR6724-3 (-54047), gene: ENSG00000274868 (-53513), gene: ENSG00000280614 (-48159), RNA5-8SN3 (-46741), gene: ENSG00000286267 (-24265), gene: ENSG00000286146 (-21497), gene: ENSG00000286054 (-20384), gene: ENSG00000286178 (-18429), MIR6724-4 (-9879), gene: ENSG00000278775 (-9326), gene: ENSG00000281181 (-3949), RNA5-8SN1 (-2525), gene: ENSG00000286149 (+19819), gene: ENSG00000286032 (+22753), gene: ENSG00000286091 (+23965), gene: ENSG00000286148 (+25819)

30927-007.R1 (paired) (Report)

Host chromosome	Unaligned ends virus	Broken reads host	Disrupted genes	Nearby genes
21	N/A	7	gene: ENSG00000280441	gene: ENSG00000280441, MIR6724-3 (-54086), gene: ENSG00000274868 (-53552), gene: ENSG00000280614 (-48198), RNA5-8SN3 (-46780), gene: ENSG00000286267 (-24304), gene: ENSG00000286146 (-21536), gene: ENSG00000286054 (-20423), gene: ENSG00000286178 (-18468), MIR6724-4 (-9918), gene: ENSG00000278775 (-9365), gene: ENSG00000281181 (-3988), RNA5-8SN1 (-2564), gene: ENSG00000286149 (+19780), gene: ENSG00000286032 (+22714), gene: ENSG00000286091 (+23926), gene: ENSG00000286148 (+25780)

30927-007.R1 (paired) (Report)

Host chromosome	Unaligned ends virus	Broken reads host	Disrupted genes	Nearby genes
21	N/A	7	gene: ENSG00000280441	gene: ENSG00000280441, MIR6724-3 (-54137), gene: ENSG00000274868 (-53603), gene: ENSG00000280614 (-48249), RNA5-8SN3 (-46831), gene: ENSG00000286267 (-24355), gene: ENSG00000286146 (-21587), gene: ENSG00000286054 (-20474), gene: ENSG00000286178 (-18519), MIR6724-4 (-9969), gene: ENSG00000278775 (-9416), gene: ENSG00000281181 (-4039), RNA5-8SN1 (-2615), gene: ENSG00000286149 (+19729), gene: ENSG00000286032 (+22663), gene: ENSG00000286091 (+23875), gene: ENSG00000286148 (+25729)

30927-007.R1 (paired) (Report)

Host chromosome	Unaligned ends virus	Broken reads host	Disrupted genes	Nearby genes
21	N/A	7	gene: ENSG00000280441	gene: ENSG00000280441, MIR6724-3 (-54145), gene: ENSG00000274868 (-53611), gene: ENSG00000280614 (-48257), RNA5-8SN3 (-46839), gene: ENSG00000286267 (-24363), gene: ENSG00000286146 (-21595), gene: ENSG00000286054 (-20482), gene: ENSG00000286178 (-18527), MIR6724-4 (-9977), gene: ENSG00000278775 (-9424), gene: ENSG00000281181 (-4047), RNA5-8SN1 (-2623), gene: ENSG00000286149 (+19721), gene: ENSG00000286032 (+22655), gene: ENSG00000286091 (+23867), gene: ENSG00000286148 (+25721)

30927-007.R1 (paired) (Report)

Host chromosome	Unaligned ends virus	Broken reads host	Disrupted genes	Nearby genes
21	N/A	5	gene: ENSG00000280441	gene: ENSG00000280441, MIR6724-3 (-54190), gene: ENSG00000274868 (-53656), gene: ENSG00000280614 (-48302), RNA5-8SN3 (-46884), gene: ENSG00000286267 (-24408), gene: ENSG00000286146 (-21640), gene: ENSG00000286054 (-20527), gene: ENSG00000286178 (-18572), MIR6724-4 (-10022), gene: ENSG00000278775 (-9469), gene: ENSG00000281181 (-4092), RNA5-8SN1 (-2668), gene: ENSG00000286149 (+19676), gene: ENSG00000286032 (+22610), gene: ENSG00000286091 (+23822), gene: ENSG00000286148 (+25676)

30927-007.R1 (paired) (Report)

Host chromosome	Unaligned ends virus	Broken reads host	Disrupted genes	Nearby genes
21	N/A	4	gene: ENSG00000280441	gene: ENSG00000280441, MIR6724-3 (-54210), gene: ENSG00000274868 (-53676), gene: ENSG00000280614 (-48322), RNA5-8SN3 (-46904), gene: ENSG00000286267 (-24428), gene: ENSG00000286146 (-21660), gene: ENSG00000286054 (-20547), gene: ENSG00000286178 (-18592), MIR6724-4 (-10042), gene: ENSG00000278775 (-9489), gene: ENSG00000281181 (-4112), RNA5-8SN1 (-2688), gene: ENSG00000286149 (+19656), gene: ENSG00000286032 (+22590), gene: ENSG00000286091 (+23802), gene: ENSG00000286148 (+25656)

30927-007.R1 (paired) (Report)

Host chromosome	Unaligned ends virus	Broken reads host	Disrupted genes	Nearby genes
21	N/A	3	gene: ENSG00000280441	gene: ENSG00000280441, MIR6724-3 (-54257), gene: ENSG00000274868 (-53723), gene: ENSG00000280614 (-48369), RNA5-8SN3 (-46951), gene: ENSG00000286267 (-24475), gene: ENSG00000286146 (-21707), gene: ENSG00000286054 (-20594), gene: ENSG00000286178 (-18639), MIR6724-4 (-10089), gene: ENSG00000278775 (-9536), gene: ENSG00000281181 (-4159), RNA5-8SN1 (-2735), gene: ENSG00000286149 (+19609), gene: ENSG00000286032 (+22543), gene: ENSG00000286091 (+23755), gene: ENSG00000286148 (+25609)

30927-007.R1 (paired) (Report)

Host chromosome	Unaligned ends virus	Broken reads host	Disrupted genes	Nearby genes
21	N/A	6	gene: ENSG00000280441	gene: ENSG00000280441, MIR6724-3 (-54451), gene: ENSG00000274868 (-53917), gene: ENSG00000280614 (-48563), RNA5-8SN3 (-47145), gene: ENSG00000286267 (-24669), gene: ENSG00000286146 (-21901), gene: ENSG00000286054 (-20788), gene: ENSG00000286178 (-18833), MIR6724-4 (-10283), gene: ENSG00000278775 (-9730), gene: ENSG00000281181 (-4353), RNA5-8SN1 (-2929), gene: ENSG00000286149 (+19415), gene: ENSG00000286032 (+22349), gene: ENSG00000286091 (+23561), gene: ENSG00000286148 (+25415)

30927-007.R1 (paired) (Report)

Host chromosome	Unaligned ends virus	Broken reads host	Disrupted genes	Nearby genes
21	N/A	6	gene: ENSG00000280441	gene: ENSG00000280441, MIR6724-3 (-54452), gene: ENSG00000274868 (-53918), gene: ENSG00000280614 (-48564), RNA5-8SN3 (-47146), gene: ENSG00000286267 (-24670), gene: ENSG00000286146 (-21902), gene: ENSG00000286054 (-20789), gene: ENSG00000286178 (-18834), MIR6724-4 (-10284), gene: ENSG00000278775 (-9731), gene: ENSG00000281181 (-4354), RNA5-8SN1 (-2930), gene: ENSG00000286149 (+19414), gene: ENSG00000286032 (+22348), gene: ENSG00000286091 (+23560), gene: ENSG00000286148 (+25414)

30927-007.R1 (paired) (Report)

Host chromosome	Unaligned ends virus	Broken reads host	Disrupted genes	Nearby genes
21	5,425	5	gene: ENSG00000280441	gene: ENSG00000280441, MIR6724-3 (-54497), gene: ENSG00000274868 (-53963), gene: ENSG00000280614 (-48609), RNA5-8SN3 (-47191), gene: ENSG00000286267 (-24715), gene: ENSG00000286146 (-21947), gene: ENSG00000286054 (-20834), gene: ENSG00000286178 (-18879), MIR6724-4 (-10329), gene: ENSG00000278775 (-9776), gene: ENSG00000281181 (-4399), RNA5-8SN1 (-2975), gene: ENSG00000286149 (+19369), gene: ENSG00000286032 (+22303), gene: ENSG00000286091 (+23515), gene: ENSG00000286148 (+25369)

30927-007.R1 (paired) (Report)

Host chromosome	Unaligned ends virus	Broken reads host	Disrupted genes	Nearby genes
21	N/A	5	gene: ENSG00000280441	gene: ENSG00000280441, MIR6724-3 (-54498), gene: ENSG00000274868 (-53964), gene: ENSG00000280614 (-48610), RNA5-8SN3 (-47192), gene: ENSG00000286267 (-24716), gene: ENSG00000286146 (-21948), gene: ENSG00000286054 (-20835), gene: ENSG00000286178 (-18880), MIR6724-4 (-10330), gene: ENSG00000278775 (-9777), gene: ENSG00000281181 (-4400), RNA5-8SN1 (-2976), gene: ENSG00000286149 (+19368), gene: ENSG00000286032 (+22302), gene: ENSG00000286091 (+23514), gene: ENSG00000286148 (+25368)

30927-007.R1 (paired) (Report)

Host chromosome	Unaligned ends virus	Broken reads host	Disrupted genes	Nearby genes
21	3,418	5	gene: ENSG00000280441	gene: ENSG00000280441, MIR6724-3 (-54515), gene: ENSG00000274868 (-53981), gene: ENSG00000280614 (-48627), RNA5-8SN3 (-47209), gene: ENSG00000286267 (-24733), gene: ENSG00000286146 (-21965), gene: ENSG00000286054 (-20852), gene: ENSG00000286178 (-18897), MIR6724-4 (-10347), gene: ENSG00000278775 (-9794), gene: ENSG00000281181 (-4417), RNA5-8SN1 (-2993), gene: ENSG00000286149 (+19351), gene: ENSG00000286032 (+22285), gene: ENSG00000286091 (+23497), gene: ENSG00000286148 (+25351)

30927-007.R1 (paired) (Report)

Host chromosome	Unaligned ends virus	Broken reads host	Disrupted genes	Nearby genes
21	N/A	4	gene: ENSG00000280441	gene: ENSG00000280441, MIR6724-3 (-54669), gene: ENSG00000274868 (-54135), gene: ENSG00000280614 (-48781), RNA5-8SN3 (-47363), gene: ENSG00000286267 (-24887), gene: ENSG00000286146 (-22119), gene: ENSG00000286054 (-21006), gene: ENSG00000286178 (-19051), MIR6724-4 (-10501), gene: ENSG00000278775 (-9948), gene: ENSG00000281181 (-4571), RNA5-8SN1 (-3147), gene: ENSG00000286149 (+19197), gene: ENSG00000286032 (+22131), gene: ENSG00000286091 (+23343), gene: ENSG00000286148 (+25197)

30927-007.R1 (paired) (Report)

Host chromosome	Unaligned ends virus	Broken reads host	Disrupted genes	Nearby genes
21	12,955	4	gene: ENSG00000280441	gene: ENSG00000280441, MIR6724-3 (-54670), gene: ENSG00000274868 (-54136), gene: ENSG00000280614 (-48782), RNA5-8SN3 (-47364), gene: ENSG00000286267 (-24888), gene: ENSG00000286146 (-22120), gene: ENSG00000286054 (-21007), gene: ENSG00000286178 (-19052), MIR6724-4 (-10502), gene: ENSG00000278775 (-9949), gene: ENSG00000281181 (-4572), RNA5-8SN1 (-3148), gene: ENSG00000286149 (+19196), gene: ENSG00000286032 (+22130), gene: ENSG00000286091 (+23342), gene: ENSG00000286148 (+25196)

30927-007.R1 (paired) (Report)

Host chromosome	Unaligned ends virus	Broken reads host	Disrupted genes	Nearby genes
21	N/A	3	gene: ENSG00000280441	gene: ENSG00000280441, MIR6724-3 (-54676), gene: ENSG00000274868 (-54142), gene: ENSG00000280614 (-48788), RNA5-8SN3 (-47370), gene: ENSG00000286267 (-24894), gene: ENSG00000286146 (-22126), gene: ENSG00000286054 (-21013), gene: ENSG00000286178 (-19058), MIR6724-4 (-10508), gene: ENSG00000278775 (-9955), gene: ENSG00000281181 (-4578), RNA5-8SN1 (-3154), gene: ENSG00000286149 (+19190), gene: ENSG00000286032 (+22124), gene: ENSG00000286091 (+23336), gene: ENSG00000286148 (+25190)

Table S4 Viral integration sites reports

30927-007.R1 (paired) (Report)

Host chromosome	Unaligned ends virus	Broken reads host	Disrupted genes	Nearby genes
21	N/A	3	gene: ENSG00000280441	gene: ENSG00000280441, MIR6724-3 (-54677), gene: ENSG00000274868 (-54143), gene: ENSG00000280614 (-48789), RNA5-8SN3 (-47371), gene: ENSG00000286267 (-24895), gene: ENSG00000286146 (-22127), gene: ENSG00000286054 (-21014), gene: ENSG00000286178 (-19059), MIR6724-4 (-10509), gene: ENSG00000278775 (-9956), gene: ENSG00000281181 (-4579), RNA5-8SN1 (-3155), gene: ENSG00000286149 (+19189), gene: ENSG00000286032 (+22123), gene: ENSG00000286091 (+23335), gene: ENSG00000286148 (+25189)

30927-007.R1 (paired) (Report)

Host chromosome	Unaligned ends virus	Broken reads host	Disrupted genes	Nearby genes
21	N/A	3	gene: ENSG00000280441	gene: ENSG00000280441, MIR6724-3 (-54691), gene: ENSG00000274868 (-54157), gene: ENSG00000280614 (-48803), RNA5-8SN3 (-47385), gene: ENSG00000286267 (-24909), gene: ENSG00000286146 (-22141), gene: ENSG00000286054 (-21028), gene: ENSG00000286178 (-19073), MIR6724-4 (-10523), gene: ENSG00000278775 (-9970), gene: ENSG00000281181 (-4593), RNA5-8SN1 (-3169), gene: ENSG00000286149 (+19175), gene: ENSG00000286032 (+22109), gene: ENSG00000286091 (+23321), gene: ENSG00000286148 (+25175)

30927-007.R1 (paired) (Report)

Host chromosome	Unaligned ends virus	Broken reads host	Disrupted genes	Nearby genes
21	N/A	3	gene: ENSG00000280441	gene: ENSG00000280441, MIR6724-3 (-54697), gene: ENSG00000274868 (-54163), gene: ENSG00000280614 (-48809), RNA5-8SN3 (-47391), gene: ENSG00000286267 (-24915), gene: ENSG00000286146 (-22147), gene: ENSG00000286054 (-21034), gene: ENSG00000286178 (-19079), MIR6724-4 (-10529), gene: ENSG00000278775 (-9976), gene: ENSG00000281181 (-4599), RNA5-8SN1 (-3175), gene: ENSG00000286149 (+19169), gene: ENSG00000286032 (+22103), gene: ENSG00000286091 (+23315), gene: ENSG00000286148 (+25169)

30927-007.R1 (paired) (Report)

Host chromosome	Unaligned ends virus	Broken reads host	Disrupted genes	Nearby genes
21	N/A	3	gene: ENSG00000280441	gene: ENSG00000280441, MIR6724-3 (-54721), gene: ENSG00000274868 (-54187), gene: ENSG00000280614 (-48833), RNA5-8SN3 (-47415), gene: ENSG00000286267 (-24939), gene: ENSG00000286146 (-22171), gene: ENSG00000286054 (-21058), gene: ENSG00000286178 (-19103), MIR6724-4 (-10553), gene: ENSG00000278775 (-10000), gene: ENSG00000281181 (-4623), RNA5-8SN1 (-3199), gene: ENSG00000286149 (+19145), gene: ENSG00000286032 (+22079), gene: ENSG00000286091 (+23291), gene: ENSG00000286148 (+25145)

30927-007.R1 (paired) (Report)

Host chromosome	Unaligned ends virus	Broken reads host	Disrupted genes	Nearby genes
21	N/A	3	gene: ENSG00000280441	gene: ENSG00000280441, MIR6724-3 (-54728), gene: ENSG00000274868 (-54194), gene: ENSG00000280614 (-48840), RNA5-8SN3 (-47422), gene: ENSG00000286267 (-24946), gene: ENSG00000286146 (-22178), gene: ENSG00000286054 (-21065), gene: ENSG00000286178 (-19110), MIR6724-4 (-10560), gene: ENSG00000278775 (-10007), gene: ENSG00000281181 (-4630), RNA5-8SN1 (-3206), gene: ENSG00000286149 (+19138), gene: ENSG00000286032 (+22072), gene: ENSG00000286091 (+23284), gene: ENSG00000286148 (+25138)

30927-007.R1 (paired) (Report)

Host chromosome	Unaligned ends virus	Broken reads host	Disrupted genes	Nearby genes
21	4,646	3	gene: ENSG00000280441	gene: ENSG00000280441, MIR6724-3 (-54739), gene: ENSG00000274868 (-54205), gene: ENSG00000280614 (-48851), RNA5-8SN3 (-47433), gene: ENSG00000286267 (-24957), gene: ENSG00000286146 (-22189), gene: ENSG00000286054 (-21076), gene: ENSG00000286178 (-19121), MIR6724-4 (-10571), gene: ENSG00000278775 (-10018), gene: ENSG00000281181 (-4641), RNA5-8SN1 (-3217), gene: ENSG00000286149 (+19127), gene: ENSG00000286032 (+22061), gene: ENSG00000286091 (+23273), gene: ENSG00000286148 (+25127)

30927-007.R1 (paired) (Report)

Host chromosome	Unaligned ends virus	Broken reads host	Disrupted genes	Nearby genes
21	4,646	3	gene: ENSG00000280441	gene: ENSG00000280441, MIR6724-3 (-54742), gene: ENSG00000274868 (-54208), gene: ENSG00000280614 (-48854), RNA5-8SN3 (-47436), gene: ENSG00000286267 (-24960), gene: ENSG00000286146 (-22192), gene: ENSG00000286054 (-21079), gene: ENSG00000286178 (-19124), MIR6724-4 (-10574), gene: ENSG00000278775 (-10021), gene: ENSG00000281181 (-4644), RNA5-8SN1 (-3220), gene: ENSG00000286149 (+19124), gene: ENSG00000286032 (+22058), gene: ENSG00000286091 (+23270), gene: ENSG00000286148 (+25124)

30927-007.R1 (paired) (Report)

Host chromosome	Unaligned ends virus	Broken reads host	Disrupted genes	Nearby genes
21	N/A	3	gene: ENSG00000280441	gene: ENSG00000280441, MIR6724-3 (-54743), gene: ENSG00000274868 (-54209), gene: ENSG00000280614 (-48855), RNA5-8SN3 (-47437), gene: ENSG00000286267 (-24961), gene: ENSG00000286146 (-22193), gene: ENSG00000286054 (-21080), gene: ENSG00000286178 (-19125), MIR6724-4 (-10575), gene: ENSG00000278775 (-10022), gene: ENSG00000281181 (-4645), RNA5-8SN1 (-3221), gene: ENSG00000286149 (+19123), gene: ENSG00000286032 (+22057), gene: ENSG00000286091 (+23269), gene: ENSG00000286148 (+25123)

30927-007.R1 (paired) (Report)

Host chromosome	Unaligned ends virus	Broken reads host	Disrupted genes	Nearby genes
21	N/A	3	gene: ENSG00000280441	gene: ENSG00000280441, MIR6724-3 (-54749), gene: ENSG00000274868 (-54215), gene: ENSG00000280614 (-48861), RNA5-8SN3 (-47443), gene: ENSG00000286267 (-24967), gene: ENSG00000286146 (-22199), gene: ENSG00000286054 (-21086), gene: ENSG00000286178 (-19131), MIR6724-4 (-10581), gene: ENSG00000278775 (-10028), gene: ENSG00000281181 (-4651), RNA5-8SN1 (-3227), gene: ENSG00000286149 (+19117), gene: ENSG00000286032 (+22051), gene: ENSG00000286091 (+23263), gene: ENSG00000286148 (+25117)

30927-007.R1 (paired) (Report)

Host chromosome	Unaligned ends virus	Broken reads host	Disrupted genes	Nearby genes
21	N/A	3	gene: ENSG00000280441	gene: ENSG00000280441, MIR6724-3 (-54751), gene: ENSG00000274868 (-54217), gene: ENSG00000280614 (-48863), RNA5-8SN3 (-47445), gene: ENSG00000286267 (-24969), gene: ENSG00000286146 (-22201), gene: ENSG00000286054 (-21088), gene: ENSG00000286178 (-19133), MIR6724-4 (-10583), gene: ENSG00000278775 (-10030), gene: ENSG00000281181 (-4653), RNA5-8SN1 (-3229), gene: ENSG00000286149 (+19115), gene: ENSG00000286032 (+22049), gene: ENSG00000286091 (+23261), gene: ENSG00000286148 (+25115)

30927-007.R1 (paired) (Report)

Host chromosome	Unaligned ends virus	Broken reads host	Disrupted genes	Nearby genes
21	N/A	3	gene: ENSG00000280441	gene: ENSG00000280441, MIR6724-3 (-54792), gene: ENSG00000274868 (-54258), gene: ENSG00000280614 (-48904), RNA5-8SN3 (-47486), gene: ENSG00000286267 (-25010), gene: ENSG00000286146 (-22242), gene: ENSG00000286054 (-21129), gene: ENSG00000286178 (-19174), MIR6724-4 (-10624), gene: ENSG00000278775 (-10071), gene: ENSG00000281181 (-4694), RNA5-8SN1 (-3270), gene: ENSG00000286149 (+19074), gene: ENSG00000286032 (+22008), gene: ENSG00000286091 (+23220), gene: ENSG00000286148 (+25074)

30927-007.R1 (paired) (Report)

Host chromosome	Unaligned ends virus	Broken reads host	Disrupted genes	Nearby genes
21	N/A	3	gene: ENSG00000280441	gene: ENSG00000280441, MIR6724-3 (-54798), gene: ENSG00000274868 (-54264), gene: ENSG00000280614 (-48910), RNA5-8SN3 (-47492), gene: ENSG00000286267 (-25016), gene: ENSG00000286146 (-22248), gene: ENSG00000286054 (-21135), gene: ENSG00000286178 (-19180), MIR6724-4 (-10630), gene: ENSG00000278775 (-10077), gene: ENSG00000281181 (-4700), RNA5-8SN1 (-3276), gene: ENSG00000286149 (+19068), gene: ENSG00000286032 (+22002), gene: ENSG00000286091 (+23214), gene: ENSG00000286148 (+25068)

30927-007.R1 (paired) (Report)

Host chromosome	Unaligned ends virus	Broken reads host	Disrupted genes	Nearby genes
21	N/A	2	gene: ENSG00000280441	gene: ENSG00000280441, MIR6724-3 (-54844), gene: ENSG00000274868 (-54310), gene: ENSG00000280614 (-48956), RNA5-8SN3 (-47538), gene: ENSG00000286267 (-25062), gene: ENSG00000286146 (-22294), gene: ENSG00000286054 (-21181), gene: ENSG00000286178 (-19226), MIR6724-4 (-10676), gene: ENSG00000278775 (-10123), gene: ENSG00000281181 (-4746), RNA5-8SN1 (-3322), gene: ENSG00000286149 (+19022), gene: ENSG00000286032 (+21956), gene: ENSG00000286091 (+23168), gene: ENSG00000286148 (+25022)

30927-007.R1 (paired) (Report)

Host chromosome	Unaligned ends virus	Broken reads host	Disrupted genes	Nearby genes
21	4,646	1	gene: ENSG00000280441	gene: ENSG00000280441, MIR6724-3 (-56657), gene: ENSG00000274868 (-56123), gene: ENSG00000280614 (-50769), RNA5-8SN3 (-49351), gene: ENSG00000286267 (-26875), gene: ENSG00000286146 (-24107), gene: ENSG00000286054 (-22994), gene: ENSG00000286178 (-21039), MIR6724-4 (-12489), gene: ENSG00000278775 (-11936), gene: ENSG00000281181 (-6559), RNA5-8SN1 (-5135), gene: ENSG00000286149 (+17209), gene: ENSG00000286032 (+20143), gene: ENSG00000286091 (+21355), gene: ENSG00000286148 (+23209)

30927-007.R1 (paired) (Report)

Host chromosome	Unaligned ends virus	Broken reads host	Disrupted genes	Nearby genes
21	4,646	8	gene: ENSG00000280441	gene: ENSG00000280441, MIR6724-3 (-57355), gene: ENSG00000274868 (-56821), gene: ENSG00000280614 (-51467), RNA5-8SN3 (-50049), gene: ENSG00000286267 (-27573), gene: ENSG00000286146 (-24805), gene: ENSG00000286054 (-23692), gene: ENSG00000286178 (-21737), MIR6724-4 (-13187), gene: ENSG00000278775 (-12634), gene: ENSG00000281181 (-7257), RNA5-8SN1 (-5833), gene: ENSG00000286149 (+16511), gene: ENSG00000286032 (+19445), gene: ENSG00000286091 (+20657), gene: ENSG00000286148 (+22511)

30927-007.R1 (paired) (Report)

Host chromosome	Unaligned ends virus	Broken reads host	Disrupted genes	Nearby genes
21	38,981	7	gene: ENSG00000280441	gene: ENSG00000280441, MIR6724-3 (-57366), gene: ENSG00000274868 (-56832), gene: ENSG00000280614 (-51478), RNA5-8SN3 (-50060), gene: ENSG00000286267 (-27584), gene: ENSG00000286146 (-24816), gene: ENSG00000286054 (-23703), gene: ENSG00000286178 (-21748), MIR6724-4 (-13198), gene: ENSG00000278775 (-12645), gene: ENSG00000281181 (-7268), RNA5-8SN1 (-5844), gene: ENSG00000286149 (+16500), gene: ENSG00000286032 (+19434), gene: ENSG00000286091 (+20646), gene: ENSG00000286148 (+22500)

30927-007.R1 (paired) (Report)

Host chromosome	Unaligned ends virus	Broken reads host	Disrupted genes	Nearby genes
21	N/A	7	gene: ENSG00000280441	gene: ENSG00000280441, MIR6724-3 (-57376), gene: ENSG00000274868 (-56842), gene: ENSG00000280614 (-51488), RNA5-8SN3 (-50070), gene: ENSG00000286267 (-27594), gene: ENSG00000286146 (-24826), gene: ENSG00000286054 (-23713), gene: ENSG00000286178 (-21758), MIR6724-4 (-13208), gene: ENSG00000278775 (-12655), gene: ENSG00000281181 (-7278), RNA5-8SN1 (-5854), gene: ENSG00000286149 (+16490), gene: ENSG00000286032 (+19424), gene: ENSG00000286091 (+20636), gene: ENSG00000286148 (+22490)

30927-007.R1 (paired) (Report)

Host chromosome	Unaligned ends virus	Broken reads host	Disrupted genes	Nearby genes
21	N/A	7	gene: ENSG00000280441	gene: ENSG00000280441, MIR6724-3 (-57389), gene: ENSG00000274868 (-56855), gene: ENSG00000280614 (-51501), RNA5-8SN3 (-50083), gene: ENSG00000286267 (-27607), gene: ENSG00000286146 (-24839), gene: ENSG00000286054 (-23726), gene: ENSG00000286178 (-21771), MIR6724-4 (-13221), gene: ENSG00000278775 (-12668), gene: ENSG00000281181 (-7291), RNA5-8SN1 (-5867), gene: ENSG00000286149 (+16477), gene: ENSG00000286032 (+19411), gene: ENSG00000286091 (+20623), gene: ENSG00000286148 (+22477)

30927-007.R1 (paired) (Report)

Host chromosome	Unaligned ends virus	Broken reads host	Disrupted genes	Nearby genes
21	N/A	6	gene: ENSG00000280441	gene: ENSG00000280441, MIR6724-3 (-57396), gene: ENSG00000274868 (-56862), gene: ENSG00000280614 (-51508), RNA5-8SN3 (-50090), gene: ENSG00000286267 (-27614), gene: ENSG00000286146 (-24846), gene: ENSG00000286054 (-23733), gene: ENSG00000286178 (-21778), MIR6724-4 (-13228), gene: ENSG00000278775 (-12675), gene: ENSG00000281181 (-7298), RNA5-8SN1 (-5874), gene: ENSG00000286149 (+16470), gene: ENSG00000286032 (+19404), gene: ENSG00000286091 (+20616), gene: ENSG00000286148 (+22470)

30927-007.R1 (paired) (Report)

Host chromosome	Unaligned ends virus	Broken reads host	Disrupted genes	Nearby genes
21	N/A	6	gene: ENSG00000280441	gene: ENSG00000280441, MIR6724-3 (-57407), gene: ENSG00000274868 (-56873), gene: ENSG00000280614 (-51519), RNA5-8SN3 (-50101), gene: ENSG00000286267 (-27625), gene: ENSG00000286146 (-24857), gene: ENSG00000286054 (-23744), gene: ENSG00000286178 (-21789), MIR6724-4 (-13239), gene: ENSG00000278775 (-12686), gene: ENSG00000281181 (-7309), RNA5-8SN1 (-5885), gene: ENSG00000286149 (+16459), gene: ENSG00000286032 (+19393), gene: ENSG00000286091 (+20605), gene: ENSG00000286148 (+22459)

30927-007.R1 (paired) (Report)

Host chromosome	Unaligned ends virus	Broken reads host	Disrupted genes	Nearby genes
21	N/A	6	gene: ENSG00000280441	gene: ENSG00000280441, MIR6724-3 (-57426), gene: ENSG00000274868 (-56892), gene: ENSG00000280614 (-51538), RNA5-8SN3 (-50120), gene: ENSG00000286267 (-27644), gene: ENSG00000286146 (-24876), gene: ENSG00000286054 (-23763), gene: ENSG00000286178 (-21808), MIR6724-4 (-13258), gene: ENSG00000278775 (-12705), gene: ENSG00000281181 (-7328), RNA5-8SN1 (-5904), gene: ENSG00000286149 (+16440), gene: ENSG00000286032 (+19374), gene: ENSG00000286091 (+20586), gene: ENSG00000286148 (+22440)

30927-007.R1 (paired) (Report)

Host chromosome	Unaligned ends virus	Broken reads host	Disrupted genes	Nearby genes
21	N/A	3	gene: ENSG00000280441	gene: ENSG00000280441, MIR6724-3 (-57459), gene: ENSG00000274868 (-56925), gene: ENSG00000280614 (-51571), RNA5-8SN3 (-50153), gene: ENSG00000286267 (-27677), gene: ENSG00000286146 (-24909), gene: ENSG00000286054 (-23796), gene: ENSG00000286178 (-21841), MIR6724-4 (-13291), gene: ENSG00000278775 (-12738), gene: ENSG00000281181 (-7361), RNA5-8SN1 (-5937), gene: ENSG00000286149 (+16407), gene: ENSG00000286032 (+19341), gene: ENSG00000286091 (+20553), gene: ENSG00000286148 (+22407)

30927-007.R1 (paired) (Report)

Host chromosome	Unaligned ends virus	Broken reads host	Disrupted genes	Nearby genes
21	41,362	3	gene: ENSG00000280441	gene: ENSG00000280441, MIR6724-3 (-57478), gene: ENSG00000274868 (-56944), gene: ENSG00000280614 (-51590), RNA5-8SN3 (-50172), gene: ENSG00000286267 (-27696), gene: ENSG00000286146 (-24928), gene: ENSG00000286054 (-23815), gene: ENSG00000286178 (-21860), MIR6724-4 (-13310), gene: ENSG00000278775 (-12757), gene: ENSG00000281181 (-7380), RNA5-8SN1 (-5956), gene: ENSG00000286149 (+16388), gene: ENSG00000286032 (+19322), gene: ENSG00000286091 (+20534), gene: ENSG00000286148 (+22388)

30927-007.R1 (paired) (Report)

Host chromosome	Unaligned ends virus	Broken reads host	Disrupted genes	Nearby genes
21	2,762	2	gene: ENSG00000280441	gene: ENSG00000280441, MIR6724-3 (-57503), gene: ENSG00000274868 (-56969), gene: ENSG00000280614 (-51615), RNA5-8SN3 (-50197), gene: ENSG00000286267 (-27721), gene: ENSG00000286146 (-24953), gene: ENSG00000286054 (-23840), gene: ENSG00000286178 (-21885), MIR6724-4 (-13335), gene: ENSG00000278775 (-12782), gene: ENSG00000281181 (-7405), RNA5-8SN1 (-5981), gene: ENSG00000286149 (+16363), gene: ENSG00000286032 (+19297), gene: ENSG00000286091 (+20509), gene: ENSG00000286148 (+22363)

30927-007.R1 (paired) (Report)

Host chromosome	Unaligned ends virus	Broken reads host	Disrupted genes	Nearby genes
21	N/A	1	gene: ENSG00000280441	gene: ENSG00000280441, MIR6724-3 (-57527), gene: ENSG00000274868 (-56993), gene: ENSG00000280614 (-51639), RNA5-8SN3 (-50221), gene: ENSG00000286267 (-27745), gene: ENSG00000286146 (-24977), gene: ENSG00000286054 (-23864), gene: ENSG00000286178 (-21909), MIR6724-4 (-13359), gene: ENSG00000278775 (-12806), gene: ENSG00000281181 (-7429), RNA5-8SN1 (-6005), gene: ENSG00000286149 (+16339), gene: ENSG00000286032 (+19273), gene: ENSG00000286091 (+20485), gene: ENSG00000286148 (+22339)

30927-007.R1 (paired) (Report)

Host chromosome	Unaligned ends virus	Broken reads host	Disrupted genes	Nearby genes
21	N/A	1	gene: ENSG00000280441	gene: ENSG00000280441, MIR6724-3 (-57528), gene: ENSG00000274868 (-56994), gene: ENSG00000280614 (-51640), RNA5-8SN3 (-50222), gene: ENSG00000286267 (-27746), gene: ENSG00000286146 (-24978), gene: ENSG00000286054 (-23865), gene: ENSG00000286178 (-21910), MIR6724-4 (-13360), gene: ENSG00000278775 (-12807), gene: ENSG00000281181 (-7430), RNA5-8SN1 (-6006), gene: ENSG00000286149 (+16338), gene: ENSG00000286032 (+19272), gene: ENSG00000286091 (+20484), gene: ENSG00000286148 (+22338)
21	4,646	846	gene: ENSG00000288578	gene: ENSG00000288555 (-39134), gene: ENSG00000288578, gene: ENSG00000280082 (+83123)
21	428	768	gene: ENSG00000288578	gene: ENSG00000288555 (-39139), gene: ENSG00000288578, gene: ENSG00000280082 (+83118)
21	2,762	668	gene: ENSG00000288578	gene: ENSG00000288555 (-39144), gene: ENSG00000288578, gene: ENSG00000280082 (+83113)

30927-007.R1 (paired) (Report)

Host chromosome	Unaligned ends virus	Broken reads host	Disrupted genes	Nearby genes
21	N/A	1		MIR3648-2 (-1385), gene: ENSG00000264063 (-1133), gene: ENSG00000279501 (+7932)
21	N/A	1	gene: ENSG00000278996, gene: ENSG00000280800	gene: ENSG00000278996, MIR6724-1 (-5694), gene: ENSG00000275664 (-5160), MIR3648-1 (-2448), gene: ENSG00000277437 (-2196), gene: ENSG00000280800, RNA5-8SN2 (+1471), gene: ENSG00000286155 (+24253), gene: ENSG00000286252 (+26548), gene: ENSG00000286057 (+27758), gene: ENSG00000286012 (+29635), MIR6724-2 (+38404), gene: ENSG00000277671 (+38959), gene: ENSG00000281383 (+43491), 5_8S_rRNA (+45680)

30927-007.R1 (paired) (Report)

Host chromosome	Unaligned ends virus	Broken reads host	Disrupted genes	Nearby genes
21	N/A	1	gene: ENSG00000278996, gene: ENSG00000280800	gene: ENSG00000278996, MIR6724-1 (-5732), gene: ENSG00000275664 (-5198), MIR3648-1 (-2486), gene: ENSG00000277437 (-2234), gene: ENSG00000280800, RNA5-8SN2 (+1433), gene: ENSG00000286155 (+24215), gene: ENSG00000286252 (+26510), gene: ENSG00000286057 (+27720), gene: ENSG00000286012 (+29597), MIR6724-2 (+38366), gene: ENSG00000277671 (+38921), gene: ENSG00000281383 (+43453), 5_8S_rRNA (+45642)
21	N/A	3	gene: ENSG00000278996	gene: ENSG00000278996, MIR6724-1 (-9801), gene: ENSG00000275664 (-9267), MIR3648-1 (-6555), gene: ENSG00000277437 (-6303), gene: ENSG00000280800 (-3901), RNA5-8SN2 (-2483), gene: ENSG00000286155 (+20146), gene: ENSG00000286252 (+22441), gene: ENSG00000286057 (+23651), gene: ENSG00000286012 (+25528), MIR6724-2 (+34297), gene: ENSG00000277671 (+34852), gene: ENSG00000281383 (+39384), 5_8S_rRNA (+41573)

30927-007.R1 (paired) (Report)

Host chromosome	Unaligned ends virus	Broken reads host	Disrupted genes	Nearby genes
21	N/A	3	gene: ENSG00000278996	gene: ENSG00000278996, MIR6724-1 (-9826), gene: ENSG00000275664 (-9292), MIR3648-1 (-6580), gene: ENSG00000277437 (-6328), gene: ENSG00000280800 (-3926), RNA5-8SN2 (-2508), gene: ENSG00000286155 (+20121), gene: ENSG00000286252 (+22416), gene: ENSG00000286057 (+23626), gene: ENSG00000286012 (+25503), MIR6724-2 (+34272), gene: ENSG00000277671 (+34827), gene: ENSG00000281383 (+39359), 5_8S_rRNA (+41548)
21	3,061	3	gene: ENSG00000278996	gene: ENSG00000278996, MIR6724-1 (-9830), gene: ENSG00000275664 (-9296), MIR3648-1 (-6584), gene: ENSG00000277437 (-6332), gene: ENSG00000280800 (-3930), RNA5-8SN2 (-2512), gene: ENSG00000286155 (+20117), gene: ENSG00000286252 (+22412), gene: ENSG00000286057 (+23622), gene: ENSG00000286012 (+25499), MIR6724-2 (+34268), gene: ENSG00000277671 (+34823), gene: ENSG00000281383 (+39355), 5_8S_rRNA (+41544)

30927-007.R1 (paired) (Report)

Host chromosome	Unaligned ends virus	Broken reads host	Disrupted genes	Nearby genes
21	N/A	3	gene: ENSG00000278996	gene: ENSG00000278996, MIR6724-1 (-9849), gene: ENSG00000275664 (-9315), MIR3648-1 (-6603), gene: ENSG00000277437 (-6351), gene: ENSG00000280800 (-3949), RNA5-8SN2 (-2531), gene: ENSG00000286155 (+20098), gene: ENSG00000286252 (+22393), gene: ENSG00000286057 (+23603), gene: ENSG00000286012 (+25480), MIR6724-2 (+34249), gene: ENSG00000277671 (+34804), gene: ENSG00000281383 (+39336), 5_8S_rRNA (+41525)
21	N/A	4	gene: ENSG00000278996	gene: ENSG00000278996, MIR6724-1 (-9869), gene: ENSG00000275664 (-9335), MIR3648-1 (-6623), gene: ENSG00000277437 (-6371), gene: ENSG00000280800 (-3969), RNA5-8SN2 (-2551), gene: ENSG00000286155 (+20078), gene: ENSG00000286252 (+22373), gene: ENSG00000286057 (+23583), gene: ENSG00000286012 (+25460), MIR6724-2 (+34229), gene: ENSG00000277671 (+34784), gene: ENSG00000281383 (+39316), 5_8S_rRNA (+41505)

30927-007.R1 (paired) (Report)

Host chromosome	Unaligned ends virus	Broken reads host	Disrupted genes	Nearby genes
21	N/A	4	gene: ENSG00000278996	gene: ENSG00000278996, MIR6724-1 (-9874), gene: ENSG00000275664 (-9340), MIR3648-1 (-6628), gene: ENSG00000277437 (-6376), gene: ENSG00000280800 (-3974), RNA5-8SN2 (-2556), gene: ENSG00000286155 (+20073), gene: ENSG00000286252 (+22368), gene: ENSG00000286057 (+23578), gene: ENSG00000286012 (+25455), MIR6724-2 (+34224), gene: ENSG00000277671 (+34779), gene: ENSG00000281383 (+39311), 5_8S_rRNA (+41500)
21	N/A	5	gene: ENSG00000278996	gene: ENSG00000278996, MIR6724-1 (-9898), gene: ENSG00000275664 (-9364), MIR3648-1 (-6652), gene: ENSG00000277437 (-6400), gene: ENSG00000280800 (-3998), RNA5-8SN2 (-2580), gene: ENSG00000286155 (+20049), gene: ENSG00000286252 (+22344), gene: ENSG00000286057 (+23554), gene: ENSG00000286012 (+25431), MIR6724-2 (+34200), gene: ENSG00000277671 (+34755), gene: ENSG00000281383 (+39287), 5_8S_rRNA (+41476)

30927-007.R1 (paired) (Report)

Host chromosome	Unaligned ends virus	Broken reads host	Disrupted genes	Nearby genes
21	N/A	5	gene: ENSG00000278996	gene: ENSG00000278996, MIR6724-1 (-9901), gene: ENSG00000275664 (-9367), MIR3648-1 (-6655), gene: ENSG00000277437 (-6403), gene: ENSG00000280800 (-4001), RNA5-8SN2 (-2583), gene: ENSG00000286155 (+20046), gene: ENSG00000286252 (+22341), gene: ENSG00000286057 (+23551), gene: ENSG00000286012 (+25428), MIR6724-2 (+34197), gene: ENSG00000277671 (+34752), gene: ENSG00000281383 (+39284), 5_8S_rRNA (+41473)
21	N/A	5	gene: ENSG00000278996	gene: ENSG00000278996, MIR6724-1 (-9903), gene: ENSG00000275664 (-9369), MIR3648-1 (-6657), gene: ENSG00000277437 (-6405), gene: ENSG00000280800 (-4003), RNA5-8SN2 (-2585), gene: ENSG00000286155 (+20044), gene: ENSG00000286252 (+22339), gene: ENSG00000286057 (+23549), gene: ENSG00000286012 (+25426), MIR6724-2 (+34195), gene: ENSG00000277671 (+34750), gene: ENSG00000281383 (+39282), 5_8S_rRNA (+41471)

30927-007.R1 (paired) (Report)

Host chromosome	Unaligned ends virus	Broken reads host	Disrupted genes	Nearby genes
21	N/A	5	gene: ENSG00000278996	gene: ENSG00000278996, MIR6724-1 (-9929), gene: ENSG00000275664 (-9395), MIR3648-1 (-6683), gene: ENSG00000277437 (-6431), gene: ENSG00000280800 (-4029), RNA5-8SN2 (-2611), gene: ENSG00000286155 (+20018), gene: ENSG00000286252 (+22313), gene: ENSG00000286057 (+23523), gene: ENSG00000286012 (+25400), MIR6724-2 (+34169), gene: ENSG00000277671 (+34724), gene: ENSG00000281383 (+39256), 5_8S_rRNA (+41445)
21	N/A	5	gene: ENSG00000278996	gene: ENSG00000278996, MIR6724-1 (-9980), gene: ENSG00000275664 (-9446), MIR3648-1 (-6734), gene: ENSG00000277437 (-6482), gene: ENSG00000280800 (-4080), RNA5-8SN2 (-2662), gene: ENSG00000286155 (+19967), gene: ENSG00000286252 (+22262), gene: ENSG00000286057 (+23472), gene: ENSG00000286012 (+25349), MIR6724-2 (+34118), gene: ENSG00000277671 (+34673), gene: ENSG00000281383 (+39205), 5_8S_rRNA (+41394)

30927-007.R1 (paired) (Report)

Host chromosome	Unaligned ends virus	Broken reads host	Disrupted genes	Nearby genes
21	N/A	5	gene: ENSG00000278996	gene: ENSG00000278996, MIR6724-1 (-9995), gene: ENSG00000275664 (-9461), MIR3648-1 (-6749), gene: ENSG00000277437 (-6497), gene: ENSG00000280800 (-4095), RNA5-8SN2 (-2677), gene: ENSG00000286155 (+19952), gene: ENSG00000286252 (+22247), gene: ENSG00000286057 (+23457), gene: ENSG00000286012 (+25334), MIR6724-2 (+34103), gene: ENSG00000277671 (+34658), gene: ENSG00000281383 (+39190), 5_8S_rRNA (+41379)
21	N/A	5	gene: ENSG00000278996	gene: ENSG00000278996, MIR6724-1 (-9996), gene: ENSG00000275664 (-9462), MIR3648-1 (-6750), gene: ENSG00000277437 (-6498), gene: ENSG00000280800 (-4096), RNA5-8SN2 (-2678), gene: ENSG00000286155 (+19951), gene: ENSG00000286252 (+22246), gene: ENSG00000286057 (+23456), gene: ENSG00000286012 (+25333), MIR6724-2 (+34102), gene: ENSG00000277671 (+34657), gene: ENSG00000281383 (+39189), 5_8S_rRNA (+41378)

30927-007.R1 (paired) (Report)

Host chromosome	Unaligned ends virus	Broken reads host	Disrupted genes	Nearby genes
21	N/A	5	gene: ENSG00000278996	gene: ENSG00000278996, MIR6724-1 (-10005), gene: ENSG00000275664 (-9471), MIR3648-1 (-6759), gene: ENSG00000277437 (-6507), gene: ENSG00000280800 (-4105), RNA5-8SN2 (-2687), gene: ENSG00000286155 (+19942), gene: ENSG00000286252 (+22237), gene: ENSG00000286057 (+23447), gene: ENSG00000286012 (+25324), MIR6724-2 (+34093), gene: ENSG00000277671 (+34648), gene: ENSG00000281383 (+39180), 5_8S_rRNA (+41369)
21	N/A	4	gene: ENSG00000278996	gene: ENSG00000278996, MIR6724-1 (-10041), gene: ENSG00000275664 (-9507), MIR3648-1 (-6795), gene: ENSG00000277437 (-6543), gene: ENSG00000280800 (-4141), RNA5-8SN2 (-2723), gene: ENSG00000286155 (+19906), gene: ENSG00000286252 (+22201), gene: ENSG00000286057 (+23411), gene: ENSG00000286012 (+25288), MIR6724-2 (+34057), gene: ENSG00000277671 (+34612), gene: ENSG00000281383 (+39144), 5_8S_rRNA (+41333)

30927-007.R1 (paired) (Report)

Host chromosome	Unaligned ends virus	Broken reads host	Disrupted genes	Nearby genes
21	N/A	4	gene: ENSG00000278996	gene: ENSG00000278996, MIR6724-1 (-10079), gene: ENSG00000275664 (-9545), MIR3648-1 (-6833), gene: ENSG00000277437 (-6581), gene: ENSG00000280800 (-4179), RNA5-8SN2 (-2761), gene: ENSG00000286155 (+19868), gene: ENSG00000286252 (+22163), gene: ENSG00000286057 (+23373), gene: ENSG00000286012 (+25250), MIR6724-2 (+34019), gene: ENSG00000277671 (+34574), gene: ENSG00000281383 (+39106), 5_8S_rRNA (+41295)
21	4,090	4	gene: ENSG00000278996	gene: ENSG00000278996, MIR6724-1 (-10299), gene: ENSG00000275664 (-9765), MIR3648-1 (-7053), gene: ENSG00000277437 (-6801), gene: ENSG00000280800 (-4399), RNA5-8SN2 (-2981), gene: ENSG00000286155 (+19648), gene: ENSG00000286252 (+21943), gene: ENSG00000286057 (+23153), gene: ENSG00000286012 (+25030), MIR6724-2 (+33799), gene: ENSG00000277671 (+34354), gene: ENSG00000281383 (+38886), 5_8S_rRNA (+41075)

30927-007.R1 (paired) (Report)

Host chromosome	Unaligned ends virus	Broken reads host	Disrupted genes	Nearby genes
21	4,090	2	gene: ENSG00000278996	gene: ENSG00000278996, MIR6724-1 (-10454), gene: ENSG00000275664 (-9920), MIR3648-1 (-7208), gene: ENSG00000277437 (-6956), gene: ENSG00000280800 (-4554), RNA5-8SN2 (-3136), gene: ENSG00000286155 (+19493), gene: ENSG00000286252 (+21788), gene: ENSG00000286057 (+22998), gene: ENSG00000286012 (+24875), MIR6724-2 (+33644), gene: ENSG00000277671 (+34199), gene: ENSG00000281383 (+38731), 5_8S_rRNA (+40920)
21	N/A	2	gene: ENSG00000278996	gene: ENSG00000278996, MIR6724-1 (-10453), gene: ENSG00000275664 (-9919), MIR3648-1 (-7207), gene: ENSG00000277437 (-6955), gene: ENSG00000280800 (-4553), RNA5-8SN2 (-3135), gene: ENSG00000286155 (+19494), gene: ENSG00000286252 (+21789), gene: ENSG00000286057 (+22999), gene: ENSG00000286012 (+24876), MIR6724-2 (+33645), gene: ENSG00000277671 (+34200), gene: ENSG00000281383 (+38732), 5_8S_rRNA (+40921)

30927-007.R1 (paired) (Report)

Host chromosome	Unaligned ends virus	Broken reads host	Disrupted genes	Nearby genes
21	N/A	2	gene: ENSG00000278996	gene: ENSG00000278996, MIR6724-1 (-10455), gene: ENSG00000275664 (-9921), MIR3648-1 (-7209), gene: ENSG00000277437 (-6957), gene: ENSG00000280800 (-4555), RNA5-8SN2 (-3137), gene: ENSG00000286155 (+19492), gene: ENSG00000286252 (+21787), gene: ENSG00000286057 (+22997), gene: ENSG00000286012 (+24874), MIR6724-2 (+33643), gene: ENSG00000277671 (+34198), gene: ENSG00000281383 (+38730), 5_8S_rRNA (+40919)
21	N/A	2	gene: ENSG00000278996	gene: ENSG00000278996, MIR6724-1 (-11001), gene: ENSG00000275664 (-10467), MIR3648-1 (-7755), gene: ENSG00000277437 (-7503), gene: ENSG00000280800 (-5101), RNA5-8SN2 (-3683), gene: ENSG00000286155 (+18946), gene: ENSG00000286252 (+21241), gene: ENSG00000286057 (+22451), gene: ENSG00000286012 (+24328), MIR6724-2 (+33097), gene: ENSG00000277671 (+33652), gene: ENSG00000281383 (+38184), 5_8S_rRNA (+40373)

30927-007.R1 (paired) (Report)

Host chromosome	Unaligned ends virus	Broken reads host	Disrupted genes	Nearby genes
21	4,646	2	gene: ENSG00000278996	gene: ENSG00000278996, MIR6724-1 (-11375), gene: ENSG00000275664 (-10841), MIR3648-1 (-8129), gene: ENSG00000277437 (-7877), gene: ENSG00000280800 (-5475), RNA5-8SN2 (-4057), gene: ENSG00000286155 (+18572), gene: ENSG00000286252 (+20867), gene: ENSG00000286057 (+22077), gene: ENSG00000286012 (+23954), MIR6724-2 (+32723), gene: ENSG00000277671 (+33278), gene: ENSG00000281383 (+37810), 5_8S_rRNA (+39999)
21	N/A	2	gene: ENSG00000278996	gene: ENSG00000278996, MIR6724-1 (-11379), gene: ENSG00000275664 (-10845), MIR3648-1 (-8133), gene: ENSG00000277437 (-7881), gene: ENSG00000280800 (-5479), RNA5-8SN2 (-4061), gene: ENSG00000286155 (+18568), gene: ENSG00000286252 (+20863), gene: ENSG00000286057 (+22073), gene: ENSG00000286012 (+23950), MIR6724-2 (+32719), gene: ENSG00000277671 (+33274), gene: ENSG00000281383 (+37806), 5_8S_rRNA (+39995)

30927-007.R1 (paired) (Report)

Host chromosome	Unaligned ends virus	Broken reads host	Disrupted genes	Nearby genes
21	N/A	4	gene: ENSG00000278996	gene: ENSG00000278996, MIR6724-1 (-13110), gene: ENSG00000275664 (-12576), MIR3648-1 (-9864), gene: ENSG00000277437 (-9612), gene: ENSG00000280800 (-7210), RNA5-8SN2 (-5792), gene: ENSG00000286155 (+16837), gene: ENSG00000286252 (+19132), gene: ENSG00000286057 (+20342), gene: ENSG00000286012 (+22219), MIR6724-2 (+30988), gene: ENSG00000277671 (+31543), gene: ENSG00000281383 (+36075), 5_8S_rRNA (+38264)
21	N/A	2	gene: ENSG00000278996	gene: ENSG00000278996, MIR6724-1 (-13124), gene: ENSG00000275664 (-12590), MIR3648-1 (-9878), gene: ENSG00000277437 (-9626), gene: ENSG00000280800 (-7224), RNA5-8SN2 (-5806), gene: ENSG00000286155 (+16823), gene: ENSG00000286252 (+19118), gene: ENSG00000286057 (+20328), gene: ENSG00000286012 (+22205), MIR6724-2 (+30974), gene: ENSG00000277671 (+31529), gene: ENSG00000281383 (+36061), 5_8S_rRNA (+38250)

30927-007.R1 (paired) (Report)

Host chromosome	Unaligned ends virus	Broken reads host	Disrupted genes	Nearby genes
21	N/A	2	gene: ENSG00000278996	gene: ENSG00000278996, MIR6724-1 (-13153), gene: ENSG00000275664 (-12619), MIR3648-1 (-9907), gene: ENSG00000277437 (-9655), gene: ENSG00000280800 (-7253), RNA5-8SN2 (-5835), gene: ENSG00000286155 (+16794), gene: ENSG00000286252 (+19089), gene: ENSG00000286057 (+20299), gene: ENSG00000286012 (+22176), MIR6724-2 (+30945), gene: ENSG00000277671 (+31500), gene: ENSG00000281383 (+36032), 5_8S_rRNA (+38221)
21	N/A	2	gene: ENSG00000278996	gene: ENSG00000278996, MIR6724-1 (-13179), gene: ENSG00000275664 (-12645), MIR3648-1 (-9933), gene: ENSG00000277437 (-9681), gene: ENSG00000280800 (-7279), RNA5-8SN2 (-5861), gene: ENSG00000286155 (+16768), gene: ENSG00000286252 (+19063), gene: ENSG00000286057 (+20273), gene: ENSG00000286012 (+22150), MIR6724-2 (+30919), gene: ENSG00000277671 (+31474), gene: ENSG00000281383 (+36006), 5_8S_rRNA (+38195)

30927-007.R1 (paired) (Report)

Host chromosome	Unaligned ends virus	Broken reads host	Disrupted genes	Nearby genes
21	4,646	2	gene: ENSG00000278996	gene: ENSG00000278996, MIR6724-1 (-13182), gene: ENSG00000275664 (-12648), MIR3648-1 (-9936), gene: ENSG00000277437 (-9684), gene: ENSG00000280800 (-7282), RNA5-8SN2 (-5864), gene: ENSG00000286155 (+16765), gene: ENSG00000286252 (+19060), gene: ENSG00000286057 (+20270), gene: ENSG00000286012 (+22147), MIR6724-2 (+30916), gene: ENSG00000277671 (+31471), gene: ENSG00000281383 (+36003), 5_8S_rRNA (+38192)
21	N/A	1	gene: ENSG00000278996	gene: ENSG00000278996, MIR6724-1 (-13202), gene: ENSG00000275664 (-12668), MIR3648-1 (-9956), gene: ENSG00000277437 (-9704), gene: ENSG00000280800 (-7302), RNA5-8SN2 (-5884), gene: ENSG00000286155 (+16745), gene: ENSG00000286252 (+19040), gene: ENSG00000286057 (+20250), gene: ENSG00000286012 (+22127), MIR6724-2 (+30896), gene: ENSG00000277671 (+31451), gene: ENSG00000281383 (+35983), 5_8S_rRNA (+38172)

30927-007.R1 (paired) (Report)

Host chromosome	Unaligned ends virus	Broken reads host	Disrupted genes	Nearby genes
21	N/A	1	gene: ENSG00000278996	gene: ENSG00000278996, MIR6724-1 (-13204), gene: ENSG00000275664 (-12670), MIR3648-1 (-9958), gene: ENSG00000277437 (-9706), gene: ENSG00000280800 (-7304), RNA5-8SN2 (-5886), gene: ENSG00000286155 (+16743), gene: ENSG00000286252 (+19038), gene: ENSG00000286057 (+20248), gene: ENSG00000286012 (+22125), MIR6724-2 (+30894), gene: ENSG00000277671 (+31449), gene: ENSG00000281383 (+35981), 5_8S_rRNA (+38170)
21	N/A	1	gene: ENSG00000278996	gene: ENSG00000278996, MIR6724-1 (-13210), gene: ENSG00000275664 (-12676), MIR3648-1 (-9964), gene: ENSG00000277437 (-9712), gene: ENSG00000280800 (-7310), RNA5-8SN2 (-5892), gene: ENSG00000286155 (+16737), gene: ENSG00000286252 (+19032), gene: ENSG00000286057 (+20242), gene: ENSG00000286012 (+22119), MIR6724-2 (+30888), gene: ENSG00000277671 (+31443), gene: ENSG00000281383 (+35975), 5_8S_rRNA (+38164)

30927-007.R1 (paired) (Report)

Host chromosome	Unaligned ends virus	Broken reads host	Disrupted genes	Nearby genes
21	N/A	1	gene: ENSG00000278996	gene: ENSG00000278996, MIR6724-1 (-13222), gene: ENSG00000275664 (-12688), MIR3648-1 (-9976), gene: ENSG00000277437 (-9724), gene: ENSG00000280800 (-7322), RNA5-8SN2 (-5904), gene: ENSG00000286155 (+16725), gene: ENSG00000286252 (+19020), gene: ENSG00000286057 (+20230), gene: ENSG00000286012 (+22107), MIR6724-2 (+30876), gene: ENSG00000277671 (+31431), gene: ENSG00000281383 (+35963), 5_8S_rRNA (+38152)
21	N/A	1	gene: ENSG00000278996	gene: ENSG00000278996, MIR6724-1 (-13232), gene: ENSG00000275664 (-12698), MIR3648-1 (-9986), gene: ENSG00000277437 (-9734), gene: ENSG00000280800 (-7332), RNA5-8SN2 (-5914), gene: ENSG00000286155 (+16715), gene: ENSG00000286252 (+19010), gene: ENSG00000286057 (+20220), gene: ENSG00000286012 (+22097), MIR6724-2 (+30866), gene: ENSG00000277671 (+31421), gene: ENSG00000281383 (+35953), 5_8S_rRNA (+38142)

30927-007.R1 (paired) (Report)

Host chromosome	Unaligned ends virus	Broken reads host	Disrupted genes	Nearby genes
21	N/A	1	gene: ENSG00000278996	gene: ENSG00000278996, MIR6724-1 (-13251), gene: ENSG00000275664 (-12717), MIR3648-1 (-10005), gene: ENSG00000277437 (-9753), gene: ENSG00000280800 (-7351), RNA5-8SN2 (-5933), gene: ENSG00000286155 (+16696), gene: ENSG00000286252 (+18991), gene: ENSG00000286057 (+20201), gene: ENSG00000286012 (+22078), MIR6724-2 (+30847), gene: ENSG00000277671 (+31402), gene: ENSG00000281383 (+35934), 5_8S_rRNA (+38123)
21	N/A	1	gene: ENSG00000278996	gene: ENSG00000278996, MIR6724-1 (-13253), gene: ENSG00000275664 (-12719), MIR3648-1 (-10007), gene: ENSG00000277437 (-9755), gene: ENSG00000280800 (-7353), RNA5-8SN2 (-5935), gene: ENSG00000286155 (+16694), gene: ENSG00000286252 (+18989), gene: ENSG00000286057 (+20199), gene: ENSG00000286012 (+22076), MIR6724-2 (+30845), gene: ENSG00000277671 (+31400), gene: ENSG00000281383 (+35932), 5_8S_rRNA (+38121)

30927-007.R1 (paired) (Report)

Host chromosome	Unaligned ends virus	Broken reads host	Disrupted genes	Nearby genes
21	41,362	1	gene: ENSG00000278996	gene: ENSG00000278996, MIR6724-1 (-13255), gene: ENSG00000275664 (-12721), MIR3648-1 (-10009), gene: ENSG00000277437 (-9757), gene: ENSG00000280800 (-7355), RNA5-8SN2 (-5937), gene: ENSG00000286155 (+16692), gene: ENSG00000286252 (+18987), gene: ENSG00000286057 (+20197), gene: ENSG00000286012 (+22074), MIR6724-2 (+30843), gene: ENSG00000277671 (+31398), gene: ENSG00000281383 (+35930), 5_8S_rRNA (+38119)
21	3,061	74		gene: ENSG00000288555 (-91298), gene: ENSG00000288578 (-31965), gene: ENSG00000280082 (+30959), gene: ENSG00000229425 (+53273)

30927-007.R1 (paired) (Report)

Host chromosome	Unaligned ends virus	Broken reads host	Disrupted genes	Nearby genes
21	N/A	1		gene: ENSG00000278996 (-26405), MIR6724-1 (-48645), gene: ENSG00000275664 (-48111), MIR3648-1 (-45399), gene: ENSG00000277437 (-45147), gene: ENSG00000280800 (-42745), RNA5-8SN2 (-41327), gene: ENSG00000286155 (-18219), gene: ENSG00000286252 (-16072), gene: ENSG00000286057 (-14959), gene: ENSG00000286012 (-13004), MIR6724-2 (-4455), gene: ENSG00000277671 (-3902), gene: ENSG00000281383 (+540), 5_8S_rRNA (+2729)

Table S4 Viral integration sites reports

30927-007.R1 (paired) (Report)

Host chromosome	Unaligned ends virus	Broken reads host	Disrupted genes	Nearby genes
21	N/A	1	gene: ENSG00000280441, gene: ENSG00000281181	gene: ENSG00000280441, MIR6724-3 (-50070), gene: ENSG00000274868 (-49536), gene: ENSG00000280614 (-44182), RNA5-8SN3 (-42764), gene: ENSG00000286267 (-20288), gene: ENSG00000286146 (-17520), gene: ENSG00000286054 (-16407), gene: ENSG00000286178 (-14452), MIR6724-4 (-5902), gene: ENSG00000278775 (-5349), gene: ENSG00000281181, RNA5-8SN1 (+1299), gene: ENSG00000286149 (+23796), gene: ENSG00000286032 (+26730), gene: ENSG00000286091 (+27942), gene: ENSG00000286148 (+29796)
21	41,362	172		gene: ENSG00000288555 (-31988), gene: ENSG00000288578 (+7038), gene: ENSG00000280082 (+90269)

30927-007.R1 (paired) (Report)

Host chromosome	Unaligned ends virus	Broken reads host	Disrupted genes	Nearby genes
21	N/A	3	gene: ENSG00000280441	gene: ENSG00000280441, MIR6724-3 (-53926), gene: ENSG00000274868 (-53392), gene: ENSG00000280614 (-48038), RNA5-8SN3 (-46620), gene: ENSG00000286267 (-24144), gene: ENSG00000286146 (-21376), gene: ENSG00000286054 (-20263), gene: ENSG00000286178 (-18308), MIR6724-4 (-9758), gene: ENSG00000278775 (-9205), gene: ENSG00000281181 (-3828), RNA5-8SN1 (-2404), gene: ENSG00000286149 (+19940), gene: ENSG00000286032 (+22874), gene: ENSG00000286091 (+24086), gene: ENSG00000286148 (+25940)

30927-007.R1 (paired) (Report)

Host chromosome	Unaligned ends virus	Broken reads host	Disrupted genes	Nearby genes
21	N/A	4	gene: ENSG00000280441	gene: ENSG00000280441, MIR6724-3 (-53968), gene: ENSG00000274868 (-53434), gene: ENSG00000280614 (-48080), RNA5-8SN3 (-46662), gene: ENSG00000286267 (-24186), gene: ENSG00000286146 (-21418), gene: ENSG00000286054 (-20305), gene: ENSG00000286178 (-18350), MIR6724-4 (-9800), gene: ENSG00000278775 (-9247), gene: ENSG00000281181 (-3870), RNA5-8SN1 (-2446), gene: ENSG00000286149 (+19898), gene: ENSG00000286032 (+22832), gene: ENSG00000286091 (+24044), gene: ENSG00000286148 (+25898)

30927-007.R1 (paired) (Report)

Host chromosome	Unaligned ends virus	Broken reads host	Disrupted genes	Nearby genes
21	N/A	7	gene: ENSG00000280441	gene: ENSG00000280441, MIR6724-3 (-54004), gene: ENSG00000274868 (-53470), gene: ENSG00000280614 (-48116), RNA5-8SN3 (-46698), gene: ENSG00000286267 (-24222), gene: ENSG00000286146 (-21454), gene: ENSG00000286054 (-20341), gene: ENSG00000286178 (-18386), MIR6724-4 (-9836), gene: ENSG00000278775 (-9283), gene: ENSG00000281181 (-3906), RNA5-8SN1 (-2482), gene: ENSG00000286149 (+19862), gene: ENSG00000286032 (+22796), gene: ENSG00000286091 (+24008), gene: ENSG00000286148 (+25862)

30927-007.R1 (paired) (Report)

Host chromosome	Unaligned ends virus	Broken reads host	Disrupted genes	Nearby genes
21	N/A	7	gene: ENSG00000280441	gene: ENSG00000280441, MIR6724-3 (-54005), gene: ENSG00000274868 (-53471), gene: ENSG00000280614 (-48117), RNA5-8SN3 (-46699), gene: ENSG00000286267 (-24223), gene: ENSG00000286146 (-21455), gene: ENSG00000286054 (-20342), gene: ENSG00000286178 (-18387), MIR6724-4 (-9837), gene: ENSG00000278775 (-9284), gene: ENSG00000281181 (-3907), RNA5-8SN1 (-2483), gene: ENSG00000286149 (+19861), gene: ENSG00000286032 (+22795), gene: ENSG00000286091 (+24007), gene: ENSG00000286148 (+25861)

30927-007.R1 (paired) (Report)

Host chromosome	Unaligned ends virus	Broken reads host	Disrupted genes	Nearby genes
21	N/A	8	gene: ENSG00000280441	gene: ENSG00000280441, MIR6724-3 (-54007), gene: ENSG00000274868 (-53473), gene: ENSG00000280614 (-48119), RNA5-8SN3 (-46701), gene: ENSG00000286267 (-24225), gene: ENSG00000286146 (-21457), gene: ENSG00000286054 (-20344), gene: ENSG00000286178 (-18389), MIR6724-4 (-9839), gene: ENSG00000278775 (-9286), gene: ENSG00000281181 (-3909), RNA5-8SN1 (-2485), gene: ENSG00000286149 (+19859), gene: ENSG00000286032 (+22793), gene: ENSG00000286091 (+24005), gene: ENSG00000286148 (+25859)

30927-007.R1 (paired) (Report)

Host chromosome	Unaligned ends virus	Broken reads host	Disrupted genes	Nearby genes
21	N/A	8	gene: ENSG00000280441	gene: ENSG00000280441, MIR6724-3 (-54008), gene: ENSG00000274868 (-53474), gene: ENSG00000280614 (-48120), RNA5-8SN3 (-46702), gene: ENSG00000286267 (-24226), gene: ENSG00000286146 (-21458), gene: ENSG00000286054 (-20345), gene: ENSG00000286178 (-18390), MIR6724-4 (-9840), gene: ENSG00000278775 (-9287), gene: ENSG00000281181 (-3910), RNA5-8SN1 (-2486), gene: ENSG00000286149 (+19858), gene: ENSG00000286032 (+22792), gene: ENSG00000286091 (+24004), gene: ENSG00000286148 (+25858)

30927-007.R1 (paired) (Report)

Host chromosome	Unaligned ends virus	Broken reads host	Disrupted genes	Nearby genes
21	N/A	6	gene: ENSG00000280441	gene: ENSG00000280441, MIR6724-3 (-54942), gene: ENSG00000274868 (-54408), gene: ENSG00000280614 (-49054), RNA5-8SN3 (-47636), gene: ENSG00000286267 (-25160), gene: ENSG00000286146 (-22392), gene: ENSG00000286054 (-21279), gene: ENSG00000286178 (-19324), MIR6724-4 (-10774), gene: ENSG00000278775 (-10221), gene: ENSG00000281181 (-4844), RNA5-8SN1 (-3420), gene: ENSG00000286149 (+18924), gene: ENSG00000286032 (+21858), gene: ENSG00000286091 (+23070), gene: ENSG00000286148 (+24924)
21	2,762	2,591	gene: ENSG00000288578	gene: ENSG00000288555 (-39274), gene: ENSG00000288578, gene: ENSG00000280082 (+82983)
21	41,362	2,594	gene: ENSG00000288578	gene: ENSG00000288555 (-39275), gene: ENSG00000288578, gene: ENSG00000280082 (+82982)
21	3,061	232	gene: ENSG00000288578	gene: ENSG00000288555 (-48826), gene: ENSG00000288578, gene: ENSG00000280082 (+73431), gene: ENSG00000229425 (+95745)

30927-008 (paired) (Report)

1. Identify Viral Integration Sites summary

Input reads	3,786,060
Host reads	2,347,009
Virus reads	512,961
Unmapped reads	926,090
Host reads (%)	61.99
Virus reads (%)	13.55
Unmapped reads (%)	24.46
Breakpoints identified	17
Viruses identified	21

2. Identify Viral Integration Sites virus content

Virus	Reads mapped
HE974383	45,342
HE974371	37,920
HE974376	31,251
LC519823	27,525
HE974374	22,549
LC365288	17,341
LC488828	16,818
HE974364	16,041
AP007263	15,298
AB778116	13,327
LC519817	10,694
HE974367	6,542
HE974381	2,417
LC753654	2,368
AB540582	1,323
LC519820	833
LC519794	695
HE974375	586
LC519811	561
LC519806	545
HE974365	525

3. Identify Viral Integration Sites breakpoint summary

Host chromosome	Host region	Virus	Virus region	Unaligned ends host
4	complement (72824512..72824513)	HE974383	1794..1795	45
4	72824684..72824685	HE974383	1791..1792	3,742
13	complement (91169306..91169307)	HE974371	2059..2060	456
16	complement (46400095..46400096)	LC519823	complement(246..247)	265
21	complement (8398676..8398677)	LC488828	complement(243..244)	351
21	complement (8398891..8398892)	HE974374	complement(1378.. 1379)	204
21	complement (8401698..8401699)	HE974371	1321..1322	360
21	complement (8442474..8442475)	HE974383	complement(996..997)	744
21	complement (8442648..8442649)	AB778116	complement(1998.. 1999)	460
21	complement (8445750..8445751)	HE974376	1403..1404	136
21	complement (8215211..8215212)	HE974374	complement(996..997)	808
21	8442460..8442461	LC365288	1345..1346	1,298
21	8446081..8446082	LC519817	1476..1477	373
Y	56696316..56696317	HE974383	1827..1828	77
Y	56742721..56742722	HE974383	1827..1828	77
Y	56703474..56703475	HE974383	1827..1828	68
Y	56728442..56728443	HE974383	1827..1828	67

Host chromosome	Unaligned ends virus	Broken reads host	Disrupted genes	Nearby genes
4	9,660	215		
4	29	440		
13	218	10		LINC00380 (-79713), LINC00379 (-37899)
16	777	35		

30927-008 (paired) (Report)

Host chromosome	Unaligned ends virus	Broken reads host	Disrupted genes	Nearby genes
21	21	1	gene: ENSG00000280441	gene: ENSG00000280441, MIR6724-3 (-10223), gene: ENSG00000274868 (-9689), gene: ENSG00000280614 (-4335), RNA5-8SN3 (-2917), gene: ENSG00000286267 (+19080), gene: ENSG00000286146 (+21998), gene: ENSG00000286054 (+23206), gene: ENSG00000286178 (+25083), MIR6724-4 (+33853), gene: ENSG00000278775 (+34408), gene: ENSG00000281181 (+38952), RNA5-8SN1 (+41146), gene: ENSG00000286149 (+63643), gene: ENSG00000286032 (+66577), gene: ENSG00000286091 (+67789), gene: ENSG00000286148 (+69643)

30927-008 (paired) (Report)

Host chromosome	Unaligned ends virus	Broken reads host	Disrupted genes	Nearby genes
21	130	1	gene: ENSG00000280441	gene: ENSG00000280441, MIR6724-3 (-10438), gene: ENSG00000274868 (-9904), gene: ENSG00000280614 (-4550), RNA5-8SN3 (-3132), gene: ENSG00000286267 (+18865), gene: ENSG00000286146 (+21783), gene: ENSG00000286054 (+22991), gene: ENSG00000286178 (+24868), MIR6724-4 (+33638), gene: ENSG00000278775 (+34193), gene: ENSG00000281181 (+38737), RNA5-8SN1 (+40931), gene: ENSG00000286149 (+63428), gene: ENSG00000286032 (+66362), gene: ENSG00000286091 (+67574), gene: ENSG00000286148 (+69428)

30927-008 (paired) (Report)

Host chromosome	Unaligned ends virus	Broken reads host	Disrupted genes	Nearby genes
21	112	1	gene: ENSG00000280441	gene: ENSG00000280441, MIR6724-3 (-13245), gene: ENSG00000274868 (-12711), gene: ENSG00000280614 (-7357), RNA5-8SN3 (-5939), gene: ENSG00000286267 (+16058), gene: ENSG00000286146 (+18976), gene: ENSG00000286054 (+20184), gene: ENSG00000286178 (+22061), MIR6724-4 (+30831), gene: ENSG00000278775 (+31386), gene: ENSG00000281181 (+35930), RNA5-8SN1 (+38124), gene: ENSG00000286149 (+60621), gene: ENSG00000286032 (+63555), gene: ENSG00000286091 (+64767), gene: ENSG00000286148 (+66621)

30927-008 (paired) (Report)

Host chromosome	Unaligned ends virus	Broken reads host	Disrupted genes	Nearby genes
21	187	3	gene: ENSG00000280441	gene: ENSG00000280441, MIR6724-3 (-54021), gene: ENSG00000274868 (-53487), gene: ENSG00000280614 (-48133), RNA5-8SN3 (-46715), gene: ENSG00000286267 (-24239), gene: ENSG00000286146 (-21471), gene: ENSG00000286054 (-20358), gene: ENSG00000286178 (-18403), MIR6724-4 (-9853), gene: ENSG00000278775 (-9300), gene: ENSG00000281181 (-3923), RNA5-8SN1 (-2499), gene: ENSG00000286149 (+19845), gene: ENSG00000286032 (+22779), gene: ENSG00000286091 (+23991), gene: ENSG00000286148 (+25845)

30927-008 (paired) (Report)

Host chromosome	Unaligned ends virus	Broken reads host	Disrupted genes	Nearby genes
21	27	1	gene: ENSG00000280441	gene: ENSG00000280441, MIR6724-3 (-54195), gene: ENSG00000274868 (-53661), gene: ENSG00000280614 (-48307), RNA5-8SN3 (-46889), gene: ENSG00000286267 (-24413), gene: ENSG00000286146 (-21645), gene: ENSG00000286054 (-20532), gene: ENSG00000286178 (-18577), MIR6724-4 (-10027), gene: ENSG00000278775 (-9474), gene: ENSG00000281181 (-4097), RNA5-8SN1 (-2673), gene: ENSG00000286149 (+19671), gene: ENSG00000286032 (+22605), gene: ENSG00000286091 (+23817), gene: ENSG00000286148 (+25671)

30927-008 (paired) (Report)

Host chromosome	Unaligned ends virus	Broken reads host	Disrupted genes	Nearby genes
21	61	1	gene: ENSG00000280441	gene: ENSG00000280441, MIR6724-3 (-57297), gene: ENSG00000274868 (-56763), gene: ENSG00000280614 (-51409), RNA5-8SN3 (-49991), gene: ENSG00000286267 (-27515), gene: ENSG00000286146 (-24747), gene: ENSG00000286054 (-23634), gene: ENSG00000286178 (-21679), MIR6724-4 (-13129), gene: ENSG00000278775 (-12576), gene: ENSG00000281181 (-7199), RNA5-8SN1 (-5775), gene: ENSG00000286149 (+16569), gene: ENSG00000286032 (+19503), gene: ENSG00000286091 (+20715), gene: ENSG00000286148 (+22569)

30927-008 (paired) (Report)

Host chromosome	Unaligned ends virus	Broken reads host	Disrupted genes	Nearby genes
21	187	1	gene: ENSG00000278996	gene: ENSG00000278996, MIR6724-1 (-9805), gene: ENSG00000275664 (-9271), MIR3648-1 (-6559), gene: ENSG00000277437 (-6307), gene: ENSG00000280800 (-3905), RNA5-8SN2 (-2487), gene: ENSG00000286155 (+20142), gene: ENSG00000286252 (+22437), gene: ENSG00000286057 (+23647), gene: ENSG00000286012 (+25524), MIR6724-2 (+34293), gene: ENSG00000277671 (+34848), gene: ENSG00000281383 (+39380), 5_8S_rRNA (+41569)

30927-008 (paired) (Report)

Host chromosome	Unaligned ends virus	Broken reads host	Disrupted genes	Nearby genes
21	64	1	gene: ENSG00000280441	gene: ENSG00000280441, MIR6724-3 (-54007), gene: ENSG00000274868 (-53473), gene: ENSG00000280614 (-48119), RNA5-8SN3 (-46701), gene: ENSG00000286267 (-24225), gene: ENSG00000286146 (-21457), gene: ENSG00000286054 (-20344), gene: ENSG00000286178 (-18389), MIR6724-4 (-9839), gene: ENSG00000278775 (-9286), gene: ENSG00000281181 (-3909), RNA5-8SN1 (-2485), gene: ENSG00000286149 (+19859), gene: ENSG00000286032 (+22793), gene: ENSG00000286091 (+24005), gene: ENSG00000286148 (+25859)

30927-008 (paired) (Report)

Host chromosome	Unaligned ends virus	Broken reads host	Disrupted genes	Nearby genes
21	58	1	gene: ENSG00000280441	gene: ENSG00000280441, MIR6724-3 (-57628), gene: ENSG00000274868 (-57094), gene: ENSG00000280614 (-51740), RNA5-8SN3 (-50322), gene: ENSG00000286267 (-27846), gene: ENSG00000286146 (-25078), gene: ENSG00000286054 (-23965), gene: ENSG00000286178 (-22010), MIR6724-4 (-13460), gene: ENSG00000278775 (-12907), gene: ENSG00000281181 (-7530), RNA5-8SN1 (-6106), gene: ENSG00000286149 (+16238), gene: ENSG00000286032 (+19172), gene: ENSG00000286091 (+20384), gene: ENSG00000286148 (+22238)
Y	1,886	18		
Y	1,886	12		
Y	1,886	9		
Y	1,886	7		

1. Identify Viral Integration Sites summary

Input reads	4,797,050
Host reads	3,356,627
Virus reads	190,584
Unmapped reads	1,249,839
Host reads (%)	69.97
Virus reads (%)	3.97
Unmapped reads (%)	26.05
Breakpoints identified	8
Viruses identified	54

2. Identify Viral Integration Sites virus content

Virus	Reads mapped
AB981580	12,135
LC516607	8,443
LC360507	6,541
LC155820	6,098
AY123041	5,495
LC516605	5,307
LC516606	4,703
AB819617	3,767
LC155816	3,635
AB819618	3,628
AB819615	3,610
AB819616	3,602
LC516608	3,568
LC170476	3,243
LC155818	2,969
LC155817	2,926
LC516610	2,769
AB540584	2,180
LC170475	1,995
LC516609	1,976
LC516604	1,623
LC535928	1,214

30927-009 (paired) (Report)

Virus	Reads mapped
LC535927	1,206
AB540583	1,186
LC456118	1,104
LC519805	1,053
LC170474	1,023
LC535946	885
LC535925	823
LC170477	751
LC753642	592
LC155815	573
LC456113	537
LC057377	521
AB644280	517
LC155814	476
AB644286	414
AB540585	394
LC753661	342
NC_003977	313
AB560662	308
LC753667	302
LC064755	287
AB644281	274
LC064754	266
LC456112	256
LC535923	243
AB644283	225
LC365290	222
LC519818	217
LC753646	215
LC535938	208
AB644284	200
LC753660	137

3. Identify Viral Integration Sites breakpoint summary

Host chromosome	Host region	Virus	Virus region	Unaligned ends host
1	complement (175455935.. 175455936)	AB819618	747..748	886

30927-009 (paired) (Report)

Host chromosome	Host region	Virus	Virus region	Unaligned ends host
2	complement (209878200.. 209878201)	LC456118	767..768	161
4	157701578.. 157701579	LC535946	complement(1822.. 1823)	423
8	complement (78187657..78187658)	AB819616	755..756	261
8	complement (121644447.. 121644448)	LC516609	complement(2942.. 2943)	325
17	24598818..24598819	AB644281	complement(575..576)	203
21	complement (8260089..8260090)	LC516607	complement(1859.. 1860)	252
21	complement (8442946..8442947)	AB819616	463..464	227

Host chromosome	Unaligned ends virus	Broken reads host	Disrupted genes	Nearby genes
1	23	9	TNR	TNR, TNR-IT1 (+82839)
2	22	18	UNC80	UNC80
4	171	46	gene: ENSG00000286133	gene: ENSG00000286133, gene: ENSG00000249275 (-34534), Y_RNA (+66434)
8	42	5		gene: ENSG00000254001 (-33744), PKIA-AS1 (+80979)
8	42	6	HAS2-AS1	HAS2 (-3007), HAS2-AS1, LINC02855 (+24486)
17	71	12		

30927-009 (paired) (Report)

Host chromosome	Unaligned ends virus	Broken reads host	Disrupted genes	Nearby genes
21	42	1		gene: ENSG00000278996 (-32443), MIR6724-1 (-54683), gene: ENSG00000275664 (-54149), MIR3648-1 (-51437), gene: ENSG00000277437 (-51185), gene: ENSG00000280800 (-48783), RNA5-8SN2 (-47365), gene: ENSG00000286155 (-24257), gene: ENSG00000286252 (-22110), gene: ENSG00000286057 (-20997), gene: ENSG00000286012 (-19042), MIR6724-2 (-10493), gene: ENSG00000277671 (-9940), gene: ENSG00000281383 (-4575), 5_8S_rRNA (-3156)

30927-009 (paired) (Report)

Host chromosome	Unaligned ends virus	Broken reads host	Disrupted genes	Nearby genes
21	27	1	gene: ENSG00000280441	gene: ENSG00000280441, MIR6724-3 (-54493), gene: ENSG00000274868 (-53959), gene: ENSG00000280614 (-48605), RNA5-8SN3 (-47187), gene: ENSG00000286267 (-24711), gene: ENSG00000286146 (-21943), gene: ENSG00000286054 (-20830), gene: ENSG00000286178 (-18875), MIR6724-4 (-10325), gene: ENSG00000278775 (-9772), gene: ENSG00000281181 (-4395), RNA5-8SN1 (-2971), gene: ENSG00000286149 (+19373), gene: ENSG00000286032 (+22307), gene: ENSG00000286091 (+23519), gene: ENSG00000286148 (+25373)

30927-010.R1 (paired) (Report)

1. Identify Viral Integration Sites summary

Input reads	1,262,190
Host reads	735
Virus reads	574,346
Unmapped reads	687,109
Host reads (%)	0.06
Virus reads (%)	45.50
Unmapped reads (%)	54.44
Breakpoints identified	0
Viruses identified	6

2. Identify Viral Integration Sites virus content

Virus	Reads mapped
K03455	129,713
NC_001802	124,885
AY173951	34,621
AY423387	6,642
AF377956	5,645
AJ249238	3,646

3. Identify Viral Integration Sites breakpoint summary

SRR8670572 (Report)

1. Identify Viral Integration Sites summary

Input reads	1,128,512
Host reads	1,069,235
Virus reads	52,616
Unmapped reads	6,661
Host reads (%)	94.75
Virus reads (%)	4.66
Unmapped reads (%)	0.59
Breakpoints identified	2
Viruses identified	7

2. Identify Viral Integration Sites virus content

Virus	Reads mapped
K03455	14,225
NC_001802	13,049
AY173951	3,127
AY331295	806
AY423387	592
U88824	376
K03454	246

3. Identify Viral Integration Sites breakpoint summary

Host chromosome	Host region	Virus	Virus region	Unaligned ends host
7	complement (33019786..33019787)	K03455	complement(9085.. 9086)	47
7	33019791..33019792	K03455	9719..9720	65

Host chromosome	Unaligned ends virus	Broken reads host	Disrupted genes	Nearby genes
7	46	326	NT5C3A	FKBP9 (-12856), RNU6-388P (-17732), NT5C3A, RN7SL505P (+26011), RP9 (+75010), BBS9 (+89770)

SRR8670572 (Report)

Host chromosome	Unaligned ends virus	Broken reads host	Disrupted genes	Nearby genes
7	N/A	367	NT5C3A	FKBP9 (-12861), RNU6-388P (-17737), NT5C3A, RN7SL505P (+26006), RP9 (+75005), BBS9 (+89765)
